# The smut fungi of Greenland

**DOI:** 10.3897/mycokeys.64.47380

**Published:** 2020-03-05

**Authors:** Teodor T. Denchev, Henning Knudsen, Cvetomir M. Denchev

**Affiliations:** 1 Institute of Biodiversity and Ecosystem Research, Bulgarian Academy of Sciences, 23 Acad. G. Bonchev St., 1113 Sofia, Bulgaria; 2 IUCN SSC Rusts and Smuts Specialist Group, Sofia, Bulgaria; 3 Natural History; 4 Museum of Denmark, Øster Voldgade 5–7, 1350 Copenhagen, Denmark

**Keywords:** Anthracoidea, Arctic fungi, Arctic–alpine fungi, Microbotryum, Schizonella, Stegocintractia, Urocystis, Ustilentyloma pleuropogonis

## Abstract

The first taxonomic treatment of the smut fungi in Greenland is provided. A total of 43 species in 11 genera are treated and illustrated by photographs of sori, microphotographs of spores in LM and SEM, and distribution maps. Two species, Anthracoidea pseudofoetidae and Urocystis tothii, are recorded as new from North America. Thirteen species, Anthracoidea altera, A. capillaris, A. limosa, A. liroi, A. pseudofoetidae, A. scirpoideae, A. turfosa, Microbotryum lagerheimii, M. stellariae, Schizonella elynae, Stegocintractia luzulae, Urocystis fischeri, and U. tothii, are reported for the first time from Greenland. Three new fungus-host combinations, Anthracoidea capillaris on Carex boecheriana, Anthracoidea pseudofoetidae on Carex maritima, and Urocystis tothii on Juncus biglumis, are given. Five plant species are reported as new hosts of smut fungi in Greenland, namely, Carex nigra for Anthracoidea heterospora, C. canescens for Anthracoidea karii, C. fuliginosa subsp. misandra for Anthracoidea misandrae, C. maritima for Orphanomyces arcticus, and C. fuliginosa subsp. misandra for Schizonella melanogramma. Three species, Microbotryum violaceum s. str. (recorded as ‘Ustilago violacea’), Urocystis anemones, and U. junci, which were previously reported from Greenland, are considered wrongly identified. Additional distribution records are given for 12 species from Greenland: Anthracoidea bigelowii, A. caricis, A. elynae, A. lindebergiae, A. misandrae, A. nardinae, A. rupestris, A. scirpi, Schizonella melanogramma, Stegocintractia hyperborea, Urocystis agropyri, and U. sorosporioides. The most numerous distribution groups are the following: circumpolar–alpine and Arctic–alpine species – 14; circumboreal–polar species – 10; and circumpolar and Arctic species – 6. The most widely distributed smut fungi in Greenland were Anthracoidea bigelowii, A. elynae, Microbotryum bistortarum, and M. vinosum. Most species were found in the High Arctic zone (29 species), while from the Low Arctic zone and the Subarctic zone, 26 and 19 species were known, respectively. Ten species, Anthracoidea bigelowii, A. capillaris, A. elynae, Microbotryum bistortarum, M. koenigiae, M. pustulatum, M. silenes-acaulis, M. vinosum, Schizonella elynae, and Urocystis sorosporioides, were recorded from all three zones. Only plants belonging to six families, Cyperaceae, Poaceae, Juncaceae, Ranunculaceae, Caryophyllaceae, and Polygonaceae, out of a total of 55 in the flora of Greenland, hosted smut fungi. Cyperaceae was the plant family with most host species (23). Carex was the genus with the highest number of host species (22). The total number of the host plants (45 species) was 8.5 % out of a total of 532 vascular plants in the flora of Greenland. A new combination in Carex, C. macroprophylla subsp. subfilifolia, is proposed for Kobresia filifolia subsp. subfilifolia.

## Historical outline of investigations of smut fungi of Greenland

Collecting smut fungi in Greenland started during an expedition to southern East Greenland led by Captain Graah (1828–1829) to explore this very inhospitable and difficult to access coast. Among the scientists was a young botanist, Jens Vahl, son of the eminent botanist Martin Vahl. Jens Vahl recorded two species of smut fungi. He classified all collections on Carex as Ustilago caricis, as was customary in those days. However, modern determination of his Carex collections has revealed six species of Anthracoidea. His other smut was the very common Microbotryum bistortarum on Bistorta vivipara. Later, E. Rostrup (1831–1907) published the records of Vahl. Rostrup was a botanist, but gradually became a plant pathologist of international standing.

At the end of the 19^th^ century, many expeditions sailed from Denmark to Greenland, encouraged by the recently formed Greenland Commission to explore the island and look for possibilities for hunting, fishing, mining, and other economically interesting enterprises. Collecting plants, which at that time included fungi, was among the duties for many of the expeditions.

During the Fylla-expedition to western Greenland in 1884, Professor E. Warming and the botanist T. Holm collected a few smut fungi. Warming encouraged Rostrup to study the material, which later led to Rostrup surveying the herbarium of Greenlandic plants in Copenhagen. The librarian, later professor of phycology, L. Kolderup Rosenvinge, also collected a few specimens of smut fungi and a student, N. Hartz, collected three more. They were collecting in SW Greenland, except for Hartz, who collected north to Sisimiut. All these expeditions, which had purposes other than collecting fungi, added eight more species to the known smut fungi from Greenland. From this period, the Swedish mycologist Thore M. Fries (1871, Anthracoidea nardinae), the German botanist E. Vanhöffen (1893, Entyloma microsporum), and H.G. Simmons (1899, Anthracoidea elynae) from Scotland each contributed by collecting one more new species of smut fungi for Greenland. By the end of the 19^th^ century, 19 species had been collected, but only seven were published (Rostrup 1888, 1891, 1894, 1904; Allescher and Hennings 1897).

Early in the 20^th^ century, the Danish activities in Greenland decreased, before increasing again in the 1920s and 1930s. Expeditions to survey Greenland geology were led by Lauge Koch in NW Greenland, during which one of his companions, I. Noe-Nygaard, collected Tilletia cerebrina. In an awkward way, an international development caused the first real boom in the collection of smut fungi. For a period, Norwegian hunters had settled in central East Greenland to make a living out of hunting and fishing. They lived in an area from ca 71°30' to 75°40'N. It was practically uninhabited due to the severe climate, but was part of the island of Greenland and therefore considered to be under Danish-Greenlandic jurisdiction. Contrary to this, the Norwegian hunters claimed that this was uninhabited land and therefore open to colonization. The Norwegian hunters gradually got the Norwegian government interested in their case to include this part of Greenland under Norwegian rule. In 1931, Norway declared the land Norwegian. The Danish government immediately took the Norwegian government to the International Court in Haag, which in 1933 declared a continued Danish-Greenlandic sovereignty of the area. Due to the dispute, the area became the focus for new visitors and expeditions for the two governments to mark their presence. In this way, international politics boosted the knowledge of smut fungi in Greenland.

Of special importance for the investigation of smut fungi was a group headed by Norwegian botanist Asbjørn Hagen, accompanied by J. Vaage, B. Bjørlykke, S. Aandstad, P.F. Scholander, and J. Devold. In the period 1929–1933, the group made 166 collections of smut fungi, including a few found during perusal of herbarium holdings. Details of these collections were published by Hagen in 1941 and 1947 as 12 species (actually, 21 species based on modern taxonomy of Anthracoidea). Most notable were their records of Haradaea nivalis, Anthracoidea caricis s. lat., Schizonella melanogramma, Stegocintractia hyperborea, and Urocystis triseti. Urocystis tothii was also collected by Hagen, but only identified in the present study. A collection by Bjørlykke was later identified by D.B.O. Savile as Anthracoidea verrucosa.

A few other notable records are from the same period. The Danish lichenologist P. Gelting found Anthracoidea altera (1946), which was identified in the current study. In this period, the Danish pharmacist J. Lind contributed a number of papers on micromycetes from Arctic areas, and in one of these he published Anthracoidea scirpi, one of the species also collected by Vahl, but identified by Rostrup under the collective name Ustilago caricis.

After World War II, there was significant development in science and technology, universities grew and new, modern methods were applied to the study of smut fungi. The invention of the scanning electron microscope allowed detailed study of the spore wall ornamentation. At the same time, a change in the view of species concepts spread among taxonomists, and a number of Scandinavian mycologists started an intense study of the smut fungi on Cyperaceae. The Finnish mycologist J.I. Liro (1872–1943) had for many years before the war collected smuts and other parasitic fungi and gathered them in his Mycotheca Fennica. His studies pioneered and stimulated other Scandinavian mycologists (J.-A. Nannfeldt, B. Lindeberg, I. Kukkonen) and the Canadian (D.B.O. Savile) to collect and study smut fungi. They published comprehensive papers on the smut fungi of northern Europe and Canada.

As a part of these studies, Greenlandic material was often used for comparison with material from other countries. Savile, Nannfeldt, Kukkonen, and Jørstad revised Greenlandic specimens and in the period 1957–1979, nine new species for Greenland were reported, mainly due to the splitting of Anthracoidea caricis (Nannfeldt and Lindeberg 1957, 1965; Kukkonen 1963, 1964, 1965, 1969; Nannfeldt 1977, 1979).

Most recently, Henning Knudsen, Torbjørn Borgen, and Steen A. Elborne collected basidiomycetes in Greenland in 2016–2018 for a forthcoming funga of Arctic and Alpine basidiomycetes. They collected in three areas: (i) Kangerlussuaq–Sisimiut, (ii) Constable Pynt on Jameson Land, and (iii) Narsarsuaq–Kangilinnguit–Kobbefjord. Their collections included 50 specimens of smuts, containing 16 species. Two were new to Greenland, but were also found when unidentified collections by J. Vahl and T. Læssøe were re-examined.

Two authors of the present treatment (T.T.D. & C.M.D.) went through the herbarium of vascular Greenlandic plants in Copenhagen, holding approximately 200 000 sheets. They repeated Rostrup’s method and examined the plants known to be hosts for smuts. The result was surprisingly good, considering that the infected parts of the plants were very small and therefore unnoticed by the collecting botanist. One hundred and twenty-one collections from 26 species were found, including many inconspicuous species difficult to see unless their occurrence was suspected. Among them, seven species were new to Greenland. They also found another eight species among the unidentified collections made by previous collectors.

## Vegetation and main habitats

Greenland is the world’s largest island stretching 2500 km from Cape Farewell (59°45'N) in the south to the northernmost land in the world, Cape Morris Jesup (83°39'N). From West (Cape Alexander 73°3'W) there is 1000 km to Nordostrundingen (11°19'W) in the East. This is at the same time the easternmost point of the North American continent.

In spite of this huge area, the climate is rather uniform, strongly influenced by the northern position, the vast interior cover of Inland Ice, and influence from drift ice from the Arctic Ocean.

Most of Greenland belongs to the Arctic zone, defined by the average temperature for the warmest month being < 10 °C. This vast part is divided into Low Arctic and High Arctic, following 70°N passing through central Disko Island in the West to Jameson Land in the East. The distance between the West coast and the East coast of Greenland is separated by 200–1000 km of permanent ice, which should be considered in any comparison between the two sides. Another important point is the drift ice, making the Eastern side of Greenland colder than the Western side, and consequently only inhabited in two settlements, Ammassalik in the southern part and Ittoqqoormiut in the central part of the coast, separated by 800 km!

Alnus crispa (Aiton) Pursh forms restricted shrubs in the SW part of Greenland and continues to a much larger scale in Canada. Several other shrubs occur over most of Greenland. Salix is represented by five species of which S. uva-ursi Pursh has the same distribution as A. crispa, whereas S. arctophila Cockerell is distributed over most of western Greenland. S. glauca subsp. callicarpaea (Trautv.) Böch., S. herbacea L., and S. arctica Pall. have a much wider, circumpolar distribution. Betula glandulosa Michx. forms extensive, 0.25–1 m high shrubs in SW Greenland, being gradually replaced by B. nana L. continuing up to 75°N.

In the western part of South Greenland, the deep fjords have a subarctic climate at the bottom. They have characteristic, well-developed copses or very locally even a kind of forest of Betula pubescens Ehrh. s. lat. In protected valleys, the trees may reach 8–9 m in height and the trunks can be ca 30 cm in diam., but this type of copse is restricted to a few km^2^. The species itself reaches ca 63°N.

Apart from the climate, the soil has an important impact on the distribution of plants and fungi in Greenland. The Greenland geology is complicated. A large part has a bedrock of acidic granite, but locally and especially in central Greenland, a broad band of calcareous rocks and soil is found on both the Western and Eastern side around 70°N (Disko Island and Jameson Land). In this area a number of calcicolous species are present.

A phytogeographical division of Greenland was proposed by Feilberg (1984), Bay (1992), Fredskild (1996) and Bay (in prep.). Each of these deals with a specific part of Greenland, viz. South, North, West, and East. The division is mainly based on the distribution of vascular plants and placed where the largest number of northern or southern distributional limits occur.

South Greenland ranges from Cape Farewell, 59°45'N to 62°20'N, which is just south of the large glacier Frederikshåb Isblink. South of this area many plants have their northern limit in Greenland. The flora province is divided into six subzones by oceanity versus continentality and Low Arctic versus Subarctic. Feilberg (1984) concluded from his data that Nathorst’s (1890) view of southern Greenland as a province more related to the East than to the West as seen by the occurring plants was correct. In South Greenland flora province 346 species of vascular plants occur, the richest area being around Narsarsuaq with 309 species.

West Greenland ranges from 62°20'N to 79°30'N, with a subdivision from 74°N to 79°30'N, the Northwest Floristic Province. On the eastern side, there is a similar Northeast Floristic Province, a part of East Greenland, from 79°30'N to 74°N. In the West Greenland flora province 390 species are found, being 379 species up to 74°N (Fredskild 1996) and another 11 up to 79°30'N. An important distributional line goes through the middle of Disko Island where 62 species have their northern limit, and 18 species have their southern limit. This is by far the most important floristic boundary in western Greenland and marks the limit between High Arctic and Low Arctic.

The phytogeographical province North Greenland ranges from the northernmost point in Greenland, Cape Morris Jesup, 83°39'N south to 79°30'N. In western Greenland, the boundary is the Humboldt Glacier, in eastern Greenland Lambert Land. Only 121 vascular plants are known from this province.

East Greenland stretches from 79°30'N and south to 62°20'N. An analysis of this region is in preparation (Bay pers. comm.).

The Greenland plant habitats are often difficult to recognize and characterize. The habitats gradually merge into one another or are mixed with each other.

Copses include species of Betula, Salix, and Alnus. Occasionally also Juniperus communis subsp. alpina (Neilr.) Čelak and Sorbus groenlandica (Schneid.) Löve & Löve may be present, but usually only as scattered trees/bushes. In the copses the ground is covered by grasses and Angelica archangelica subsp. norvegica (Rupr.) Nordh. is common along with Bartsia alpina L., Hieracium ssp., Ranunculus ssp., and other herbs.

Herbslopes are formed on south-exposed slopes with a good supply of water. They are often conspicuous in the landscape containing plants with large flowers and low shrubs. Typical species are Rhodiola rosea L., Angelica archangelica subsp. norvegica, Alchemilla vulgaris L. s. lat., Veronica alpina L., V. fruticans Jacq., Saxifraga spp., Thalictrum alpinum L., Oxyria digyna (L.) Hill, and Platanthera hyperborea (L.) Lindl.

Snowbeds are formed where the snow persists for a long time into the summer, leaving a patch of bare soil well watered from the melting snow and without much competition from other plants. Most plants here are small, e.g. Harrimanella hypnoides (L.) Coville, Sibbaldia procumbens L., Oxyria digyna, Taraxacum spp., Carex bigelowii Torr., and Koenigia islandica L.

Grassland slopes and steppe are formed in flat, dry, open areas on clay or sand and characterized by many different herbs, such as Potentilla pulchella R. Br., P. nivea L., Puccinellia spp., Gentiana nivalis L., G. aurea L., Braya thorild-wulfii Ostf., B. linearis Rouy, B. purpurascens (R. Br.) Bge., Plantago maritima subsp. borealis (Lge.) Blytt & Dahl, Deschampsia flexuosa (L.) Trin., Calamagrostis purpurascens R. Br., Juncus biglumis L., Carex myosuroides Vill., and C. nardina Fr.

Dwarf shrub heaths are dominated by low shrubs of Betula nana L. or B. glandulosa Michx., Salix glauca L., and S. arctophila Cockerell, species from the heather family like Empetrum nigrum subsp. hermaphroditum (Hagerup) Böcher, Vaccinium uliginosum L., V. vitis-idaea subsp. minus (Lodd., G. Lodd. & W. Lodd.) Hultén, Arctostaphylos uva-ursi (L.) Spreng., A. alpina (L.) Spreng., Ledum groenlandicum Oeder, L. palustre subsp. decumbens (Aiton) Hultén, in northern areas also Cassiope tetragona (L.) D. Don. In moist hollows grows Pinguicula vulgaris L., Platanthera hyperborea (L.) Lindl., Trichophorum cespitosum (L.) Hartm., Silene uralensis (Rupr.) Bocquet, and Carex misandra R. Br.

Fell-fields are open, windswept areas on stony ground, including areas with polygons and solifluction. The unstable conditions and rough exposure are strong limiting factors for plants and only a few are found, like Koenigia islandica L., Papaver radicatum Rottb., Saxifraga tricuspidata Rottb., Draba alpina L., Sagina intermedia Fenzl, Viscaria alpina (L.) G. Don, Silene acaulis (L.) Jacq., and Carex nardina Fr.

Dunes and beach vegetation are found mainly along the rocky shores in protected places on riverbanks and in deltas where rivers flow into the sea. In the sandy dunes grow Elymus spp., Cochlearia groenlandica L., Plantago maritima subsp. borealis (Lge.) Blytt & Dahl, and Carex glareosa Wbg.

Fens and marshes of different nature occur in low-lying places, with many species of Cyperaceae (including Eriophorum spp., Trichophorum cespitosum, Carex spp.), of Juncaceae, as well as Ranunculus spp., Calamagrostis neglecta (Ehrh.) P. Gaertn. et al., and Comarum palustre L.

## Delimitation of Greenland

There is no generally accepted delimitation of Greenland. The proposed divisions vary considerably, as they are prepared for different purposes (e.g., Feilberg 1984; Bay 1992; Yurtsev 1994; Higgins 2010; Daniëls et al. 2013; Walker et al. 2016; Elven et al. 2018).

**Figure 1.**
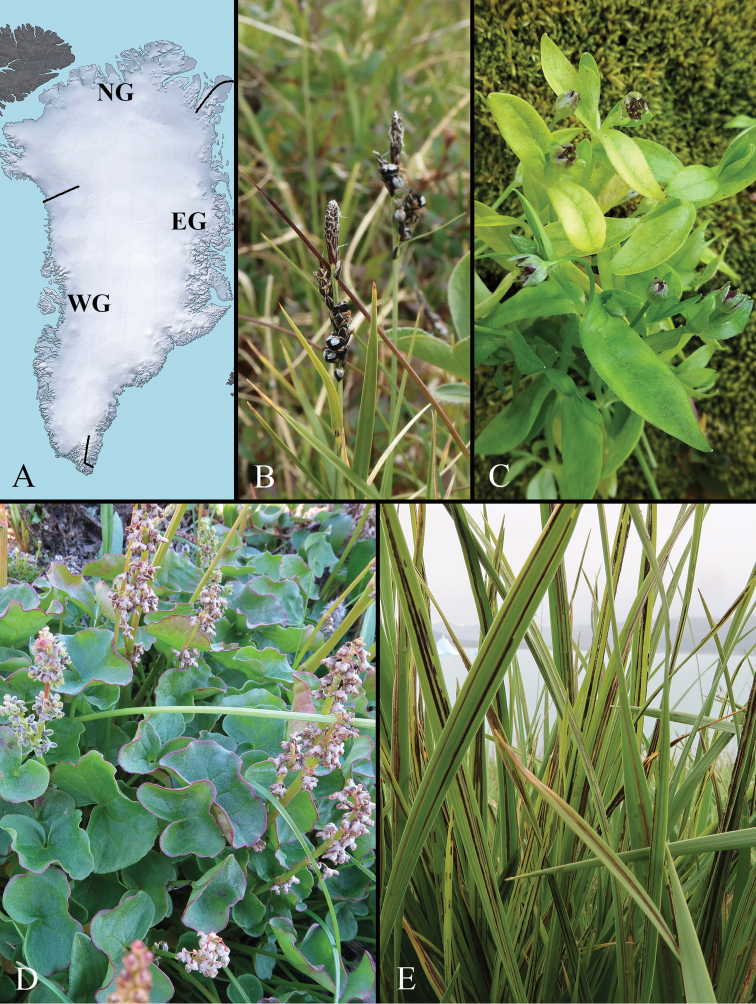
Smut fungi of Greenland **A** subdivision of Greenland into three regions: North (NG), West (WG), and East (EG) **B**Anthracoidea bigelowii on Carex bigelowii (C-F-105073) **C**Microbotryum stellariae on Stellaria calycantha (C-F-108447) **D**Microbotryum vinosum on Oxyria digyna (C-F-104902) **E**Urocystis agropyri on Leymus arenarius (C-F-111316). Photos **B–E** H. Knudsen.

For this study, Greenland is divided into three regions (Fig. 1A), following a delimitation applied for the *Panarctic Flora* project (PAF – Elven et al. 2018); the only exception being the location of the boundary between North and East Greenland:

**North Greenland** (as part of Ellesmere Land – Northern Greenland Region) includes the northernmost parts of Greenland from Melville Bay in the west (ca 75°25'N) to Nordostrundingen in the east (at 81°26'N). The boundary between North Greenland and East Greenland chosen here follows the SW–NE-trending watershed in Crown Prince Christian Land (after Ostenfeld 1926: 21; Higgins 2010), while the boundary proposed in PAF lies north of the glaciers between Germania Land and Lambert Land.

**West Greenland** includes western and southernmost Greenland to Lindenow Fjord (ca 60°30'N) in SE Greenland.

**East Greenland** – from Nordostrundingen to Lindenow Fjord.

The examined and/or recorded specimens of fungi are arranged from north to south (first from the western side to Lindenow Fjord in the southeast, then from the eastern part).

## Materials and methods

This study is based on examination of specimens from the following dried reference collections: **C** – Natural History Museum of Denmark, University of Copenhagen, Copenhagen, Denmark; **DAOM** – Canadian National Mycological Herbarium, Ottawa, Canada; **E** – Royal Botanic Garden Edinburgh, Edinburgh, U.K.; **GZU** – Karl-Franzens-Universität Graz, Graz, Austria; **K(M)** – Kew Fungarium, Royal Botanic Gardens, Kew, U.K.; **O** – Botanical Museum, University of Oslo, Oslo, Norway; **S** – Swedish Museum of Natural History, Stockholm, Sweden; **U** – Utrecht, now transferred to **L** (Naturalis, Leiden, the Netherlands), but curated as a separate collection; **WSP** – Washington State University, Pullman, Washington, U.S.A.

Dried specimens were examined using a stereo zoom microscope (for observation of sori), light microscope (LM), and scanning electron microscope (SEM). For LM observations and measurements, spores were mounted in lactoglycerol solution (w : la : gl = 1 : 1 : 2) on glass slides, gently heated to boiling point to rehydrate the spores, and then cooled. The measurements of spores are given as min–max (extreme values) (mean ± 1 standard deviation). For SEM, spores were attached to specimen holders by double-sided adhesive tape and coated with platinum or gold in an ion sputter. The surface structure of spores was observed and photographed at 10 kV accelerating voltage using a JEOL JSM 6610-LV scanning electron microscope (Natural History Museum Vienna) and Hitachi SU3500 (National Museum of Natural History, Paris). The type of spore ornamentation and height of ornamenting elements (warts, spines, striae, muri) were analyzed and measured in SEM. The height of ornamenting elements was additionally measured in Olympus BX-51 (in magnification ×2000, through an image analysis software). In the case of verruculose or verrucose spore ornamentation, the type of ornamentation was defined in accordance with Denchev et al. (2013: 10). The descriptions below are based entirely on the specimens examined. The shapes of spores are arranged in descending order of frequency.

Spore size ranges of the Canadian Anthracoidea species were discussed by Savile (1952) and assigned by him to one of the following groups: (i) small-sized spores, 13–21(–23) × 9–17(–20) µm, (ii) medium-sized spores, 15–25(–27) × 10–21 µm, and (iii) large-sized, 18–33 × 13–28 µm. In this case, spore width is of very little use. Spore length of the known 111 species of Anthracoidea was analyzed by us and the following modification of Savile’s system for spore length ranges of species in this genus is suggested herein (Table 1).

**Table 1.** Spore length ranges of the species in Anthracoidea.

Spore length ranges	Length (µm)
very small-sized	9–13
small-sized	(11–)13–21
medium-sized	15–25(–27)
large-sized	18–33(–40)

Geographic distribution of the fungi is arranged by country from west to east then north to south.

Throughout the taxonomic section, Anthracoidea is abbreviated as ‘*A.*’, Carex as ‘C.’, Cintractia as ‘*Ci.*’, Tuburcinia as ‘*Tub.*’, Urocystis as ‘*Ur.*’, and Ustilago as ‘*U.*’. The regions of Greenland are abbreviated as follows: **NG** = North Greenland, **WG** = West Greenland, and **EG** = East Greenland.

On the plates with illustrations, scale bars on macrophotographs represent 0.5 cm, those of LM and SEM microphotographs 10 µm and 5 µm, respectively, and of maps 500 km.

## Taxonomic treatment

### Key to the genera of smut fungi in Greenland, based on host plant families

**On Caryophyllaceae**

**Table:** 

1	Sori in anthers	** Microbotryum **
1*	Sori in ovules	** Haradaea **

**On Cyperaceae**

**Table:** 

1	Sori as black crusts on the leaf surface	** Orphanomyces **
1*	Sori not so	**2**
2	Sori in female flowers, around aborted nuts; spores single	**3**
2*	Sori in leaves as long striae between the veins; spores in pairs or in balls	**4**
3	Spores with a thick-walled equatorial band and two, thin-walled polar areas	** Planetella **
3*	Spores without an equatorial band	** Anthracoidea **
4	Spores joined in pairs	** Schizonella **
4*	Spores in balls; spore balls composed of one to several, pigmented, fertile spores, surrounded by a layer of paler sterile cells	** Urocystis **

**On Juncaceae**

**Table:** 

1	Sori in spikelets; spores single	** Stegocintractia **
1*	Sori in leaves as long striae between the veins; spores in balls; spore balls composed of one to several, pigmented, fertile spores, surrounded by a layer of paler sterile cells	** Urocystis **

**On Poaceae**

**Table:** 

1	Sori in ovaries; spores single	** Tilletia **
1*	Sori in leaves as long striae between the veins; spores in balls; spore balls composed of one to several, pigmented, fertile spores, surrounded by a layer of paler sterile cells	** Urocystis **

**On Polygonaceae**

**Table:** 

Sori in stems, leaves, flowers or inflorescences; spores single		** Microbotryum **

**On Ranunculaceae**

**Table:** 

1	Sori in leaves and petioles as hard pustules or swellings; spores single	** Entyloma **
1*	Sori in leaves as long striae between the veins; spores in balls; spore balls composed of one to several, pigmented, fertile spores, surrounded by a layer of paler sterile cells	** Urocystis **

### Anthracoidea Bref., Unters. Gesammtgeb. Mykol. 12: 143, 1895. ≡ Cintractia*sensu* ampl. Magnus, Verh. Bot. Vereins Prov. Brandenburg 37: 78, 1896, non Cintractia Cornu, Ann. Sci. Nat., Bot., Sér. 6, 15: 279, 1883 (sensu str. orig.). — Type: A. caricis (Pers.) Bref.

= Cintractiomyxa Golovin, Bot. Mater. Otd. Sporov. Rast. Bot. Inst. Akad. Nauk S.S.S.R. 8: 108, 1952. — Type: C. caricis Golovin.

**Sori** in and around ovaries of cyperaceous plants (in the cases of Carex – scattered in female spikes or in female flowers of mixed spikes), usually partly hidden by the glumes; as globose, subglobose, broadly ellipsoidal or ovoid, rarely ellipsoidal, black, hard bodies; composed of the remainder of the nut in the center and a spore mass around it (spore formation on the outer surface of the nut); originally covered by a thin, white, grayish or silvery peridium of fungal cells, hyphae, and fragments of host cells, which ruptures exposing the spore mass. **Spore mass** initially firmly agglutinated, later powdery or semi-agglutinated on the surface, less often mature sori agglutinated on the surface and breaking into small, irregular pieces; composed only of teliospores, sterile cells absent. **Spores** formed singly, usually flattened, in plane view more or less regular in outline (orbicular, suborbicular or broadly elliptical) or more or less irregular, relatively large (compared to the spores of other genera of smut fungi); surrounded by a gelatinous sheath that breaks down at maturity (sometimes mature spores with remnants of that gelatinous sheath). Spore wall unevenly or evenly thickened; rarely with pale, thinner-walled polar regions; with or without protuberances, often with internal swellings and/or light-refractive areas, usually ornamented with warts, rarely punctate or smooth, few species with coarse (up to 2 μm high), irregular ornaments, apically flattened and slightly enlarged. **Spore germination** results in a two-celled aerial basidium forming one or more basidiospores on each cell; basidiospores globose, subglobose, ovoid or cylindrical. **Anamorph** present in some species. **Host-parasite interaction** (after Vánky 2013) by intracellular hyphae, coated by an electron-opaque matrix. Mature **septa** (after Vánky 2013) poreless.

Initially, the sori are covered by a thin membrane. Mature teliospores are liberated and dispersed by wind after the membrane ruptures. At an early stage of plant flowering, spores germinate, produce basidiospores, and infect flowers. The infection is local, floral (confined to individual flowers). Hyphae are localized in the ovaries and walls of single nuts. The spores are produced on the outer nut surface (Kukkonen 1963; Kukkonen and Vatanen 1968; Vánky 2002). Infected flowers do not form seeds as the ovaries are destroyed.

Based on the type of spore germination, the genus Anthracoidea is divided into two subgenera. Anthracoidea subgen. Anthracoidea is characterized by small to medium-sized spores (13–25 µm long) and globose, subglobose or ovoid basidiospores, up to 30 μm long, several produced per basidial cell. Anthracoidea subgen. Proceres Kukkonen is characterized by medium to large spores (22–37 μm long) and cylindrical basidiospores, 40–90 μm long, only one produced per basidial cell (Kukkonen 1963; Vánky 2011). Unfortunately, the type of spore germination is known only for some of the species, making it difficult to refer all of the species to a subgenus.

The genus Anthracoidea comprises **111** species. It is a cosmopolitan genus, but more widely distributed in Temperate, Subarctic, and Arctic regions of the Northern Hemisphere.

The species of Anthracoidea are restricted to host plants belonging to the same or closely related sections of Carex (Vánky 1979).

#### Key to the relevant Anthracoidea species, based on host plant taxonomy (arranged in genera and sections)

The host plants that occur in Greenland are given in square brackets.

**Table:** 

**On Carex**		
**On sect. Acrocystis.** [On C. deflexa]		**A. caricis** s. lat.
**On sect. Aulocystis**		
1	Spores small-sized, up to 21 µm long, spore mean length 17.0 ± 1.2 µm; spore wall minutely verruculose, warts up to 0.2(–0.3) μm high, spore profile not affected or very slightly affected. [On C. fuliginosa subsp. misandra	**A. altera**
1*	Spores medium-sized, up to 25 µm long, spore mean length 21.2 ± 1.2 µm; spore wall moderately verruculose to verrucose, warts up to 0.5(–0.6) µm high, spore profile affected. [On C. atrofusca, C. fuliginosa subsp. misandra]	**A. misandrae**
**On Bicolores–Paniceae clade.** [On C. vaginata]		**A. paniceae** s. lat.
**On sect. Chlorostachyae.** [On C. boecheriana, C. capillaris]		**A. capillaris**
**On sect. Foetidae.** [On C. maritima]		**A. pseudofoetidae**
**On sect. Glareosae.** [On C. brunnescens, C. canescens]		**A. karii** s. str.
**On sect. Limosae.** [On C. rariflora]		**A. limosa**
**On Myosuroides clade.** [On C. myosuroides]		**A. elynae**
**On sect. Nardinae**. [On C. nardina s. lat., C. nardina subsp. hepburnii]		**A. nardinae**
**On *sect.*Ovales**. [On C. macloviana var. macloviana]		**A. verrucosa**
**On *sect.*Phacocystis**		
1	Spores 12–21.5 µm long, spore mean length up to 18.5 µm; spore germination of Anthracoidea-type. [On hybrids of C. bigelowii and on C. nigra]	**A. heterospora**
1*	Spores 16–28 µm long, spore mean length usually higher; spore germination of Proceres-type	2
2	Spore mean length less than 20.5 µm; internal swellings absent; spore wall moderately verruculose, warts up to 0.4(–0.5) μm high, spore profile affected. [On C. bigelowii (and its hybrids), C. concolor]	**A. bigelowii**
2*	Spore mean length more than 20.5 µm; occasionally a weak internal swelling present; spore wall minutely verruculose, warts up to 0.2(–0.3) µm high, spore profile not affected or sometimes very slightly affected. [On C. subspathacea]	**A. liroi**
**On sect. Physoglochin.** [On C. parallela subsp. parallela]		**A. turfosa**
**On sect. Rupestres.** [On C. rupestris subsp. rupestris]		**A. rupestris** s. str.
**On sect. Scirpinae.** [On C. scirpoidea subsp. scirpoidea]		**A. scirpoideae**
**On Simpliciuscula clade.** [On C. simpliciuscula]		**A. lindebergiae**
**On Trichophorum**		
[On T. cespitosum subsp. cespitosum]		**A. scirpi**

#### 1(1) Anthracoidea altera Nannf., Symb. Bot. Upsal. 22(3): 12, 1979. — Holotype on Carex fuliginosa subsp. misandra (as ‘C. misandra’), Finland, Lapponia enontekiensis, Kilpisjärvi, Mt. Saana, 26 Jul 1957, leg. I. Kukkonen (H).

Fig. 2A–F

**Infection** local. **Sori** in some female flowers, around aborted nuts as ovoid, broadly ellipsoidal or ellipsoidal hard bodies, 1.0–1.5 mm long, initially covered by a thin, grayish peridium that later flakes away exposing a black spore mass, powdery on the surface. **Spores** small-sized, flattened, in plane view irregularly rounded, irregularly subpolygonal, broadly elliptical or suborbicular in outline, sometimes with a small protuberance, in plane view (14–)15–20(–21) × (12.5–)13.5–17(–18) (17.0 ± 1.2 × 15.3 ± 1.1) μm (n/_2_ = 200), in side view 9–12 μm thick, medium reddish brown; wall slightly unevenly thickened, 0.8–1.4(–1.6) μm thick, sometimes with 1–2(–3) weak internal swellings, sometimes with light refractive areas; surface minutely verruculose, warts up to 0.2(–0.3) μm high, spore profile not affected or very slightly affected. In SEM warts sometimes partly confluent, forming short rows or small groups. **Spore germination** of Anthracoidea-type (after Kukkonen 1963), resulting in a two-celled basidium, producing short and ovoid basidiospores.

**Figure 2.**
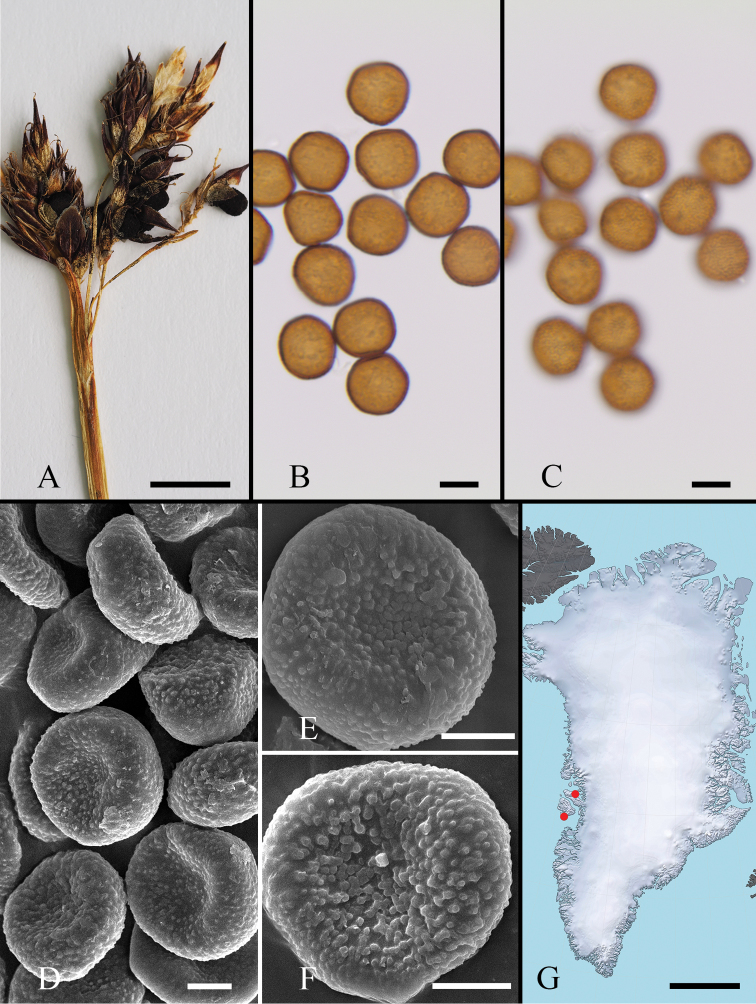
Anthracoidea altera on Carex fuliginosa subsp. misandra (P. Gelting, s.n., C-Greenland herb., s.n.). **A** habit **B, C** spores in LM, in median and surface view, respectively **D–F** spores in SEM**G** distribution map. Scale bars: 0.5 cm (**A**); 10 µm (**B, C**); 5 µm (**D–F**); 500 km (**G**).

**Hosts and distribution within the studied area** — On Cyperaceae: Carex sect. Aulocystis: Carex fuliginosa subsp. misandra (C. misandra R. Br.) – West Greenland (Fig. 2G).

**Specimens examined or recorded.**

On **Carex fuliginosa** subsp. **misandra** (R. Br.) Nyman:

**WG**, Avannaata, Nuussuaq Peninsula (as ‘Nûgssuaq Pen.’), Kûtsiaq, 70°40'N, 52°27'W, 10 Aug 1947, leg. T. Sørensen, The Danish Botanical Expedition to West Greenland 1947, no. 8810 (C-Greenland herb.!, s.n., the host as ‘C. misandra’).

**WG**, Disko Island, Qeqertarsuaq (as ‘Godhavn’), Stubben, ca 69°N, 53°W, 30 Jul 1946, leg. P. Gelting, s.n. (C-Greenland herb.!, s.n., the host as ‘C. misandra’); **ditto**, Lyngmarken, N of Qeqertarsuaq, ca 69°15'N, alt. 50–320 m, July 1983, leg. J. Poelt & H. Ullrich, s.n. (GZU 000323448!, the host as ‘C. misandra’).

**Known hosts** — On Cyperaceae: Carex sect. Aulocystis Dumort.: Carex fuliginosa subsp. misandra.

**General distribution. Arctic Europe**: Svalbard, Finland. **North America**: Canada, Greenland.

**Comments** — Dietrich (1967) recognized two subspecies of Carex fuliginosa, distributed in the alpine and Arctic regions of Europe, respectively. The specimens from mountains of Central and Southeast Europe are treated as belonging to subsp. fuliginosa, while those from the Arctic region are referred to subsp. misandra (= C. misandra). This infraspecific scheme is accepted in many recent sources (e.g., *Flora Europaea*, Chater 1980; *Euro+Med PlantBase*, Jiménez-Mejías and Luceño 2011; *Panarctic Flora*, Elven et al. 2018) and is applied herein. In the *Flora of North America* treatment (Ball and Mastrogiuseppe 2002), however, Carex misandra is considered to be conspecific with C. fuliginosa.

Carex fuliginosa subsp. misandra is a circumpolar taxon, distributed in northernmost Fennoscandia (restricted), Svalbard, Franz Joseph Land, Russian Arctic, Siberia, Russian Far East, Alaska, Canada (reaching southwards in the mountains of western U.S.A.), and Greenland (Hultén and Fries 1986: 517; Bay 1992; Fredskild 1996; Egorova 1999; Elven et al. 2018). Although the host plant is considered as frequent in the Arctic, Anthracoidea altera is known only from a few localities: Finland (Mt. Saana, Nannfeldt 1979), Svalbard (Spitsbergen — Lomfjorden, Hagen 1950b, as ‘Ci. caricis’; Isfjorden, Kukkonen 1963, as ‘A. misandrae’), and Canada (Baffin Island, Frobisher Bay, Kukkonen 1963, as ‘A. misandrae’; Nannfeldt 1979), and it seems that this smut fungus is a rare species. Its northernmost collection is reported from Spitsbergen (Lomfjorden), at ca 79°23'N. Anthracoidea altera is an Arctic species that is recorded here for the first time from Greenland.

It is worth noting that A. altera is not found on Carex fuliginosa subsp. fuliginosa, while A. misandrae is known to infect both subspecies of C. fuliginosa.

#### 2(2) Anthracoidea bigelowii Nannf., in Nannfeldt and Lindeberg, Svensk Bot. Tidskr. 59: 203, 1965. ≡ Cintractia limosa var. minor Savile, Canad. J. Bot. 30: 426, 1952, non C. minor (G.P. Clinton) H.S. Jacks., Mycologia 12: 153, 1920 (q.e. C. limitata G.P. Clinton). — Holotype on Carex bigelowii, Canada, Quebec, Great Whale River, 28 Jul 1949, leg. D.B.O. Savile, no. 536 (DAOM 28197).

Figs 1B, 3A–F

**Infection** local. **Sori** in some female flowers, around aborted nuts as subglobose to ovoid hard bodies, 0.8–1.5 mm long, initially covered by a thin, grayish peridium that later flakes away exposing a black spore mass, powdery on the surface. **Spores** medium-sized, flattened, in plane view suborbicular, broadly elliptical, irregularly rounded or ovate in outline, in plane view (16–)17–22(–23.5) × (14.5–)15.5–20(–21) (19.4 ± 1.1 × 17.6 ± 1.0) μm (n/_3_ = 300), in side view 10.5–13.5 μm thick, medium or dark reddish brown; wall slightly unevenly thickened, 1.0–1.7(–2.0) μm thick, internal swellings, light refractive areas, and protuberances absent; moderately verruculose, warts up to 0.4(–0.5) μm high, spore profile affected. In SEM warts densely packed, often partly confluent, forming short rows or small groups. **Spore germination** of Proceres-type (after Nannfeldt and Lindeberg 1965), resulting in a two-celled basidium, producing long and cylindrical basidiospores.

**Figure 3.**
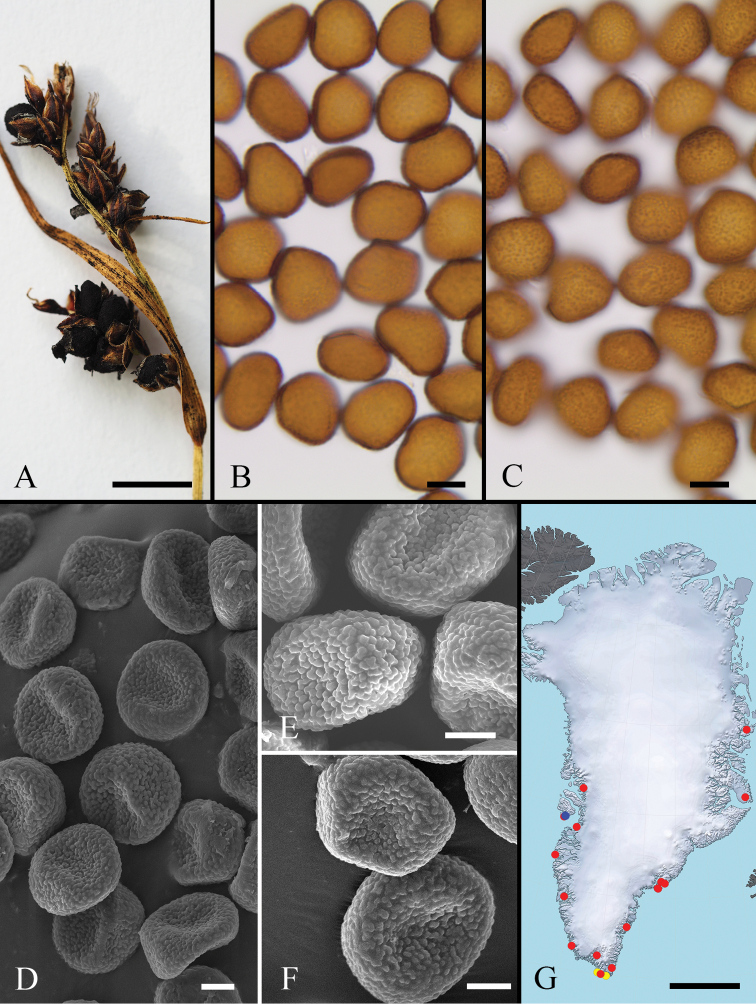
Anthracoidea bigelowii on Carex bigelowii. **A** habit (C-F-107497) **B, C** spores in LM, in median and surface view, respectively (C-F-108011) **D–F** spores in SEM (**D, E** 13 Aug 1928, J. Eugenius, s.n., C-Greenland herb., s.n.; **F** C-F-102524) **G** distribution map (red circles – on Carex bigelowii; yellow circles – on hybrids of C. bigelowii; blue circle – on C. concolor). Scale bars: 0.5 cm (**A**); 10 µm (**B, C**); 5 µm (**D–F**); 500 km (**G**).

**Hosts and distribution within the studied area** — On Cyperaceae: Carex sect. Phacocystis: Carex bigelowii – West and East Greenland; hybrids of C. bigelowii – West Greenland; C. concolor (C. aquatilis var. minor Boott, C. stans Drejer, C. aquatilis subsp. stans (Drejer) Hultén) – West Greenland (Fig. 3G).

**Specimens examined or recorded.**

On **Carex bigelowii** Torr. ex Schwein.:

**WG**, near Maamorilik, 71°06'N, 51°14'W, alt. 150 m, 7 Aug 1983, leg. J. Poelt & H. Wiese, s.n. (C-F-107990!, SOMF!, Vánky, Ustilaginales Exsiccata, no. 453; Vánky 1985b, 2011).

**WG**, Disko Island, Lyngmarksbugten near Qeqertarsuaq (Godhavn), 69°14'24"N, 53°32'24"W, 13 Aug 1967, leg. M. Lange, no. ML 561 (C-F-108011!).

**WG**, Qasigiannguit (as ‘Christianshåb’), 68°49'N, September 1835, leg. J. Vahl, s.n. (C-F-102531!, as ‘*U. caricis* on Carex rigida’; Rostrup 1888, as ‘*U. caricis* on Carex rigida’; rev. J.A. Nannfeldt, as ‘*Ci. bigelowii*’).

**WG**, Sisimiut, Nasaasaaq (as ‘Kællingehætten’), 66°55'48"N, 53°38'24"W, 16 Aug 2016, leg. H. Knudsen, nos HK 16.169, HK 16.170, HK 16.171 (C-F-108406!, C-F-108407!, C-F-108408!).

**WG**, Godthåbsfjord (as ‘Baals Revier’), 64°08–45'N, 1828–1836, leg. J. Vahl, s.n. (n.v.; not found in C; Rostrup 1888, as ‘*U. caricis* on Carex rigida’).

**WG**, near Neria, 61°33'N, 13 Aug 1928, leg. J. Eugenius, s.n. (C-Greenland herb.!, s.n., the host as ‘Carex goodenowii J. Gay’); **ditto**, sine dat., leg. J. Eugenius, s.n. (C-Greenland herb.!, s.n., the host as ‘C. goodenowii’).

**WG**, Narsarsuaq, Hospitalsdalen, 61°10'N, 45°26'W, alt. 40 m, 9 Aug 1985, leg. T. Borgen, no. TB 85.194 (C-F-108012!).

**WG**, Frederiksdal, 60°N, July 1829, leg. J. Vahl, s.n. (C-F-102532!, 102533!, as ‘*U. caricis* on Carex rigida’; Rostrup 1888, as ‘*U. caricis* on C. rigida’; rev. J.A. Nannfeldt, as ‘*Ci. bigelowii*’).

**WG**, Torsukatak, ca 60°N, July 1829, leg. J. Vahl, s.n. (C-F-102528!, as ‘*U. caricis* on Carex rigida’; Rostrup 1888, as ‘*U. caricis* on C. rigida’; rev. J.A. Nannfeldt, as ‘*Ci. bigelowii*’).

**EG**, Zackenberg, Ulvehøj, 74°28'N, 20°34'W, 7 Aug 1999, leg. T. Borgen, no. TB 99.240 (C-F-106650!).

**EG**, Nerlerit Inaat/Constable Pynt, half way up the Gåseelv valley, N-side, 70°46'N, 22°45'W, 8 Aug 2017, leg. H. Knudsen, no. HK 17.171B (C-F-105073!); **ditto**, Gåseelv, 70°46'N, 12 Aug 2017, leg. S.A. Elborne, no. SAE-2017.233GR (C-F-107497!); **ditto**, Gåseelv, 70°46'N, 12 Aug 2017, leg. H. Knudsen, s.n. (C-F-108008!).

**EG**, Qingorssuaq, ca 66°01–07'N, 37°09–16'W, 15 Aug 1932, leg. N. Tinbergen, no. 34 (U 1227699!, as ‘Ci. caricis on Carex rigida’).

**EG**, Tasiilaq Island, Amagâ Tasiusak, 65°38'N, 37°37'W, 21 Aug 1902, leg. C. Krusse, Expeditio Danica in Groenlandiam orientalem 1901–1902, s.n. (C-F-102527!, as ‘*U. caricis* on Carex rigida’; Rostrup 1904, as ‘*U. caricis* on C. rigida’; rev. J.A. Nannfeldt, as ‘*Ci. bigelowii*’).

**EG**, Tasiilaq Island, Tasiusak, 65°37'N, 37°33'W, 22 Jul 1902, leg. C. Krusse, Expeditio Danica in Groenlandiam orientalem 1901–1902, s.n. (C-F-102526!, as ‘*U. caricis*’; Rostrup 1904, as ‘*U. caricis* on Carex rigida’; rev. J.A. Nannfeldt as ‘*Ci. bigelowii*’); **ditto**, 65°37'N, 6 Sep 1932, leg. T. Bøcher, The Scoresby Sound Committee’s 2^nd^ East Greenland Expedition in 1932 to King Christian IX’s Land, no. 600 (C-F-102525!, as ‘Ci. caricis on Carex rigida’; rev. J.A. Nannfeldt, as ‘*Ci. bigelowii*’).

**EG**, Ikatek, 65°56'N, 36°34'W, 1898–1899, leg. C. Krusse, Expeditio Danica in Groenlandiam orientalem 1898–1899, s.n. (C-F-102524!, as ‘*U. caricis* on Carex sp.’; Rostrup 1904, as ‘*U. caricis* on Carex sp.’; rev. J.A. Nannfeldt, as ‘*Ci. bigelowii*’).

**EG**, NW of Griffenfeldt Island (as ‘Umanak’), N of Sehesteds Fjord, Claradalen, ca 63°08'N, 12 Sep 1932, leg. J. Devold, s.n. (O!, s.n., as ‘Ci. caricis on Carex rigida’; Hagen 1947).

**EG**, Lindenow Fjord, Møretun, 60°28'N, 43°18'W, 31 Jul 1932, leg. J. Devold & P.F. Scholander, s.n. (n.v.; not found in O; Hagen 1947, as ‘Ci. caricis on Carex rigida’); **ditto**, 31 Jul 1932, leg. J. Devold & P.F. Scholander, s.n. (O!, s.n., as ‘Ci. caricis on C. rigida ad *haematolepidem* Drejer’; Hagen 1947, as ‘Ci. caricis on C. rigida’; rev. J.A. Nannfeldt, as ‘*Ci. bigelowii*’).

On **hybrids** of **Carex bigelowii**:

**WG**, Tasermiut, 60°05'N, sine dat., leg. J. Vahl, s.n. (C-F-102529!, 102534!, as ‘*U. caricis* on Carex hyperborea’; Rostrup 1888, as ‘*U. caricis* on *C. hyperborea*’; rev. J.A. Nannfeldt, as ‘*Ci. bigelowii* on C. bigelowii × ?’).

**WG**, Ilua, 59°55'N, 1889, leg. E. Lundholm, s.n. (C-F-102530!, as ‘*U. caricis* on Carex hyperborea’; Rostrup 1891, as ‘*U. caricis* on *C. hyperborea*’; rev. J.A. Nannfeldt, as ‘*Ci. bigelowii* on C. bigelowii × ?’).

On **Carex concolor** R. Br.:

**WG**, Disko Island, Qeqertarsuaq (as ‘Godhavn’), 69°14'50"N, September 1931, leg. F. Johansen, s.n. (O!, s.n.; Hagen 1947, as ‘Ci. caricis’ on ‘Carex stans’; rev. J.A. Nannfeldt, as ‘*Ci. bigelowii*’); **ditto**, NE of Qeqertarsuaq, ca 69°14'50"N, 53°32'W, alt. 50 m, 29 Jul 1982, leg. J. Poelt & H. Ullrich, s.n. (GZU 000323434, the host as ‘C. stans’).

**Known hosts** — On Cyperaceae: Carex sect. Phacocystis Dumort.: Carex bigelowii subsp. bigelowii, C. bigelowii subsp. ensifolia (Turcz. ex Gorodkov) Holub (incl. C. bigelowii subsp. arctisibirica (Jurtzev) Á. Löve & D. Löve), C. bigelowii subsp. rigida W. Schultze-Motel, hybrids of C. bigelowii, C. concolor, C. elata All., C. scopulorum Holm, C. scopulorum var. bracteosa (L.H. Bailey) F.J. Herm. (C. gymnoclada Holm).

**General distribution. North** (Iceland, Faeroes, UK, Norway, Sweden, Finland, Arctic Russia) and **Central Europe. Asia**: Arctic Russia, West Siberia, Russian Far East. **North America**: Canada, Greenland, U.S.A.

**Earlier reports from Greenland**: Rostrup (1888, 1891, 1904, as ‘*U. caricis*’), Clinton (1904, 1906, as ‘Ci. caricis’), Hagen (1947, as ‘Ci. caricis’), Fischer (1953, as ‘Ci. caricis’), Nannfeldt and Lindeberg (1965), Nannfeldt (1979), Vánky (1985b).

**Comments** — Anthracoidea bigelowii is one of the most widespread species of this genus in Greenland. In this study, many infected specimens of Carex bigelowii from Greenland were seen but only some of them were unambiguously assigned to A. bigelowii due to the following main problems: (i) the taxonomy of the C. bigelowii complex is not sufficiently solved, (ii) the sori in some collections are too young, and (iii) the number of Anthracoidea species on sedges in the C. bigelowii complex seems to be higher than currently known.

The Carex bigelowii complex includes a group of taxa distributed throughout the Arctic and in the mountains of the Temperate zone (Hultén and Fries 1986: 474; Holub 1968). Chater (1980: 320) considered Carex bigelowii as ‘an extremely variable species whose infraspecific taxonomy is confused ... by the occurrence of numerous hybrids and by the fact that published work on it has never taken full account of the variation over the whole range’. Brief overviews of the studies of this complex of sedges and lists with published synonyms are given in Brooker et al. (2001) and Schönswetter et al. (2008). In *Flora Europaea* (Chater op. c.), a single species, C. bigelowii, with four subspecies were accepted: (i) subsp. bigelowii in North Europe (also in Greenland and northeastern North America); (ii) subsp. rigida W. Schultze-Motel in Central and northwestern Europe, and western Fennoscandia; (iii) subsp. arctisibirica (Jurtzev) Á. Löve & D. Löve in northern Russia (and Siberia); and (iv) subsp. ensifolia (Turcz. ex Gorodkov) Holub in the southern Ural (and Siberia).

In the *Flora of North America* treatment (Standley et al. 2002), two subspecies were distinguished within C. bigelowii in North America: subsp. bigelowii, distributed in Central Canada, North American Atlantic Region, Greenland, and North Europe, and subsp. lugens (Holm) T.V. Egorova with an amphi-Beringian distribution.

For the area under current discussion, Böcher et al. (1978) accepted C. bigelowii with two subspecies: subsp. bigelowii (incl. C. hyperborea Drejer), distributed in southern and western Greenland, and subsp. nardeticola Holub (corresponding to subsp. rigida sensu Chater 1980) in eastern Greenland.

Based on results from molecular investigations of Schönswetter et al. (2008), three subspecies are recognized within C. bigelowii in *Panarctic Flora* (Elven et al. 2018): (i) subsp. bigelowii – a North American–amphi-Atlantic taxon (distributed in northeastern North America, Greenland, Iceland, and northern Scandinavia); (ii) subsp. rigida – a European (central and northwestern)–amphi-Atlantic taxon (distributed in mountains of Central Europe, and in Norway, British Isles, Iceland, and Greenland); and (iii) subsp. ensifolia – a European (northeastern)–Asian (northern)–amphi-Beringian taxon (distributed in Svalbard, northeastern Europe, northern Asia, and northwestern North America). This complex of species is in need of additional molecular and morphological studies.

Considered as coextensive with its principal host (Nannfeldt and Lindeberg 1965; Nannfeldt 1979), Anthracoidea bigelowii is known on Carex bigelowii s. lat. from Iceland, the Faeroes, Scotland, Fennoscandia, Arctic Russia, West Siberia, Russian Far East, Canada (Nunavut, Quebec, and Labrador – Nannfeldt and Lindeberg 1965; Parmelee 1983), and Greenland.

In accordance with the practice of the time (cfr Holub 1968), the illegitimate name Carex rigida Gooden. 1794 (non C. rigida Schrank 1789, q.e. C. ferruginea) was used for some of the infected specimens of C. bigelowii, published for Greenland in the last century.

Ødum (1958), in an article about the flora of the islands Alanngorsuaq and Nunarsuit, published a brief note for occurrence of Carex stylosa var. nigritella (Drejer) Fernald infected with ‘Cintractia caricis’. The following specimens of Ødum are kept in the Greenland Herbarium (C): (i) Alanngorsuaq Island, ca 60°50'N, 4 Aug 1957, leg. S. Ødum, no. 131b (C-Greenland herb.!, s.n., host plant initially identified as ‘C. stylosa var. nigritella’ but later revised by J. Feilberg as ‘C. bigelowii’), and (ii) Nunarsuit Island, ca 60°42'N, 48°05'W, Jun–Sep 1957, leg. S. Ødum (C-Greenland herb.!, s.n.). Nannfeldt (1979: 37) referred these specimens to as ‘Anthracoidea sp. 10’ on Carex stylosa C.A. Mey., ‘found smutted only in two localities in S.W. Greenland (Ødum 1958: 391) and one in Alaska (Jørstad 1962: 15)’. The Greenlandic specimens must be removed from this list since their host is Carex bigelowii but not C. stylosa.

The second host plant listed here for Greenland, Carex concolor, is a circumpolar–alpine species (distributed in northeastern Europe, Russian Arctic, Siberia, Russian Far East, Alaska, Canada, and Greenland) (Hultén and Fries 1986: 473; Egorova 1999; Saarela et al. 2017; Elven et al. 2018). This sedge has been variously treated, at a different rank (as ‘C. aquatilis var. minor’, e.g., Standley et al. 2002; Saarela et al. 2013; or as ‘C. aquatilis subsp. stans’, e.g., Chater 1980; Hultén and Fries 1986; Egorova 1999; Aiken et al. 2007). When considered at the rank of species, the name C. concolor R. Br. 1823 has priority before C. stans Drejer 1841.

Carex concolor was included by Vánky (2011) among the hosts of Anthracoidea heterospora and A. liroi, but was omitted among the hosts of A. bigelowii, despite the presence of earlier reports from East Siberia (Lena-Kolyma region) and the Russian Far East (Okhotsk region) (Govorova 1990; Azbukina et al. 1995). These specimens are not revised by us, but the data about their morphology, given in Azbukina et al. (1995: 20), matches the description of A. bigelowii. This fungus-host combination is recorded here for the first time from Greenland.

Considering its distribution on all hosts, A. bigelowii is a circumboreal–polar species.

#### 3(3) Anthracoidea capillaris Kukkonen, Ann. Bot. Soc. Zool.-Bot. Fenn. ‘Vanamo’ 34(3): 50, 1963. — Holotype on Carex capillaris, Finland, Lapponia enontekiensis, Kilpisjärvi, Mt. Saana, 27 Jul 1955, leg. Y. Mäkinen (TUR).

Fig. 4A–F

**Infection** local. **Sori** in some female flowers, around aborted nuts as subglobose, ovoid or broadly ellipsoidal hard bodies, 0.3–0.6 mm long, initially covered by a thin, grayish peridium that later flakes away exposing a black spore mass, powdery on the surface. **Spores** small-sized, flattened, in plane view irregularly rounded, angular, suborbicular, broadly elliptical or ovate in outline, occasionally with a protuberance, in plane view (14.5–)15.5–21.5(–22) × (13–)14–17.5(–18.5) (17.6 ± 1.2 × 15.8 ± 1.1) µm (n/_3_ = 300), in side view 9.5–12.5 μm thick, medium or dark reddish brown; wall unevenly thickened, 1.0–2.4(–2.8) μm thick, thickest at the angles and protuberances, often with 1–4(–5), usually well visible internal swellings, sometimes light refractive areas present; surface minutely verruculose to almost smooth, spore profile not affected. In SEM warts up to 0.15 μm high, usually solitary, sometimes partly confluent, forming short rows or small groups, sometimes rugulose in the middle part of the flattened sides. **Spore germination** of Anthracoidea-type (after Kukkonen 1963), resulting in a two-celled basidium, 120–170 μm long, the apical cell 40–70 μm long and 3.5–4.5 μm thick; producing 2–3 basidiospores on each cell; basidiospores ellipsoidal, ovoid or obovoid, (5–)6–12(–14) × 2.5–6 μm.

**Figure 4.**
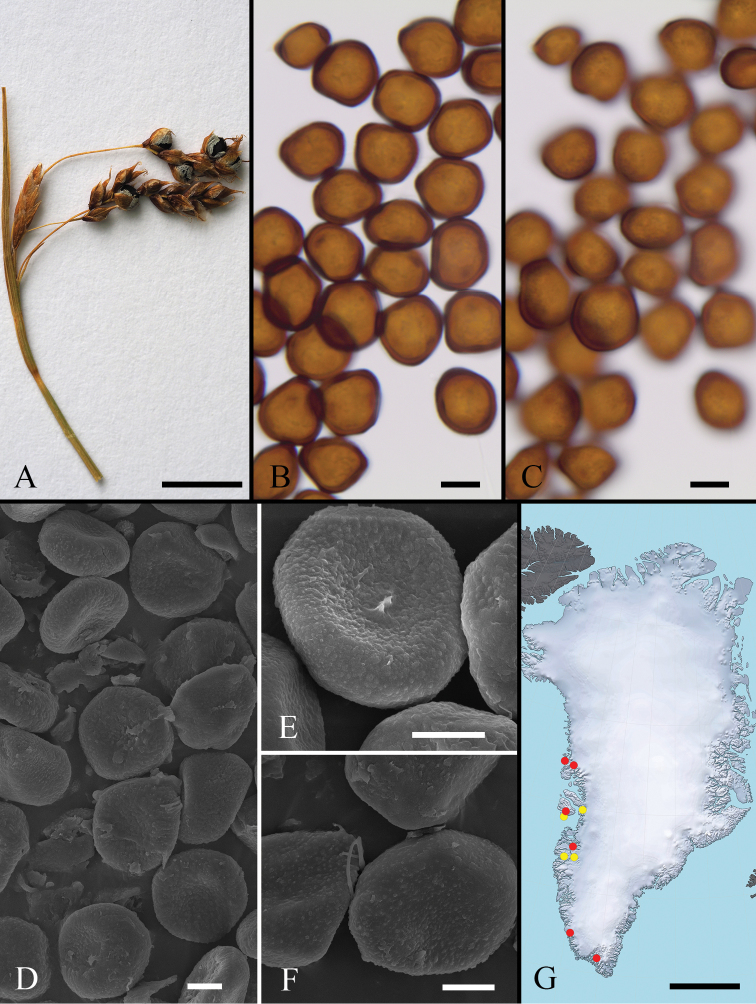
Anthracoidea capillaris on Carex capillaris (**A–C, E, F**) and C. boecheriana (**D**). **A** Habit (S. Frederiksen & L.B. Jørgensen, no. 68-1699, C-Greenland herb., s.n.) **B, C** spores in LM, in median and surface view, respectively (V. Dalgaard, no. 86-396, C-Greenland herb., s.n.) **D–F** spores in SEM (**D** 20 Jul 1926, leg. M.P. Porsild, s.n., C-Greenland herb., s.n.; **E** S. Frederiksen & L.B. Jørgensen, no. 68-1699, C-Greenland herb., s.n.; **F** 19 Jul 1939, leg. M.P. Porsild, s.n., C-Greenland herb., s.n.) **G** distribution map (red circles – on Carex capillaris; yellow circles – on C. boecheriana). Scale bars: 0.5 cm (**A**); 10 µm (**B, C**); 5 µm (**D–F**); 500 km (**G**).

**Hosts and distribution within the studied area** — On Cyperaceae: Carex sect. Chlorostachyae Meinsh.: Carex boecheriana – West Greenland; C. capillaris – West Greenland (Fig. 4G).

**Specimens examined or recorded.**

On **Carex boecheriana** Á. Löve, D. Löve & Raymond:

**WG**, Alluttoq Island (Arve-Prinsens Ejland), Arsivik, 69°51–52'N, 50°42–43'W, alt. 40 m, 8 Aug 1981, leg. C. Bay et al., no. 1918 (C-Greenland herb.!, s.n.; the host plant confirmed by P.W. Ball for *Flora of North America*).

**WG**, Disko Island, near Arctic Station, 69°15'N, 20 Jul 1926, leg. M.P. Porsild, s.n. (C-Greenland herb.!, s.n.; the host plant as ‘C. capillaris var. robustior Lange’, rev. P.W. Ball for *Flora of North America*).

**WG**, Søndre Strømfjord, N of the airfield, ca 67°00'N, 50°42'W, alt. 20 m, 12 Aug 1983, leg. J. Poelt, s.n. (GZU!, s.n.).

**WG**, Ikertôq, head of Akugdleq, at the river Eqatdlivia, 66°56'N, 52°15'W, alt. 10 m, 1 Aug 1978, leg. C. Bay & S. Hanfgarn, Pl. Vascul. Groenl. Exsicc., no. 632 (C-Greenland herb.!, s.n.; the host plant confirmed by P.W. Ball for *Flora of North America*).

On **Carex capillaris** L.:

**WG**, Prøven Island, 72°23'N, 55°33'W, 4 Sep 1934, leg. M.P. Porsild, s.n. (C-Greenland herb.!, s.n.).

**WG**, Ulvkusigssat Fjord, Iviangernat, 72°15'N, 53°46'W, 17 Aug 1950, leg. K. Jakobsen, no. 5796 (C-Greenland herb.!, s.n.).

**WG**, Disko Island, Disko Fjord, Orpît, 69°35'N, 53°26'W, alt. ca 100 m, 23 Jul 1986, leg. V. Dalgaard, no. 86-396 (C-Greenland herb.!, s.n.; the host plant confirmed by P.W. Ball for *Flora of North America*).

**WG**, Disko Island, Arctic Station, 69°15'N, 19 Jul 1939, leg. M.P. Porsild, s.n. (C-Greenland herb.!, s.n.).

**WG**, Nordre Strømfjord, Sanerut, 67°37'N, 51°11'W, alt. 250 m, 8 Jul 1988, leg. B. Fredskild & V. Dalgaard, no. 88-361 (C-Greenland herb.!, s.n.; the host plant as ‘C. cfr. boecheriana’, rev. P.W. Ball for *Flora of North America*).

**WG**, Nigerdlîp Qôrorssua, 62°21'N, 49°25'W, alt. 330 m, 25 Jul 1968, leg. S. Frederiksen & L.B. Jørgensen, no. 68-1699 (C-Greenland herb.!, s.n.; the host plant as ‘*C. glacialis*’, rev. B. Fredskild).

**WG**, Igaliko, 60°59'N, 45°25'W, 1 August, leg. ?, s.n. (C-Greenland herb.!, s.n.).

**Known hosts** — On Cyperaceae: Carex sect. Chlorostachyae: Carex boecheriana, C. capillaris, C. ledebouriana C.A. Mey. ex Trevir., C. tenuiformis H. Lév. & Vaniot.

**General distribution. Europe**: Iceland, UK, Norway, Sweden, Finland, Germany, Switzerland, Austria, Slovenia, Russia, Ukraine. **Asia**: Russian Far East, Japan. **North America**: Canada, Greenland.

**Comments** — In this study, Carex capillaris (the type host plant of Anthracoidea capillaris) is considered in its broad sense. In its broad circumscription, this sedge is a circumboreal–polar species, distributed in the Arctic but characteristic also for the boreal zone and mountains of Central and South Europe and North America (Hultén and Fries 1986: 512; Egorova 1999; Elven et al. 2018). Carex capillaris has been variously interpreted in the botanical literature. In the *Flora of North America* treatment (Ball 2002c), infraspecific taxa were not recognized within this species. In some recent treatments, however, C. capillaris is considered as a highly polymorphic species at a circumpolar scale (Saarela et al. 2013; Alsos et al. 2018; Elven et al. 2018), and accepted as a core species in a species aggregate, including C. capillaris — with two subspecies: subsp. capillaris (var. capillaris and var. elongata Olney ex Fernald) and subsp. fuscidula (V.I. Krecz. ex T.V. Egorova) Á. Löve & D. Löve), and C. krausei Boeckeler (Elven et al. 2018). A third species, C. boecheriana (known from Greenland and Ellesmere Island), is also referred to this species aggregate, but some authors (e.g. Elven et al. 2018) consider it synonymous with C. krausei. Whether the morphological differences are sufficient to warrant its recognition as a distinct species is yet to be satisfactorily resolved. All five taxa within the C. capillaris aggr. are listed as occurring in Greenland, because of which we prefer to consider the host plant of the infected Greenlandic specimens in its broad sense. The case of C. boecheriana is an exception, as the identification of the cited specimens is confirmed by P.W. Ball.

Anthracoidea capillaris on Carex capillaris s. lat. has been previously reported from Canada, North Europe, the Alps, the Carpathians, and Kurile Islands (Nannfeldt 1979; Denchev and Minter 2010; Denchev et al. 2013). In Canada, it is known from Yukon, Northwest Territories, Nunavut, British Columbia, Manitoba, Quebec, and Newfoundland and Labrador (Kukkonen 1963; Parmelee 1983, 1988).

Anthracoidea capillaris is recorded here for the first time from Greenland, on two host plants: Carex boecheriana and C. capillaris. The Greenlandic specimens show the typical features of A. capillaris (cfr Nannfeldt 1979; Vánky 2011; Denchev et al. 2013).

The spores of an examined specimen of A. capillaris on Carex boecheriana (Disko Island, near Arctic Station, C-Greenland herb.) measured (14.5–)15.5–21.5(–22) × (13–)14–17.5(–18.5) (17.6 ± 1.1 × 15.9 ± 1.0) μm. Carex boecheriana is recorded here for the first time as a host of Anthracoidea capillaris.

Anthracoidea capillaris is a circumboreal–polar species.

#### 4(4) Anthracoidea caricis (Pers. : Pers.) Bref., s. lat., Unters. Gesammtgeb. Mykol., 12, Brandpilze 3: 144, 1895. ≡ Uredo caricis Pers. : Pers., Syn. Meth. Fung. 1: 225, 1801. ≡ Caeoma caricis (Pers. : Pers.) Link, in Willdenow, Linné’s Species Plantarum, 4th edn, 6(2): 5, 1825. ≡ Ustilago caricis (Pers. : Pers.) Unger, Ueber den Einfluss des Bodens: 211, 1836. ≡ Cintractia caricis (Pers. : Pers.) Magnus, Verh. Bot. Vereins Prov. Brandenburg 37[1895]: 78, 1896. — Neotype on Carex pilulifera, Germany, Brandenburg, Kreis Zauch-Belzig, between Beelitz and Schlunkendorf, 10 Jun 1935, leg. E. Fahrendorff (TUR!) (design. by Kukkonen 1963: 58); isoneotypes in Sydow, Mycoth. German., no. 2880 (as ‘Cintractia caricis’).

Fig. 5A–F

**Infection** local. **Sori** in some female flowers, around aborted nuts as globose, subglobose or ovoid hard bodies, 0.5–0.8 mm long, initially covered by a thin, grayish peridium that later flakes away exposing a black spore mass, powdery on the surface. **Spores** medium-sized, flattened, in plane view irregularly rounded, angular, suborbicular, ovate, broadly elliptical, elliptical or elongate in outline, occasionally with a protuberance, in plane view (16–)17–24(–26) × (13.5–)14.5–19(–20) (19.8 ± 2.0 × 16.4 ± 1.2) µm (n/_1_ = 100), in side view 11.5–14.5 µm thick, medium or dark reddish brown; wall unevenly thickened, 1.0–2.6(–3.2) µm thick, thickest at the angles and protuberances, often with 1–3 internal swellings, light refractive areas present; minutely verruculose, spore profile not affected. In SEM warts up to 0.2 µm high, sometimes partly confluent, forming short rows or small groups. **Spore germination** of Anthracoidea-type (after Kukkonen 1963), resulting in a two-celled basidium, 100–150 μm long, the apical cell 20–45 μm long and 3.5–5.5 μm thick; producing 2–3 basidiospores on each cell; basidiospores ellipsoidal or ovoid to obovoid, 6–14 × 3.5–5.5 μm.

**Figure 5.**
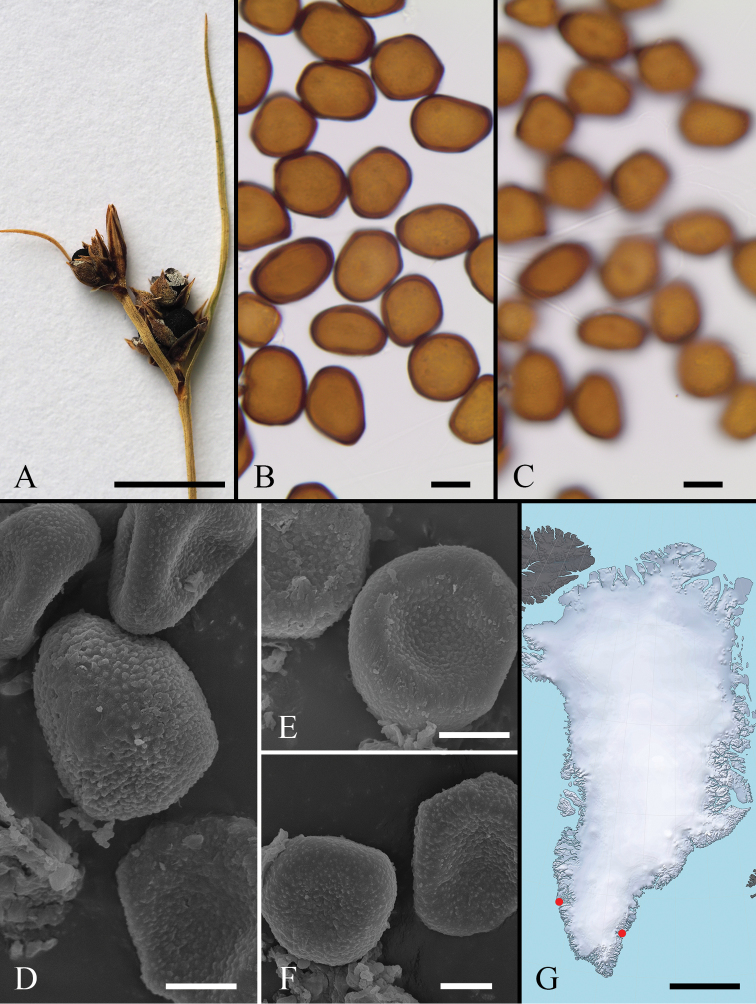
Anthracoidea caricis on Carex deflexa. **A** Habit (leg. P.F. Scholander, s.n., O, s.n.) **B, C** spores in LM, in median and surface view, respectively (leg. P.F. Scholander, s.n., O, s.n.) **D–F** spores in SEM (T.W. Böcher, no. 81, C-Greenland herb., s.n.) **G** distribution map. Scale bars: 0.5 cm (**A**); 10 µm (**B, C**); 5 µm (**D–F**); 500 km (**G**).

**Hosts and distribution within the studied area** — On Cyperaceae: Carex sect. Acrocystis Dumort.: Carex deflexa – West and East Greenland (Fig. 5G).

**Specimens examined or recorded.**

On **Carex deflexa** Hornem. var. **deflexa**:

**WG**, Godthåbsfjord, ca 64°03–08'N, 25 Jul 1976, leg. T.W. Böcher, no. 81 (C-Greenland herb.!, s.n.).

**EG**, Tingmiarmit Island, Brattneset, ca 62°43'N, 42°20'W, 8 Aug 1932, leg. P.F. Scholander, s.n. (O!, s.n., as ‘Ci. caricis’; Hagen 1947, as ‘Ci. caricis’).

**Known hosts** — On Cyperaceae: Carex sect. Acrocystis: Carex amgunensis F. Schmidt, C. deflexa var. deflexa, C. deflexavar. boottii L.H. Bailey (C. brevipes W. Boott in S. Watson, nom. inval.), C. inops subsp. heliophila (Mack.) Crins, C. montana L., C. oxyandra (Franch. & Sav.) Kudô, C. peckii Howe, C. pensylvanica Lam., C. pilulifera L., C. rossii Boott, C. umbellata Schkuhr ex Willd. (C. abdita E.P. Bicknell), [(?) Carex vanheurckii Müll. Arg.]; Carex sect. Hallerianae: Carex halleriana Asso.

**General distribution. Europe**: Iceland, UK, Norway, Sweden, Finland, Denmark, Estonia, Lithuania, France, Belgium, Germany, Poland, Switzerland, Austria, Czech Republic, Slovakia, Hungary, Romania, Ukraine, Andorra, Spain, Italy, Slovenia, Bosnia & Herzegovina, Bulgaria, Russia. **Asia**: Russian Far East, Japan, Iran, Mongolia. **North America**: Canada, Greenland, U.S.A.

**Earlier reports from Greenland**: Hagen (1947, as ‘Ci. caricis’), Fischer (1953, as ‘Ci. caricis’), Kukkonen (1963), Nannfeldt (1979).

**Comments** — Anthracoidea caricis is the type species of its genus. Initially, this species (as ‘Ci. caricis’) was generally considered to have a wide host range, including many species of Carex (comp. Fischer 1953; Zundel 1953). Later, it was recognized as a collective species (including Ci. irregularis Liro) that was parasitic on sedges in two sections, Acrocystis and Digitatae (Kukkonen 1963). Cintractia irregularis was recognized by Boidol and Poelt (1963) as a distinct species and transferred to Anthracoidea. Nannfeldt (1979) reduced the host range of A. caricis to sedges of section Acrocystis. Vánky (1994) and Guo (2000) proposed Carex halleriana (section Hallerianae) and some species of C. sect. Digitatae to be added to the list of hosts of Anthracoidea caricis. The current status of Anthracoidea on these sedges, as well as on C. vanheurckii, was discussed by Denchev et al. (2013) in the comments given to A. caricis and A. caryophylleae, respectively. The smut fungi, currently referred to as ‘A. caricis’, clearly form a species complex that requires further study. In the present treatment, A. caricis is considered in a broad sense.

Carex deflexa is a North American species. The Greenlandic plants belong to var. deflexa, distributed in Canada, northeastern U.S.A., and Greenland (Crins and Rettig 2002). The Cordilleran var. boottii (from mountain regions of the western North America) is also reported as a host of A. caricis (Kukkonen 1963).

In Canada, A. caricis is known on Carex deflexa, C. inops subsp. heliophila, C. peckii, C. pensylvanica, C. rossii, and C. umbellata from Northwest Territories, British Columbia, Alberta, Saskatchewan, Manitoba, Ontario, Quebec, and Nova Scotia (Savile 1952; Kukkonen 1963; Nannfeldt 1979; Parmelee 1983). For Greenland, A. caricis has been known only from a single collection on Carex deflexa, made in 1932 (Hagen 1947). A second Greenlandic locality is reported herein.

In its strict sense, Anthracoidea caricis is a Eurasiatic species with a disjunct distribution, mainly in the territory of temperate Eurasia. It is found in Europe, Iran, Mongolia, and East Asia (Denchev et al. 2013). The circumscription of this species is not satisfactorily resolved. In the present treatment, focused only on the smut fungi of Greenland, A. caricis is considered in a broad sense, as a circumboreal species. The taxonomic status of A. caricis complex will be discussed elsewhere.

#### 5(5) Anthracoidea elynae (Syd.) Kukkonen, Ann. Bot. Soc. Zool.-Bot. Fenn. ‘Vanamo’ 34(3): 65, 1963. ≡ Cintractia elynae Syd., Ann. Mycol. 22: 289, 1924. ≡ Cintractia carpophila var. elynae (Syd.) Savile, Canad. J. Bot. 30: 419, 1952. — Lectotype on Kobresia myosuroides, Switzerland, ‘Horald bei Zermatt’, 29 Aug 1886, leg. P. Magnus (S) (design. by Kukkonen 1961: 157).

Fig. 6A–F

**Infection** local. **Sori** in some female flowers, around aborted nuts as subglobose, ovoid or broadly ellipsoidal hard bodies, 0.5–1 mm long, initially covered by a thin, grayish peridium that later flakes away exposing a black spore mass, powdery on the surface. **Spores** small- to medium-sized, flattened, in plane view suborbicular, broadly elliptical, ovate or slightly irregularly rounded in outline, in plane view (16–)17–21(–22) × (14–)15–19(–20) (18.8 ± 0.9 × 17.0 ± 0.9) µm (n/_3_ = 300), in side view 8.5–13.5 µm thick, often with a more or less conspicuous hyaline sheath on the flattened sides, medium or dark reddish brown; wall unevenly thickened, 1.0–2.0(–2.4) µm thick, often with 1–3 internal swellings, light refractive areas and protuberances absent; smooth to minutely verruculose, spore profile not affected. In SEM spore wall smooth, often minutely verruculose to rugulose in the middle part of the flattened sides. **Spore germination** of Anthracoidea-type (after Kukkonen 1961, 1963), resulting in a two-celled basidium, 70–100 μm long, the apical cell 25–40 μm long and 4–5 μm thick; initially producing only one basidiospore on each cell, later a second basidiospore being formed from a secondary branch of the primary sterigma; basidiospores ellipsoidal, ovoid or obovoid, 6–18 × 4–9 µm.

**Figure 6.**
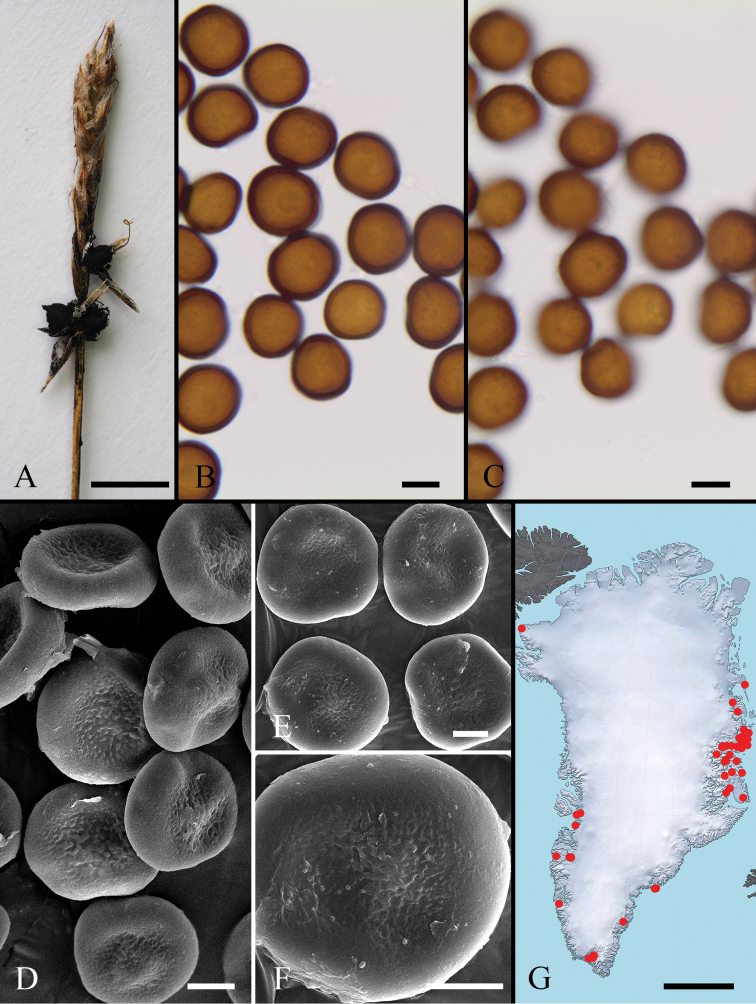
Anthracoidea elynae on Carex myosuroides. **A** Habit (C-F-111309) **B, C** spores in LM, in median and surface view, respectively (C-F-111309) **D–F** spores in SEM (**D**GZU 000323433; **E, F** 11 Aug 1994, leg. R. David & S. David, s.n., C-Greenland herb., s.n.) **G** distribution map. Scale bars: 0.5 cm (**A**); 10 µm (**B, C**); 5 µm (**D–F**); 500 km (**G**).

**Hosts and distribution within the studied area** — On Cyperaceae: Carex (the Myosuroides clade): Carex myosuroides – North, West and East Greenland (Fig. 6G).

**Specimens examined or recorded.**

On **Carex myosuroides** Vill. (Elyna myosuroides (Vill.) Fritsch; E. spicata Schrad., E. bellardii (All.) K. Koch, Kobresia bellardii (All.) Degl., K. myosuroides (Vill.) Fiori & Paol., K. scirpina Willd.):

**NG**, Foulk Fjord, 78°18'N, in clivo arenoso ad Reindeer Point, 11–12 Aug 1899, leg. H.G. Simmons, s.n. (O!, s.n., as ‘Ci. caricis’; Hagen 1947, as ‘Ci. caricis’).

**WG**, Diskofjord, N of head of Qaungulik, 69°40'N, 50°28'W, alt. 175 m, 26 Jul 1981, leg. B. Fredskild et al., no. 287 (C-Greenland herb.!, s.n.).

**WG**, Alluttoq Island (Arve-Prinsens Ejland), 69°32'N, 51°04'W, alt. 0–100 m, 29 Aug 1961, leg. S. Lægaard, no. 692 (C-Greenland herb.!, s.n.).

**WG**, E of Laksebugt near Qasigiannguit (as ‘Christianshåb’), 68°55'N, 50°56'W, alt. 350 m, 11 Aug 1986, leg. B. Fredskild & V. Dalgaard, no. 86-534 (C-Greenland herb.!, s.n.).

**WG**, Kangerlussuaq, 66–67°N, alt. 150 m, 26 Jul 1946, leg. T.W. Böcher, The Botanical Expedition to West-Greenland 1946, no. 221 (C-Greenland herb.!, s.n.); **ditto**, Kangerlussuaq Harbour, ca 66°59'N, 50°58'W, alt. 20 m, 27 Jul 1958, leg. Beschel, no. 8201 (C-Greenland herb.!, s.n.); **ditto**, Kangerlussuaq, N of the airport, ca 67°00'N, 50°42'W, alt. 100 m, 11 Aug 1983, leg. J. Poelt, s.n. (GZU 000323433!); **ditto**, Kangerlussuaq, Hassells Fjeld, Kløftsøerne, 67°00'N, 50°42'W, 28 Aug 2018, leg. H. Knudsen, nos HK 18.398A & HK 18.398B (C-F-111308, C-F-111309); **ditto**, Kangerlussuaq, slopes SW of Lake Ferguson, 66°57'36"N, 50°41'24"W, 29 Aug 2018, leg. H. Knudsen, no. HK 18.408 (C-F-111310); **ditto**, Kangerlussuaq, Lake Ferguson, Tasersuatsiaq, 66°57'36"N, 50°41'24"W, 22 Aug 2016, leg. S.A. Elborne, no. SAE-2016.148-GR (C-F-107280).

**WG**, Sarfanguak, 66°53'N, 1886, leg. Th. Holm, s.n. (Rostrup 1888, as ‘*U. caricis*’).

**WG**, Ameralik Fjord, 64°03'N, August 1830, leg. J. Vahl, s.n. (C-F-102537!, as ‘*U. caricis*’; Rostrup 1888, as ‘*U. caricis*’).

**WG**, Narsarsuaq, 61°10'04"N, 45°24'18"W, 14 Aug 2015, leg. H. Knudsen, no. HK 15.045 (C-F-8198!); **ditto**, Hospitalsdalen, 61°10'N, 45°25'W, 10 Aug 2018, leg. H.F. Gøtzsche, no. HFG 2018, 005 (C-F-113175!).

**WG**, Tunulliarfik Fjord (as ‘Tunugdliarfik’), 60°59'N, September 1828, leg. J. Vahl, s.n. (C-F-102539!, as ‘*U. caricis*’; Rostrup 1888, as ‘*U. caricis*’).

**WG**, Dyrnæs, 60°56'N, 46°04'W, 1 Aug 1963, leg. K. Damsholt & K. Holmen, no. F.1856 (C-Greenland herb.!, s.n.); **ditto**, alt. 25 m, 4 Aug 1963, leg. C. Hansen & K. Jakobsen, Plantae vasculares groenlandicae exsiccatae, no. 205 (E!, s.n.).

**EG**, Germania Land, Termometerfjeldet (as ‘Termometerfjeld’) near Danmark Havn, 76°46.6'N, 18°38.5'W, 15 Aug 1907, leg. A. Lundager, “Danmark” Expeditionen 1906–1908, s.n. (C-F-102540!, as ‘Ci. caricis’; Lind 1910, as ‘Ci. caricis’; Kukkonen 1961, as ‘Ci. elynae’).

**EG**, Bessel Fjord, 75°59'N, 21°55'W, alt. 10–50 m, 15–19 Jun 1989, leg. D. Boertmann, s.n. (C-Greenland herb.!, s.n.).

**EG**, Bredefjord, the east part, 75°28'N, 21°12'W, alt. 150 m, 8 Aug 1989, leg. C. Bay, no. 89-810 (C-Greenland herb.!, s.n.).

**EG**, Zackenberg, Ulvehøj, 74°28'N, 20°34'W, 24 July 1999, leg. T. Borgen, no. TB 99.147 (C-F-106648!).

**EG**, Wollaston Forland, Herschellhus (as ‘Kap Herschel’), ca 74°14.6'N, 19°41.1'W, 29 Jul 1929, leg. J. Vaage, s.n. (O!, s.n., as ‘Ci. caricis on Cobresia scirpina’); **ditto**, 1 Aug 1933, leg. A. Hagen, the Norwegian Expedition to NE Greenland 1933, s.n. (O!, s.n., as ‘Ci. caricis’).

**EG**, Clavering Island, Kap Mary, 74°09.7'N, 20°11.7'W, 5 Aug 1933, leg. A. Hagen, the Norwegian Expedition to NE Greenland 1933, s.n. (O!, s.n., as ‘Ci. caricis’); **ditto**, Eskimonæs, 74°06'N, 21°20'W, 18 Aug 1931, leg. P. Gelting, no. 37 (C-Greenland herb.!, s.n.).

**EG**, Strindberg Land (as ‘Strindbergs halvøya’), ca 1 km E of the Danish Hut, 30 Jul 1933, leg. A. Hagen, the Norwegian Expedition to NE Greenland 1933, s.n. (O!, s.n., as ‘Ci. caricis’); **ditto**, N of mouth of Broget Dal, 73°44'N, 24°25'W, 30 Jul 1994, leg. R. David & S. David, s.n. (C-Greenland herb.!, s.n.); **ditto**, between Bellavista and Sortefjeld, 73°45–47'N, 25°10–15'W, 11 Aug 1994, leg. R. David & S. David, s.n. (C-Greenland herb.!, s.n.).

**EG**, Andrée Land, the north part, Moränedal, N-side, 73°42'N, 25°17'W, alt. 400 m, 17 Aug 1949, leg. F.H. Schwarzenbach, no. 3456 (C-Greenland herb.!, s.n.); **ditto**, Moränedal, S-side, 73°41'N, 25°12'W, alt. 380 m, 18 Aug 1949, leg. F.H. Schwarzenbach, no. 3507 (C-Greenland herb.!, s.n.); **ditto**, Renbugten (on the N-side of Isfjord, as ‘Reinbukta’), 73°20.0'N, 26°28.5'W, 14 Aug 1929, leg. J. Vaage, s.n. (O!, s.n., as ‘Ci. caricis’).

**EG**, Hudson Land, Hoelsbo (as ‘Hoelsbu’), on the N-side of Moskusoksefjord, ca 73°42.2'N, 23°26.3'W, 9 Aug 1932, leg. S. Aandstad, s.n. (O!, s.n., as ‘Ci. caricis’); **ditto**, Hoelsbo, near the houses, 29 Jul 1933, leg. A. Hagen, the Norwegian Expedition to NE Greenland 1933, s.n. (O!, s.n., as ‘Ci. caricis’).

**EG**, Hudson Land, Ankerpladsen, on the N-side of Moskusoksefjord, 73°36.1'N, 22°22.5'W, 6 Aug 1930, leg. P.F. Scholander, s.n. (O!, s.n., as ‘Ci. caricis’); **ditto**, 6 Aug 1930, leg. J. Vaage, s.n. (O!, s.n., as ‘Ci. caricis’).

**EG**, Loch Fyne (between Hudson Land and Hold with Hope), 73°39–45'N, 21°25–45'W, alt. 0–50 m, 1–4 Aug 1988, leg. R. David & S. David, s.n. (C-Greenland herb.!, s.n.).

**EG**, Hold with Hope, Kap Stosch, 74°03.6'N, 21°43.8'W, 24 Jul 1930, leg. P.F. Scholander, s.n. (O!, s.n., as ‘Ci. caricis’); **ditto**, Knorten, 73°42.4'N, 20°34.5'W, 18 Aug 1933, leg. A. Hagen, the Norwegian Expedition to NE Greenland 1933, s.n. (O!, s.n., as ‘Ci. caricis’); **ditto**, (?) Gryhelien [handwritten and hard to read], 16 Aug 1933, leg. A. Hagen, the Norwegian Expedition to NE Greenland 1933, s.n. (O!, s.n., as ‘Ci. caricis’).

**EG**, Hold with Hope, Myggbukta (on the N-side of Mackenzie Bugt), on the shore, at the houses, 73°29.4'N, 21°33.4'W, 3 Jul 1933, leg. A. Hagen, the Norwegian Expedition to NE Greenland 1933, s.n. (O!, s.n., as ‘Ci. caricis’); **ditto**, ca 1 km NW of the houses, 22 Jul 1933, leg. A. Hagen, the Norwegian Expedition to NE Greenland 1933, s.n. (O!, two specimens, s.n., as ‘Ci. caricis’); **ditto**, Myggbukta, 2 Aug 1929, leg. J. Vaage, s.n. (O!, s.n., as ‘Ci. caricis’).

**EG**, Vesle Finsch Island, ca 74°00'N, 18 Jul 1933, leg. A. Hagen, the Norwegian Expedition to NE Greenland 1933, s.n. (O!, s.n., as ‘Ci. caricis’).

**EG**, Gael Hamke Bugt, Jackson Island, ca 73°55'N, 11 Aug 1933, leg. A. Hagen, the Norwegian Expedition to NE Greenland 1933, s.n. (O!, s.n., as ‘Ci. caricis’).

**EG**, Holland Island, ca 73°36'N, 20°21'W, 13 Aug 1933, leg. A. Hagen, the Norwegian Expedition to NE Greenland 1933, s.n. (O!, s.n., as ‘Ci. caricis’).

**EG**, Ymer Island, Dusén Fjord, ca 73°14'N, 24°00'W, in the W-part, 7 Aug 1933, leg. A. Hagen, the Norwegian Expedition to NE Greenland 1933, s.n. (O!, two specimens, s.n., as ‘Ci. caricis’); **ditto**, in the E-part, 7 Aug 1933, leg. A. Hagen, the Norwegian Expedition to NE Greenland 1933, s.n. (O!, s.n., as ‘Ci. caricis’).

**EG**, Geographical Society Island, 5 km W of Husbukta (ca 72°49.7'N, 22°52.5'W), 16 Aug 1930, leg. J. Vaage, s.n. (O!, two specimens, s.n., as ‘Ci. caricis’); **ditto**, 15 km W of Husbukta, 17 Aug 1930, leg. P.F. Scholander, s.n. (O!, s.n., as ‘Ci. caricis’).

**EG**, Maria Island, Nattvika, 72°57.8'N, 24°50.9'W, 12 Aug 1930, leg. J. Vaage, s.n. (O!, s.n., as ‘Ci. caricis’).

**EG**, Ella Island, Cape Oswald, 72°53'N, 25°08'W, leg. Povelsen, s.n. (UPS, n.v.; Kukkonen 1963); **ditto**, Kap Elisabeth, 72°54.3'N, 24°48.5'W, 8 Aug 1930, leg. J. Vaage, s.n. (O!, s.n., as ‘Ci. caricis’); **ditto**, 72°50'N, 17 Aug (?) 1930, leg. ?, no. 1048 (C-Greenland herb.!, s.n.).

**EG**, Traill Island, Kap Simpson, 72°08.1'N, 22°11.6'W, 12 Aug 1929, leg. J. Vaage, s.n. (O!, s.n., as ‘Ci. caricis’).

**EG**, Stauning Alper, near Alpefjord, 28 Jul 1933, leg. A. Hagen, the Norwegian Expedition to NE Greenland 1933, s.n. (O!, s.n., as ‘Ci. caricis’); **ditto**, Skeldal, 72°15'N, 24°W, 22 Jul 1963, leg. D.R. Spearing et al., no. 352 (C-Greenland herb.!, s.n.).

**EG**, Kjoveland, E of the mouth of Nordvest Fjord, 71°22–25'N, 24°43–54'W, 8 Aug 1984, leg. S. Holt, 84-043 (C-Greenland herb.!, s.n.).

**EG**, Bjørne Islands (NE of Milne Land), 71°07'N, 13 Aug 1951, leg. H.B. Andersen, s.n. (C-Greenland herb.!, s.n.).

**EG**, Jameson Land, Constable Pynt, 70°44–46'N, 22°39–42'W, 22–30 Jul 1989, leg. H. Andersson, s.n. (C-Greenland herb.!, s.n.).

**EG**, Sarfakajik, 65°38'N, 37°25'W, 26 Aug 1902, leg. C. Krusse, Expeditio Danica in Groenlandiam orientalem 1901–1902, s.n. (C-F-102538!, as ‘*U. caricis*’; Rostrup 1904, as ‘*U. caricis*’).

**EG**, Angmagsalik Island, near Kong Oscar Havn, ca 65°37'N, 37°37'W, alt. ca 200 m, 14 Aug 1968, leg. F.J.A. Daniëls & J.G. de Molenaar, no. 151 (U 1297037!); **ditto**, at the bottom of Kong Oscar Havn, alt. ca 220 m, 15 Aug 1968, leg. F.J.A. Daniëls & J.G. de Molenaar, no. 19 (U 1297036!).

**EG**, Akorninarmiut, Skjoldungenområdet, Dronning Marias Dal, 63°28'N, 41°53'W, 12 Aug 1932, leg. J. Devold & P.F. Scholander, s.n. (O!, s.n., as ‘Ci. caricis’; Hagen 1947, as ‘Ci. caricis’).

**Known hosts** — On Cyperaceae: Carex myosuroides (principal host). Reported also on Carex alatauensis S.R. Zhang (Kobresia humilis (Trautv.) Serg.), C. borealipolaris S.R. Zhang (K. sibirica (Turcz. ex Ledeb.) Boeckeler, K. smirnovii N.A. Ivanova), C. capillifolia (Decne.) S.R. Zhang (K. capillifolia (Decne.) C.B. Clarke, K. macrolepis Meinsh.), C. deasyi (C.B. Clarke) O. Yano & S.R. Zhang (K. schoenoides (C.A. Mey.) Steud.), C. pseudolaxa (C.B. Clarke) O. Yano & S.R. Zhang (K. laxa Nees).

**General distribution. Europe**: Iceland, Norway, Sweden, France, Germany, Switzerland, Austria, Romania, Spain, Italy. **Asia**: Arctic Russia, West and East Siberia, Russian Far East, Russian Caucasus, Georgia, Kazakhstan, Kyrgyzstan, Mongolia, China, Pakistan, India. **North America**: Canada, Greenland, U.S.A.

**Earlier reports from Greenland**: Rostrup (1888, 1904, as ‘*U. caricis*’), Clinton (1904, 1906, as ‘Ci. caricis’), Lind (1910, as ‘Ci. caricis’), Hagen (1947, as ‘Ci. caricis’), Kukkonen (1961, as ‘Ci. elynae’, 1963), Savile and Parmelee (1964), Cabot et al. (1988).

**Comments** — The circumscription of the genus Carex was recently expanded to include all species of the genera *Cymophyllus, Kobresia, Schoenoxiphium*, and Uncinia (Global Carex Group 2015, 2016). The new taxonomic scheme of Carex will reflect on the taxonomy of the Anthracoidea species on hosts of the former genus Kobresia, but the consequences thereof will not be discussed here since the present study is focused only on the smut fungi of Greenland, where only two Anthracoidea species on ‘Kobresia’ are distributed and they can be morphologically distinguished.

Carex myosuroides is a circumpolar–alpine species (Hultén and Fries 1986: 423; Elven et al. 2018), distributed in Eurasia and North America. Anthracoidea elynae is a widespread species, probably coextensive with its principal host. In North America, it is a common species in the Canadian Arctic and Greenland. In Canada, it is recorded from Yukon, Northwest Territories, Nunavut, British Columbia, Alberta, Quebec, and Labrador (Linder 1947, as ‘Ci. caricis’; Savile 1952, as ‘Ci. carpophila var. elynae’; Kukkonen 1961, 1963; Savile and Parmelee 1964; Parmelee 1969, 1988; Gremmen and Parmelee 1972; Farr and Rossman 2019); in the U.S.A., it is known from Wyoming and Colorado (Kukkonen 1961, 1963).

Anthracoidea elynae is a circumpolar–alpine species. It is one of the most widespread Anthracoidea species in Greenland. It was reported by J. Vaage and A. Hagen as a widely distributed species throughout an area studied by them in East Greenland between 71°30'–75°40'N (‘Eirik Raudes Land’), where Carex myosuroides was found by Vaage (1932: 64, as ‘Cobresia scirpina’) to be ‘frequently attacked by fungi’ (most likely by Anthracoidea elynae that is the most visible parasitic fungus on this sedge), and was collected by Hagen (1947: 284), infected with ‘Cintractia caricis’, in 26 localities.

#### 6(6) Anthracoidea heterospora (B. Lindeb.) Kukkonen, Ann. Bot. Soc. Zool.-Bot. Fenn. ‘Vanamo’ 34(3): 63, 1963. ≡ Cintractia heterospora B. Lindeb., in Nannfeldt and Lindeberg, Svensk Bot. Tidskr. 51: 500, 1957. ≡ C. variabilis Lehtola, Acta Agralia Fenn. 42: 45, 1940 (later homonym), non C. variabilis S. Ito, Trans. Sapporo Nat. Hist. Soc. 14: 92, 1935 (q.e. Anthracoidea variabilis (S. Ito) Kakish.). — Lectotype on Carex nigra, Finland, Nyland, Tikkurila, 1915, leg. E. Kitunen (UPS) (design. by Nannfeldt and Lindeberg 1957: 500); isolectotypes in Mycoth. Fenn., no. 36/a.

Fig. 7A–F

**Infection** local. **Sori** in some female flowers, around aborted nuts as subglobose to ovoid hard bodies, 0.7–1.2 mm long, initially covered by a thin, grayish peridium that later flakes away exposing a black spore mass, powdery on the surface. **Spores** small-sized, flattened, in plane view suborbicular, broadly elliptical, irregularly rounded, subpolygonal or ovate in outline, in plane view (12–)13–19.5(–21.5) × (11–)12–18(–20) (16.6 ± 1.7 × 15.0 ± 1.6) µm (n/_1_ = 100), in side view 9–12.5 µm thick, medium reddish brown; wall slightly unevenly thickened, 1.0–1.8(–2.2) µm thick, with 1–4(–5) well-developed internal swellings, light refractive areas and protuberances absent; minutely verruculose, warts up to 0.2(–0.3) µm high, spore profile not affected or very slightly affected. In SEM warts often partly confluent, forming short rows or small groups. **Spore germination** of Anthracoidea-type (after Lehtola 1940; Nannfeldt and Lindeberg 1965), resulting in a two-celled basidium, producing ellipsoidal basidiospores with small sizes (mean 8.2 × 2.1 µm).

**Figure 7.**
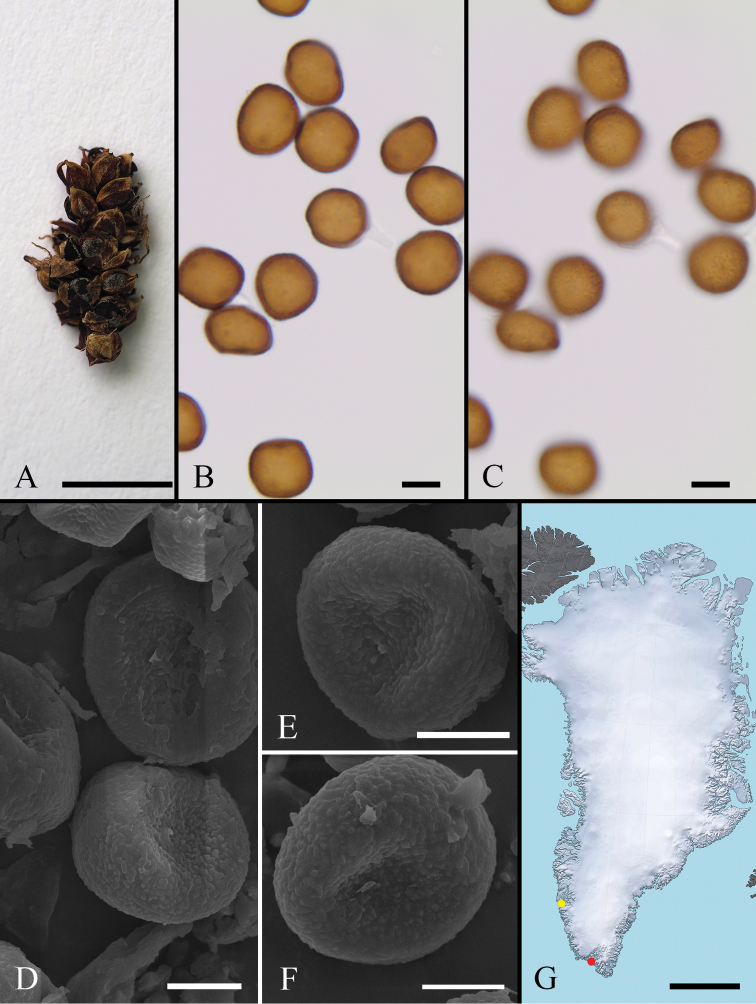
Anthracoidea heterospora on Carex nigra (leg. C.A. Jørgensen, s.n., C-Greenland herb., s.n.). **A** Habit **B, C** spores in LM, in median and surface view, respectively **D–F** spores in SEM**G** distribution map (red circle – on Carex nigra; yellow circle – on a hybrid of C. bigelowii). Scale bars: 0.5 cm (**A**); 10 µm (**B, C**); 5 µm (**D–F**); 500 km (**G**).

**Hosts and distribution within the studied area** — On Cyperaceae: Carex sect. Phacocystis: hybrids of Carex bigelowii – West Greenland; C. nigra – West Greenland (Fig. 7G).

**Specimens examined or recorded.**

On a **hybrid** of **Carex bigelowii**:

**WG**, Ameralik Fjord, 64°03'N, August 1830, leg. J. Vahl, s.n. (C-F-102513!, as ‘*U. caricis* on Carex hyperborea’; Rostrup 1888, as ‘Ci. caricis’; Nannfeldt and Lindeberg 1965: 195 and 203, and Nannfeldt 1979: 20, as ‘A. heterospora on Carex bigelowii × ?’).

On **Carex nigra** (L.) Reichard:

**WG**, Qaqortoq (as ‘Julianehåb district’), Vraget, ca 60°43'20"N, 46°02'25"W, 21 Jul 1956, leg. C.A. Jørgensen, s.n. (C-Greenland herb.!, s.n.).

**Known hosts** — On Cyperaceae: Carex sect. Phacocystis: Carex acuta L., C. andersonii Boott, C. angustata Boott (C. stricta var. angustata (Boott) L.H. Bailey), C. appendiculata (Trautv. & C.A. Mey.) Kük., C. aquatilis Wahlenb. var. aquatilis, C. aquatilisvar. dives (Holm) Kük. (C. sitchensis J.D. Prescott ex Bong.), C. bigelowii subsp. lugens (Holm) T.V. Egorova (C. lugens Holm), C. bigelowii subsp. ensifolia (Turcz. ex Gorodkov) Holub, C. cespitosa L., C. concolor R. Br. (C. aquatilis var. minor Boott), C. confusa Hamlin (C. ternaria G. Forst., nom. nud.), C. coriacea Hamlin, C. decidua Boott, C. elata All. subsp. elata, C. elata subsp. omskiana (Meinsh.) Jalas (C. omskiana Meinsh.), C. gaudichaudiana Kunth, C. geminata Schkuhr, C. × halophila F. Nyl., C. haydenii Dewey, C. lenticularis Michx., C. lessoniana Steud., C. lyngbyei Hornem. (C. lyngbyei subsp. cryptocarpa (C.A. Mey.) Hultén), C. nigra subsp. nigra, C. nigra subsp. juncea (Fr.) Soó (C. juncella (Fr.) Th. Fr.), C. orbicularis Boott, C. paleacea Schreb. ex Wahlenb., C. ramenskii Kom., C. recta Boott, C. salina Wahlenb. var. salina (C. lanceata Dewey), C. schmidtii Meinsh., C. sinclairii Boott ex Cheeseman, C. stricta Lam., C. subdola Boott, C. trinervis Degl., C. vacillans Drejer.

**General distribution. Europe** (North and Central). **Asia**: Arctic Russia, Russian Far East, Mongolia, China. **North America**: Canada, Greenland, U.S.A. **South America** (Argentina, Chile). **Australasia.**

**Earlier reports from Greenland**: Rostrup (1888, as ‘*U. caricis*’), Nannfeldt and Lindeberg (1965), Nannfeldt (1979).

**Comments** — Anthracoidea heterospora has a bipolar distribution. It is a widely distributed smut fungus (mainly in North Europe and northern North America), reported on many species in the section Phacocystis. The following sedges can be listed as principal hosts: Carex acuta, C. aquatilis, C. cespitosa, C. nigra (type host), C. lyngbyei, and C. trinervis. In North America, A. heterospora is known from numerous localities in Alaska, Yukon, Northwest Territories, British Columbia, Alberta, Saskatchewan, Manitoba, Ontario, Quebec, Labrador, and Newfoundland (Nannfeldt and Lindeberg 1965; Parmelee 1983, 1988).

Three specimens of A. heterospora, on hybrids of Carex bigelowii, have been recorded from Greenland (Nannfeldt and Lindeberg 1965: 195, as A. heterospora on ‘C. bigelowii × ?’). These specimens were revised by us but only one of them was accepted as correctly identified. The other two specimens — East Greenland, Akorninarmiut, Skjoldungenområdet, Dronning Marias Dal, 63°28'N, 41°53'W, 12 Aug 1932, leg. J. Devold & P.F. Scholander, s.n. (O!, s.n.; Hagen 1947, as ‘Ci. caricis’ on ‘Carex rigida Good.’); ditto, 24 Jul 1932, leg. J. Devold & P.F. Scholander, s.n. (O!, s.n., as ‘Ci. caricis’ on ‘Carex rigida ad *haematolepidem* Drejer’; Hagen 1947, as ‘Ci. caricis’ on ‘Carex rigida Good.’) — cannot be reliably referred to A. heterospora due to the presence of slightly higher spore wall ornamentation than is typical for A. heterospora; higher value of the minimum spore length, (15–)16 µm versus (12–)13 µm for A. heterospora; and slightly higher values of the mean spore length and width, (18.2 ± 1.0 × 16.7 ± 1.0 µm), than is typical for A. heterospora.

The description given here is based on the specimen of A. heterospora on C. nigra.

Carex nigra belongs to the European floristic element. This sedge occurs also in Siberia, North Africa, and South Greenland. In East Canada and northeastern U.S.A., it is an alien species (Egorova 1999). Carex nigra is recorded here for the first time as a host of A. heterospora in Greenland.

#### 7(7) Anthracoidea karii (Liro) Nannf., s. str., Bot. Not. 130: 368, 1977. ≡ Cintractia karii Liro, Mycoth. Fenn., no. 106, 1934. — Holotype on Carex brunnescens, Finland, Lapponia enontekiensis, Enontekiö, Vuontisjärvi, Peltovuoma, 24 Jul 1933, leg. H. Roivainen & J.I. Liro (?); isotypes in Mycoth. Fenn., no. 106.

Fig. 8A–F

**Infection** local. **Sori** in some female flowers, around aborted nuts as subglobose or ovoid hard bodies, 1.0–2.0 mm long, initially covered by a thin, grayish peridium that later flakes away exposing a black spore mass, powdery on the surface. **Spores** small-sized, flattened, in plane view suborbicular, broadly elliptical, orbicular or ovate in outline, in plane view (14–)15–20(–21) × (13–)14–18(–19) (17.6 ± 1.0 × 16.0 ± 1.0) µm (n/_3_ = 300), in side view 9.5–13 µm thick, medium reddish brown; wall evenly or slightly unevenly thickened, 0.8–1.4(–1.7) µm thick, with 1–4(–5), usually conspicuous internal swellings, light refractive areas and protuberances absent; minutely verruculose, spore profile not affected. In SEM spore wall punctate to minutely verruculose, ornaments up to 0.15 µm high, solitary or partly confluent, forming short rows or small groups. **Spore germination** (after Nannfeldt 1977) of Anthracoidea-type.

**Figure 8.**
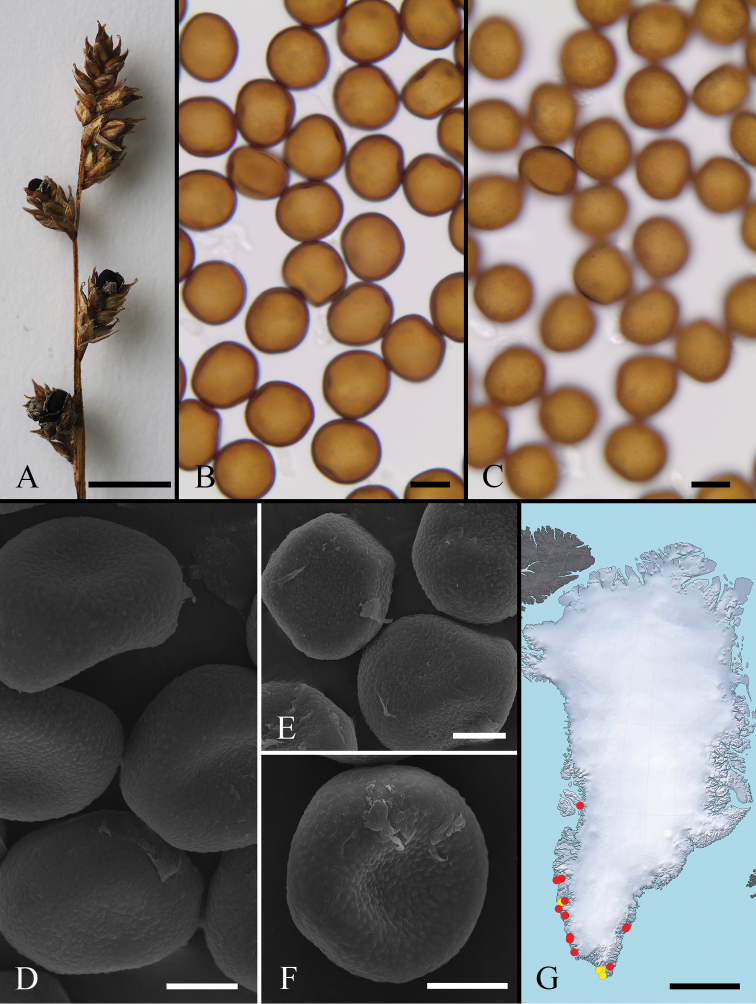
Anthracoidea karii on Carex brunnescens. **A** Habit (C-F-107980) **B, C** spores in LM, in median and surface view, respectively (S. Lægaard, no. 65-3190, C-Greenland herb., s.n.) **D–F** spores in SEM (**D** S. Lægaard, no. 65-3190, C-Greenland herb., s.n.; **E** H. Andersen & J. Feilberg, no. G.B.U. 4245, C-Greenland herb., s.n.; **F** A.E. Porsild, no. 8058, C-Greenland herb., s.n.) **G** distribution map (red circles – on Carex brunnescens; yellow circles – on a C. canescens). Scale bars: 0.5 cm (**A**); 10 µm (**B, C**); 5 µm (**D–F**); 500 km (**G**).

**Hosts and distribution within the studied area** — On Cyperaceae: Carex sect. Glareosae: Carex brunnescens – West and East Greenland; C. canescens – West Greenland (Fig. 8G).

**Specimens examined or recorded.**

On **Carex brunnescens** (Pers.) Poir.:

**WG**, Nûgssuaq, Qôrorssuaq, 70°04'N, 51°16'W, 16 Jul 1948, leg. K. Jakobsen, no. 2222 (C-Greenland herb.!, s.n.).

**WG**, head of Søndre Isortoq, head of Kangerdlug, 65°34'N, 51°57'W, alt. 10 m, 20 Jul 1977, leg. M. Hansen & S. Holt, no. 185 (C-Greenland herb.!, s.n.).

**WG**, near Sukkertoppen, 65°25'N, 52°40'W, 4 Aug 1940, leg. A.E. Porsild, no. 8058 (C-Greenland herb.!, s.n.).

**WG**, Umanap Suvdlua, Qorqut, W of river, 64°16'N, 50°54'W, 15 Aug 1987, leg. H.F. Gøtzsche, no. HFG 87.076 (C-F-107980!).

**WG**, Færingehavn, 63°42'N, 51°33'W, 12 Jun 1947, leg. K. Jakobsen, no. 63 (C-Greenland herb.!, s.n.).

**WG**, Qeqertarsuatsiaat (Fiskenæsset), Navdlúnguaq, Grædefjorden, 63°22'N, 50°28'W, 28 Jul 1972, leg. H. Andersen & J. Feilberg, no. G.B.U. 4245 (C-Greenland herb.!, s.n.).

**WG**, Nigerdleq, 62°05'N, 49°20'W, 23–24 Jul 1963, leg. K. Damsholt & K. Holmen, no. F. 238 (C-Greenland herb.!, s.n.).

**WG**, Kangerdluarssukasik, 61°55'N, 49°18'W, 14 Aug 1965, leg. S. Lægaard, no. 65-3190 (C-Greenland herb.!, s.n.).

**WG**, Arsuk Fjord, ca 61°10'N, 48°27'W, 11 Aug 1952, leg. N. Kjølsen, s.n. (C-Greenland herb.!, s.n.).

**EG**, NW of Griffenfeldt Island (as ‘Umanak’), N of Sehesteds Fjord, Claradalen, ca 63°08'N, 12 Sep 1932, leg. J. Devold, s.n. (O!, s.n., as ‘Ci. caricis’; Hagen 1947, as ‘Ci. caricis’; rev. J.A. Nannfeldt, as ‘Ci. karii’); **ditto**, NW of Griffenfeldt Island, Inn Fjord, ca 63°03'N, 11 Sep 1932, leg. J. Devold & P.F. Scholander, s.n. (O!, s.n., as ‘Ci. caricis’; Hagen 1947, as ‘Ci. caricis’; rev. J.A. Nannfeldt, as ‘Ci. karii’).

**EG**, Lindenow Fjord, Narsak, ca 60°32'N, 43°32'W, 29 Jul 1932, leg. J. Devold & P.F. Scholander, s.n. (O!, s.n., as ‘Ci. caricis’; Hagen 1947, as ‘Ci. caricis’; rev. J.A. Nannfeldt, as ‘Ci. karii’).

On **Carex canescens** L.:

**WG**, Umanap Suvdlua, Qorqut, W of hotel, 64°16'N, 50°52'W, 15 Aug 1987, leg. H.F. Gøtzsche, no. HFG 87.077 (C-F-107983!).

**WG**, Kobbefjord, Nuuk Basic, 64°08'N, 51°23'W, 24 Aug 2018, leg. H. Knudsen, no. HK 18.313 (C-F-111311!).

**WG**, plantation at Tasiusaq, 60°16'N, 44°33'W, 3 Aug 1984, leg. T. Læssøe, no. TL 84.422 (C-F-108010!).

**WG**, Tasermiut, 60°13'N, 5 Sep 1889, leg. N. Hartz, s.n. (C-Greenland herb.!, s.n.).

**WG**, Tôrnârssuk, Nûa, 59°54'N, 44°21'W, 1 Aug 1967, leg. C. Hansen et al., no. 67-1792 (C-Greenland herb.!, s.n.).

**Known hosts** — On Cyperaceae: Carex sect. Glareosae G. Don: Carex brunnescens, C. canescens, C. glareosa Schkuhr ex Wahlenb., C. heleonastes L. f., C. lachenalii Schkuhr, C. lapponica O. Lang, C. loliacea L., C. marina Dewey, C. tenuiflora Wahlenb., C. trisperma Dewey, C. ursina Dewey, and hybrids.

In its broader circumscription, this fungus is also reported on sedges in sect. *Dispermae – C. disperma* Dewey; sect. Physoglochin: C.davalliana Sm., C. dioica L., C. gynocrates Wormsk., C. parallela (Laest.) Sommerf., and hybrids; sect. Stellulatae: C. echinata Murray (C. cephalantha (L.H. Bailey) E.P. Bicknell), C. interior L.H. Bailey, C. omiana Franch. & Sav. var. omiana, and C. omiana var. monticola Ohwi (Nannfeldt 1979; Vánky 2011; Denchev et al. 2013).

**General distribution** (in its broader circumscription). **Europe**: Iceland, the Faeroes, UK, Norway, Sweden, Finland, northern European Russia, Denmark, Latvia, Lithuania, France, Germany, Poland, Switzerland, Austria, Czech Republic, Hungary, Romania, Ukraine, Italy, Bulgaria. **Asia**: West Siberia, Russian Far East, Japan, Mongolia, China. **North America**: Alaska, Canada (British Columbia, Manitoba, Ontario, Quebec, Newfoundland), Greenland, northeastern U.S.A.

**Earlier reports from Greenland** (in the broader circumscription of the species): Rostrup (1888, as ‘*U. caricis*’), Clinton (1904, 1906, as ‘Ci. caricis’), Hagen (1947, as ‘Ci. caricis’), Nannfeldt (1977).

**Comments** — Anthracoidea karii, considered in a broad sense, is a circumboreal–polar species with a very wide host range. It is reported to infect 19 sedges, belonging to four sections of Carex subg. Vignea (Nannfeldt 1977; Vánky 2011; Denchev et al. 2013), among which the following can be considered as principal hosts: in sect. Glareosae — C. brunnescens and C. lachenalii, in *sect.*Physoglochin — C. dioica and C. parallela, and in sect. Stellulatae — C. echinata.

For the purpose of this treatment, many Greenlandic specimens of infected sedges of the host range of A. karii were studied, but only that on C. brunnescens and C. canescens were accepted as belonging to A. karii. The Anthracoidea specimens on other sedges do not fit well the characters of A. karii on its type host, C. brunnescens, observed by us in comparative specimens from Europe. The excluded specimens (see the list below) possess spores with similar shape, sizes, and wall thickness but variable in height of the spore wall ornamentation, and number and conspicuousness of the internal swellings. For this reason, the fungus in the current study is accepted in a strict sense. The description given here is based on specimens of A. karii on C. brunnescens. Molecular data are needed for clarification of the taxonomic status of the Anthracoidea species on Carex glareosa, C. gynocrates, C. lachenalii, and C. paralella.

For the time being, the following Anthracoidea specimens cannot be reliably referred to A. karii:

On **Carex glareosa** Schkuhr ex Wahlenb.:

**WG**, Sisimiut (as ‘Holstensborg’), 66°56'N, 31 Jul 1871, leg. Th.M. Fries, s.n. (O-V-670026!; Hagen 1947, as ‘Ci. caricis’).

**WG**, Maniitsoq (as ‘Sukkertoppen’), 65°25'N, 1886, leg. Th. Holm, s.n. (n.v.; not found in C; Rostrup 1888, as ‘*U. caricis*’).

**WG**, Kangikitsup, Qingua, 60°18'N, 24 Jul 1925, leg. A.E. Porsild & M.P. Porsild, s.n. (C-Greenland herb.!, s.n.).

**EG**, Akorninarmiut, Imarsivik Island at Krappsundet, ca 63°22'N, 41°08'W, 24 Aug 1931, leg. B. Bjørlykke, The Norwegian Expedition to Southeast Greenland 1931, s.n. (O-V-670174!; Hagen 1947, as ‘Ci. caricis’).

**EG**, Akorninarmiut, Skjoldungenområdet, Dronning Marias Dal, ca 63°28'N, 41°53'W, 5 Aug 1931, leg. B. Bjørlykke, The Norwegian Expedition to Southeast Greenland 1931, s.n. (O!, s.n.; Hagen 1947, as ‘Ci. caricis’).

**EG**, Sehesteds Fjord, NW of Griffenfeldt Island (as ‘Umanak’), Pilerkit, ca 63°12'N, 42°08'W, 14 Aug 1931, leg. B. Bjørlykke, s.n. (n.v.; not found in O; Hagen 1947, as ‘Ci. caricis’).

**EG**, Timmiarmiit Island (as ‘Tingmiarmiut’), Lomvatnet, ca 62°47'N, 42°18'W, 2 Aug 1931, leg. B. Bjørlykke, The Norwegian Expedition to Southeast Greenland 1931, s.n. (O-V-670176!; Hagen 1947, as ‘Ci. caricis’); **ditto**, Brattneset, ca 62°43'N, 42°20'W, 8 Aug 1932, leg. J. Devold & P.F. Scholander, s.n. (n.v.; not found in O; Hagen 1947, as ‘Ci. caricis’).

**EG**, Nenese, 60°28'N, July 1829, leg. J. Vahl, s.n. (C-F-102512!, as ‘*U. caricis*’; Rostrup 1888, as ‘*U. caricis*’; rev. J.A. Nannfeldt, as ‘Ci. karii’).

On **Carex gynocrates** Wormsk.:

**WG**, Nûgssuaq, Sarqaqdalen, 70°04'N, 52°07'W, 10 Jul 1948, leg. K. Jakobsen, no. 2149 (C-Greenland herb.!, s.n).

**WG**, Head of Kangerlussuaq, 1 km ENE of Qardligssuit, 67°10'N, 50°43'W, alt. 500 m, 8 Jul 1981, leg. S. Holt, no. 2444 (C-Greenland herb.!, s.n.).

**WG**, Sermiligârssuk, 61°32'N, 48°35'W, alt. 50 m, 8 Aug 1965, leg. J. Johansen & K. Hansen, no. 65-272 (C-Greenland herb.!, s.n.).

**WG**, Igaliku, 61°00'N, 45°27'W, alt. 25 m, 24 Jul 1962, leg. B. Fredskild, no. 2234 (C-Greenland herb.!, s.n.).

On **Carex lachenalii** Schkuhr:

**WG**, Disko Island, near Arctic Station, 69°15'N, 14 Sep 1939, leg. M.P. Porsild, s.n. (C-Greenland herb.!, s.n.).

**WG**, Mississippi, head of Bjørnesund, 63°04'N, 49°47'W, 18 Jul 1971, leg. H. Andersen & K. Damsholt, no. G.B.U. 2838 (C-Greenland herb.!, s.n.).

**EG**, Fridtjof Nansens Halvø, SE-end, N of Kiataq, 64°20'N, 40°26'W, 2 Aug 1968, leg. K. Gormsen, s.n. (C-Greenland herb.!, s.n.).

**EG**, Akorninarmiut, Trollfjordeidet, ca 63°30'N, 41°20'W, 13 Aug 1931, leg. B. Bjørlykke, s.n. (O!, s.n.; Hagen 1947, as ‘Ci. caricis’).

**EG**, Akorninarmiut, Skjoldungen Island, inner N side, ca 63°28'N, 41°39'W, 7 Aug 1931, leg. B. Bjørlykke, s.n. (O!, s.n.; Hagen 1947, as ‘Ci. caricis’).

**EG**, Akorninarmiut, Floneset, 63°23'N, 41°10'W, 18 Aug 1932, leg. J. Devold, s.n. (O!, s.n.; Hagen 1947, as ‘Ci. caricis’).

**EG**, Tingmiarmit Island, Tvihamna, ca 62°46'N, 42°25'W, 9 Sep 1932, leg. J. Devold, s.n. (O!, s.n.; Hagen 1947, as ‘Ci. caricis’); **ditto**, Brattneset, ca 62°43'N, 42°20'W, 8 Aug 1932, leg. J. Devold & P.F. Scholander, s.n. (O!, s.n.; Hagen 1947, as ‘Ci. caricis’).

**EG**, Lindenow Fjord, Narsak, ca 60°32'N, 43°32'W, 29 Jul 1932, leg. J. Devold & P.F. Scholander, s.n. (O!, s.n.; Hagen 1947, as ‘Ci. caricis’); **ditto**, Møretun, ca 60°28'N, 43°18'W, 3 Aug 1932, leg. J. Devold & P.F. Scholander, s.n. (O!, s.n.; Hagen 1947, as ‘Ci. caricis’).

On **Carex parallela** (Laest.) Sommerf.:

**EG**, Røde Island, 70°28'N, 28°05'W, 15 Aug 1891, leg. N. Hartz, Expeditio Danica in Groenlandiam orientalem 1891–1892, s.n. (C-Greenland herb.!, s.n.).

**EG**, Scoresby Sund, Ispynt (on the north coast of inner Vestfjord, as ‘Isfjord’), 70°27'N, 29°10'W, 10 May 1892, leg. N. Hartz, Expeditio Danica in Groenlandiam orientalem 1891–1892, s.n. (C-Greenland herb.!, s.n.).

From Greenland, Anthracoidea karii is also reported on Carex canescens × C. lachenalii (Nannfeldt 1977) and C. ursina (Fischer 1953, as ‘Ci. caricis’; Nannfeldt 1977) but no voucher specimens were cited or are known to exist.

#### 8(8) Anthracoidea limosa (Syd.) Kukkonen, Ann. Bot. Soc. Zool.-Bot. Fenn. ‘Vanamo’ 34(3): 91, 1963. ≡ Cintractia limosa Syd., Ann. Mycol. 22: 288, 1924. — Lectotype on Carex limosa, Norway, Troms, near Tromsö, Aug 1894, leg. G. Lagerheim, s.n. (UPS) (design. by Kukkonen 1963: 91); isolectotypes in Sydow, Ustilag., no. 76 (as ‘Cintractia caricis’).

Fig. 9A–E

= Cintractia subglobosa S. Ito, Trans. Sapporo Nat. Hist. Soc. 14: 92, 1935. — Holotype on Carex limosa (as ‘C. limosa var. fuscocuprea’), Hokkaido, Nemuro-shi, Shunkuntan, 17 Aug 1924, leg. M. Tatewaki, s.n. (SAPA, s.n.!) (syn. by Nannfeldt 1979: 28).

= Cintractia gigantissima Lehtola, Acta Agralia Fenn. 42(1): 129, 1940. ≡ C. limosa var. gigantissima (Lehtola) Savile, Canad. J. Bot. 30: 426, 1952. — Type on Carex rariflora, Finland, Russian Karelia, Siberia, and Norway; no special collection designated (syn. by Kukkonen 1963: 91).

**Infection** local. **Sori** in some female flowers, around aborted nuts as ovoid or broadly ellipsoidal hard bodies, 2.0–3.0 mm long, initially covered by a thin, grayish peridium that later flakes away exposing a black spore mass, powdery on the surface. **Spores** large-sized, slightly flattened, in plane view suborbicular, broadly elliptical, slightly irregular, subpolygonal or ovate in outline, in plane view (20.5–)22–31(–34) × (18–)19–25.5(–27) (26.3 ± 2.3 × 23.0 ± 1.8) µm (n/_1_ = 100), in side view 14.5–20 µm thick, medium or dark reddish brown; wall slightly unevenly thickened, (1.0–)1.2–2.0(–2.4) µm thick, internal swellings, light refractive areas, and protuberances absent; minutely to moderately verruculose, warts up to 0.4 µm high, spore profile not affected or slightly affected. In SEM warts partly confluent, forming short rows or small groups. **Spore germination** of Proceres-type (after Kukkonen 1963: 92, Figs 36–38), resulting in a two-celled basidium, 250–350 μm long, the apical cell 40–70 × 3–6 μm; producing cylindrical basidiospores, straight or slightly curved, 32–104 × 3–7 μm.

**Figure 9.**
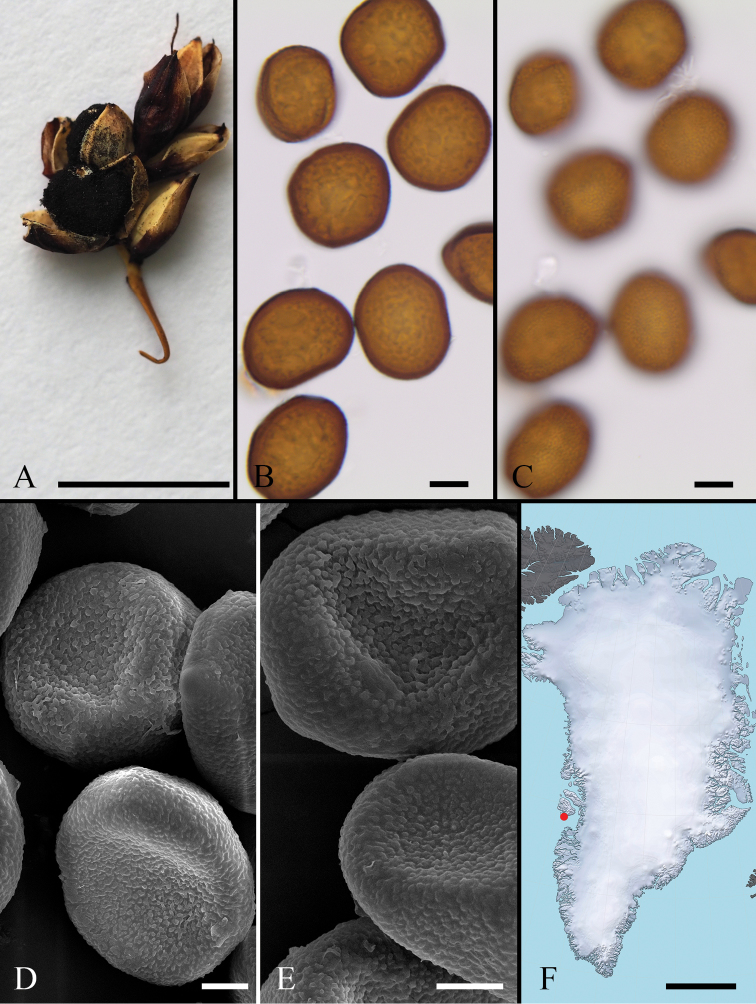
Anthracoidea limosa on Carex rariflora (GZU 000323439). **A** Habit **B, C** spores in LM, in median and surface view, respectively **D, E** spores in SEM**F** distribution map. Scale bars: 0.5 cm (**A**); 10 µm (**B, C**); 5 µm (**D, E**); 500 km (**F**).

**Hosts and distribution within the studied area** — On Cyperaceae: Carex sect. Limosae Meinsh.: Carex rariflora – West Greenland (Fig. 9G).

**Specimens examined or recorded.**

On **Carex rariflora** (Wahlenb.) Sm.:

**WG**, Disko Island, SW of Qeqertarsuaq (as ‘Godhavn’), ca 69°14'50"N, 53°32'W, 12 Aug 1982, leg. J. Poelt & H. Ullrich, s.n. (GZU 000323439!).

**Known hosts** — On Cyperaceae: Carex sect. Limosae: Carex limosa L., C. magellanica subsp. irrigua (Wahlenb.) Hiitonen (C. paupercula Michx.), C. pluriflora Hultén (C. rariflora subsp. pluriflora (Hultén) T.V. Egorova), C. rariflora, and hybrids between some of these species.

**General distribution. Europe**: Iceland, UK, Norway, Sweden, Finland, European Russia, Denmark, Estonia, Latvia, Lithuania, Germany, Poland, Switzerland, Austria, Czech Republic, Ukraine, Italy. **Asia**: East Siberia, Russian Far East, Japan. **North America**: Alaska, Canada, Greenland, northeastern U.S.A.

**Comments** — The principal host of Anthracoidea limosa, Carex limosa, is a circumboreal species, occurring in Europe, Caucasus, Siberia, Russian Far East, Sakhalin, Kuriles, Japan, North Korea, Mongolia, NE China, and North America (Canada and U.S.A.) (Hultén and Fries 1986: 497; Egorova 1999). It is a widely distributed species across Canada, U.S.A., and northern Eurasia. On this sedge, A. limosa is reported from North and Central Europe, European part of Russia, East Siberia, Russian Far East, Kuriles, Japan, Canada, and U.S.A. (Denchev and Minter 2011a; Denchev et al. 2013).

Carex rariflora is a circumpolar species, distributed in North Europe, Arctic Russia, Siberia, Russian Far East, Alaska, Canada, and Greenland (Hultén and Fries 1986: 498; Egorova 1999; Elven et al. 2018; Govaerts 2018). Records of A. limosa on C. rariflora are known from Norway, Sweden, Finland, Russian northeastern Arctic and Far East, northern Kuriles, Alaska, and Canada (Kukkonen 1963; Govorova 1987, 1990; Azbukina et al. 1995; Scholler et al. 2003).

In Canada, Anthracoidea limosa is reported from Yukon, Northwest Territories, Nunavut, British Columbia, Alberta, Saskatchewan, Manitoba, Ontario, Quebec, Labrador, Newfoundland, New Brunswick, and Prince Edward Island; found on Carex limosa, C.magellanica subsp. irrigua, C. pluriflora, C. rariflora, and hybrids between some of them (Savile 1952; Kukkonen 1963; Parmelee 1983, 1988).

Anthracoidea limosa is a circumboreal–polar species. Further studies must be carried out in order to determine whether or not the eastern Asian and North American specimens belong to distinct species (Denchev et al. 2013).

Anthracoidea limosa is reported here for the first time from Greenland.

#### 9(9) Anthracoidea lindebergiae (Kukkonen) Kukkonen, Ann. Bot. Soc. Zool.-Bot. Fenn. ‘Vanamo’ 34(3): 68, 1963. ≡ Cintractia lindebergiae Kukkonen, Canad. J. Bot. 39: 161, 1961. — Holotype on Kobresia simpliciuscula, Canada, Nunavut, Baffin Island, Frobisher Bay, on sea beach near airport, 11 Aug 1959, leg. D.B.O. Savile et al. (TUR); isotype in DAOM.

Fig. 10A–F

**Infection** local. **Sori** in some female flowers, around aborted nuts as subglobose to ovoid hard bodies, 0.5–1 mm long, initially covered by a thin, grayish peridium that later flakes away exposing a black spore mass, powdery on the surface. **Spores** small-sized, flattened, in plane view suborbicular, orbicular, ovate or broadly elliptical in outline, sometimes slightly irregularly rounded, in plane view (14.5–)15.5–20(–21) × (13.5–)14–17.5(–18.5) (17.4 ± 1.0 × 16.1 ± 0.9) µm (n/_3_ = 300), in side view 9–12.5 µm thick, medium or dark reddish brown; wall evenly or slightly unevenly thickened, 1.0–1.8(–2.0) µm thick, internal swellings, light refractive areas, and protuberances absent; minutely verruculose, warts up to 0.2(–0.3) µm high, spore profile not affected or very slightly affected. In SEM spore wall sparsely minutely verruculose. **Spore germination** of Anthracoidea-type (after Kukkonen 1963), resulting in a two-celled basidium, 100–150 μm long, the apical cell 40–50 μm long and 3.5–4.2 μm thick; producing 1–3 basidiospores on each cell; basidiospores cylindrical or narrowly ellipsoidal, ovoid or obovoid, 8–16 × 3.5–6 μm.

**Figure 10.**
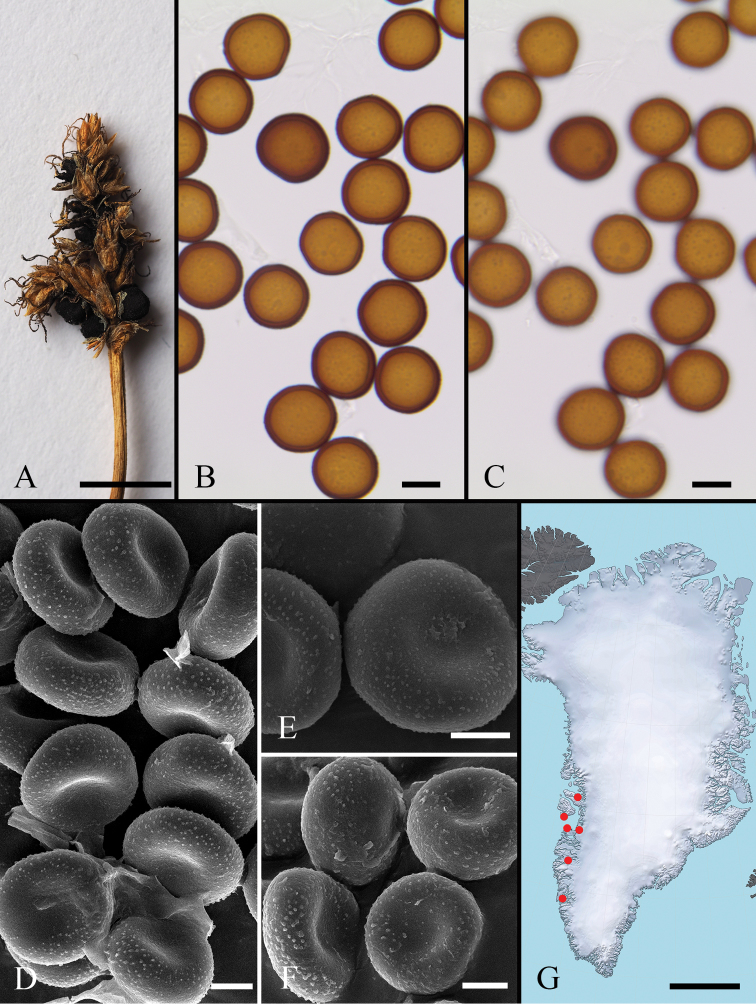
Anthracoidea lindebergiae on Carex simpliciuscula. **A** Habit (K. Jakobsen, no. 1779, C-Greenland herb., s.n.) **B, C** spores in LM, in median and surface view, respectively (K. Jakobsen, no. 1779, C-Greenland herb., s.n.) **D–F** spores in SEM (**D, E** T.W. Böcher, The Botanical Expedition to West Greenland 1958, no. 1463, C-Greenland herb., s.n.; **F** K. Jakobsen, no. 1779, C-Greenland herb., s.n.) **G** distribution map. Scale bars: 0.5 cm (**A**); 10 µm (**B, C**); 5 µm (**D–F**); 500 km (**G**).

**Hosts and distribution within the studied area** — On Cyperaceae: Carex (the Simpliciuscula clade): Carex simpliciuscula – West Greenland (Fig. 10G).

**Specimens examined or recorded.**

On **Carex simpliciuscula** Wahlenb. (syn. Kobresia simpliciuscula (Wahlenb.) Mack.) (see the comments to this smut fungus):

**WG**, Nûgssuaq, Umlartorfik, 70°31'N, 51°55'W, 19 Aug 1947, leg. K. Jakobsen, no. 1779 (C-Greenland herb.!, s.n.).

**WG**, Disko Island, Qeqertarsuaq (as ‘Godhavn’), 69°15'N, 6 Aug 1923, leg. A.E. Porsild, s.n. (C-Greenland herb.!, s.n.; DAOM, as ‘Ci. lindebergiae’, Kukkonen 1961); **ditto**, ca 69°14'50"N, 53°32'W, 24 Sep 1947, leg. T. Sørensen, no. 306 (C-Greenland herb.!, s.n.).

**WG**, Aasiaat (as ‘Tupilaq, Egedesminde’), ca 68°42'N, 52°52'W, 29 Aug 1958, leg. T.W. Böcher, The Botanical Expedition to West Greenland 1958, no. 1463 (C-Greenland herb.!, s.n.).

**WG**, Sydostbugten, S side of Orpigsûp nunâ, 68°39'N, 50°40–45'W, 22–23 Jul 1981, sine coll., no. 707 (C-Greenland herb.!, s.n.).

**WG**, Søndre Strømfjord, 66°44'N, 18 Sep 1956, leg. T.W. Böcher, no. 148 (C-Greenland herb.!, s.n.).

**WG**, Godthåbsfjord, Itivnera, 64°22'N, 19 Aug 1931, leg. M.P. Porsild, s.n. (C-Greenland herb.!, s.n.; DAOM, as ‘Ci. lindebergiae’, Kukkonen 1961).

**Known hosts** — On Cyperaceae: Carex (the Simpliciuscula clade): Carex alatauensis S.R. Zhang (Kobresia humilis (C.A.Mey. ex Trautv.) Serg.), C. kokanica (Regel) S.R. Zhang (Kobresia royleana (Nees) Boeckeler), C. simpliciuscula (principal host).

**General distribution. Europe**: Norway, Sweden, Switzerland, Austria. **Asia**: Russian Far East, Kazakhstan, Tadzhikistan, China. **North America**: Canada, Greenland, U.S.A.

**Earlier reports from Greenland**: Kukkonen (1961, as ‘Ci. lindebergiae’, 1963).

**Comments** — The host plants of Anthracoidea lindebergiae were previously recognized as members of the genus Kobresia. As already noted in the comments to Anthracoidea elynae, the circumscription of the genus Carex was recently expanded to include all species of the genera *Cymophyllus, Kobresia, Schoenoxiphium*, and Uncinia (Global Carex Group 2015, 2016) that will reflect on the taxonomy of the Anthracoidea species on hosts, previously recognized as members of Kobresia, as a distinct genus.

In the *Flora of North America* treatment (Ball 2002a), Kobresia simpliciuscula (a principal host of A. lindebergiae) is considered in a broad sense, as a circumpolar–alpine species, without recognition of distinct infraspecific taxa. In Egorova’s taxonomic revision of Kobresia in Russia (1983), three subspecies were distinguished: subsp. simpliciuscula, accepted as a European taxon; K. simpliciuscula subsp. subholarctica T.V. Egorova, described there as distributed in Arctic Russia, Siberia, Russian Far East, and North America; and K. simpliciuscula subsp. subfilifolia (T.V. Egorova et al.) T.V. Egorova, as endemic to the northeastern Russian Arctic and Subarctic. Currently, two subspecies are recognized within Carex simpliciuscula: subsp. simpliciuscula, a mainland European taxon, not distributed in the Arctic, and subsp. subholarctica, an Asian (northeastern)–amphi-Beringian–North American (northern)–amphi-Atlantic taxon, distributed in Siberia, Russian Far East, Alaska, Canada, Greenland, and Svalbard (Saarela et al. 2017; Alsos et al. 2018; Elven et al. 2018); while subsp. subfilifolia is accepted within Kobresia filifolia, as an Asian (northeastern)–amphi-Beringian taxon (Elven et al. 2018). As the species of Kobresia are now recognized in Carex (Global Carex Group 2015) and the correct name for K. filifolia in Carex is C. macroprophylla (Y.C. Yang) S.R. Zhang (op. c.), a new combination for subsp. subfilifolia is necessary to be proposed:

**Carex macroprophylla** subsp. **subfilifolia** (T.V. Egorova, Jurtzev & V.V. Petrovsky) Denchev & T. Denchev, **comb. nov.** — Basionym: Kobresia filifolia subsp. subfilifolia T.V. Egorova, Jurtzev & V.V. Petrovsky, Bot. Zhurn. (Moscow & Leningrad) 66: 1042, 1981.

The host plant of Anthracoidea lindebergiae in Greenland should be accepted as belonging to Carex simpliciuscula subsp. subholarctica (T.V. Egorova) Saarela.

On Carex simpliciuscula subsp. simpliciuscula, Anthracoidea lindebergiae is known from Norway, Sweden, and the Alps (in Switzerland and Austria) (Kukkonen (1961, as ‘Ci. lindebergiae’; Jørstad 1963; Gjærum 1972; Nannfeldt 1979; Zwetko and Blanz 2004).

On Carex simpliciuscula subsp. subholarctica (as ‘Kobresia simpliciuscula’ s. lat.), this smut fungus has been previously reported from the Russian Far East, Alaska, Canada (Nunavut, British Columbia, Manitoba, and Quebec), Greenland, and Svalbard (Lind 1928, as ‘Ci. caricis’ on ‘Cobresia caricina’; Linder 1947, as ‘Ci. caricis’; Savile 1952, as ‘Ci. carpophylla var. elynae’; Kukkonen 1961, as ‘Ci. lindebergiae’, 1963; Parmelee 1983; Karatygin and Azbukina 1989; Govorova 1990; Elvebakk et al. 1996).

Taking into account some records on other hosts from Central Asia and China (e.g., Gutner 1941, as ‘Ci. elynae’; Schwarzman 1960, as ‘Ci. elynae’; Kukkonen 1961, as ‘Ci. lindebergiae’; Guo 1994, 2006), which are in need of additional revision, Anthracoidea lindebergiae should be considered as a circumpolar–alpine species.

#### 10(10) Anthracoidea liroi (Lehtola) Nannf. & B. Lindeb., Svensk Bot. Tidskr. 59: 205, 1965. ≡ Cintractia liroi Lehtola, Acta Agralia Fenn. 42(1): 46, 1940. — Neotype on Carex bigelowii × C. nigra, Finland, Lapponia enontekiensis, Kilpisjärvi, July 1934, H. Roivainen & J.I. Liro (H.U.V. 158) (design. by Vánky 1990: 274); isoneotypes in Mycoth. Fenn., no. 104 (as ‘Cintractia caricis’ on ‘Carex rigida’, = Carex bigelowii × C. nigra, comp. Nannfeldt and Lindeberg 1965: 206).

Fig. 11A–E

**Infection** local. **Sori** in some female flowers, around aborted nuts as subglobose to ovoid hard bodies, 1.0–1.5 mm long, initially covered by a thin, grayish peridium that later flakes away exposing a black spore mass, powdery on the surface. **Spores** medium- to large-sized, flattened, in plane view irregularly rounded, suborbicular, broadly elliptical, subpolygonal or ovate in outline, in plane view (18–)19–25.5(–28) × (16–)17–22(–24) (22.2 ± 1.7 × 19.8 ± 1.4) µm (n/_1_ = 100), in side view 12.5–15.5 µm thick, dark reddish brown; wall slightly unevenly thickened, 1.0–1.7(–2.0) µm thick, occasionally a weak internal swelling may be present, light refractive areas and protuberances absent; minutely verruculose, warts up to 0.2(–0.3) µm high, spore profile not affected or sometimes very slightly affected. In SEM warts often partly confluent. **Spore germination** of Proceres-type (after Nannfeldt and Lindeberg 1965), resulting in a two-celled basidium, producing narrowly cylindrical basidiospores, straight or slightly curved and very long (M = 55 × 5.6 µm).

**Figure 11.**
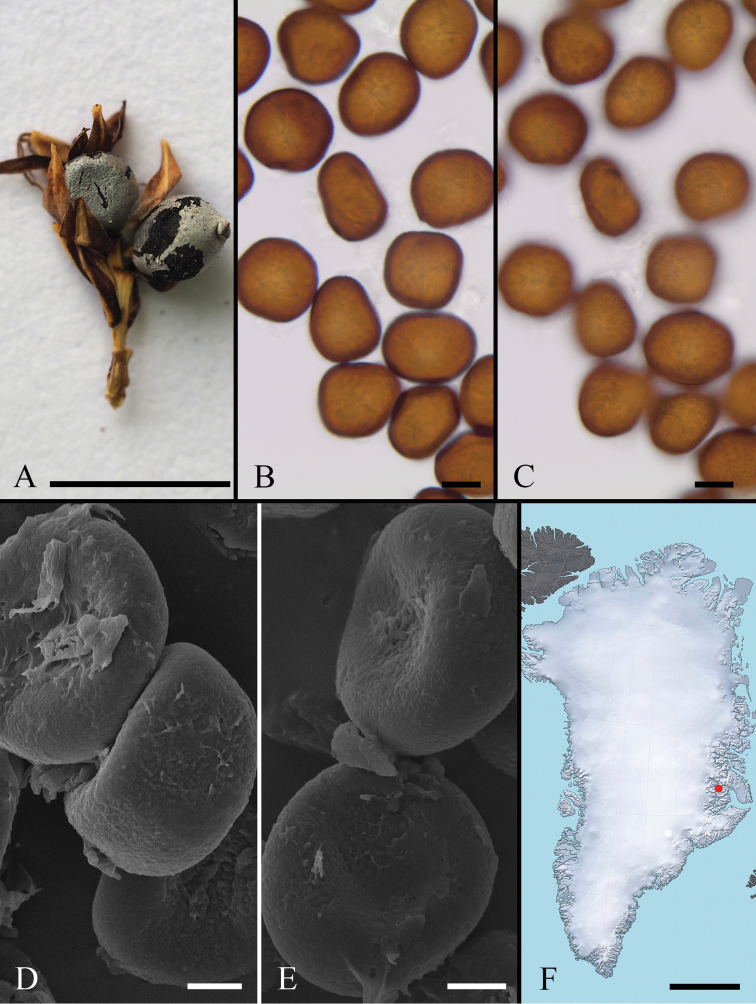
Anthracoidea liroi on Carex subspathacea (leg. G. Halliday, s.n., E, s.n.). **A** Habit **B, C** spores in LM, in median and surface view, respectively **D, E** spores in SEM**F** distribution map. Scale bars: 0.5 cm (**A**); 10 µm (**B, C**); 5 µm (**D, E**); 500 km (**F**).

**Hosts and distribution within the studied area** — On Cyperaceae: Carex sect. Phacocystis: Carex subspathacea – East Greenland (Fig. 11F).

**Specimens examined or recorded.**

On **Carex subspathacea** Wormsk. ex Hornem.:

**EG**, Renland near Nordvestfjord, on flats at head of Edvard Bay, 71°27'N, 27°13'W, 7 Aug 1971, leg. G. Halliday, s.n. (E!, s.n.).

**Known hosts** — On Cyperaceae: Carex sect. Phacocystis: Carex aquatilis Wahlenb., C. bigelowii subsp. ensifolia (Turcz. ex Gorodkov) Holub (C. lugens H.T. Holm), C. cespitosa L., C. concolor R. Br. (C. aquatilis var. minor Boott), C. elata All., C. lyngbyei Hornem. subsp. lyngbyei, C. lyngbyei subsp. cryptocarpa (C.A. Mey.) Hultén, C. middendorffii F. Schmidt, C. nigra (L.) Reichard subsp. nigra, C. nigra subsp. juncea (Fr.) Soó (C. nigra subsp. juncella (Fr.) Lemke), C. salina Wahlenb. var. salina (C. lanceata Dewey), C. schmidtii Meinsh., C. subspathacea Wormsk. ex Hornem., and hybrids (C. aquatilis × C. bigelowii, C. aquatilis × C. nigra, C. aquatilis × C. nigra subsp. juncea, C. aquatilis × C. paleacea, C. aquatilis × C.salina, C. aquatilis × C. subspathacea, C. bigelowii × C. nigra, C. bigelowii × C. nigra subsp. juncea, C. elata × C. nigra, C. lyngbyei × C. nigra, C. nigra × C. subspathacea).

**General distribution. North Europe**: Svalbard, Iceland, Faeroes, UK, Norway, Sweden, Finland, Denmark, Estonia, North and Arctic Russia. **Asia**: East Siberia, Arctic Russia, Russian Far East. **North America**: Arctic and Subarctic Canada.

**Comments** — Anthracoidea liroi is a circumpolar species. It attacks many sedges in Carex sect. Phacocystis, but, as pointed out in Nannfeldt (1979: 28), designation of principal hosts for this smut fungus would be meaningless for the following reasons. A significant number of the specimens of A. liroi, stored in the collections, are on hybrids (e.g., more than half of the Swedish specimens, studied by Nannfeldt and Lindeberg), and this material often is insufficient for an exact identification of the host (cfr Nannfeldt and Lindeberg 1965: 206). In assessing the significance of the hosts, the ecological requirements of this smut fungus should be taken into account. Anthracoidea liroi is a northern (without records outside the northern countries), montane, and maritime species, while A. heterospora (on sedges in the same section) follows on the whole the distribution of its hosts, except for the far North (Nannfeldt and Lindeberg 1965; Nannfeldt 1979).

Carex subspathacea is a circumpolar species, occurring in Svalbard, Iceland, Norway, Arctic Russia, Siberia, Russian Far East, Hokkaido, Alaska, Canada, and Greenland (Egorova 1999). On this sedge, Anthracoidea liroi has been previously reported only from Svalbard (Spitsbergen — Hagen 1941; Nannfeldt and Lindeberg 1965; Scholler et al. 2003). A record from Canada (Nunavut, as ‘Cintractia limosa var. limosa’ on ‘Carex salina var. subspathacea’, n.v.; Savile 1952) probably belongs to this smut fungus.

Anthracoidea liroi is reported here as new to Greenland.

#### 11(11) Anthracoidea misandrae Kukkonen, Ann. Bot. Soc. Zool.-Bot. Fenn. ‘Vanamo’ 34(3): 82, 1963. — Holotype on Carex fuliginosa subsp. misandra (as ‘C. misandra’), Canada, Nunavut, Baffin Island, Frobisher Bay, ca 2 miles E of the airport, 10 Aug 1959, D.B.O. Savile et al. (TUR); isotype DAOM 66 933.

Fig. 12A–F

**Infection** local. **Sori** in some female flowers, around aborted nuts as subglobose, ovoid or broadly ellipsoidal hard bodies, 1.0–1.5 mm long, initially covered by a thin, grayish peridium that later flakes away exposing a black spore mass, powdery on the surface. **Spores** medium-sized, flattened, in plane view suborbicular, broadly elliptical, irregularly rounded or ovate in outline, in plane view (17.5–)18.5–24(–25) × (16–)17–21(–22.5) (21.2 ± 1.2 × 19.3 ± 1.0) µm (n/_3_ = 300), in side view 10.5–14.5 µm thick, medium or dark reddish brown; wall usually evenly thickened, 1.0–1.8(–2.0) µm thick, internal swellings, light refractive areas, and protuberances absent; moderately verruculose to verrucose, warts up to 0.5(–0.6) µm high, spore profile affected. In SEM warts often partly confluent, forming short rows or small groups. **Spore germination** unknown.

**Figure 12.**
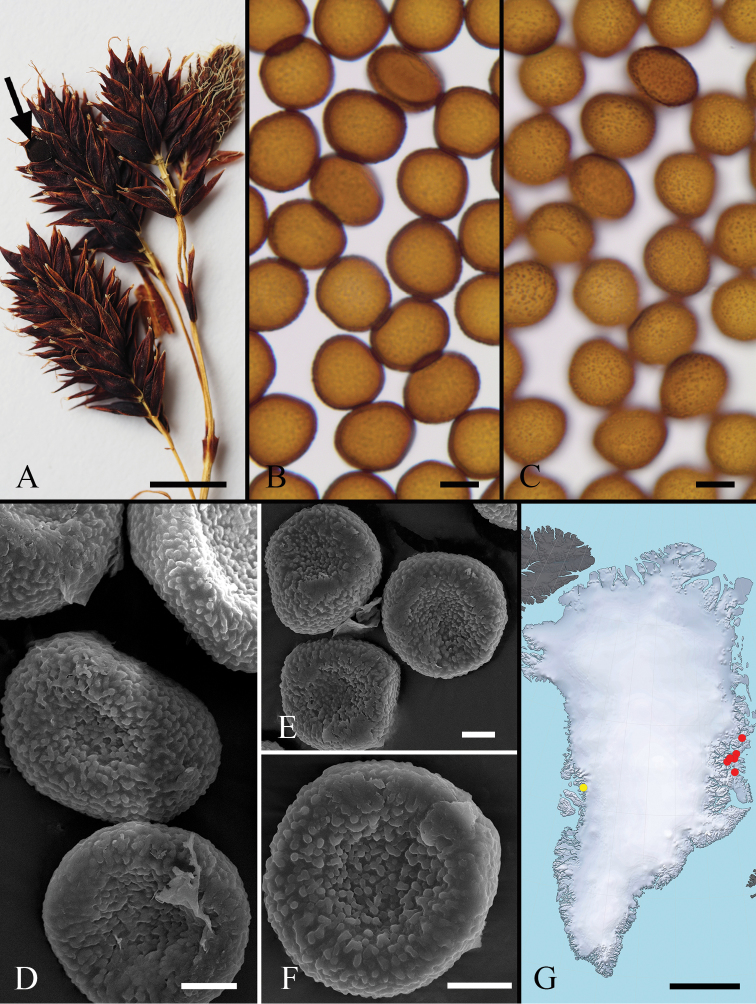
Anthracoidea misandrae on Carex atrofusca (**A–C, F**) and C. fuliginosa subsp. misandra (**D, E**). **A** Habit (24 Jul 1930, leg. J. Vaage, s.n., O, s.n.) **B, C** spores in LM, in median and surface view, respectively (leg. D.R. Spearing & N.P. Lasca, s.n., C-Greenland herb., s.n.) **D–F** spores in SEM (**D, E**GZU 000323449; **F** T. Sørensen, no. 3101, C-Greenland herb., s.n.) **G** distribution map (red circles – on Carex atrofusca; yellow circle – on C. fuliginosa subsp. misandra). Scale bars: 0.5 cm (**A**); 10 µm (**B, C**); 5 µm (**D–F**); 500 km (**G**).

**Hosts and distribution within the studied area** — On Cyperaceae: Carex sect. Aulocystis: Carex atrofusca – East Greenland; C. fuliginosa subsp. misandra – West Greenland (Fig. 12G).

**Specimens examined or recorded.**

On **Carex atrofusca** Schkuhr:

**EG**, Hold with Hope, Kap Stosch, ca 74°03.6'N, 21°43.8'W, 24 Jul 1930, leg. J. Vaage, s.n. (O!, s.n.; Hagen 1947, as ‘Ci. caricis’).

**EG**, Ymer Island, Dusén Fjord W, at the rivulet beneath Skredbergene, 7 Aug 1933, leg. A. Hagen, the Norwegian Expedition to NE Greenland 1933, s.n. (O!, s.n.; Hagen 1947, as ‘Ci. caricis’; dubl. in DAOM, Kukkonen 1963); **ditto**, Dusén Fjord E, near Sandvelta, 7 Aug 1933, leg. A. Hagen, the Norwegian Expedition to NE Greenland 1933, s.n. (O!, s.n.; Hagen 1947, as ‘Ci. caricis’); **ditto**, Kapp Graah near Dusén Fjord, 17 Aug 1929, leg. J. Vaage, s.n. (O!, s.n.; Hagen 1947, as ‘Ci. caricis’); **ditto**, Botanikerbugt, 73°02'N, 8 Aug 1932, leg. T. Sørensen, The Three-year expedition to East Greenland 1931–1933 under the leadership of Dr. L. Koch, no. 3101 (C-Greenland herb.!, s.n.).

**EG**, Geographical Society Island, Sofia Sund, 2–3 km SW of Strømhytta, 21 Aug 1933, leg. A. Hagen, the Norwegian Expedition to NE Greenland 1933, s.n. (O!, s.n.; Hagen 1947, as ‘Ci. caricis’).

**EG**, Ella Island, 72°53'N, 25°10'W, 14 Aug 1950, leg. K. Holmen, s.n. (C-Greenland herb.!, s.n.).

**EG**, northern Stauning Alper, West Skeldal, wet meadow, 72°15'N, 24°W, 29 Aug 1962, leg. D.R. Spearing & N.P. Lasca, s.n. (C-Greenland herb.!, s.n.).

On **Carex fuliginosa** subsp. **misandra** (R. Br.) Nyman:

**WG**, S of Maamorilik, ca 71°07'N, 51°16'W, alt. 30–200 m, 7 Aug 1983, leg. J. Poelt & H. Ullrich, s.n. (GZU 000323449!, the host as ‘C. misandra’).

**Known hosts** — On Cyperaceae: Carex sect. Aulocystis: Carex atrofusca, C. fuliginosa Schkuhr subsp. fuliginosa, C. fuliginosa subsp. misandra (C.misandra R. Br.), C. stenantha var. taisetsuensis Akiyama (C. ktausipali Meinsh.).

**General distribution. Europe**: Norway, Sweden, Finland, Austria, Slovakia, Romania. **Asia**: Russian Far East. **North America**: Alaska, Canada (Nunavut, British Columbia), Greenland.

**Earlier reports from Greenland**: Hagen (1947, as ‘Ci. caricis’), Kukkonen (1963), Nannfeldt (1979).

**Comments** — Anthracoidea misandrae is a circumpolar–alpine species. It has been reported on ten sedges in section Aulocystis: Carex atrofusca, C. ferruginea Scop., C. firma Host, C. fuliginosa subsp. fuliginosa, C. fuliginosa subsp. misandra, C. luzulifolia W. Boott, C. petricosa Dewey, C. setosa Boott var. setosa (C. pachyrrhiza Franch.), C. stenantha var. taisetsuensis (C. ktausipali), and C. stenocarpa Turcz. ex V.I. Krecz.

Carex atrofusca and C. fuliginosa subsp. misandra are the principal hosts of this smut fungus. On Carex atrofusca (a circumpolar–alpine species), Anthracoidea misandrae is known from Fennoscandia, Canada (Nunavut), and Greenland (Savile 1952, as ‘*Ci. limosa var. minor*’; Kukkonen 1963; Nannfeldt 1979).

On Carex fuliginosa subsp. misandra (a circumpolar taxon), A. misandrae has been previously recorded from Fennoscandia, Alaska, and Canada (British Columbia, Nunavut) (Linder 1947, as ‘Ci. caricis’; Savile 1952, as ‘Cintractia limosa var. minor’; Kukkonen 1963; Nannfeldt 1979). There are three records of a smut fungus on this sedge from Svalbard (Spitsbergen) (Gjærum 1991). For the first time, it was mentioned by Lind (1928, as ‘Ci. caricis on Carex misandra’), but it is necessary his voucher specimen (if any) to be re-identified. Afterwards, it was reported by Hagen (1950b, as ‘Ci. caricis on Carex misandra’), but considering Hagen’s spore measurements, this record should be referred rather to Anthracoidea altera than to A. misandrae. A third specimen from Spitsbergen was published by Kukkonen (1963), as a paratype of A. misandrae, but later it was assigned to A. altera (Nannfeldt 1979). Carex fuliginosa subsp. misandra is reported here as a new host of A. misandrae for Greenland.

As noted, Anthracoidea altera is not found on Carex fuliginosa subsp. fuliginosa, while A. misandrae is known to infect both subspecies of C. fuliginosa. Carex fuliginosa subsp. fuliginosa is an alpine, Central and South European plant (Hultén and Fries 1986: 517; Egorova 1999) that is recorded as infected by A. misandrae from Austria, Slovakia, and Romania (Vánky 1985a; Zwetko and Blanz 2004).

Carex stenantha var. taisetsuensis is reported as infected by A. misandrae from the northern Kurile Islands (Paramushir — Govorova 1987, 1990; Denchev et al. 2013). There are no molecular data for this sedge. Because of this reason, we continue to treat it as a member of section Aulocystis and a host of A. misandrae.

The current status of the smut fungi on Carexferruginea, C. firma, C. luzulifolia, C. petricosa, C. setosa, and C. stenocarpa, reported as ‘A. misandrae’, will be discussed elsewhere.

The spore germination of A. misandrae was considered as belonging to Proceres-type (after Kukkonen 1963: 83 and Fig. 32; s. also Vánky 1979, 1994, 2011), but this observation was based on germination of an Anthracoidea specimen on Carex petricosa, and should be referred to another smut fungus.

#### 12(12) Anthracoidea nardinae (Kukkonen) Nannf., Symb. Bot. Upsal. 22(3): 29, 1979. ≡ Anthracoidea elynae var. nardinae Kukkonen, Ann. Bot. Soc. Zool.-Bot. Fenn. ‘Vanamo’ 34(3): 66, 1963. — Holotype on Carex nardina, Canada, Nunavut, Baffin Island, Frobisher Bay, 11 Aug 1959, leg. D.B.O. Savile et al. (TUR); isotypes in DAOM, H.

Fig. 13A–F

**Infection** local. **Sori** in some female flowers, around aborted nuts as subglobose or ovoid hard bodies, 1.0–2.0 mm long, initially covered by a thin, grayish peridium that later flakes away exposing a black spore mass, powdery on the surface. **Spores** medium-sized, flattened, in plane view suborbicular, slightly irregular, broadly elliptical, orbicular or ovate in outline, in plane view (16–)17–22(–23) × (15–)16–19.5(–20.5) (19.2 ± 0.9 × 17.7 ± 0.8) µm (n/_3_ = 300), in side view 10–14.5 µm thick, medium or dark reddish brown; wall slightly unevenly thickened, (1.0–)1.2–1.8(–2.2) µm thick, often with 1–3 internal swellings, light refractive areas and protuberances absent; smooth. In SEM spore wall smooth, often rugulose in the middle parts of the flattened sides. **Spore germination** of Anthracoidea-type (after Kukkonen 1963: 67), resulting in a two-celled basidium, 100–125 µm long, the apical cell 30–35 × 4.5–6.0 µm; producing 1–3 basidiospores on each cell; basidiospores subglobose to ellipsoidal or ovoid to obovoid, 7–15 × 4–8 μm.

**Figure 13.**
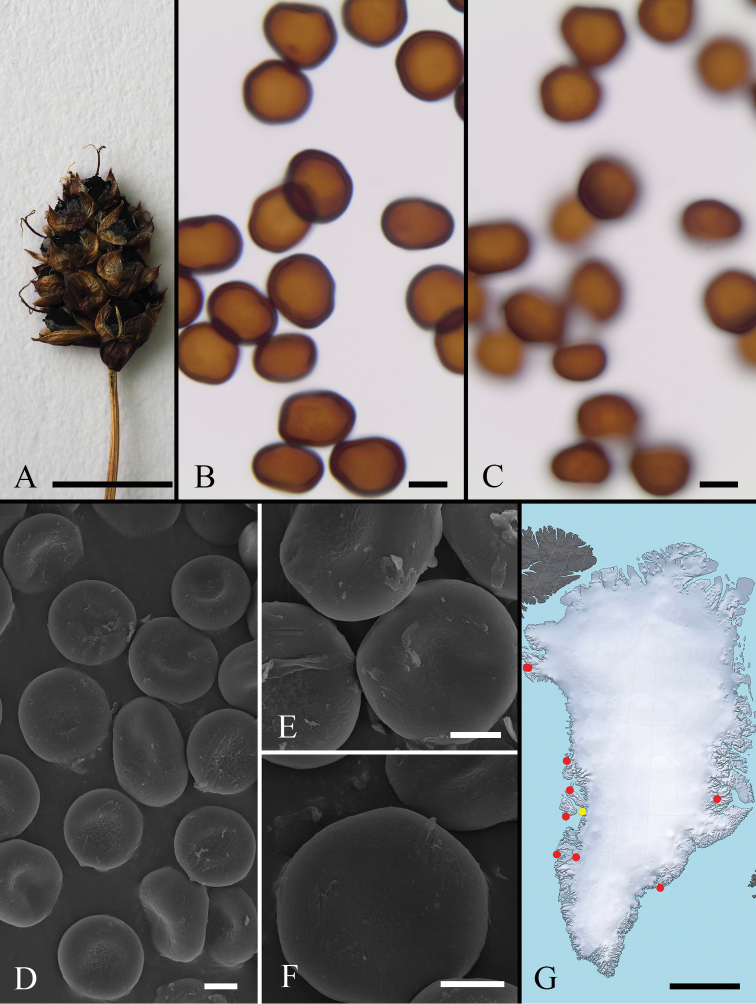
Anthracoidea nardinae on Carex nardina. **A** Habit (leg. Soejgaard, s.n., C-Greenland herb., s.n.) **B, C** spores in LM, in median and surface view, respectively (C-F-107989) **D–F** spores in SEM (**D, F** leg. M.P. Porsild, s.n., C-Greenland herb., s.n.; **E** leg. L. Koch, s.n., C-Greenland herb., s.n.) **G** distribution map (red circles – on Carex nardina; yellow circle – on C. nardina var. hepburnii). – Scale bars: 0.5 cm (**A**); 10 µm (**B, C**); 5 µm (**D–F**); 500 km (**G**).

**Hosts and distribution within the studied area** — On Cyperaceae: Carex sect. Nardinae (Tuck.) Mack.: Carex nardina s. lat. – North, West, and East Greenland; C. nardina subsp. hepburnii – West Greenland (Fig. 13G).

**Specimens examined or recorded.**

On **Carex nardina** (Hornem.) Fr., **s. lat.**:

**NG**, Wolstenholme Fjord (as ‘Wolstenholme Sound’), Umanaq, at ca 76°30–33'N, 7 Aug 1916, leg. L. Koch, s.n. (C-Greenland herb.!, s.n.); **ditto**, Thule, 1988, leg. S.A. Elborne, no. SAE-88.214-GR (C-F-107989!).

**WG**, Prøven Island, 72°23'N, 4 Sep 1934, leg. M.P. Porsild, s.n. (C-Greenland herb.!, s.n.).

**WG**, Nuussuaq Peninsula, Niaqornat Peninsula (as ‘Niakornak’) near Uummannaq Fjord, 70°48'N, 53°49'W, 7 Sep 1892, leg. E. Vanhöffen, no. 106(265) (C-Greenland herb.!, s.n.).

**WG**, Disko Island, near Qeqertarsuaq (as ‘Godhavn’), E of Arctic Station, 69°15'N, alt. 100 m, 28 Jul 1982, leg. J. Poelt & H. Ullrich, s.n. (GZU 000323443!).

**WG**, Kangerlussuaq, ‘loc. 3’, 66–67°N, 6 Aug 1946, leg. T.W. Böcher, The Botanical Expedition to West-Greenland 1946, s.n. (C-F-107988!).

**WG**, Sisimiut (as ‘Holstensborg’), 66°56'N, 5 Aug 1884, leg. E. Warming & Th. Holm, s.n. (n.v.; not found in C; Rostrup 1888, as ‘*U. caricis*’).

**EG**, Scoresby Sund, Harefjord, 70°51'N, 28°00'W, August 1957, leg. Soejgaard, s.n. (C-Greenland herb.!, s.n.).

**EG**, Tasiilaq Island, Kordlortok Tasiusak, 65°40'N, 37°31'W, August 1902, leg. C. Krusse, Expeditio Danica in Groenlandiam orientalem 1901–1902, s.n. (C-F-102519!, as ‘*U. caricis*’; Rostrup 1904, as ‘*U. caricis*’; dubl. in UPS, as ‘Anthracoidea elynae var. nardinae’, Kukkonen 1963).

On **Carex nardina** subsp. **hepburnii** (Boott) Á. Löve, D. Löve & B.M. Kapoor:

**WG**, Alluttoq Island (Arve-Prinsens Ejland), Ritenbenk’s Coalpit, 13 Jul 1871, leg. Th.M. Fries, s.n. (O!, s.n.; Hagen 1947, as ‘Ci. caricis’).

**Known hosts** — On Cyperaceae: Carex sect. Nardinae (syn. C. sect. Filifoliae): Carex elynoides Holm (C. filifolia var. miser L.H. Bailey), C. nardina subsp. nardina, C. nardina subsp. hepburnii.

**General distribution. North Europe**: Svalbard, Norway. **North America**: Canada, Greenland, U.S.A. (Wyoming, Colorado).

**Earlier reports from Greenland**: Rostrup (1888, 1904, as ‘*U. caricis*’), Clinton (1904, 1906, as ‘Ci. caricis’), Hagen (1947, as ‘Ci. caricis’), Fischer (1953, as ‘Ci. caricis’), Nannfeldt and Lindeberg (1957, as ‘Ci. caricis’), Kukkonen (1963, as ‘*A. elynae* var. *nardinae*’), Savile and Parmelee (1964, as ‘*A. elynae* var. *nardinae*’), Nannfeldt (1979), Vánky (2011).

**Comments** — Kukkonen (1963) described Anthracoidea elynae var. nardinae to accommodate a smut on Carex nardina. In its protologue, this sedge was designated as a single host. In a comment to Anthracoidea externa in the same treatment, however, Kukkonen assigned two specimens of a smut fungus on Carex elynoides also to A. elynae var. nardinae. Carex elynoides has in previous times been considered as belonging to Carex sect. Filifoliae (cfr Mastrogiuseppe 2002). For this reason, in the world monograph of Vánky (2011), the hosts of A. nardinae, Carex nardina and C. elynoides, are referred to different sections — Nardinae and Filifoliae, respectively. However, results of recent molecular studies (cfr Global Carex Group 2016) show that section Filifoliae should be merged with section Nardinae. That is why, in the current treatment, all hosts of A. nardinae are referred to Carex sect. Nardinae. The smut fungi on Carex nardina complex and C. elynoides deserve further study.

The taxonomic status of the taxa in the Carex nardina complex has been a subject of much debate. Some authors (e.g., Egorova 1966, 1999) accepted Carex nardina and C. hepburnii as distinct species, while other considered them as a single variable species (e.g., Chater 1980; Murray 2002; Aiken et al. 2007; Sawtell 2012; Saarela et al. 2017). In *Panarctic Flora* (Elven et al. 2018) and *Flora of Svalbard* (Alsos et al. 2018), however, two subspecies are recognized within Carex nardina: subsp. nardina, an amphi-Atlantic taxon, distributed in the mountains of Arctic Norway and Sweden, Iceland, (?) Greenland, and (?) Canada, and subsp. hepburnii, an amphi-Beringian–North American–amphi-Atlantic taxon, known from the Russian northeastern Arctic, Alaska, Canada, U.S.A. (the Cordilleras), Greenland, and Svalbard. Unfortunately, Greenland falls within the area with the highest uncertainty regarding the infraspecific delimitation of this sedge: ‘doubts about Greenland and Canada are about whether both subspecies are present’ (Elven et al. 2018). That is why, in the current treatment a broader circumscription for the Greenlandic specimens of Carex nardina is applied.

Carex elynoides is a Cordilleran species (from NW, SW, and SC U.S.A.) (Govaerts 2018).

Anthracoidea nardinae is a circumpolar–alpine species that is reported on Carex nardina subsp. nardina from Norway, Greenland, and (?) the eastern Canadian Arctic; on C. nardina subsp. hepburnii from Canada (British Columbia, Nunavut, Quebec), Greenland, and Svalbard (Spitsbergen) (Rostrup 1888, 1904, as ‘*U. caricis*’; Hagen 1947, as ‘Ci. caricis’; Linder 1947, as ‘Ci. caricis’; Kukkonen 1963, as ‘*A. elynae* var. *nardinae*’; Savile and Parmelee 1964; Nannfeldt 1979); and on C. elynoides from Wyoming and Colorado (Kukkonen 1963; Nannfeldt 1979).

#### 13(13) Anthracoidea paniceae Kukkonen, s. lat., Ann. Bot. Soc. Zool.-Bot. Fenn. ‘Vanamo’ 34(3): 76, 1963. — Holotype on Carex panicea, Finland, Ahvenanmaa [Åland], Kökar, Idö, 4 Aug 1947, leg. L.E. Kari (TUR); isotypes in Fungi Exsicc. Fenn., Fasc. 3, no. 129 (as ‘Cintractia caricis’).

**Hosts and distribution within the studied area** — On Cyperaceae: Carex (the Bicolores–Paniceae clade): Carex vaginata.

**Specimens recorded.**

On **Carex vaginata** Tausch:

**East Greenland** (see the comments on this species).

**Known hosts** (in its broader circumscription) — On Cyperaceae: Carex (the Bicolores–Paniceae clade): Carex aurea Nutt., C. bicolor All., C. livida (Wahlenb.) Willd., C. panicea L., C. vaginata var. vaginata, C. vaginatavar. petersii (C.A. Mey. ex F. Schmidt) Akiyama (C. falcata Turcz.), and hybrids.

**General distribution** (in its broader circumscription). **Europe**: Iceland, Faeroes, UK, Norway, Sweden, Finland, Denmark, Estonia, Latvia, Lithuania, Germany, Poland, Switzerland, Austria, Czech Republic, Slovakia, Romania, Ukraine, Spain, Italy, Russia. **Asia**: West Siberia, Russian Far East, China. **North America**: Canada, Greenland, U.S.A.

**Comments** — This smut fungus is reported from Greenland by Nannfeldt (1979: 30), but no voucher specimen is cited or is known to exist. In Greenland, the host plant, Carex vaginata, is only known from one locality in Clavering Island (74°05–06'N).

For description and illustrations of Anthracoidea paniceae, see Denchev and Minter (2011b), Vánky (2011), Denchev et al. (2013).

Carex panicea (a Eurasiatic species) and C. vaginata var. vaginata (a temperate-Eurasiatic taxon) are the principal hosts of Anthracoidea paniceae, but this smut fungus occurs also on other sedges in the C. vaginata complex as well as on C. bicolor and C. aurea belonging to the former section Bicolores (cfr Ball 2002b).

Anthracoidea paniceae, in its broad sense, is a circumboreal species. In North America, it is known from Alaska, Canada (Yukon, Northwest Territories, British Columbia, Alberta, Saskatchewan, Manitoba, Quebec, Labrador, and Newfoundland), north-central and northeastern U.S.A., and Greenland (Savile 1952, as ‘Ci. caricis’ or ‘Ci. limosa var. limosa’; Kukkonen 1963; Gremmen and Parmelee 1972; Nannfeldt 1979; Parmelee 1983, 1988).

#### 14(14) Anthracoidea pseudofoetidae L. Guo, Fung. Diversity 21: 84, 2006. — Holotype on Carex pseudofoetida, China, Xizang (Tibet Autonomous Region), Gégyai Xian, Alingshan, alt. 5200 m, 15 Aug 1976, Qinghai-Xizang expedition, no. 13486 (HMAS 130321); isotype H.U.V. 20091.

Fig. 14A–F

**Infection** local. **Sori** in some female flowers, around aborted nuts as subglobose, ovoid or broadly ellipsoidal hard bodies, 0.7–1.2 mm long, initially covered by a thick, dark brown peridium that later flakes away exposing a blackish brown spore mass, powdery on the surface. **Spores** very small-sized, irregularly rounded, subglobose, broadly ellipsoidal, ovoid or ellipsoidal, (9–)9.5–11.5(–12.5) × (8–)8.5–10.5(–11.5) (10.5 ± 0.6 × 9.6 ± 0.5) µm (n/_2_ = 200), medium reddish brown; wall unevenly thickened, 0.9–1.5 µm thick, with a few paler, rounded areas with thinner wall (0.5–0.9 µm thick), internal swellings, light refractive areas, and protuberances absent; minutely verruculose-echinulate, spore profile not affected. In SEM spore wall depressed on 3–6 places, ornaments up to 0.15 µm high, usually solitary and sparsely spaced, occasionally partly confluent, forming short rows or small groups. **Spore germination** unknown.

**Figure 14.**
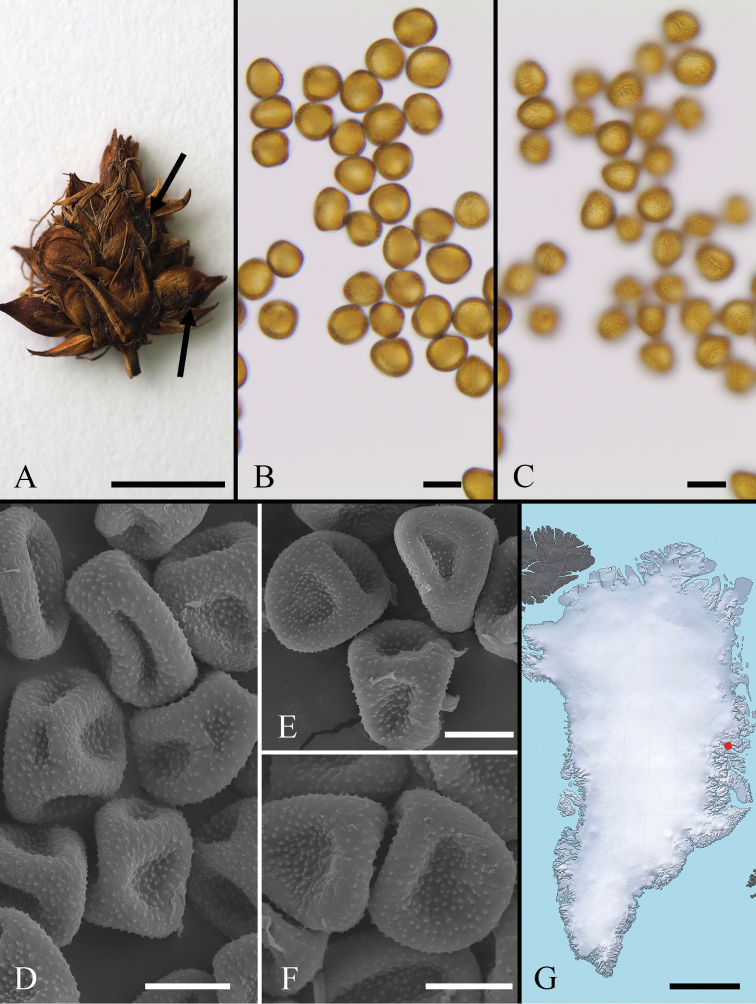
Anthracoidea pseudofoetidae on Carex maritima (T. Sørensen, no. 3085, C-Greenland herb., s.n.). **A** Habit (black arrows show sori) **B, C** spores in LM, in median and surface view, respectively **D–F** spores in SEM**G** distribution map. Scale bars: 0.5 cm (**A**); 10 µm (**B, C**); 5 µm (**D–F**); 500 km (**G**).

**Hosts and distribution within the studied area** — On Cyperaceae: Carex sect. Foetidae: Carex maritima – East Greenland (Fig. 14G).

**Specimens examined or recorded.**

On **Carex maritima** Gunnerus:

**EG**, Strindberg Land (as ‘Strindbergs Halvøya’), Nordfjord, 73°45'N, 12 Aug 1932, leg. T. Sørensen, The Three-year expedition to East Greenland 1931–1933 under the leadership of Dr. L. Koch, no. 3085 (C-Greenland herb.!, s.n., the host as ‘C. incurva Lightf.’); **ditto**, Broget Dal, 73°44–47'N, 24°37–59'W, 31 Jul 1994, leg. R. David & S. David, s.n. (C-Greenland herb.!, s.n.).

**Known hosts** — On Cyperaceae: Carex sect. Foetidae (Tuck. ex L.H. Bailey) Kük.: Carex pseudofoetida Kük., C. maritima.

**General distribution. Asia**: China (Xizang). **North America**: Greenland.

**Comments** — Anthracoidea pseudofoetidae is reported here for the first time from Greenland and North America and Carex maritima is a new host for this smut fungus.

Anthracoidea pseudofoetidae was previously known only on C. pseudofoetida, from the type collection (Guo 2006). Both sedges are members of Carex sect. Foetidae but C. pseudofoetida is a Central Asiatic species, with distribution restricted to mountains in Central Asia, while C. maritima is a widespread species, with a bipolar distribution (in South America from Ecuador to Argentina), being a circumpolar–alpine species in the Northern Hemisphere — distributed there in Alaska, Canada, Greenland, and northern Eurasia, as well as in alpine regions of Europe and Central Asia (Hultén and Fries 1986: 436; Egorova 1999; Reznicek 2002; Villaverde et al. 2015; Elven et al. 2018; Govaerts 2018).

The two localities recorded here significantly extend the geographic range of A. pseudofoetidae and reveal an unexpected disjunct distribution. Of course, this disjunction may reflect insufficient sampling — many parasitic fungi in the Arctic are superficially known and it may be expected that additional localities of A. pseudofoetidae will be found — but considering that, at least, one of its hosts is a widespread species, it seems that this smut fungus is a very rare species.

The type locality of A. pseudofoetidae is in Tibet, at 5200 m; while the Greenlandic localities are in the High Arctic, at ca 73°44–47'N. This smut fungus is an Arctic–alpine species with restricted distribution and perfect adaptation to extreme conditions: low temperatures and a short growing season of the host plants.

Anthracoidea pseudofoetidae possesses a suite of distinctive features that includes: (i) sori covered by a thick, dark brown peridium; (ii) small spore sizes (the smallest spores in the genus), (iii) a characteristic spore wall, depressed on 3–6 places where the wall is paler and thinner, and (iv) absence of internal swellings, light refractive areas, and protuberances.

#### 15(15) Anthracoidea rupestris Kukkonen, s. str., Ann. Bot. Soc. Zool.-Bot. Fenn. ‘Vanamo’ 34(3): 47, 1963. — Holotype on Carex rupestris, Finland, Enontekiön Lappi, Kilpisjärvi, on the S slope of Mt. Saana, 19 Jul 1961, leg. I. Kukkonen, no. 730 (TUR); isotypes in H.

Fig. 15A–F

**Infection** local. **Sori** in some female flowers, around aborted nuts as subglobose or ovoid hard bodies, 1.2–2.0 mm long, initially covered by a thin, grayish peridium that later flakes away exposing a black spore mass, powdery on the surface. **Spores** medium-sized, flattened, in plane view usually irregularly rounded to angular, sometimes broadly elliptical or suborbicular in outline, sometimes with a protuberance, (15.5–)17–23(–26) × (14–)15–20.5(–22) (20.3 ± 1.5 × 17.9 ± 1.3) µm (n/_3_ = 300), in side view 10.5–14.5 µm thick, dark reddish brown; wall unevenly thickened, 1.0–3.0(–3.8) µm thick, thickest at the angles and protuberances, usually with 1–4, well visible internal swellings, light refractive areas often present; minutely verruculose, warts up to 0.2(–0.3) µm high, spore profile not affected or very slightly affected. In SEM warts sometimes partly confluent, forming short rows or small groups. **Spore germination** of Anthracoidea-type (after Kukkonen 1963: 47, Fig. 23), resulting in a two-celled basidium, 100–150 μm long, the apical cell 30–60 μm long and 3.5–6 μm thick; producing several basidiospores on each cell; basidiospores ellipsoidal, ovoid or obovoid, 5–12 × 3–6 μm.

**Figure 15.**
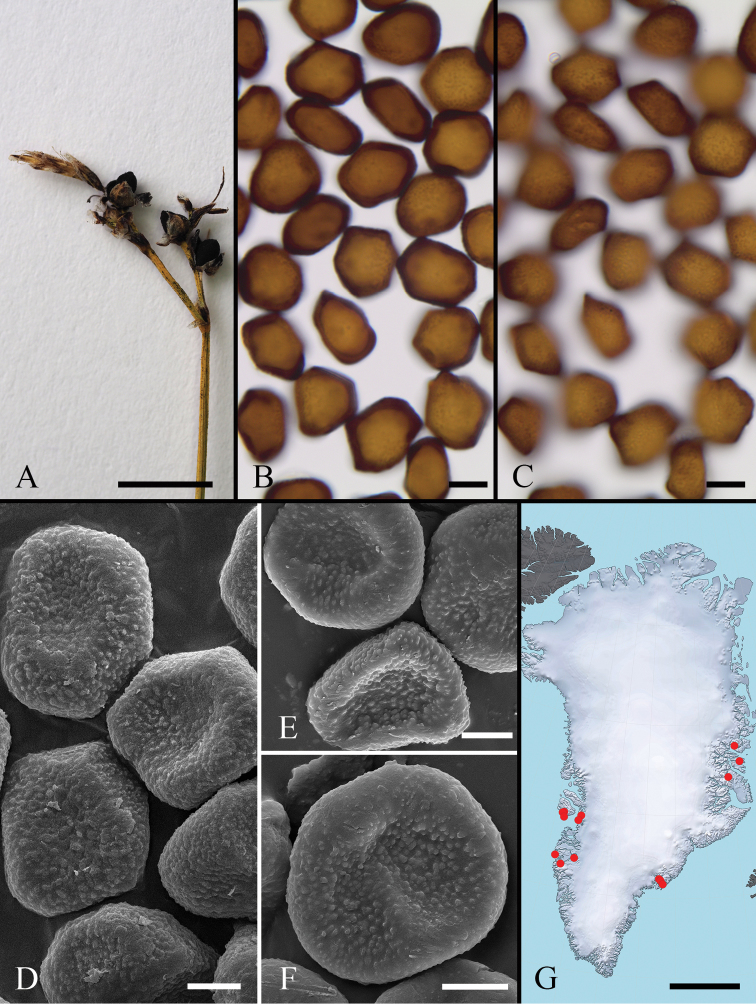
Anthracoidea rupestris on Carex rupestris. **A** Habit (C-F-111312) **B, C** spores in LM, in median and surface view, respectively (C-F-111312) **D–F** spores in SEM (**D** leg. Å. Jensen, s.n., C-Greenland herb., s.n.; **E, F** leg. T. Sørensen, s.n., C-Greenland herb., s.n.) **G** distribution map. Scale bars: 0.5 cm (**A**); 10 µm (**B, C**); 5 µm (**D–F**); 500 km (**G**).

**Hosts and distribution within the studied area** — On Cyperaceae: Carex sect. Rupestres (Tuck.) Mack.: Carex rupestris subsp. rupestris – West and East Greenland (Fig. 15G).

**Specimens examined or recorded.**

On **Carex rupestris** All. subsp. **rupestris**:

**WG**, Disko Island, Diskofjord, between Kangerdluarssuk and Eqalunguit, 69°32'N, 53°41–44'W, 23 Jul 1980, leg. B. Fredskild et al., no. 133 (C-Greenland herb.!, s.n.); **ditto**, prope pagum Diskofjord, 69°29'N, 53°55'W, 4 Aug 1982, leg. J. Poelt & H. Ullrich, s.n. (C-F-102536!, Vánky, Ustilaginales Exsiccata, no. 388; Vánky 1983, 2011); **ditto**, Qeqertarsuaq (as ‘Godhavn’), 69°15'N, 53°32'W, 9 Aug 1912, leg. Th. Porsild, s.n. (C-Greenland herb.!, s.n.); **ditto**, near Arctic Station, 69°15'N, 7 Aug 1936, leg. Å. Jensen, s.n. (C-Greenland herb.!, s.n.); **ditto**, Qeqertarsuaq, 69°14'N, 27 Jul 1886, leg. K. Rosenvinge, s.n. (C-F-102514!, as ‘*U. caricis*’; Rostrup 1888, as ‘*U. caricis*’); **ditto**, NE of Qeqertarsuaq, 29 Jul 1982, leg. J. Poelt & H. Ullrich, s.n. (GZU 000323436!); **ditto**, near Disko Bugt, 69°14'N, 12 Aug 1898, leg. M. Pedersen, no. 3975 (C-F-102520!, as ‘*U. caricis*’).

**WG**, Pâkitsoq, Berggrens Havn, 69°31'N, 50°46'W, alt. 20 m, 26 Jul 1981, leg. J. Feilberg, no. 2794 (C-Greenland herb.!, s.n.).

**WG**, Ilulissat (as ‘Jakobshavn’), 69°13'N, 51°06'W, 19 Jul 1892, leg. G.H. Sørensen, s.n. (C-Greenland herb.!, s.n.).

**WG**, Kangerlussuaq, slopes SW of Lake Ferguson, 66°57'36"N, 50°41'24"W, 29 Aug 2018, leg. H. Knudsen, no. HK 18.409 (C-F-111312); **ditto**, north slope towards Lake Ferguson, 66°57'26"N, 50°41'40"W, alt. 434 m, 29 Aug 2018, leg. S.A. Elborne, no. SAE-2018.423-GR (C-F-112970).

**WG**, near Sisimiut (as ‘Holsteinsborg’), 66°56'N, 1 Aug 1886, leg. T. Holm, s.n. (n.v.; not found in C; Rostrup 1888, as ‘*U. caricis*’); **ditto**, Skarnsak Island near Sisimiut, sine dat., leg. T. Holm, s.n. (n.v.; Schröter 1888, as ‘*U. caricis*’); **ditto**, Sisimiut, 31 Jul 1947, leg. T. Sørensen, s.n. (C-Greenland herb.!, s.n.).

**WG**, Inugsugtussoq Island, Tunulliarfik, E part, alt. 800 m, ca 66°28'N, 52°35'W, 13 Aug 1958, leg. Beschel, no. 8499 (C-Greenland herb.!, s.n.).

**EG**, Hoelsbo (as ‘Hoelsbu’), on the north side of Moskusoksefjord, ca 73°42.2'N, 23°26.3'W, 29 Jul 1933, leg. A. Hagen, the Norwegian Expedition to NE Greenland 1933, s.n. (O!, s.n.; Hagen 1947, as ‘Ci. caricis’).

**EG**, Geographical Society Island, 5 km W of Husbukta (ca 72°49.7'N, 22°52.5'W), 16 Aug 1930, leg. J. Vaage, s.n. (O!, s.n.; Hagen 1947, as ‘Ci. caricis’); **ditto**, 15 km W of Husbukta, 17 Aug 1930, leg. P.F. Scholander, s.n. (O!, s.n.; Hagen 1947, as ‘Ci. caricis’).

**EG**, Alpefjord, Stauning Alper, 28 Jul 1933, leg. A. Hagen, the Norwegian Expedition to NE Greenland 1933, s.n. (O!, s.n.; Hagen 1947, as ‘Ci. caricis’; dupl. DAOM 25 889, Kukkonen 1963).

**EG**, Tasiilaq, Sermilik, Siarqigteq, 66°12'N, 37°28'W, 25 Jul 1979, leg. B. Rode et al., no. 106 (C-Greenland herb.!, s.n.).

**EG**, Tasiilaq, Qingertivaq, 66°06'N, 37°13'W, 18 Jul 1969, leg. O. Hamann & L. Kliim-Nielsen, no. 69-1496 (C-Greenland herb.!, s.n.).

**EG**, Tasiilaq, Ilivtiartik, Torssukatak (Tûnok), 65°53'N, 36°53'W, 26 Jul 1969, leg. L. Kliim-Nielsen, no. 69-1808 (C-Greenland herb.!, s.n.).

**Known hosts** (in the narrow circumscription of the species) — On Cyperaceae: Carex sect. Rupestres: Carex rupestris subsp. rupestris.

**General distribution** (in the narrow circumscription of the species). **Europe**: Norway, Sweden, Finland, Denmark, France, Switzerland, Austria, Romania, Spain. **Asia**: Mongolia (Altay Mts), Russian Far East. **North America**: Canada, Greenland.

**Earlier reports from Greenland**: Rostrup (1888, as ‘*U. caricis*’), Schröter (1888, as ‘*U. caricis*’), Clinton (1904, 1906, as ‘Ci. caricis’), Lind (1934, as ‘Ci. caricis’), Hagen (1947, as ‘Ci. caricis’), Fischer (1953, as ‘Ci. caricis’), Kukkonen (1963), Nannfeldt (1979), Vánky (1983, 2011).

**Comments** — In the current treatment, Anthracoidea rupestris is considered in its strict sense, i.e., as distributed only on Carex rupestris, although a smut fungus under this name is reported from Greenland also on C. glacialis Mack. (Kukkonen 1963; Nannfeldt 1979). The taxonomic status of the species on C. glacialis will be discussed elsewhere, but on the basis of specimens studied by us, it may be noted that this species is distributed in West Greenland at least at 66° – 70°35'N, and in East Greenland at least at 66° – 74°28'N.

Carex rupestris is a circumpolar–alpine species, distributed in Eurasia and North America, including some mountain ranges in Central and South Europe, Central Asia, and western U.S.A. (Hultén and Fries 1986: 427; Egorova 1999; Elven et al. 2018). In Europe, Anthracoidea rupestris on Carex rupestris is known from North Europe, the Alps, the Carpathians, and the Pyrenees (Kukkonen 1963; Nannfeldt 1979; Vánky 1985a; Zogg 1986; Almaraz 2002; Zwetko and Blanz 2004); in Asia — from the Mongolian Altay Mts (Braun 1999) and the Russian Far East (Govorova 1990). In Canada, A. rupestris on Carex rupestris is reported from Nunavut, British Columbia, Manitoba, Quebec, Labrador, and Newfoundland (Linder 1947, as ‘Ci. caricis’; Savile 1952, as ‘Ci. caricis var. caricis’; Kukkonen 1963; Savile and Parmelee 1964; Parmelee 1969, 1983, 1988). According to Savile and Parmelee (1964) and Parmelee (1988), this smut fungus is widespread in Arctic and Subarctic Canada, with a northernmost locality on Axel Heiberg Island, at 79°25'N. Anthracoidea rupestris is a circumpolar–alpine species, like its host plant, and it is a good example of a smut fungus that is coextensive with its host.

#### 16(16) Anthracoidea scirpi (J.G. Kühn) Kukkonen, Ann. Bot. Soc. Zool.-Bot. Fenn. ‘Vanamo’ 34(3): 69, 1963. ≡ Ustilago urceolarum f. scirpi J.G. Kühn, in Rabenhorst, Fungi Eur. Exsicc., no. 1698, 1873; Hedwigia 12: 150, 1873. ≡ Cintractia scirpi (J.G. Kühn) Schellenb., Kryptogamenfl. Schweiz 3: 77, 1911. — Type on Trichophorum cespitosum, Germany, Harz Mts, Mt. Brocken, ‘Brockenfeld’, near ‘Brockenkegel’, 6 Sep 1871, leg. J.G. Kühn; isotypes in Rabenhorst, Fungi Europ. Exsicc., no. 1698.

Fig. 16A–F

**Infection** local. **Sori** in some female flowers, around aborted nuts as globose or subglobose hard bodies, 0.8–1.5 mm long, initially covered by a thin, grayish peridium that later flakes away exposing a black spore mass, powdery on the surface. **Spores** medium-sized, flattened, in plane view suborbicular, slightly irregular, broadly elliptical, orbicular or ovate in outline, in plane view (17–)18–22(–23) × (15.5–)16.5–20(–21) (19.8 ± 1.0 × 18.3 ± 0.9) µm (n/_3_ = 300), in side view 11–14 µm thick, medium or dark reddish brown; wall slightly unevenly thickened, (1.0–)1.2–2.0(–2.2) µm thick, internal swellings, light refractive areas, and protuberances absent; smooth. In SEM spore wall rugulose or punctate; ornamentation up to 0.10 µm high. **Spore germination** of Anthracoidea-type (after Kukkonen 1963: 69, Fig. 29), resulting in a two-celled basidium, 60–120 μm long, the apical cell 24–40 × 3.5–7 μm; basidiospores lacrymiform (with a sharp end towards the sterigma, rounded at the apex), ovoid or obovoid, 14–27(–29) × 3.5–7(–9) μm.

**Figure 16.**
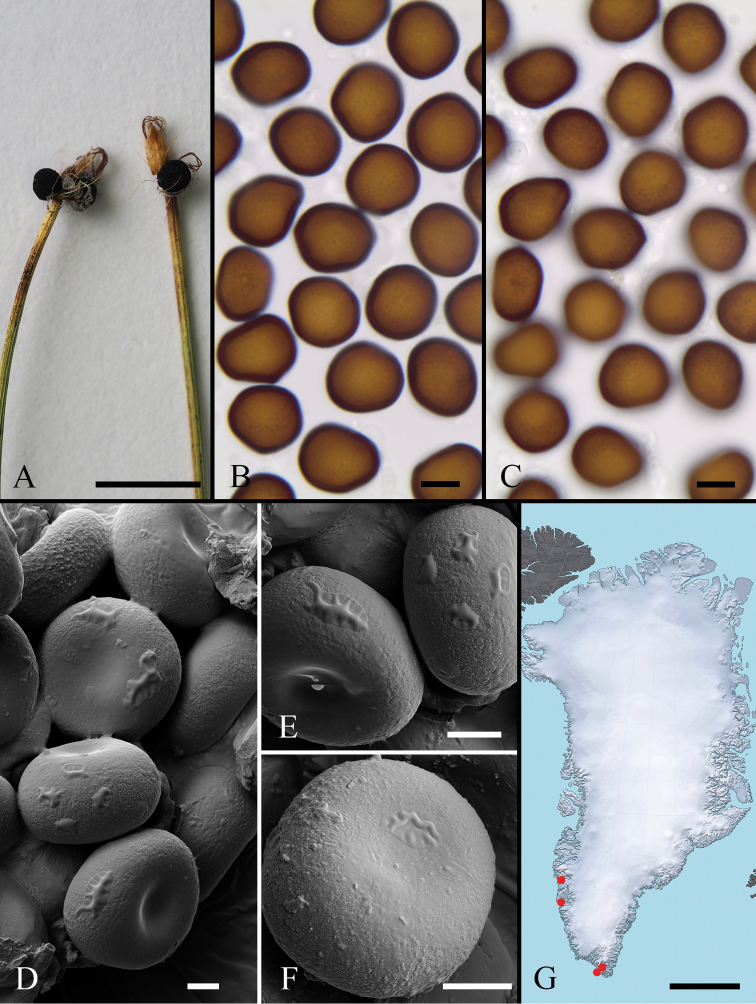
Anthracoidea scirpi on Trichophorum cespitosum (C-F-111313). **A** Habit **B, C** spores in LM, in median and surface view, respectively **D–F** spores in SEM**G** distribution map. Scale bars: 0.5 cm (**A**); 10 µm (**B, C**); 5 µm (**D–F**); 500 km (**G**).

**Hosts and distribution within the studied area** — On Cyperaceae: Trichophorum cespitosum subsp. cespitosum – West Greenland (Fig. 16G).

**Specimens examined or recorded.**

On **Trichophorum cespitosum** (L.) Hartm. subsp. **cespitosum**:

**WG**, Tupertalik, 65°29'N, 51°58'W, alt. 200–250 m, 4 Aug 1977, leg. A. Alstrup, no. 77960 (C-Greenland herb.!, s.n.).

**WG**, Kobbefjord, Nuuk Basic, 64°08'N, 51°23'W, 24 Aug 2018, leg. H. Knudsen, nos HK 18.312, HK 18.314 (C-F-111313!, C-F-111314!).

**WG**, Tasermiutsiak near Tasermiut, 60°27'N, 1889, leg. N. Hartz, s.n. (C-F-102511!, as ‘*U. caricis*’).

**WG**, Tasermiut, 60°05'N, August 1829, leg. J. Vahl, s.n. (C-F-102510!, as ‘*U. caricis*’; Rostrup 1888, as ‘*U. caricis*’).

**Known hosts** — On Cyperaceae: Trichophorum cespitosum subsp. cespitosum (Scirpus cespitosus L., Baeothryon cespitosum (L.) A. Dietr., Trichophorum cespitosum subsp. austriacum (Palla) Hegi), T. cespitosum subsp. germanicum (Palla) Hegi (Scirpus cespitosus subsp. germanicus (Palla) Brodd.), T. cespitosum nothosubsp. foersteri Swan (Trichophorum × foersteri (Swan) D.A.Simpson), T. pumilum (Vahl) Schinz & Thell. (Scirpus pumilus Vahl).

**General distribution. Europe**: Iceland, UK, Norway, Sweden, Finland, Germany, Switzerland, northwestern Russia. **Asia**: Russian Far East. **North America**: Alaska, Canada, Greenland, northeastern U.S.A.

**Earlier reports from Greenland**: Rostrup (1888, as ‘*U. caricis*’), Clinton (1902, 1904, 1906, as ‘Ci. caricis’), Lind (1934, as ‘Ci. scirpi’).

**Comments** — Trichophorum cespitosum subsp. cespitosum is a circumboreal–polar taxon, T. cespitosum subsp. germanicum is an Atlantic European taxon (Elven et al. 2018; Govaerts 2018), and T. pumilum is a circumboreal species (Egorova 1976; Govaerts 2018).

Anthracoidea scirpi is a circumboreal species. In Eurasia, it is known from North Europe and some mountains in Central Europe (Liro 1938; Jørstad 1963; Kukkonen 1963, 1964; Jørstad and Gjærum 1966; Gjærum 1972; Karatygin et al. 1999; Zogg 1986; Scholz and Scholz 1988, 2000, 2004; Scholler et al. 2003; Helgi Hallgrímsson and Guðríður Gyða Eyjólfsdóttir 2004; Smith 2010; Klenke and Scholler 2015), as well as from the Russian Far East (Govorova 1990). This smut fungus is recorded also on T. cespitosum nothosubsp. foersteri (a hybrid between both subspecies of T. cespitosum), from the Outer Hebrides (Smith 2010). From North America, A. scirpi has been reported from Alaska, Canada (Northwest Territories, Quebec, Newfoundland, and Nova Scotia) (Savile 1952, as ‘Ci. scirpi’; Kukkonen 1963; Parmelee 1983, 1988), and U.S.A. (Michigan — Fischer 1953, as ‘Ci. scirpi’), and from a single locality in Greenland (Rostrup 1888).

The specific epithet *scirpi* reflects the former status of the type host, as a member of the genus Scirpus; in fact, A. scirpi does not infect species of Scirpus in its modern circumscription.

#### 17(17) Anthracoidea scirpoideae Kukkonen, Ann. Bot. Soc. Zool.-Bot. Fenn. ‘Vanamo’ 34(3): 78, 1963. — Holotype on Carex scirpoidea s. lat., Canada, British Columbia, Mile 397 Alaska Highway, McDonald Creek near Rocky Mountain Lodge, gravelly bank of the creek, 24 Jul 1960, leg. I. Kukkonen (no. 515) & J.A. Calder (TUR); isotypes in DAOM, H.

Fig. 17A–F

**Infection** local. **Sori** in some female flowers, around aborted nuts as subglobose hard bodies, ca. 1 mm long, initially covered by a thin, grayish peridium that later flakes away exposing a black spore mass, powdery on the surface. **Spores** medium- to large-sized, flattened, in plane view suborbicular, broadly elliptical, slightly irregularly rounded or ovate in outline, in plane view (18–)19–26(–27) × (16.5–)17.5–22(–23.5) (22.1 ± 1.7 × 20.0 ± 1.2) µm (n/_1_ = 100), in side view 13–15.5 µm thick, often with a hyaline sheath on the flattened sides, medium or dark reddish brown; wall evenly or slightly unevenly thickened, 1.3–2.4(–2.8) µm thick, often with 1–3(–4) internal swellings, light refractive areas and protuberances absent; minutely verruculose, warts up to 0.2(–0.3) µm high, spore profile not affected, sometimes very slightly affected. In SEM warts usually isolated, sometimes partly confluent, forming short rows or small groups, punctate between the warts. **Spore germination** of Proceres-type (after Kukkonen 1963: 78, Fig. 31), resulting in a two-celled basidium, 225–300 μm long, the apical cell 65–80 × 4–6 µm; producing cylindrical basidiospores, straight or slightly curved, 28–64(–72) × 3.4–6.4 μm.

**Figure 17.**
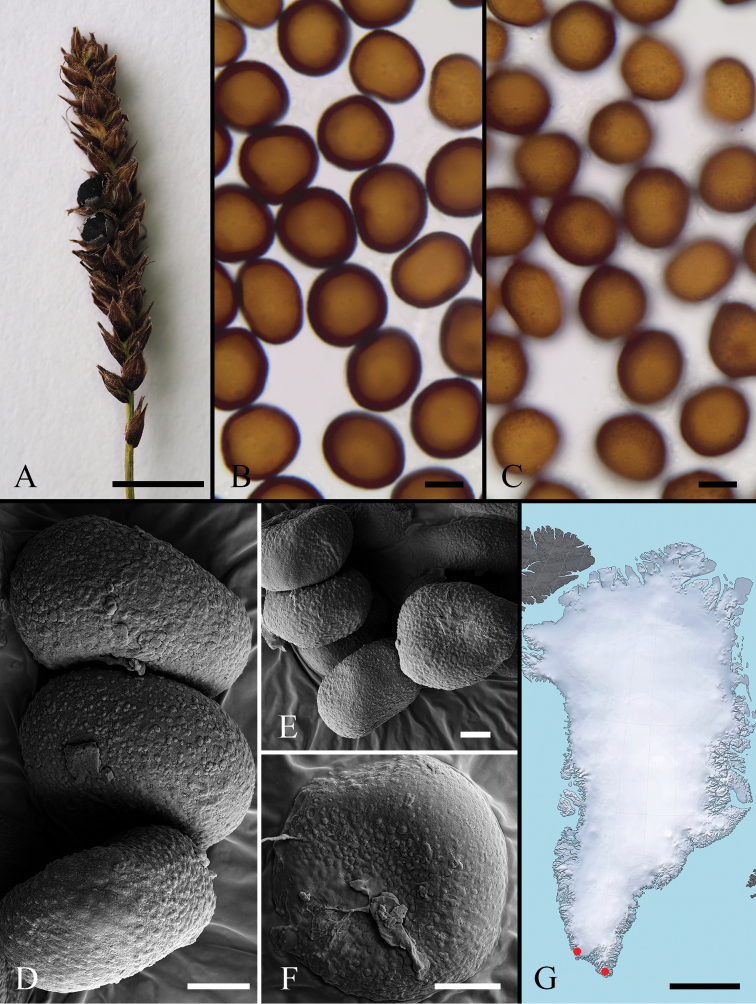
Anthracoidea scirpoideae on Carex scirpoidea (C-F-111315). **A** Habit **B, C** spores in LM, in median and surface view, respectively **D–F** spores in SEM**G** distribution map. Scale bars: 0.5 cm (**A**); 10 µm (**B, C**); 5 µm (**D–F**); 500 km (**G**).

**Hosts and distribution within the studied area** — On Cyperaceae: Carex sect. Scirpinae (Tuck.) Kük.: Carex scirpoidea subsp. scirpoidea – West Greenland (Fig. 17G).

**Specimens examined or recorded.**

On **Carex scirpoidea** Michx. subsp. **scirpoidea**:

**WG**, bottom of Laksefjord, 61°15'N, 48°04'48"W, 21 Aug 2018, leg. H. Knudsen, no. HK 18.294 (C-F-111315!).

**WG**, Ujaragsarsuk (as ‘Ujarsarksoit’) in Prins Christians Sund, 60°10'N, July 1829, leg. J. Vahl, s.n. (C-F-102521!, as ‘*U. caricis*’; Rostrup 1888, as ‘*U. caricis*’).

**Known hosts** — On Cyperaceae: Carex sect. Scirpinae: Carex scirpoidea subsp. scirpoidea, C. scirpoidea subsp. convoluta (Kük.) Dunlop, C. scirpoidea subsp. pseudoscirpoidea (Rydb.) Dunlop, C. scirpoidea subsp. stenochlaena (Holm) Á. Löve & D. Löve.

**General distribution. Asia**: Russia (Far East). **North America**: Alaska, Canada, Greenland, northwestern U.S.A.

**Comments** — Anthracoidea scirpoideae infects only one sedge, Carex scirpoidea, belonging to Carex sect. Scirpinae — a section with three species, distributed primarily in North America (Dunlop 2002). Four subspecies are recognized in the C. scirpoidea complex: (i) subsp. scirpoidea is the most widely ranging taxon in section Scirpinae, distributed from East Siberia, Russian Far East, Alaska, Yukon, and British Columbia, across northern North America, east to Newfoundland, northern New England, and Greenland, and in the mountains in western U.S.A., as well as with a disjunct population in Norway (an Asian (northeastern)–amphi-Beringian–North American (northern)–amphi-Atlantic (western) taxon; Elven et al. 2018); (ii) subsp. convoluta occurs only along the shores of Lake Huron; (iii) subsp. stenochlaena is distributed in mountains from Alaska and Yukon to northwestern U.S.A.; and (iii) subsp. pseudoscirpoidea that is distributed at higher altitudes (3300–3900 m) in the mountains in southern British Columbia and western U.S.A. (Dunlop and Crow 1999).

Anthracoidea scirpoideae is an amphi-Beringian–North American (northern)–Cordilleran species, reported from Russian Far East, Alaska, Yukon, Northwest Territories, Nunavut, British Columbia, Alberta, Manitoba, Ontario, Quebec, Newfoundland (Linder 1947, as ‘Ci. caricis’; Savile 1952, as ‘Ci. limosa var. limosa’; Kukkonen 1963; Gremmen and Parmelee 1972; Parmelee 1983, 1988; Govorova 1990), and northwestern U.S.A. (Kukkonen 1963; on C. scirpoideae subsp. pseudoscirpoidea). Of particular interest is the information about this smut fungus in the monographic treatment of Carex section Scirpinae by Dunlop (1990: 28): ‘Smut infected plants were common throughout the range of Carex scirpoidea (all subspecies), while populations of C. curatorum were characteristically free from any smuts. Additionally, specimens of C. gigas and C. scabriuscula were not infected by smuts…’ (see also op.c., table 5). From this source, we have reliable information that Anthracoidea scirpoideae infects all sedges in the C. scirpoidea complex while C. curatorum Stacey and C. scabriuscula Mack. (incl. C. gigas (Holm) Mack.) are resistant. It seems that, especially on C. scirpoidea subsp. scirpoidea, the fungus is a widely distributed species in Canada (see also Parmelee 1988).

#### 18(18) Anthracoidea turfosa (Syd.) Kukkonen, Ann. Bot. Soc. Zool.-Bot. Fenn. ‘Vanamo’ 34(3): 24, 1963. ≡ Cintractia turfosa Syd., Ann. Mycol. 22: 289, 1924. — Type on Carex dioica, Norway, Finnmark, Alta Kåfjord, July 1895, leg. G. Lagerheim; isotypes in Sydow, Ustilag., no. 75 (as ‘Cintractia caricis’).

Fig. 18A–C

**Infection** local. **Sori** in some female flowers, around aborted nuts as ovoid to broadly ellipsoidal hard bodies, 1.2–2 mm long, initially covered by a thin, grayish peridium that later flakes away exposing a black spore mass, powdery on the surface. **Spores** medium- to large-sized, flattened, in plane view irregularly rounded, broadly elliptical, subpolygonal or suborbicular in outline, sometimes slightly irregularly rounded, in plane view (17.5–)19–26.5(–28) × (16–)17–22.5(–23.5) (22.6 ± 1.8 × 20.1 ± 1.4) µm (n/_1_ = 100), in side view 11.5–14.5 µm thick, dark reddish brown; wall slightly unevenly thickened, 1.2–2.3(–2.6) µm thick, sometimes with 1(–3) internal swellings (hard to observe because of the dark-colored spores), light refractive areas and protuberances absent; minutely verruculose, spore profile not affected. **Spore germination** (after Lehtola 1940) of Proceres-type.

**Figure 18.**
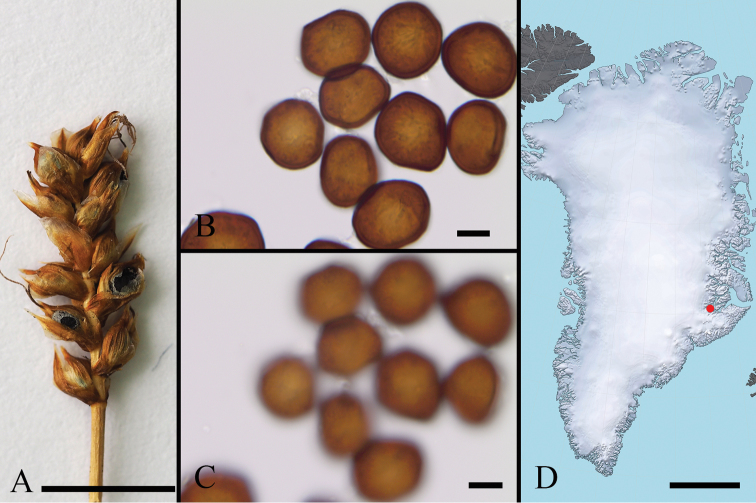
Anthracoidea turfosa on Carex parallela subsp. parallela (S. Lægaard, no. 138, C-Greenland herb., s.n.). **A** Habit **B, C** spores in LM, in median and surface view, respectively **D** distribution map. Scale bars: 0.5 cm (**A**); 10 µm (**B, C**); 500 km (**D**).

**Hosts and distribution within the studied area** — On Cyperaceae: Carex sect. Physoglochin Dumort.: Carex parallela subsp. parallela – East Greenland (Fig. 18D).

**Specimens examined or recorded.**

On **Carex parallela** (Laest.) Sommerf. subsp. **parallela**:

**EG**, Scoresby Sund, Gåseland, Faxe Sø, 70°15'N, 29°W, alt. 325 m, 16 Jul 1958, leg. S. Lægaard, no. 138 (C-Greenland herb.!, s.n.).

**Known hosts** — On Cyperaceae: Carex sect. Physoglochin: Carex davalliana Sm., C. dioica L., C. gynocrates Wormsk., C. parallela subsp. parallela, C. parallela subsp. redowskiana (C.A. Mey.) T.V. Egorova, and hybrids (C. dioica × C. parallela); Carex sect. Stellulatae: Carex exilis Dewey. On intersectional hybrids: Carex brunnescens × C. dioica, C. canescens × C. dioica, C. dioica × C. heleonastes, C. dioica × C. lachenalii, C. dioica × maritima, C. lachenalii × C. parallela.

On Carex sect. Glareosae: Carex heleonastes L. f. (as an accidental host).

**General distribution. North Europe**: Iceland, UK, Norway, Sweden, Finland, northwestern and Actic Russia, Latvia, Lithuania. **North America**: Canada, Greenland.

**Comments** — Anthracoidea turfosa is an amphi-Atlantic–European (northern) species, distributed in the eastern boreal part of North America, Greenland, and the northern temperate and boreal parts of Europe. In Canada, this smut fungus is known on Carex gynocrates from Quebec (Savile 1952, as ‘*Ci. limosa* var. *limosa*’), and on Carex exilis from Quebec, Labrador, and Nova Scotia (Savile 1952, as ‘*Ci. pratensis*’; Nannfeldt 1977; Parmelee 1983). Anthracoidea turfosa and A. karii (a species with smaller spores) have common hosts belonging to sections Physoglochin (including hybrids), Stellulatae, and Glareosae, or to intersectional hybrids. For this reason, some published records (especially, some from North America) need re-examination (see also Nannfeldt 1977: 368–372).

Anthracoidea turfosa is reported here for the first time from Greenland.

#### 19(19) Anthracoidea verrucosa (Savile) Nannf., Bot. Not. 130: 372, 1977. ≡ Cintractia carpophila var. verrucosa Savile, Canad. J. Bot. 30: 420, 1952. — Type on Carex ebenea (as ‘*C. festivella*’), U.S.A., Wyoming, Medicine Bow Mts, Lake Marie, 21 Aug 1941, leg. W.G. Solheim, no. 2009; isotypes in W.G. Solheim, Mycoflora Saximontanensis Exsiccata, no. 439 (as ‘Cintractia caricis’).

Fig. 19A–F

**Infection** local. **Sori** in some female flowers, around aborted nuts as subglobose to ovoid hard bodies, 0.5–1 mm long, initially covered by a thin, grayish peridium that later flakes away exposing a black spore mass, powdery on the surface. **Spores** small-sized, flattened, in plane view suborbicular, orbicular, broadly elliptical or ovate in outline, in plane view (14.5–)15.5–19(–20) × (13.5–)14.5–17(–18) (17.0 ± 0.9 × 15.6 ± 0.8) µm (n/_3_ = 300), in side view 9–12.5 µm thick, medium reddish brown; wall evenly or slightly unevenly thickened, 0.7–1.4 µm thick, often with 1–3(–4) weak internal swellings, light refractive areas and protuberances absent; minutely verruculose, warts up to 0.3 µm high, spore profile slightly affected. In SEM warts sometimes partly confluent, forming short rows or small groups, punctate between the warts. **Spore germination** unknown.

**Figure 19.**
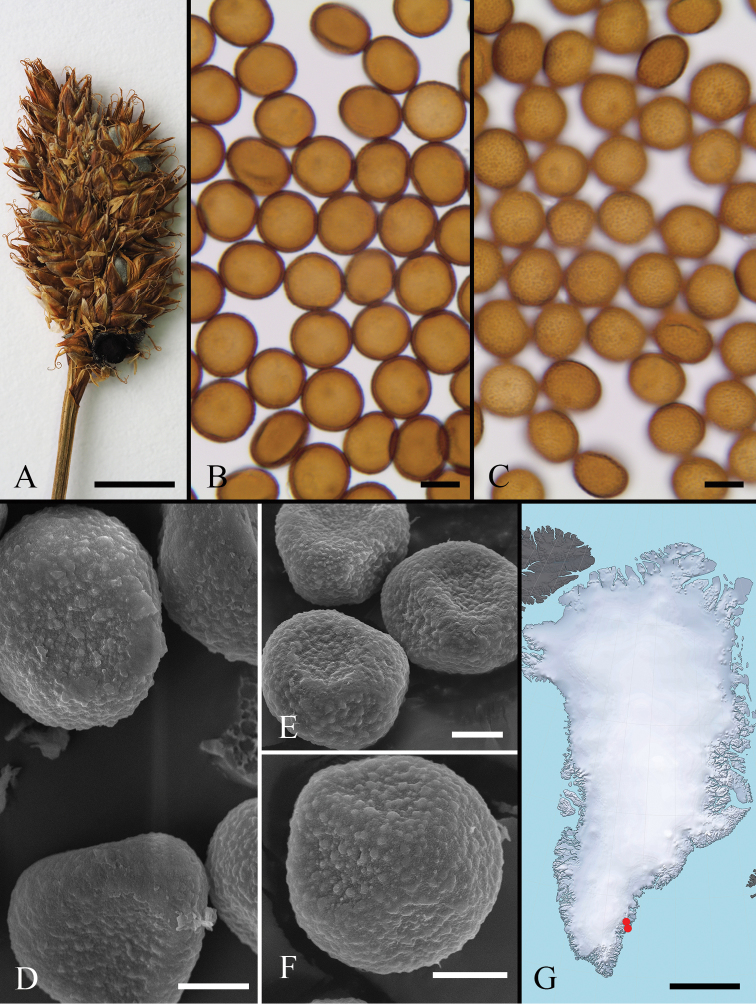
Anthracoidea verrucosa on Carex macloviana. **A** Habit (leg. J. Devold & P.F. Scholander, s.n., O, s.n.) **B, C** spores in LM, in median and surface view, respectively (M. Astrup & L. Kliim-Nielsen, no. G.B.U. 898, C-Greenland herb., s.n.) **D–F** spores in SEM (M. Astrup & L. Kliim-Nielsen, no. G.B.U. 898, C-Greenland herb., s.n.) **G** distribution map. Scale bars: 0.5 cm (**A**); 10 µm (**B, C**); 5 µm (**D–F**); 500 km (**G**).

**Hosts and distribution within the studied area** — On Cyperaceae: Carex sect. Ovales Kunth: Carex macloviana var. macloviana – East Greenland (Fig. 19G).

**Specimens examined or recorded.**

On **Carex macloviana** d’Urv. var. **macloviana**:

**EG**, Akorninarmiut, Skjoldungenområdet, Dronning Marias Dal, 63°28'N, 41°53'W, 5 Aug 1931, leg. B. Bjørlykke, s.n. (O!, s.n.; Hagen 1947, as ‘Ci. caricis’; Nannfeldt 1977); **ditto**, 24 Jul 1932, leg. J. Devold & P.F. Scholander, s.n. (O!, s.n.; Hagen 1947, as ‘Ci. caricis’; Nannfeldt 1977); **ditto**, 63°28'N, 41°55'W, 15 Aug 1970, leg. M. Astrup & L. Kliim-Nielsen, no. G.B.U. 898 (C-Greenland herb.!, s.n.).

**EG**, NW of Griffenfeldt Island (as ‘Umanak’), ca 63°03'N, 11 Sep 1932, leg. J. Devold, s.n. (O!, s.n., Hagen 1947, as ‘Ci. caricis’; Nannfeldt 1977).

**Known hosts** — On Cyperaceae: Carex sect. Ovales: Carex bebbii (L.H. Bailey) Olney ex Fernald, C. ebenea Rydb., C. illota L.H. Bailey, C. macloviana var. macloviana (C. festiva Dewey), C. microptera Mack. (C. festivella Mack.), C. pachystachya Cham. ex Steud. (C. macloviana var. pachystachya (Cham. ex Steud.) Kük.), C. phaeocephala Piper, C. preslii Steudel, C. subfusca W. Boott; Carex sect. Phaestoglochin: Carex hoodii Boott.

**General distribution. North America**: Alaska, Canada (British Columbia), western U.S.A., Greenland.

**Earlier reports from Greenland**: Hagen (1947, as ‘Ci. caricis’), Savile (1952, as ‘Ci. carpophila var. verrucosa’), Nannfeldt and Lindeberg (1957), Nannfeldt (1977, 1979), Vánky (2011).

**Comments** — Anthracoidea verrucosa infects North American sedges in the sections Ovales and Phaestoglochin. This smut fungus has been purposefully studied by Savile (1952) and Nannfeldt (1977, 1979) but in fact, information about its hosts and distribution continues to be insufficient. Based on available records (Hagen 1947; Savile 1952; Nannfeldt and Lindeberg 1957; Nannfeldt 1977; Farr and Rossman 2019), A. verrucosa is distributed on several closely related sedges in Alaska and mountains in British Columbia (Mt. Brent, Mt. Apex) and western U.S.A. (in Wyoming, Utah, Colorado, and Lessen Volcanic National Park in California), and on C. macloviana var. macloviana in East Greenland. Since C. macloviana is a widespread species, it is still unclear whether A. verrucosa is a smut fungus with a large disjunction in the distribution or this disjunction reflects insufficient sampling.

Carex macloviana var. macloviana is a sedge with bipolar distribution (in South America from Peru to Tierra del Fuego and Falkland Islands), being an amphi-Pacific–Cordilleran–North American (northern)–amphi-Atlantic taxon in the Northern Hemisphere — distributed there in Russian Far East, Hawaiian Islands, Alaska, Subarctic Canada, western U.S.A., Greenland, Iceland, and northern Fennoscandia (Hultén and Fries 1986: 466; Egorova 1999; Mastrogiuseppe et al. 2002; Elven et al. 2018). Surprisingly, on this sedge Anthracoidea verrucosa is known only from East Greenland (Hagen 1947).

Based on the cited records, A. verrucosa is a North American (northern)–Cordilleran species.

### Anthracoidea sp.

Allescher and Hennings (1897: 40) reported a collection of Cintractia caricis on Carex incurva made in 1892 in West Greenland. The name Carex incurva is reduced to a synonym of C. maritima (Govaerts 2018). Two smut fungi are known to infect flowers of C. maritima: Anthracoidea pseudofoetidae and Planetella lironis. In this treatment, both species are reported for the first time from Greenland.

The Allescher and Hennings’ specimen possesses spores 18–22 µm in diam., based on their measurement. If it is the real spore length, their collection certainly does not belong to Anthracoidea pseudofoetidae or Planetella lironis, which have much smaller spores — up to 12.5 µm and 14.5 µm, respectively. Nannfeldt (1979: 36) referred this collection to as ‘Anthracoidea sp. 5’. Unfortunately, no voucher specimen is known to exist which makes the interpretation of this record impossible.

**Specimen recorded.**

On **Carex maritima** Gunnerus:

**West Greenland**, Uummannaq Island (as ‘Umanak’), 70°41'N, 52°07'W, 28 Jun 1892, leg. E. Vanhöffen, s.n. (n.v.; Allescher and Hennings 1897, as ‘Ci. caricis’ on ‘Carex incurva’).

### Entyloma de Bary, Bot. Zeitung (Berlin) 32: 101, 1874. — Type: E. microsporum (Unger) J. Schröt.

**Sori** in vegetative parts of dicotyledonous host plants, mostly in leaves and stems, usually forming spots, sometimes pustules, swellings or galls. **Spores** solitary or adhering in irregular groups, permanently embedded in the host tissue, hyaline, yellow or pale yellowish brown; spore wall usually smooth, often with a hyaline gelatinous sheath. **Spore germination** of Tilletia-type. **Host-parasite interaction** by simple interaction apparatus, haustoria absent. **Septal pore** simple, with two membrane caps. **Anamorph** present in some species (Vánky 2013).

#### 1(20) Entyloma microsporum (Unger) J. Schröt., s. lat., in Rabenhorst, Fungi Eur. Exsicc., no. 1872, 1874. ≡ Protomyces microsporus Unger, Die Exantheme der Pflanzen, etc.: 343, 1833. ≡ Entyloma ungerianum de Bary, Bot. Zeitung (Berlin) 32: 101, 1874 (nom. nov. superfl. pro P. microsporus). — Neotype on Ranunculus repens L., Germany, Hesse, county Groß-Gerau, Ginsheim-Gustavsburg, bikeway to Mainspitzdreieck, wayside, 49°59'37"N, 08°17'46"E, 17 Nov 2013, leg. J. Kruse (GLM-F107661) (design. by Kruse et al. 2018: 185).

= Entyloma microsporum var. pygmaeum Allesch., in Allescher and Hennings, Bibliotheca Botanica 8(42): 40, 1897. ≡ Entyloma pygmaeum (Allesch.) Cif., Ann. Mycol. 26: 51, 1928. — Type on Ranunculus pygmaeus, Greenland, Qarassap Nunataa (as ‘Karajak-Nunatak’), 24 Jul 1893, leg. E. Vanhöffen.

**Hosts and distribution within the studied area** — On Ranunculaceae: Ranunculus pygmaeus – West Greenland.

**Specimens recorded.**

On **Ranunculus pygmaeus** d’Urv.:

**West Greenland**, Umanakfjord, Qarassap Nunataa (as ‘Karajak-Nunatak’), 70°28'N, 50°33'W, 24 Jul 1893, leg. E. Vanhöffen, s.n. (n.v.; Allescher and Hennings 1897: 40).

For description and illustrations of this smut fungus, see Vánky (2011).

**Known hosts** — On Ranunculaceae: on 30 species of Ranunculus (see Vánky (2011: 195).

**General distribution.** Cosmopolitan.

**Earlier reports from Greenland**: Allescher and Hennings (1897), Clinton (1902, 1904, 1906).

**Comments** — Entyloma microsporum var. pygmaeum is recorded only from the type locality. No voucher specimen is known to exist.

### Haradaea Denchev, in Denchev, Moore, and Shin, Mycol. Balcanica 3: 72, 2006. — Type: H. duriaeana (Tul. & C. Tul.) Denchev & H.D. Shin.

**Sori** in ovules of plants belonging to Caryophyllaceae, filling the capsules with a purplish or dark reddish brown spore mass; peridium and columella lacking, sterile cells absent. **Spores** single; spore wall reticulate, rarely incompletely reticulate.

The genus Haradaea was described for accommodation of a group of former Ustilago species on caryophyllaceous plants that destroy ovules, filling the capsules with a purplish or dark reddish brown spore mass (Denchev et al. 2006a, b). Haradaea comprises nine species.

#### 1(21) Haradaea nivalis (Liro) Denchev & H.D. Shin, in Denchev, Moore, and Shin, Mycol. Balcanica 3: 72, 2006. ≡ Ustilago nivalis Liro, Ann. Acad. Sci. Fenn., Ser. A 17(1): 42, 1924. ≡ Microbotryum nivale (Liro) Vánky, Mycotaxon 67: 47, 1998. — Holotype on Sagina nivalis, Svalbard, Spitsbergen, Advent Bay, 9 Aug 1882, leg. A.G. Nathorst (H).

Fig. 20A–F

**Infection** systemic, all capsules of an infected plant affected. **Sori** in ovules, filling the swollen capsules with a semi-agglutinated, dark reddish brown spore mass. **Spores** subglobose, broadly ellipsoidal, slightly irregular, globose, ovoid or ellipsoidal, (9.5–) 10–13(–14) × (9–)9.5–11.5(–12.5) (11.5 ± 0.7 × 10.6 ± 0.6) µm (n/_2_ = 200), light to medium vinaceous; wall reticulate, (1.5–)1.7–2.2(–2.4) µm thick (including reticulum), meshes (5–)6–8(–9) per spore diameter, polyhedral or irregular, 0.5–1.7(–2.5) μm wide, muri up to 0.9(–1.1) µm high. Immature hyaline spores may be present. In SEM meshes minutely verruculose on the bottom, with a hemispherical protuberance.

**Figure 20.**
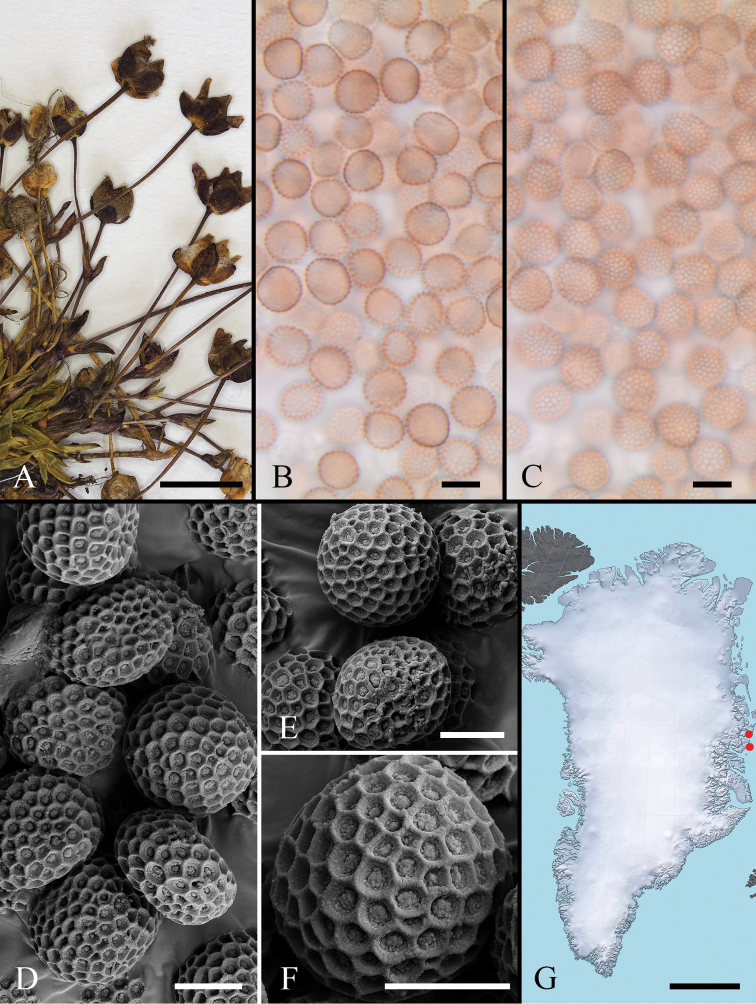
Haradaea nivalis on Sagina intermedia. **A** Habit (leg. S. Aandstad, s.n., O, s.n.) **B, C** spores in LM, in median and surface view, respectively (leg. A. Hagen, s.n., O, s.n.) **D–F** spores in SEM (leg. S. Aandstad, s.n., O, s.n.) **G** distribution map. Scale bars: 0.5 cm (**A**); 10 µm (**B, C**); 5 µm (**D–F**); 500 km (**G**).

**Hosts and distribution within the studied area** — On Caryophyllaceae: Sagina nivalis (S. intermedia Fenzl ex Ledeb.) – East Greenland (Fig. 20G).

**Specimens examined or recorded.**

On **Sagina nivalis** (Lindblad) Fr.:

**EG**, Clavering Island, Kap Mary, 74°11'N, 5 Aug 1933, leg. A. Hagen, the Norwegian Expedition to NE Greenland 1933, s.n. (O!, s.n., as ‘*U. nivalis*’; Hagen 1941, 1947, as ‘*U. nivalis*’).

**EG**, Hold with Hope, Kap Hold with Hope Station, ca 73°30'N, 13 Aug 1932, leg. S. Aandstad, the Norwegian Expedition to Eirik Raudes Land 1932, s.n. (O!, s.n., as ‘*U. nivalis*’; Hagen 1941, 1947, as ‘*U. nivalis*’).

**Known hosts** — On Caryophyllaceae: Sagina apetala Ard., S. nivalis.

**General distribution. Arctic Europe**: Svalbard. **North America**: Greenland. **Australasia**: Australia, New Zealand.

**Earlier reports from Greenland**: Hagen (1941, 1947, as ‘*U. nivalis*’).

**Comments** — Haradaea nivalis is a rare species, so far reported only from Spitsbergen and East Greenland, on Sagina nivalis (Liro 1924; Hagen 1941, 1947; Lind 1928), and from Australia and New Zealand, on S. apetala (Brook 1957, as ‘Ustilago duriaeana’; Vánky and McKenzie 2002; Vánky and Shivas 2008).

Sagina nivalis is a circumpolar species (Hultén and Fries 1986: 758; Elven et al. 2018) while S. apetala is a Euro-Mediterranean species (Hultén and Fries 1986: 763; Marhold 2011) that is naturalized in many regions outside its native range (e.g., in North America, Australia, New Zealand). There is no simple explanation for the large disjunction in the distribution of Haradaea nivalis. According to Vánky and McKenzie (2002) and Vánky and Shivas (2008), there is a small morphological difference in the spores of the fungus on Sagina apetala compared with that on S. nivalis. Molecular data are not available to clarify whether or not the fungi on these host plants belong to distinct species. Until this problem is resolved, we consider H. nivalis as an Arctic species.

### Microbotryum Lév., Ann. Sci. Nat., Bot., Sér. 3, 8: 372, 1847. — Type: M. violaceum (Pers.) G. Deml & Oberw.

**Sori** in various organs of the host plants in dicotyledonous families. Spore mass dusty, pale to dark purplish brown. **Spores** solitary; surface variously ornamented (often reticulate, also echinulate, verrucose or striate). Peridium, columella and capillitium-like threads absent in the sori. **Sterile cells** absent between the spores that are not catenulate. **Spore germination** results in phragmobasidia with successive production of sessile basidiospores, sterigmata absent. **Host-parasite interaction** by intercellular hyphae lacking interactions with deposits of specific fungal vesicles. Mature **septa** poreless (Vánky 2013).

#### Key to the relevant Microbotryum species, based on host plant taxonomy

**Table:** 

**On Caryophyllaceae** (Sori in the anthers)		
On Silene acaulis		**M. silenes-acaulis**
On Silene uralensis		**M. arcticum**
On Stellaria		**M. stellariae**
On Viscaria		**M. lagerheimii**
**On Polygonaceae**		
On Bistorta		
1	Sori in the inflorescences, destroying flowers and bulbils	**M. bistortarum**
1*	Sori in leaves, as rounded pustules, scattered or arranged in two rows along the median vein	**M. pustulatum**
On Koenigia. Sori in the stem or leaves		**M. koenigiae**
On Oxyria. Sori in the four perianth-segments of each flower, swelling them considerably		**M. vinosum**

#### 1(22) Microbotryum arcticum T. Denchev, Denchev, Kemler & Begerow, in Denchev et al., Willdenowia 49: 246, 2019. — Holotype on Silene uralensis subsp. arctica, Greenland, Peary Land, 10 km NW of Mudderbugt, just S of Ndr. Ladegårdså, 82°29–30'N, 21°30–35'W, 7 Aug 1991, leg. B. Fredskild 91-433 (SOMF 29 999!).

Fig. 21A–F

**Infection** systemic. **Sori** in the considerably swollen anthers, filling the pollen sacs with a pulverulent, dark livid or livid vinaceous spore mass. **Spores** subglobose, globose, broadly ellipsoidal or ovoid, sometimes ellipsoidal or slightly irregular, (5–)5.5–7.5(–8.5) × (4.5–)5–6.5(–7.5) (6.5 ± 0.5 × 6.0 ± 0.4) μm (n/_5_ = 700), pale vinaceous; wall reticulate, 0.8–1.3(–1.5) μm thick (including reticulum); meshes 5–8(–9) per spore diameter, polyhedral or irregular, 0.3–1.0(–1.5) μm long; muri (15–)16–21(–23) on equatorial circumference, up to 0.4 μm high; in SEM meshes smooth or rugulose on the bottom. **Spore germination** (after Parmelee, in Denchev et al. 2019) results in a 4-celled basidium, separating during basidiospore formation as a 3-celled basidium (one cell remaining attached to the teliospore) and producing basidiospores laterally and terminally.

**Figure 21.**
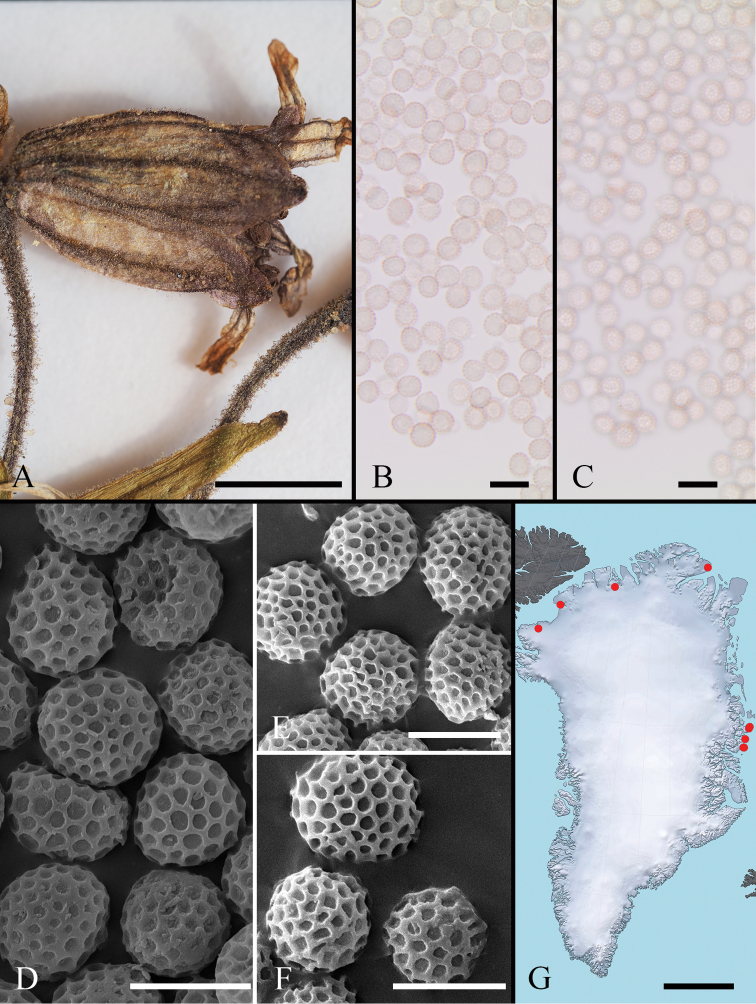
Microbotryum arcticum on Silene uralensis subsp. arctica. **A** Habit (holotype) **B, C** spores in LM, in median and surface view, respectively (holotype) **D–F** spores in SEM (**D** leg. P. Frykman & B. Fredskild, s.n., C-Greenland herb., s.n.; **E, F** SOMF 29 998) **G** distribution map. – A, D, E reproduced, with permission, from Denchev et al. 2019. Scale bars: 0.5 cm (**A**); 10 µm (**B, C**); 5 µm (**D–F**); 500 km (**G**).

**Hosts and distribution within the studied area** — On Caryophyllaceae: Silene uralensis subsp. arctica – North and East Greenland (Fig. 21G).

**Specimens examined or recorded.**

On **Silene uralensis** subsp. **arctica** (Fr.) Bocquet:

**NG**, Peary Land, 10 km NW of Mudderbugt, just S of Ndr. Ladegårdså, 82°29–30'N, 21°30–35'W, 7 Aug 1991, leg. B. Fredskild, no. 91-433 (SOMF 29 999, ex C-Greenland herb.!, s.n., the host as ‘Melandrium apetalum subsp. arcticum (Fr.) Hultén’).

**NG**, Warming Land, GGU (Grønlands Geologiske Undersøgelse) Base Camp, 81°32'N, 51°31'W, 13 Aug 1985, leg. C. Bay, no. 85-434 (C-Greenland herb.!, s.n., the host as ‘*M. apetalum* subsp. *arcticum*’).

**NG**, Washington Land, Cass Fjord, Nygaard Bugt, 80°06'N, 65°10'W, alt. 10 m, 5 Aug 1976, leg. P. Frykman & B. Fredskild, s.n. (C-Greenland herb.!, s.n., the host as ‘*M. apetalum* subsp. *arcticum*’).

**NG**, Inglefield Land, central inland, plain plateau, alt. 450 m, 78°40'N, 68°18'W, 16 Aug 1999, leg. J. Feilberg, no. 534 (SOMF 29 998, ex C-Greenland herb.!, s.n., the host as ‘*S. uralensis* subsp. *apetala* (L.) Bocquet’).

**EG**, Sabine Island, Germania Havn (on the south side of the island, ca 74°32.2'N, 18°49.9'W), 22 Jul 1932 & 16 Aug 1932, leg. S. Aandstad (n.v.; not found in O; Hagen 1947, as ‘U. violacea’); **ditto**, 21 Jul 1933, leg. A. Hagen, the Norwegian Expedition to NE Greenland 1933, s.n. (n.v.; not found in O; Hagen 1947, as ‘U. violacea’).

**EG**, Wollaston Forland, Landingsdalen, ca 74°27.5'N, 19°03.1'W, 28 Jul 1929, leg. J. Vaage, s.n. (O-V-688113!; Hagen 1947, as ‘U. violacea’).

**EG**, Gael Hamke Bugt, Jackson Island, ca 73°55'N, 11 Aug 1933, leg. A. Hagen, the Norwegian Expedition to NE Greenland 1933, s.n. (n.v.; not found in O; Hagen 1947, as ‘U. violacea’).

**EG**, Hold with Hope, Stormdalen, ca 73°29.5'N, 20°46.9'W, 9 Aug 1933, leg. A. Hagen, the Norwegian Expedition to NE Greenland 1933, s.n. (n.v.; not found in O; Hagen 1947, as ‘U. violacea’); **ditto**, Troldsøen (as ‘Trollvatnet’), ca 73°29'N, 20°39'W, 9 Aug 1933, leg. A. Hagen, the Norwegian Expedition to NE Greenland 1933, s.n. (n.v.; not found in O; Hagen 1947, as ‘U. violacea’).

**Known hosts** — On Caryophyllaceae: Silene uralensis subsp. arctica.

**General distribution. Arctic North America**: Canada, Greenland.

**Earlier reports from Greenland**: Denchev et al. (2019).

**Comments** — The anther-smut fungi of Microbotryum on hosts in the Caryophyllaceae cause formation of teliospores instead of pollen in the anthers of bisexual flowers. When female flowers of dioecious species (e.g., in the cases of Silene latifolia and S. dioica) are infected, suppression of stamen development does not occur, and development of spore-bearing anthers is induced (Kazama et al. 2005). The most widely studied anthericolous smuts are those in the anthers of Silene. It is a group of seventeen, highly host specific fungi. Regarding the sorus morphology, they may be divided into two groups: (i) species causing typical anther infection, with sori restricted to the anthers (four species, M. arcticum, M. lagerheimii, M. silenes-acaulis, and M. stellariae, in Greenland); and (ii) species causing atypical infection, with sori usually formed not only in the anthers but also in the filaments, and causing formation of swollen and deformed flowers, completely filled with spore mass (one species, M. savilei, potentially occurring in Greenland) (Denchev et al. 2019).

Microbotryum arcticum on Silene uralensis subsp. arctica was recently described from the High Arctic of Greenland and the Canadian Arctic Archipelago (Denchev et al. 2019). Four specimens from North Greenland (including the holotype) and a specimen from East Greenland are listed in the protologue, as examined. Other six specimens from East Greenland — reported by Hagen (1947) as ‘U. violacea’ on ‘Melandrium apetalum’, but not found in the herbarium in Oslo — were also considered as belonging to M. arcticum (Denchev et al. 2019).

The taxonomic status of the host plant was briefly discussed in Denchev et al. (2019). Silene uralensis (sect. Physolychnis) is a very variable species complex (Morton 2005) with not completely clarified specific and infraspecific delimitation. In Bocquet’s treatment of Silene sect. Physolychnis (1967), four subspecies have been recognized within Silene uralensis: uralensis, apetala, arctica, and porsildii (a tetraploid plant). The populations in Svalbard have been treated as belonging to an endemic subspecies, arctica, while those in Scandinavia and Bering Sea islands have been recognized as subsp. apetala (S. wahlbergella Chowdhuri). The remaining populations have been referred to as subsp. uralensis (with a northern circumpolar distribution). Hultén (1968) accepted two subspecies: Melandrium apetalum subsp. arcticum , mapped by him as having a circumpolar distribution, and subsp. apetalum from Scandinavia. In *Flora Nordica* (Jonsell 2001), however, the Fennoscandian plants were treated as a distinct species, S. wahlbergella, and accordingly, only the Arctic plants from North America and Asia were related to S. uralensis. In the Silene treatment for *Flora of North America* (Morton 2005), three subspecies were recognized within the S. uralensis complex: subsp. uralensis, as a widespread, Arctic circumpolar entity; subsp. porsildii, distributed in Yukon, Alaska, and Arctic Asia; and subsp. ogilviensis from the Canadian Low Arctic. Of these, only subsp. uralensis was given as represented in Greenland and the eastern Canadian Arctic Archipelago. Elven et al. (2018) disagreed with Bocquet’s view that subsp. arctica was restricted to Svalbard, and recognized three subspecies of S. uralensis: subsp. uralensis, with a circumpolar distribution (NE Europe, Arctic Asia, Bering Sea islands, Alaska, Canada, and W & S Greenland); subsp. arctica, also with a circumpolar distribution (Arctic Far East of Russia, northernmost Alaska and Canada, Greenland, and Svalbard); and subsp. ogilviensis.

Thus, according to the taxonomic scheme of Elven et al. (2018), two subspecies of S. uralensis are represented in Greenland. Silene uralensis subsp. uralensis is characterized by a calyx that is not strongly inflated and usually longer than broad, and petals slightly emerging from the calyx, less so than in subsp. arctica (Elven et al. 2018). The calyx of S. uralensis subsp. arctica is inflated, in flower stage ca. 1.5 times as long as broad (Alsos et al. 2018). In Greenland, there is an overlap in the ranges of the northern subsp. arctica and more southern subsp. uralensis at 70–71°N but there are no obvious transitional plants (Elven et al. 2018). In the first half of the last century, the High Arctic entity in Greenland, arctica, was referred to as ‘Melandrium apetalum’ (e.g., Kruuse 1905; Ostenfeld and Lundager 1910; Hartz and Kruuse 1911; Ostenfeld 1926; Porsild, M. 1926). The southernmost localities of subsp. arctica are at 69°42'N in West Greenland (Porsild, M. 1926) and 69°30'N in East Greenland (Kruuse 1905) while northwards it reaches 83°06'N (Maguire 1950). Considering that subsp. uralensis is distributed only on the west and south coasts of Greenland, the host plant of the Microbotryum specimens recorded by Hagen (1947) on ‘Melandrium apetalum’ from East Greenland (at 73°29'–74°32'N) were accepted as belonging to subsp. arctica (Denchev et al. 2019).

In Canada, M. arcticum is recorded from the eastern Arctic Archipelago (Ellesmere Is., Axel Heiberg Is., Somerset Is., Baffin Is., and Mansel Is.; Denchev et al. 2019).

On the same host plant, another Microbotryum species, M. savilei, is known from the eastern Canadian Arctic Archipelago (Southampton Island) (Denchev 2007b; Denchev et al. 2019). Microbotryum savilei causes atypical infection and can be differentiated from M. arcticum by the soral morphology, changes in the affected flowers, and spore sizes.

#### 2(23) Microbotryum bistortarum (DC.) Vánky, Mycotaxon 67: 40, 1998. ≡ Uredo bistortarum var. bistortarum DC., Fl. Franç., ed. 3 (Paris) 6: 76, 1815 (as ‘γ ustilaginea’). ≡ Ustilago ustilaginea (DC.) Liro, Ann. Acad. Sci. Fenn., Ser. A 17(1): 7, 1924. ≡ Sphacelotheca ustilaginea (DC.) S. Ito, Trans. Sapporo Nat. Hist. Soc. 14: 90, 1935. ≡ Sphacelotheca ustilaginea (DC.) Cif., Fl. Ital. Crypt., Pars I, Fungi, Fasc. 17: 273, 1938 (comb. superfl.). — Lectotype on Bistorta vivipara, France, leg. A.P. de Candolle (design. by Lindeberg 1959: 111).

Fig. 22A–F

= Ustilago candollei Tul. & C. Tul., Ann. Sci. Nat., Bot., Sér. 3, 7: 93, 1847 [based on ‘Uredo bistortarum γ ustilaginea DC. Fl. Fr. VI, 76 (pro parte?)’]. — Type on Bistorta major, France, leg. A.P. de Candolle.

= Sphacelotheca hydropiperis var. borealis G.P. Clinton, Proc. Boston Soc. Nat. Hist. 31: 395, 1904. ≡ Sphacelotheca borealis (G. P. Clinton) Schellenb., Ann. Mycol. 5: 386, 1907. ≡ Ustilago borealis (G.P. Clinton) Cif., Omagiu lui Traian Savulescu (Bucharest): 166, 1959. — Type on Bistorta bistortoides (Pursh) Small, U.S.A., Washington, Rainier Mt., August 1895, leg. C.V. Piper (BPI 177282, 177283).

= Ustilago bistortarum [unranked] inflorescentiae Trel., in Saccardo, Peck, and Trelease, Harriman Alaska Exped. 5 (Crypt. Bot.): 35, 1904. ≡ Ustilago inflorescentiae (Trel.) Maire, in Brockmann-Jerosch and Maire, Oesterr. Bot. Z. 57: 273, 1907. ≡ Sphacelotheca inflorescentiae (Trel.) Jaap, Ann. Mycol. 6: 194, 1908. — Lectotype on Bistorta vivipara, U.S.A., Alaska, Kodiak Isl., 1899, leg. W. Trelease, no. 675. (BPI 177282, 177283) (design. by Lindeberg 1959: 112).

= Sphacelotheca polygoni-vivipari Schellenb., Ann. Mycol. 5: 388, 1907. — Type on Bistorta vivipara, Europe, the Alps, 1899, leg. H.C. Schellenberg..

**Infection** systemic. **Sori** in all flowers of an infected plant; spore mass semi-agglutinated to powdery, dark reddish brown. **Spores** subglobose, slightly irregular, broadly ellipsoidal, ovoid, globose or ellipsoidal, (8.5–)9.5–15.5(–17) × (8–)9.5–13.5(–15) (12.3 ± 1.4 × 11.1 ± 1.1) µm (n/_3_ = 300), medium vinaceous; wall 0.7–1.2 µm thick, moderately verruculose, warts up to 0.4(–0.5) µm high, spore profile affected. Immature hyaline spores, smooth or with verruculose wall, may be present. In SEM warts usually isolated, sometimes confluent in short rows or small groups; wall surface punctate between the warts. **Spore germination** (after Ingold 1988: 505, Fig. 1) results in a four-celled basidium with width tending to increase from base to apex, producing basidiospores laterally on each cell.

**Figure 22.**
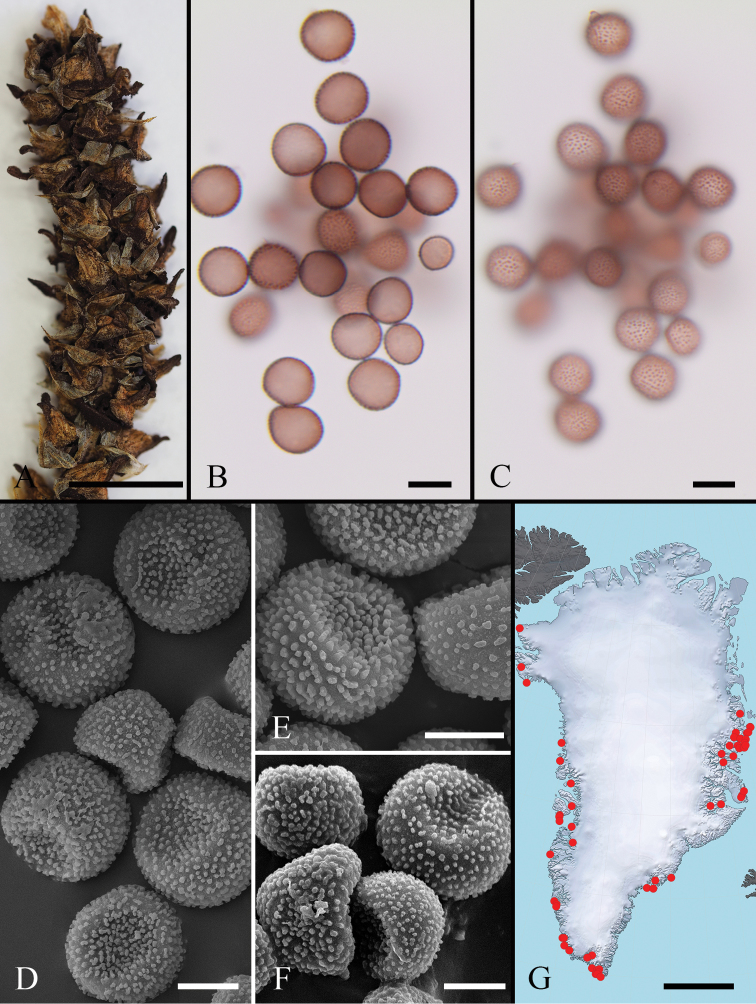
Microbotryum bistortarum on Bistorta vivipara. **A** Habit (leg. A. Hagen, s.n., O, s.n.) **B, C** spores in LM, in median and surface view, respectively (B. Hanfgarn & B. Jacobsen, no. 553, C-Greenland herb., s.n.) **D–F** spores in SEM (**D, E** J. Feilberg, no. 1189, C-Greenland herb., s.n.; **F** B. Hanfgarn & B. Jacobsen, no. 553, C-Greenland herb., s.n.) **G** distribution map. Scale bars: 0.5 cm (**A**); 10 µm (**B, C**); 5 µm (**D–F**); 500 km (**G**).

**Hosts and distribution within the studied area** — On Polygonaceae: Bistorta vivipara – North, West, and East Greenland (Fig. 22G).

**Specimens examined or recorded.**

On **Bistorta vivipara** (L.) Delarbre:

**NG**, Etah Bay, 78°19'N, 11–12 Aug 1899, leg. R. Platt, no. 269 (n.v.; WSP 38240, as ‘Sph. hydropiperis’ on ‘Polygonum sp.’).

**NG**, Wolstenholme Fjord (as ‘Wolstenholme Sound’), Umanaq, at ca 76°30–33'N, 21 Jul 1916, leg. L. Koch, s.n. (C-F-107996!, as ‘U. Inflorescentiae’); **ditto**, Qaanaaq, 1988, leg. S.A. Elborne, no. SAE-88.274-GR (C-F-108006!, as ‘*U. bistortarum*’).

**NG**, Thule, Cape York, ca 75°54'N, 66°24'W, 31 Jul 1914, leg. P. Freuchen, s.n. (C-F-107997!, as ‘U. inflorescentiae’).

**WG**, Tasiusaq, 73°22'N, 29 Jul 1884, leg. E. Warming & Th. Holm, s.n. (C-F-102496!, as ‘Sph. hydropiperis’; Rostrup 1888, as ‘Sph. hydropiperis’).

**WG**, Prøven Island, 72°23'N, 21 Jul 1886, leg. K. Rosenvinge, s.n. (C-F-107998!, as ‘U. inflorescentiae’).

**WG**, Upernivik Island (N of Disko Island), ca 71°16'N, 52°45'W, 16 Jul 1886, leg. K. Rosenvinge, s.n. (C-F-102504!, as ‘Sph. hydropiperis’; Rostrup 1888; as ‘Sph. hydropiperis’).

**WG**, Nuussuaq Peninsula, W of Sarqaq, 70°00'N, 51°50'W, alt. 60 m, 8 Jul 1969, leg. G. Bengtsson, no. 47 (C-Greenland herb.!, s.n.).

**WG**, Disko Island, Blæsedalen, ca 69°21'N, 53°30'W, 3 Jul 1932, sine coll. (C-F-108009!, as ‘*U. bistortarum*’); **ditto**, Ins. Disko, E pag. Qeqertarsuaq (as ‘Godhavn’), in valle Bläsedal, 69°16'N, 53°30'W, alt. 10–20 m, 31 Jul 1982, leg. J. Poelt & H. Ullrich, s.n. (C-F-102469!, Vánky, Ustilaginales Exsiccata, no. 421, as ‘*U. bistortarum*’; Vánky 1983); Disko Island, Østerli near Qeqertarsuaq (as ‘Godhavn’), ca 69°15'N, 14 (on the label as ‘17’) Aug 1967, leg. M. Lange, no. 67-186 (C-F-108005!, as ‘*U. bistortarum*’); **ditto**, Lyngmarken near Qeqertarsuaq (as ‘Godhavn’), ca 69°15'N, 25 Jul 1886, leg. K. Rosenvinge, s.n. (C-F-102507!, as ‘Sph. hydropiperis’); **ditto**, Qeqertarsuaq, 27 Jul 1886, leg. K. Rosenvinge, s.n. (C-F-102506!, as ‘Sph. hydropiperis’; Rostrup 1888, as ‘Sph. hydropiperis’).

**WG**, Kronprinsens Islands (S of Disko Island), 16 Jul 1897, leg. C. Krusse, no. 353 (C-F-102501!, as ‘Sph. hydropiperis’).

**WG**, Qasigiannguit (as ‘Christianshåb’), 68°49'N, 1884, leg. E. Warming & Th. Holm, s.n. (n.v.; not found in C; Rostrup 1888, as ‘Sph. hydropiperis’).

**WG**, Arfersierfik, Itivdliarssuk, 67°54'N, 50°34'W, alt. 10 m, 9 Jul 1987, leg. B. Fredskild & V. Dalgaard, no. 87-78 (C-Greenland herb.!, s.n.).

**WG**, Sisimiut (as ‘Holstensborg’), 66°56'N, 1 Aug 1884, leg. E. Warming & Th. Holm, s.n. (C-F-102494!, as ‘Sph. hydropiperis’; Rostrup 1888, as ‘Sph. hydropiperis’).

**WG**, Kerortusok, 66°55'N, 1884, leg. E. Warming & Th. Holm, s.n. (n.v.; not found in C; Rostrup 1888, as ‘Sph. hydropiperis’).

**WG**, Nuuk-area, Karra in Godthåbsfjord, 5 Aug 1976, leg. E. Neergaard, no. 76-150 (CP 1023603, n.v.; CP 1023605, n.v.).

**WG**, Godthåbsfjord (as ‘Baals Revier’), 64°08–45'N, July 1831, leg. J. Vahl, s.n. (C-F-102495!, as ‘Sph. hydropiperis’; Rostrup 1888, as ‘Sph. hydropiperis’).

**WG**, Nuuk (Godthåb), Kangiliartorfik, 64°15'N, 1885, leg. S. Hansen, s.n. (n.v.; not found in C; Rostrup 1888, as ‘Sph. hydropiperis’); **ditto**, Nuuk (as ‘Godthåb’), 20 Jul 1895, leg. ? (C-F-102486!, as ‘Sph. hydropiperis’).

**WG**, Præstefjord, 64°01'N, 51°17'W, alt. 100 m, 21 Jul 1973, leg. J. Feilberg, no. G.B.U. 5235 (C-Greenland herb.!, s.n.).

**WG**, Buksefjorden, 3 km N of Amitsorssuaq, 63°52'30"N, 51°17'W, alt. 110 m, 3 Aug 1979, leg. B. Hanfgarn & B. Jacobsen, no. 553 (C-Greenland herb.!, s.n.).

**WG**, Paamiut (as ‘Frederikshåb’), ca 62°00'N, 49°40'W, 15 Aug 1886, leg. K. Rosenvinge, s.n. (C-F-102505!, as ‘Sph. hydropiperis’; Rostrup 1888, as ‘Sph. hydropiperis’).

**WG**, Kvane Fjord (as ‘Kuanersok’), 62°N, 11 Jul 1889, leg. N. Hartz, s.n. (C-F-102508!, as ‘Sph. hydropiperis’; Rostrup 1891, as ‘Sph. hydropiperis’).

**WG**, Smallesund, ca 61°32'N, 15 Jun 1888, leg. K. Rosenvinge, s.n. (C-F-102509!, as ‘Sph. hydropiperis’; Rostrup 1891, as ‘Sph. hydropiperis’).

**WG**, Arsuk, 61°17'N, 48°30'W, 25 Jul 1957, leg. S. Lægaard, no. 359 (C-Greenland herb.!, s.n.).

**WG**, Narsarsuaq, Hospitalsdalen, 61°10'N, 45°25'W, 10 Aug 2018, leg. H.F. Gøtzsche, no. HFG 2018, 004 (C-F-113174!).

**WG**, Narssaq, Ilimaussaq, 60°59'N, 46°01'W, alt. 100–200 m, 30 Jul 1978, leg. S. Holt, no. 1426 (C-Greenland herb.!, s.n.).

**WG**, Sermersooq, near Qaqortoq (as ‘Julianehåb’), 60°21'N, 45°14'W, alt. 25, 22 Jul 1975, leg. J. Feilberg, no. 1189 (C-Greenland herb.!, s.n.).

**WG**, Tasermiut Fjord, Qinqua-valley at Taserssuaq Lake, 60°16'N, 44°33'W, 1 Aug 1984, leg. H. Knudsen, no. HK 84.283 (C-F-107982!, as ‘*U. bistortarum*’).

**WG**, Narsarmijit (as ‘Frederiksdal’), ca 60°00'N, 44°40'W, 24 Jul 1883, leg. P. Eberlin, s.n. (C-F-107999!, as ‘U. inflorescentiae’).

**WG**, Kangikitsoq, 60°20'N, 44°17'W, 29 Jul 1964, leg. C. Hansen & P.M. Petersen, no. 64-190 (C-Greenland herb.!, s.n.).

**WG**, Pamiagdluk, Sagsivik, 60°07'N, 44°20'W, 5 Jul 1967, leg. C. Hansen et al., no. 67-915 (C-Greenland herb.!, s.n.); **ditto**, Kûngmiut, 60°00'N, 44°28'W, alt. 100 m, 3 Jul 1967, leg. C. Hansen et al., no. 67-1001 (C-Greenland herb.!, s.n.).

**WG**, Eggers Ø, Eqaluit, 59°51'N, 44°00'W, 12 Jul 1967, leg. C. Hansen et al., no. 67-1158 (C-Greenland herb.!, s.n.).

**EG**, Dronning Margrethe II Land, Hochstetter Forland, Jónsbú (NE of the mouth of Ardencaple Fjord), ca 75°19.2'N, 20°23.3'W, 3 Aug 1933, leg. A. Hagen, the Norwegian Expedition to NE Greenland 1933, s.n. (O!, s.n., as ‘U. inflorescentiae’).

**EG**, Sabine Island, Germania Havn (on the south side of the island), ca 74°32.2'N, 18°49.9'W, 21 Jul 1933, leg. A. Hagen, the Norwegian Expedition to NE Greenland 1933, s.n. (O!, s.n., three specimens, as ‘U. inflorescentiae’).

**EG**, Hvalrosø Island (as ‘Kvalrossoya’), ca 74°30.8'N, 18°45.8'W, 21 Jul 1933, leg. A. Hagen, the Norwegian Expedition to NE Greenland 1933, s.n. (O!, s.n., as ‘U. inflorescentiae’).

**EG**, Revet, ca 74°21.7'N, 21°51.4'W, 22 Jul 1930, leg. J. Vaage, s.n. (O!, s.n., as ‘U. inflorescentiae’).

**EG**, Wollaston Forland, near Herschellhus (Kap Herschel), ca 74°14.6'N, 19°41.1'W, 1 Aug 1933, leg. A. Hagen, the Norwegian Expedition to NE Greenland 1933, s.n. (O!, s.n., as ‘U. inflorescentiae’).

**EG**, Kap Borlase Warren, ca 74°16.0'N, 19°22.7'W, 1900, leg. C. Krusse, s.n. (C-F-102491!, G. Amdrup’s Expedition to East Greenland in 1898–1900, as ‘Sph. hydropiperis’; Rostrup 1904, as ‘Sph. hydropiperis’).

**EG**, Jordanhill (at the front of Wordie Gletscher), ca 74°07.6'N, 22°19.9'W, 20 Jul 1933, leg. A. Hagen, the Norwegian Expedition to NE Greenland 1933, s.n. (O!, s.n., as ‘U. inflorescentiae’).

**EG**, Vesle Finsch Island, ca 74°00'N, 18 Jul 1933, leg. A. Hagen, the Norwegian Expedition to NE Greenland 1933, s.n. (O!, s.n., two specimens, as ‘U. inflorescentiae’).

**EG**, Gael Hamke Bugt, Jackson Island (ca 73°55'N), route 1, 11 Aug 1933, leg. A. Hagen, the Norwegian Expedition to NE Greenland 1933, s.n. (O!, s.n., as ‘U. inflorescentiae’); **ditto**, route 2, 11 Aug 1933, leg. A. Hagen, the Norwegian Expedition to NE Greenland 1933, s.n. (O!, s.n., as ‘U. inflorescentiae’); **ditto**, route 3, 12 Aug 1933, leg. A. Hagen, the Norwegian Expedition to NE Greenland 1933, s.n. (O!, s.n., as ‘U. inflorescentiae’).

**EG**, Hudson Land, Hoelsbo (as ‘Hoelsbu’, on the north side of Moskusoksefjord), ca 73°42.2'N, 23°26.3'W, 29 Jul 1933, leg. A. Hagen, the Norwegian Expedition to NE Greenland 1933, s.n. (O!, s.n., as ‘U. inflorescentiae’).

**EG**, Loch Fyne, ca 73°42'N, 4 Aug 1930, leg. G. Seidenfaden, no. 908 (C-F-108002!, as ‘*U. bistortarum*’).

**EG**, Hold with Hope, Knudshoved, ca 73°43.9'N, 20°27.1'W, 15 Aug 1933, leg. A. Hagen, the Norwegian Expedition to NE Greenland 1933, s.n. (O!, s.n., as ‘U. inflorescentiae’); **ditto**, on the beach N of Knudshoved, 19 Aug 1933, leg. A. Hagen, the Norwegian Expedition to NE Greenland 1933, s.n. (O!, s.n., two specimens, as ‘U. inflorescentiae’); **ditto**, as ‘Öyneset’, 16 Aug 1933, leg. A. Hagen, the Norwegian Expedition to NE Greenland 1933, s.n. (O!, s.n., as ‘U. inflorescentiae’).

**EG**, Hold with Hope, Myggbukta (on the north side of Mackenzie Bugt), ca 73°29.4'N, NW of the station, 22 Jul 1933, leg. A. Hagen, the Norwegian Expedition to NE Greenland 1933, s.n. (O!, s.n., as ‘U. inflorescentiae’); **ditto**, 1 km N of the station, 19 Aug 1933, leg. A. Hagen, the Norwegian Expedition to NE Greenland 1933, s.n. (O!, s.n., as ‘U. inflorescentiae’); **ditto**, on the coastal plain around the station, 31 Jul 1933, leg. A. Hagen, the Norwegian Expedition to NE Greenland 1933, s.n. (O!, s.n., as ‘U. inflorescentiae’).

**EG**, Hold with Hope, Stormdalen, ca 73°29.5'N, 20°46.9'W, 9 Aug 1933, leg. A. Hagen, the Norwegian Expedition to NE Greenland 1933, s.n. (O!, s.n., two specimens, as ‘U. inflorescentiae’); **ditto**, Troldsøen (as ‘Trollvatnet’), ca 73°29'N, 20°39'W, 9 Aug 1933, leg. A. Hagen, the Norwegian Expedition to NE Greenland 1933, s.n. (O!, s.n., as ‘U. inflorescentiae’).

**EG**, Holland Island, ca 73°36'N, 20°21'W, 13 Aug 1933, leg. A. Hagen, the Norwegian Expedition to NE Greenland 1933, s.n. (O!, s.n., as ‘U. inflorescentiae’).

**EG**, Ymer Island, Dusén Fjord, in the western part, ca 73°19'N, 7 Aug 1933, leg. A. Hagen, the Norwegian Expedition to NE Greenland 1933, s.n. (O!, s.n., as ‘U. inflorescentiae’); **ditto**, Kjelbotn (on the SE part of the island), 73°06.6'N, 23°00'W, 24 Jul 1933, leg. A. Hagen, the Norwegian Expedition to NE Greenland 1933, s.n. (O!, s.n., as ‘U. inflorescentiae’).

**EG**, Ella Island, 72°50'N, 17 Aug 1930, leg. G. Seidenfaden, the Godthaab Expedition 1930 to East-Greenland, no. 1039 (C-F-108000, as ‘*U. bistortarum*’).

**EG**, Jameson Land, leg. N. Hartz, C. Ryder’s Expedition to East Greenland in 1891–1892, s.n. (n.v.; not found in C; Rostrup 1894, as ‘Sph. hydropiperis’).

**EG**, Hurry Inlet (between Jameson Land and Liverpool Land), ca 70°50'N, 1900, leg. C. Krusse, G. Amdrup’s Expedition to East Greenland in 1898–1900, s.n. (C-F-102487!, as ‘Sph. hydropiperis’; Rostrup 1904, as ‘Sph. hydropiperis’); **ditto**, Fame Islands in Hurry Inlet, ca 70°50'N, 1900, leg. C. Krusse, G. Amdrup’s Expedition to East Greenland in 1898–1900, s.n. (C-F-102497!, as ‘Sph. hydropiperis’; Rostrup 1904, as ‘Sph. hydropiperis’); **ditto**, Constable Pynt, Gåseelv, 70°45'36"N, 22°39'W, leg. S.A. Elborne, no. SAE-2017.157-GR (C-F-107750!, as ‘*U. bistortarum*’).

**EG**, Liverpool Land, the east coast, Kangertivit Anginersaat (as ‘Storefjord’), N side, 71°05'N, 22°00'W, 22 Jul 1933, leg. A. Noe-Nygaard, no. 549 (C-F-107993!, as ‘U. inflorescentiae’).

**EG**, Røde Island, ca 70°27.7'N, 28°05'W, August 1891, leg. N. Hartz, C. Ryder’s Expedition to East Greenland in 1891–1892, s.n. (C-F-102503!, as ‘Sph. hydropiperis’; Rostrup 1894, as ‘Sph. hydropiperis’).

**EG**, Danmark Island, ca 70°30'N, 26°15'W, July 1892, leg. N. Hartz, C. Ryder’s Expedition to East Greenland in 1891–1892, s.n. (C-F-102502!, as ‘Sph. hydropiperis’; Rostrup 1894, as ‘Sph. hydropiperis’).

**EG**, Kap Wandel, 66°18'N, 34°53'W, 1 Aug 1899, leg. C. Krusse, G. Amdrup’s Expedition to East Greenland in 1898–1900, s.n. (C-F-102488!, as ‘Sph. hydropiperis’; Rostrup 1904, as ‘Sph. hydropiperis’).

**EG**, Kingorsuak near Tasiilaq Fjord, 66°08'N, 27 Jul 1902, leg. C. Krusse, s.n. (C-F-102493!, as ‘Sph. hydropiperis’; Rostrup 1904, as ‘Sph. hydropiperis’).

**EG**, Tasiilaq distr., Tasâlâlik, 65°39'N, 38°30'W, alt. 100 m, 1 Aug 1969, leg. L. Kliim-Nielsen, no. 69-3044 (C-Greenland herb.!, s.n.).

**EG**, Tasiilaq Island, Tasiusak, 65°37'N, 37°33'W, 8 Jul 1902, leg. C. Krusse, Expeditio Danica in Groenlandiam orientalem 1901–1902, s.n. (C-F-102498!, as ‘Sph. hydropiperis’).

**Known hosts** — On Polygonaceae: Bistorta bistortoides (Pursh) Small (Polygonum bistortoides Pursh), B. elliptica (Willd. ex Spreng.) D.F. Murray & Elven (P. ellipticum Willd. ex Spreng.), B. macrophylla (D. Don) Soják (P. macrophyllum D. Don), B. major Gray subsp. major (P. bistorta L.), B. major subsp. carnea Soják (P. carneumC. Koch), B. taipaishanensis (H.W. Kung) Yonek. & H. Ohashi (P. taipaishanense H.W. Kung), B. tenuifolia (H.W. Kung) Miyam. & H. Ohba (P. tenuifolium H.W. Kung), B. vivipara (P. viviparum L.).

**General distribution. Europe. Asia. North America**: Canada, Greenland, U.S.A.

**Earlier reports from Greenland**: Rostrup (1888, 1891, 1894, 1904, as ‘Sph. hydropiperis’), Clinton (1902, 1904, as ‘Sph. hydropiperis’; 1906, as ‘*U. bistortarum* var. inflorescentiae’), Lind (1927, 1934, as ‘U. inflorescentiae’), Hagen (1947, as ‘U. inflorescentiae’), Vánky (1983, as ‘*U. bistortarum*’).

**Comments** — In the past, Bistorta was merged with Polygonum or Persicaria. Currently, on the basis of molecular, morphological, and palynological data, it is considered as a distinct genus (e.g. Galasso et al. 2009; Hernández-Ledesma et al. 2015).

The principal host plant of M. bistortarum, Bistorta vivipara, is a circumboreal–polar species (Hultén and Fries 1986: 654; Elven et al. 2018).

Microbotryum bistortarum is one of the most widespread smut fungi in Greenland. It is a circumboreal–polar species. In Aleutian Islands, Arctic Canada, Greenland, Svalbard, Jan Mayen, Iceland, Faeroes, Fennoscandia, and Novaya Zemlya, M. bistortarum occurs nearly as co-distributed with Bistorta vivipara (Lind 1927, 1934; Hagen 1941, 1947, 1950a, b; Linder 1947; Savile 1953, 1959; Lindeberg 1959; Savile and Parmelee 1964; Parmelee 1969; Karatygin at al. 1999). Its northernmost collections are reported from Ellesmere Island (Canada), at 82°32'N (Savile 1959), and Floraberget in Murchisonfjorden (Spitzbergen), at 80°03'N (Hagen 1950b).

#### 3(24) Microbotryum koenigiae (Rostr.) Vánky, Mycotaxon 67: 45, 1998. ≡ Ustilago koenigiae Rostr., Meddel. Grønland 3: 532, 1888. — Holotype on Koenigia islandica, Greenland, Sisimiut, 6 Aug 1884, leg. E. Warming & T. Holm, s.n. (C-F-102468!).

Fig. 23A–F

**Sori** in the stem forming fusiform, broadly fusiform, reniform, crescent-shaped or irregularly elongated bodies or in leaves as irregular bodies that ruptures irregularly, exposing semi-agglutinated to pulverulent dark reddish brown spore mass. **Spores** variable in shape and size, subglobose, globose, ovoid, ellipsoidal or broadly ellipsoidal, sometimes elongated, irregular, pyriform or lacrymiform, (5–)6–9.5(–12) × (4.5–)5–7.5(–8.5) (7.5 ± 0.9 × 6.3 ± 0.5) µm (n/_3_ = 500), single irregularly elongated spores can reach up to 13.5 µm in length, light to medium vinaceous; wall 0.8–1.2 µm thick, in some spores indistinctly two-layered, finely striate; striae up to 0.2 µm high. In SEM spore wall striate, striae parallel or irregularly arranged, often ramifying, sometimes anastomosing.

**Figure 23.**
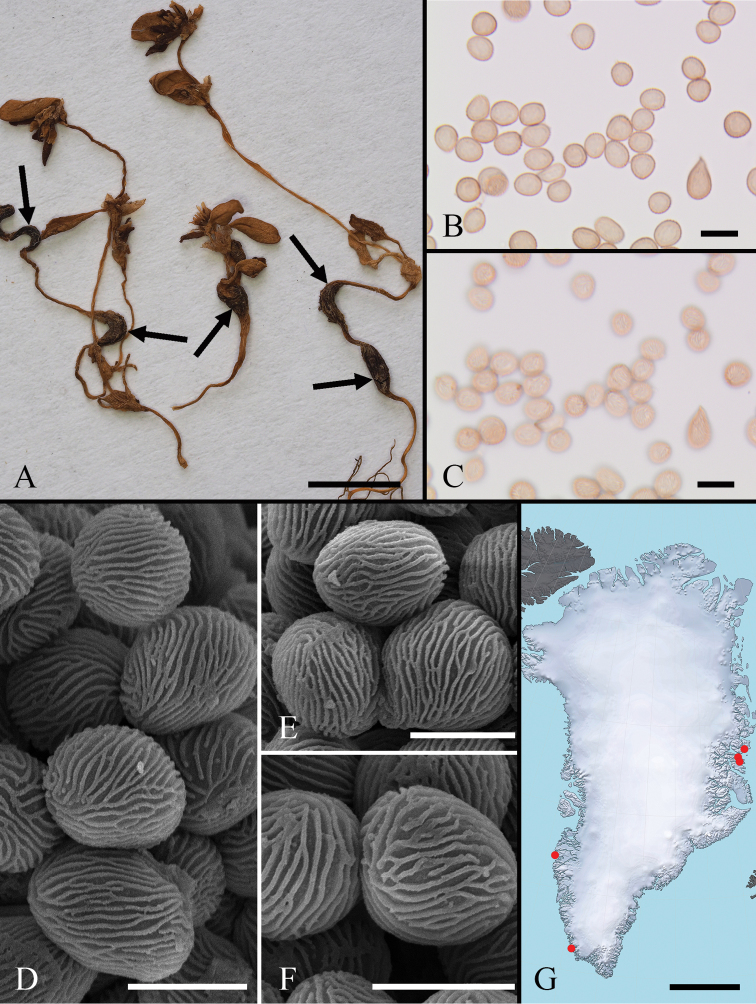
Microbotryum koenigiae on Koenigia islandica (holotype). **A** Habit (black arrows show sori) **B, C** spores in LM, in median and surface view, respectively **D–F** spores in SEM**G** distribution map. Scale bars: 0.5 cm (**A**); 10 µm (**B, C**); 5 µm (**D–F**); 500 km (**G**).

**Hosts and distribution within the studied area** — On Polygonaceae: Koenigia islandica – West and East Greenland (Fig. 23G).

**Specimens examined or recorded.**

On **Koenigia islandica** L.:

**WG**, Sisimiut (as ‘Holsteinsborg’), 66°56'20"N, 6 Aug 1884, leg. E. Warming & T. Holm, s.n. (holotype, C-F-102468!, as ‘U. koenigiae’; Rostrup 1888, as ‘U. koenigiae’); **ditto**, 14 Jul 1886, leg. K. Rosenvinge, s.n. (C-F-102467!, as ‘U. koenigiae’); **ditto**, sine dat., sine coll. (C-F-102466!, as ‘U. koenigiae’).

**WG**, Kangarsuk near Tindingen, 61°25'N, 1889, leg. N. Hartz, s.n. (C-F-102464!, 102465!, as ‘U. koenigiae’, dupl. in W 1901-0007339!; Rostrup 1891, as ‘U. koenigiae’).

**EG**, Hold with Hope, Myggbukta (on the north side of Mackenzie Bugt), 73°29.5'N, 1 Aug 1930, leg. J. Vaage, s.n. (O!, s.n., as ‘U. koenigiae’; Hagen 1947, as ‘U. koenigiae’); **ditto**, Myggbukta, the damp plain east of the houses, 31 Jul 1933, leg. A. Hagen, the Norwegian Expedition to NE Greenland 1933, s.n. (O!, s.n., as ‘U. koenigiae’; Hagen 1947, as ‘U. koenigiae’).

**EG**, Ymer Island, Kap Humboldt, 73°06'N, 3 Aug 1929, leg. J. Vaage, s.n. (O!, s.n., as ‘U. koenigiae’; Hagen 1947, as ‘U. koenigiae’).

**EG**, Geographical Society Island, Husbukta, 72°51'N, 8 Aug 1929, leg. J. Vaage, s.n. (O!, s.n., as ‘U. koenigiae’; Hagen 1947, as ‘U. koenigiae’).

**Known hosts** — On Polygonaceae: Koenigia islandica, K. pilosa Maxim.

**General distribution. Europe**: Iceland, Fennoscandia. **Asia**: Russian Far East. **North America**: Greenland.

**Earlier reports from Greenland**: Rostrup (1888, 1891), Clinton (1902, 1904, 1906), Hagen (1947), Vánky and Oberwinkler (1994) — as ‘U. koenigiae’, Vánky (2011).

**Comments** — The principal host plant, Koenigia islandica, is a circumboreal–polar species (Hultén and Fries 1986: 643). Microbotryum koenigiae is a circumboreal–polar species, reported on Koenigia islandica from Greenland (Rostrup 1888, 1891; Hagen 1947), Iceland (Helgi Hallgrímsson and Guðríður Gyða Eyjólfsdóttir 2004), Fennoscandia (Norway, Sweden – Norrland, and Kola Peninsula; Liro 1924; Lindeberg 1959; Karatygin et al. 1999; Karatygin 2012), and Russian Far East (northern Kuril Islands; Govorova 1990; Azbukina et al. 1995); and on K. pilosa from China (Guo 2000).

#### 4(25) Microbotryum lagerheimii Denchev, Mycol. Balcanica 4: 64, 2007. — Holotype on Viscaria vulgaris (as ‘Lychnis viscaria subsp. viscaria), Finland, Ab., Kakskerta, Monnonen, 12 Jun 1933, leg. L.E. Kari, s.n. (H s.n.!); isotypes in Fungi Exsicc. Fenn., no. 293 (as ‘Ustilago silenes-inflatae’). Paratypes: Finland, Al., Lemland, Jersö, 13 Jun 1919, leg. T. Putkonen, s.n. (H s.n.!; isoparatypes in Liro, Mycoth. Fenn., no. 405, as ‘U. silenes-inflatae’); Finland, Sat., Huittinen, Raskalanmäki, 30 Jun 1919, leg. W.M. Linnaniemi, s.n. (H s.n.!, as ‘U. silenes-inflatae’); Finland, Ta., Sääksmäki, Maatiala, Urpola, 30 Jul 1918, leg. J.I. Liro, s.n. (H s.n.!, as ‘U. silenes-inflatae’); Germany, Sachsen, Königstein, 16 Jun 1888, leg. W. Krieger (GZU s.n.!; isoparatypes in Krieger, Fungi Saxon., no. 458, as ‘U. violacea’).

Fig. 24A–F

[Ustilago violacea var. pallida Lagerh., in Sydow, Ustilag., no. 65 (as ‘β pallida’) (nom. nud.)]. — ‘Type’ on Viscaria alpina, Norway, Alten, Kåfjord, August 1895, leg. G. Lagerheim (FH! – on the label as ‘U. pallida Lagerh.’); ‘isotypes’ in Sydow, Ustilag., no. 65 (H!, KSC!, M!, NY!).

[Ustilago pallida Lagerh., in Sydow, Ustilag., no. 111, 1897 (nom. nud.)]. — Ustilago pallida Lagerh. ex Bubák, Arch. Přír. Výzk. Čech. 15(3): 22, 1912 (nom. illegit., ICN Art. 53.1); non U. pallida Körn., Hedwigia 16: 34, 1877, q.e. U. cynodontis (Henn.) Henn.; nec U. pallida J. Schröt., in Fischer von Waldheim, Aperçu Syst. Ustilag.: 30, 1877, q.e. Microbotryum anomalum (J. Kunze ex G. Winter) Vánky). — Type on Viscaria vulgaris, Sweden, Öland, Borgholm, July 1896, leg. G. Lagerheim, s.n.; isotypes in Sydow, Ustilag., no. 111 (KSC!, M!, S!).

**Infection** systemic. **Sori** in the considerably swollen anthers, filling the pollen sacs with a pulverulent, fawn spore mass. **Spores** globose, subglobose, broadly ellipsoidal or ovoid, (5.5–)6–8.5(–9.5) × (5–)5.5–8(–8.5) (7.4 ± 0.6 × 6.8 ± 0.5) µm (n/_3_ = 300), subhyaline with vinaceous tint; wall reticulate, 0.9–1.4 µm thick (including reticulum), meshes 5–8(–9) per spore diameter, polyhedral or irregular, 0.3–1.2(–1.5) µm wide, muri up to 0.4 µm high. In SEM meshes smooth or rugulose on the bottom.

**Figure 24.**
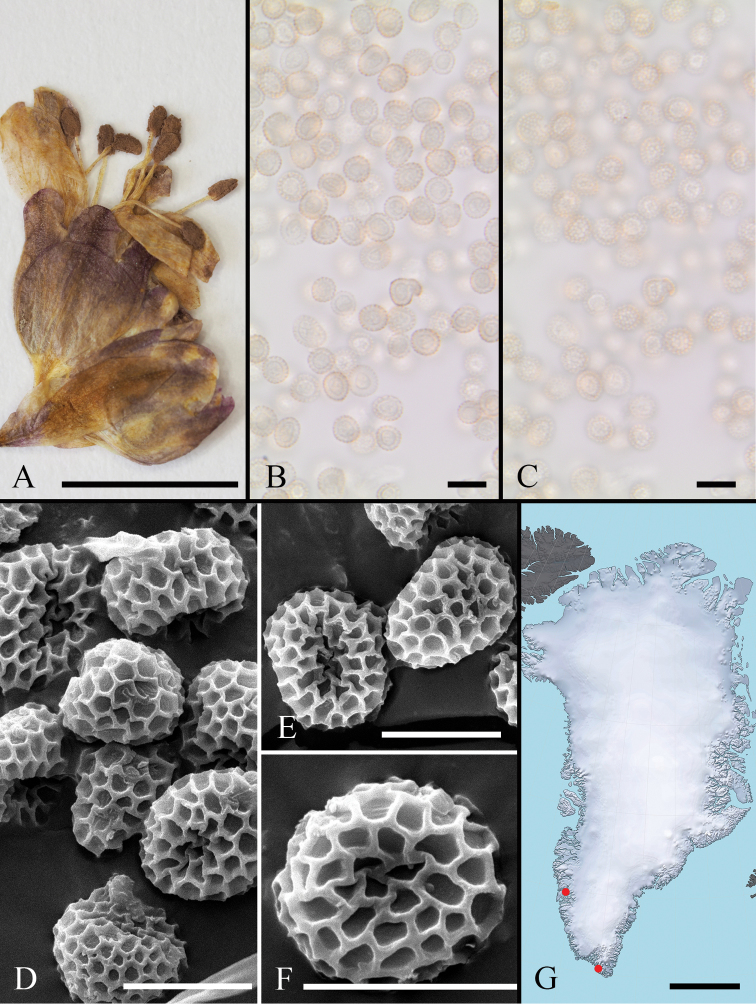
Microbotryum lagerheimii on Viscaria alpina. **A** Habit (leg. I. Hauge, s.n., C-Greenland herb., s.n.) **B, C** spores in LM, in median and surface view, respectively (leg. P.M. Hansen, s.n., C-Greenland herb., s.n.) **D–F** spores in SEM (**D** leg. I. Hauge, s.n., C-Greenland herb., s.n.; **E** leg. P.M. Hansen, s.n., C-Greenland herb., s.n.; **F** K. Gormsen, no. A. 16, C-Greenland herb., s.n.) **G** distribution map. Scale bars: 0.5 cm (**A**); 10 µm (**B, C**); 5 µm (**D–F**); 500 km (**G**).

**Hosts and distribution within the studied area** — On Caryophyllaceae: Viscaria alpina – West Greenland (Fig. 24G).

**Specimens examined or recorded.**

On **Viscaria alpina** (L.) G. Don:

**WG**, Godthåbsfjord, Komak, 6 Jul 1927, leg. P.M. Hansen, s.n. (C-Greenland herb.!, s.n.); **ditto**, Narssarssuaq, 64°49'N, 51°00'W, alt. ca 25 m, 31 Jul 1987, leg. I. Hauge, s.n. (C-Greenland herb.!, s.n.).

**WG**, Amitsoq, 60°20'N, 45°02'W, alt. 300 m, 11 Aug 1963, leg. K. Gormsen, no. A. 16 (C-Greenland herb.!, s.n.).

**Known hosts** — On Caryophyllaceae: Atocion rupestre (L.) Oxelman (Silene rupestris L.), Silene uniflora Roth, S. vulgaris (Moench) Garcke, Viscaria alpina (Lychnis alpina L., Silene suecica (Lodd.) Greuter & Burdet), V. vulgaris Bernh. subsp. vulgaris (Lychnis viscaria L. subsp. viscaria), V. alpina × V. vulgaris (Lychnis alpina × L. vulgaris).

**General distribution. Europe**: UK, Norway, Sweden, Finland, Russia (Arctic and Karelia), Denmark, Latvia, France, Germany, Poland, Switzerland, Austria, Czech Republic, Italy. **North America**: Greenland.

**Comments** — Viscaria vulgaris and V. alpina are principal hosts of M. lagerheimii that is also known on Atocion rupestre, Silene vulgaris, and S. uniflora (Denchev 2007a; Hood et al. 2010; Abbate et al. 2018). Silene vulgaris is a host for three anthericolous Microbotryum species: one with verruculose-incompletely reticulate spores, M. violaceoirregulare (Brandenb. & Schwinn) G. Deml & Oberw., and two species with reticulate spores, M. silenes-inflatae (DC. ex Liro) G. Deml & Oberw. and M. lagerheimii (Denchev 1994, 2007a; Zwetko and Blanz 2004; Denchev and Minter 2008; Abbate et al. 2018). Additionally, it is known that these species occur at different elevations: the localities of M. violaceoirregulare are at high elevations while that of M. silenes-inflatae and M. lagerheimii are at lower elevations (Abbate et al. 2018).

The earlier circumscription of M. silenes-inflatae (initially as ‘Ustilago silenes-inflatae’) included Viscaria species as hosts, based on the artificial infection experiment made by Liro (1924). Liro successfully carried out artificial infection of Silene vulgaris with light-colored spores of Ustilago (‘U. pallida’) from Viscaria vulgaris and concluded that V. vulgaris and V. alpina were additional hosts of Ustilago silenes-inflatae. This taxonomic proposition was accepted by many mycologists for a long time (e.g. Deml and Oberwinkler 1982, 1983; Scholz and Scholz 1988; Vánky 1994, 1998, 2011). The status of Microbotryum species with dark and light colored spore masses on Silene vulgaris, Viscaria vulgaris, and V. alpina was, however, reviewed by Denchev (2007a), who emended the circumscription of M. silenes-inflatae and described M. lagerheimii. Microbotryum lagerheimii differs from M. silenes-inflatae by having a spore mass of medium or low color intensity (fawn, hazel, livid vinaceous, salmon, flesh, pale vinaceous or rosy vinaceous) and subhyaline to pale colored spores versus spore mass of high color intensity (dark brick, sepia, dark livid, dark vinaceous, rarely dark purple or purple slate) and darker spores for M. silenes-inflatae on Silene vulgaris (Denchev 2007a). Later, the distinctness of M. lagerheimii was confirmed with molecular methods (Devier et al. 2009, 2010; Hood et al. 2010; Piątek et al. 2012; Abbate et al. 2018).

The plants of Viscaria alpina in Greenland and northeastern North America, possessing coarser and broader cauline leaves than the plants in North and Central Europe, were considered by some authors (e.g., Böcher 1963; Feilberg 1984) to fall into a distinct subspecies, V. alpina subsp. americana (Fernald) Böcher (or Lychnis alpina subsp. americana (Fernald) J. Feilberg), but according to Morton (2005), Aiken et al. (2007), and Elven et al. (2018), subsp. americana does not merit recognition.

Microbotryum lagerheimii is reported here for the first time from Greenland. It is an amphi-Atlantic–European species.

#### 5(26) Microbotryum pustulatum (DC.) R. Bauer & Oberw., in Bauer, Oberwinkler, and Vánky, Canad. J. Bot. 75: 1309, 1997. ≡ Uredo bistortarum var. pustulata DC., Fl. Franç., ed. 3 (Paris) 6: 76, 1815 (as ‘α pustulata’). ≡ Ustilago pustulata (DC.) G. Winter, Hedwigia 19: 109, 1880. ≡ Ustilago pustulata (DC.) Bubák, Arch. Naturwiss. Landesdurchf. Böhmen 15: 17, 1916 (comb. superfl.). ≡ Ustilago bistortarum var. pustulata (DC.) B. Lindeb., Symb. Bot. Upsal. 16(2): 111 1959. — Lectotype on Bistorta major, France, the Alps, leg. A.P. de Candolle (design. by Lindeberg 1959: 111).

Fig. 25A–F

= Tilletia bullata Fuckel, Jahrb. Nassauischen Vereins Naturk. 23–24: 40 1870. — Type on Bistorta vivipara, Austria, Tirol, leg. C.F.P. v. Martius.

= Ustilago bullata var. glabra Rostr., Bot. Tidsskr. 15: 229, 1886. ≡ Ustilago bistortarum var. glabra (Rostr.) de Toni, in Saccardo, Syll. Fung. 7: 469[bis], 1888. — Holotype on Bistorta vivipara, Norway, Troms, Tromsø, 1885, leg. E. Warming, s.n. (C).

**Infection** local. Sori in leaves as yellowish brown to dark vinaceous, round, blisterlike pustules, 1–4 mm in diam., often larger by fusion, scattered or often arranged in two rows along the median vein, initially covered by the epidermis which later ruptures, disclosing a semi-agglutinated to powdery, dark reddish brown mass of spores. **Spores** subglobose, broadly ellipsoidal, slightly irregular, ovoid, globose or ellipsoidal, (12–)13–18(–20) × (11–)12–16(–17) (15.3 ± 1.3 × 13.5 ± 0.9) µm (n/_3_ = 300), medium vinaceous; wall 0.7–1.3 µm thick, moderately verruculose, warts up to 0.4(–0.5) µm high, spore profile affected. In SEM warts usually isolated, sometimes confluent in short rows or small groups. **Spore germination** (after Liro 1924: 181) of Ustilago-type.

**Figure 25.**
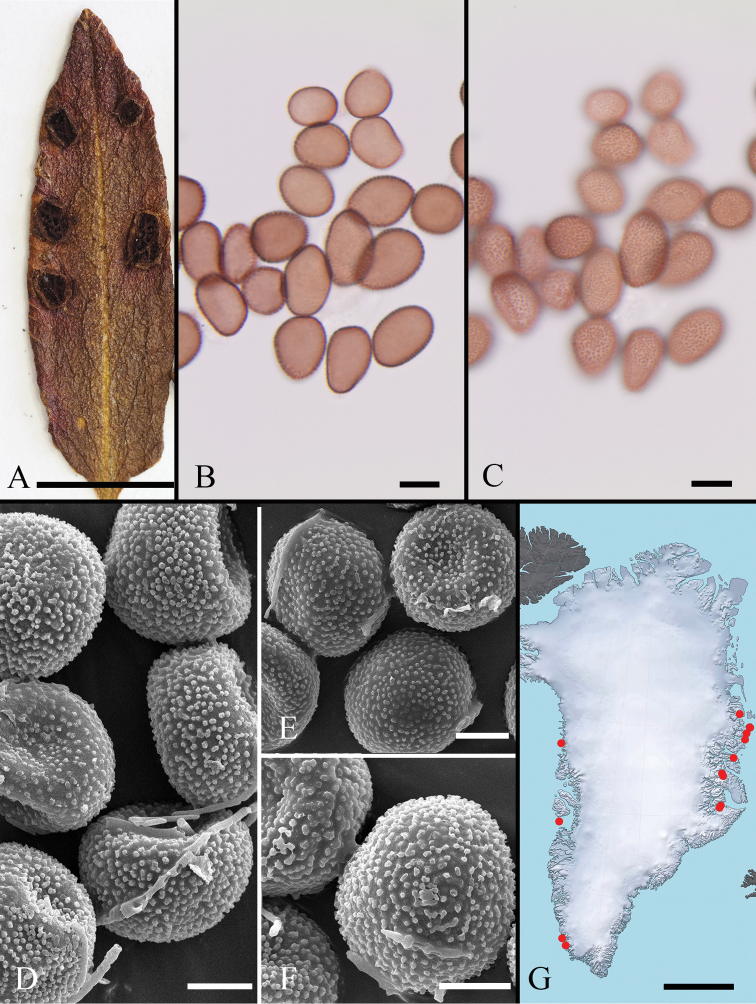
Microbotryum pustulatum on Bistorta vivipara. **A** Habit (3 Aug 1933, leg. A. Hagen, s.n., O, s.n.) **B, C** spores in LM, in median and surface view, respectively (3 Aug 1933, leg. A. Hagen, s.n., O, s.n.) **D–F** spores in SEM (C-F-102474) **G** distribution map. Scale bars: 0.5 cm (**A**); 10 µm (**B, C**); 5 µm (**D–F**); 500 km (**G**).

**Hosts and distribution within the studied area** — On Polygonaceae: Bistorta vivipara – West and East Greenland (Fig. 25G).

**Specimens examined or recorded.**

On **Bistorta vivipara** (L.) Delarbre:

**WG**, Tasiusaq, 73°22'N, 1884, leg. E. Warming & Th. Holm, s.n. (n.v.; not found in C; Rostrup 1888, as ‘*U. bistortarum*’).

**WG**, Kronprinsens Islands (S of Disko Island), ca 69°00'N, 13 Jul 1897, leg. C. Krusse, no. 265 (C-F-102472!, as ‘*U. bistortarum*’).

**WG**, Paamiut (as ‘Frederikshåb’), 1889, leg. N. Hartz, s.n. (C-F-102474!, as ‘*U. bistortarum*’; Rostrup 1891, as ‘*U. bistortarum*’).

**WG**, Kingua Neriak, 61°35'N, 1889, leg. N. Hartz, s.n. (C-F-102473!, as ‘*U. bistortarum*’; Rostrup 1891, as ‘*U. bistortarum*’).

**EG**, Dronning Margrethe II Land, Hochstetter Forland, Jónsbú (NE of the mouth of Ardencaple Fjord), ca 75°19.2'N, 20°23.3'W, 3 Aug 1933, leg. A. Hagen, the Norwegian Expedition to NE Greenland 1933, s.n. (O!, s.n., as ‘*U. bistortarum*’; Hagen 1947, as ‘*U. bistortarum*’).

**EG**, Sabine Island, Germania Havn (on the south side of the island), ca 74°32.2'N, 18°49.9'W, 21 Jul 1933, leg. A. Hagen, the Norwegian Expedition to NE Greenland 1933, s.n. (O!, s.n., as ‘*U. bistortarum*’; Hagen 1947, as ‘*U. bistortarum*’).

**EG**, Hvalrosø Island (as ‘Kvalrossoya’), ca 74°30'N, 21 Jul 1933, leg. A. Hagen, the Norwegian Expedition to NE Greenland 1933, s.n. (O!, s.n., as ‘*U. bistortarum*’; Hagen 1947, as ‘*U. bistortarum*’).

**EG**, Wollaston Forland, near Herschellhus (Kap Herschel), ca 74°14.6'N, 19°41'W, 30 Jul 1929, leg. J. Vaage, s.n. (O!, s.n., Hagen 1947, as ‘*U. bistortarum*’).

**EG**, Gael Hamke Bugt, Jackson Island (ca 73°55'N), ‘hundegården’, 11 Aug 1933, leg. A. Hagen, the Norwegian Expedition to NE Greenland 1933, s.n. (O!, s.n., as ‘*U. bistortarum*’; Hagen 1947, as ‘*U. bistortarum*’); **ditto**, Jackson Island, route 3, 12 Aug 1933, leg. A. Hagen, the Norwegian Expedition to NE Greenland 1933, s.n. (O!, s.n., as ‘*U. bistortarum*’; Hagen 1947, as ‘*U. bistortarum*’).

**EG**, Geographical Society Island, Sofia Sund, near Strømhytta, ca 73°02'N, 22°55'W, 21 Aug 1933, leg. A. Hagen, the Norwegian Expedition to NE Greenland 1933, s.n. (O!, s.n., two specimens as ‘*U. bistortarum*’; Hagen 1947, as ‘*U. bistortarum*’).

**EG**, East side of Alpefjord, Stauning Alper, ca 72°15'N, 28 Jul 1933, leg. A. Hagen, the Norwegian Expedition to NE Greenland 1933, s.n. (O!, s.n., as ‘*U. bistortarum*’; Hagen 1947, as ‘*U. bistortarum*’); **ditto**, N of Gullygletscher (as ‘Gullybreen’), ca 72°06.3'N, 27 Jul 1933, leg. A. Hagen, the Norwegian Expedition to NE Greenland 1933, s.n. (O!, s.n., as ‘*U. bistortarum*’; Hagen 1947, as ‘*U. bistortarum*’).

**EG**, Danmark Island, Hekla Havn, ca 70°26.9'N, 26°14.7'W, August 1891, leg. N. Hartz, C. Ryder’s Expedition to East Greenland in 1891–1892, s.n. (C-F-102470!, ‘*U. bistortarum*’; Rostrup 1894, as ‘*U. bistortarum*’).

**EG**, Gåseland, 10 Aug 1891, leg. N. Hartz, C. Ryder’s Expedition to East Greenland in 1891–1892, s.n. (C-F-102471!, as ‘*U. bistortarum*’; Rostrup 1894, as ‘*U. bistortarum*’).

**Known hosts** — On Polygonaceae: Bistorta elliptica (Willd. ex Spreng.) D.F. Murray & Elven (P. ellipticum Willd. ex Spreng., P. nitens (Fisch. & C.A. Mey.) Petrov ex Kom.), B. major Gray subsp. major (P. bistorta L.), B. major subsp. carnea Soják (P. carneumC. Koch), B. vivipara (P. viviparum L.).

**General distribution. Europe. Asia. North America**: Canada, Greenland, U.S.A.

**Earlier reports from Greenland**: Rostrup (1888, 1891, 1894), Clinton (1902, 1904, 1906), Lind (1927), Hagen (1947) — all records as ‘*U. bistortarum*’.

#### 6(27) Microbotryumsilenes-acaulis M. Lutz, Piątek & Kemler, in Lutz et al., Mycol. Res. 112: 1289, 2008. — Holotype on Silene acaulis, Poland, Tatra Mts, 300 m N of the top of Kopa Kondracka Mt., alt. 1925 m, 25 Jun 2005, leg. M. Piątek & J. Cabała, s.n. (KRAM F55485).

Fig. 26A–F

**Infection** systemic. **Sori** in the considerably swollen anthers, filling the pollen sacs with a pulverulent, vinaceous spore mass. **Spores** subglobose, globose, broadly ellipsoidal or ovoid, sometimes ellipsoidal, (5.5–)6–9(–9.5) × (5–)5.5–7.5(–8.5) (7.1 ± 0.7 × 6.4 ± 0.5) µm (n/_3_ = 300), light vinaceous; wall reticulate, 0.9–1.4 µm thick (including reticulum), meshes (4–)5–8(–9) per spore diameter, polyhedral or irregular, 0.3–1.6(–2.0) µm wide, muri up to 0.4 µm high. In SEM meshes smooth or rugulose on the bottom, sometimes with a hemispherical protuberance. **Spore germination** (after Lutz et al. 2008: 1289) results in 3–4-celled basidia, 12–18 × 2.5–3.5 µm, producing ovoid basidiospores.

**Figure 26.**
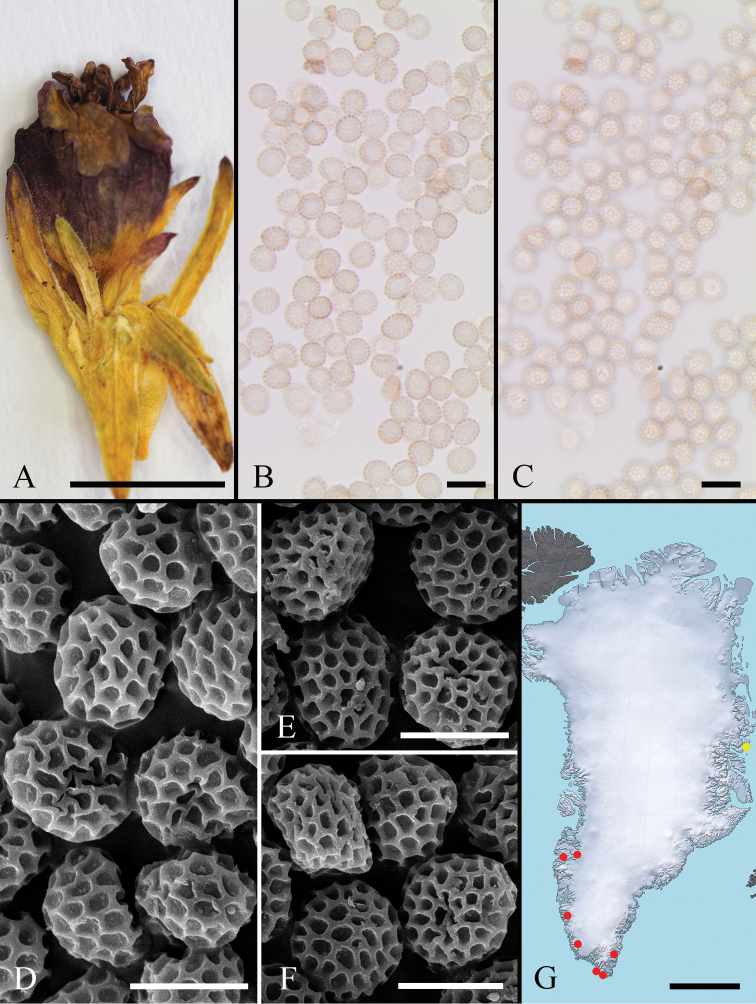
Microbotryum silenes-acaulis on Silene acaulis. **A** Habit (N. Jacobsen, no. G.B.U. 1717, C-Greenland herb., s.n.) **B, C** spores in LM, in median and surface view, respectively (H. Andersen & J. Feilberg, no. G.B.U. 4775, C-Greenland herb., s.n.) **D–F** spores in SEM (**D**C. Bay et al., no. G.B.U. 78-1571, C-Greenland herb., s.n.; **E, F** S. Holt, no. 1308, C-Greenland herb., s.n.) **G** distribution map. Scale bars: 0.5 cm (**A**); 10 µm (**B, C**); 5 µm (**D–F**); 500 km (**G**).

**Hosts and distribution within the studied area** — On Caryophyllaceae: Silene acaulis – West and East Greenland (Fig. 26G).

**Specimens examined or recorded.**

On **Silene acaulis** (L.) Jacq.:

**WG**, head of Søndre Strømfjord, SE of Isunguata Sermia, 67°11'N, 50°17'W, alt. 400 m, 22 Jul 1978, leg. S. Holt, no. 1308 (C-Greenland herb.!, s.n.).

**WG**, Ikertôq, E of Akuliaruseq, 66°53'N, 52°19'W, alt. ca 500 m, 24 Jul 1978, leg. C. Bay et al., no. G.B.U. 78-1571 (C-Greenland herb.!, s.n.).

**WG**, Head of Grædefjord, 63°23'N, 50°10'W, 1 Aug 1972, leg. H. Andersen & J. Feilberg, no. G.B.U. 4775 (C-Greenland herb.!, s.n.).

**WG**, 610 m lake, 61°43'N, 48°08'W, 22 Jul 1965, leg. J. Johansen et al., no. 65-2722 (C-Greenland herb.!, s.n.).

**WG**, Nanortalik, 60°09'N, 45°15'W, 28 Jun 1964, leg. C. Hansen et al., no. 64-1034 (C-Greenland herb.!, s.n.).

**WG**, Pamiagdluk, entrance of Tasiussaq, 59°57'N, 44°21'W, 17 Jul 1970, leg. N. Jacobsen, Kap Farvel Expeditionen 1970, no. G.B.U. 1717 (C-Greenland herb.!, s.n.).

**EG**, ‘Eirik Raudes Land’ (area bounded between the latitudes 71°30'–75°40'N), leg. A. Hagen (n.v.; 27 specimens recorded by Hagen 1947, as ‘U. violacea’, but not found in the herbarium in Oslo).

**EG**, Lindenow Fjord, Møretun, ca 60°28'N, 43°18'W, 3 Aug 1932, leg. J. Devold & P.F. Scholander, s.n. (n.v.; not found in O; Hagen 1947, as ‘U. violacea’).

**Known hosts** — On Caryophyllaceae: Silene acaulis.

**General distribution. Europe**: Svalbard, Iceland, UK, Norway, Sweden, Russia (Murmansk Region, Novaya Zemlya), France, Germany, Poland, Switzerland, Austria, Slovakia, Romania, Italy, Bulgaria. **Asia**: Russia (Far East). **North America**: Alaska, Canada, Greenland, U.S.A. (the Cordillera, northeastern U.S.A.).

**Comments** — Silene acaulis is a circumpolar–alpine species, with a large Siberian disjunction in the distribution (Hultén and Fries 1986: 791). In the *Flora of North America* treatment (Morton 2005), it was considered as a variable species but without recognition of infraspecific taxa. In Hultén and Fries (1986) and in *Panarctic Flora* (Elven et al. 2018), however, two subspecies are recognized within S. acaulis: subsp. acaulis, a North American (northeastern)–amphi-Atlantic–European–Asian (northwestern) taxon, and subsp. subacaulescens (F.N. Williams) Hultén, an amphi-Beringian–Cordilleran taxon (distributed in the Russian Far East, Aleutian Islands, and the Cordillera — extending down from Alaska to Arizona and New Mexico). In Europe, S. acaulis is a polar–alpine species with occurrences in the Arctic, Subarctic, Ural, and the higher mountains.

On the amphi-Beringian–Cordilleran entity, Microbotryum silenes-acaulis is recorded from the Russian Far East (northern Kuril Islands and Commander Islands), Alaska, and the Cordillera (Govorova 1990, as ‘U. violacea’; Bueker et al. 2016). On subsp. acaulis, M. silenes-acaulis follows its host in the Canadian Arctic Archipelago, northeastern U.S.A., Greenland, the European Arctic and Subarctic, as well as in the refugia of this plant in Central and South Europe: in the Alps, Pyrenees, Tatra Mts, Carpathian Mts, and Rila Mts (Lind 1928; Hagen 1941, 1950b; Vánky 1985a; Scholz and Scholz 1988; Karatygin et al. 1999; Zwetko and Blanz 2004 — as ‘U. violacea’; Lutz et al. 2008; Denchev et al. 2011; Karatygin 2012; Bueker et al. 2016; Smith et al. 2017).

#### 7(28) Microbotryum stellariae (Sowerby) G. Deml & Oberw., Phytopathol. Z. 104: 354, 1982. ≡ Farinaria stellariae Sowerby, Coloured Figures of English Fungi 3(no. 27): Pl. 396, fig. 1, 1803. ≡ Ustilago stellariae (Sowerby) Liro, Ann. Acad. Sci. Fenn., Ser. A 17(1): 39, 1924. ≡ Ustilago violacea var. stellariae (Sowerby) Savile, Canad. J. Bot. 31: 674, 1953. — Host plants indicated by Sowerby (1803): Stellaria graminea and S. holostea. The generic name Farinaria, and accordingly the name F. stellariae, were erroneously considered by Vánky (1998: 51) as invalidly published. The lectotype of M. stellariae, as it is designated by Vánky (1998: 52), is referred to Ustilago stellariae ‘Liro’: ‘on Stellaria graminea, Germany, Baden-Württemberg, Freiburg i/B., near Littenweiler, July 1876, leg. J. Schröter, s.n., H, s.n.)’.

Figs 1C, 27A–F

**Infection** systemic. **Sori** in the considerably swollen anthers, filling the pollen sacs with a pulverulent, dark reddish brown spore mass. **Spores** subglobose, globose, broadly ellipsoidal, ovoid, ellipsoidal or slightly irregular, 5–8.5(–9.5) × (4.5–)5–7(–8) (6.8 ± 0.6 × 6.0 ± 0.5) μm (n/_3_ = 300), light vinaceous; wall reticulate, 0.9–1.3 μm thick (including reticulum), meshes (5–)6–9(–10) per spore diameter, polyhedral or irregular, 0.3–1.4(–2.0) μm wide, muri up to 0.4 µm high. In SEM meshes smooth or rugulose on the bottom.

**Figure 27.**
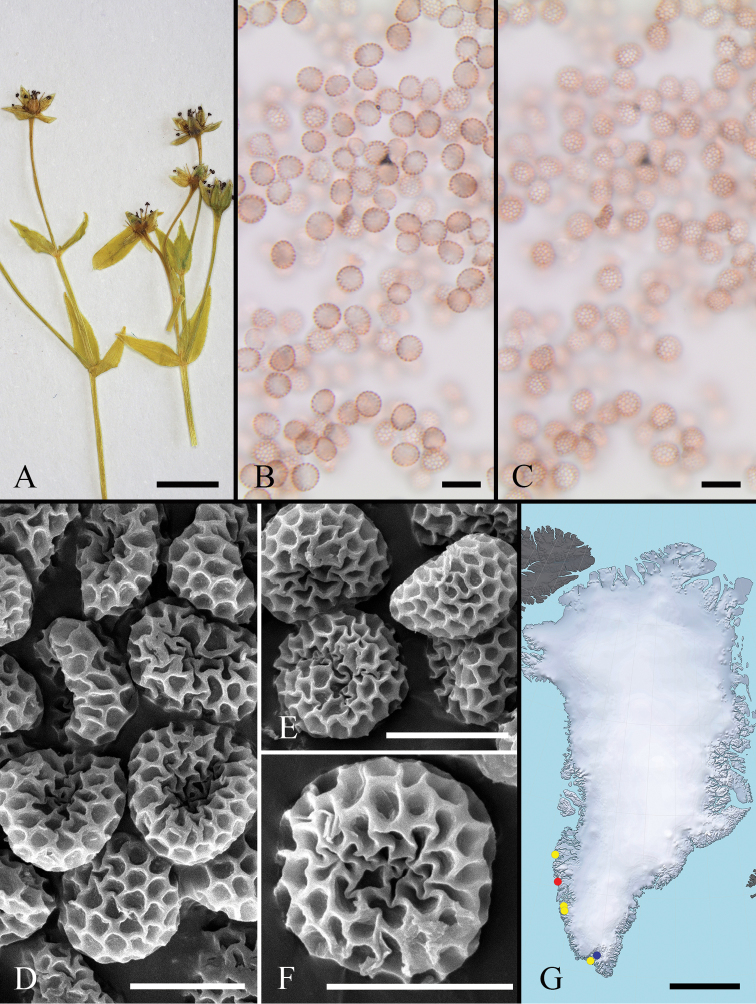
Microbotryum stellariae on Stellaria calycantha. **A** Habit (J. Feilberg, no. G.B.U. 5570, C-Greenland herb., s.n.) **B, C** spores in LM, in median and surface view, respectively (J. Feilberg, no. G.B.U. 5570, C-Greenland herb., s.n.) **D–F** spores in SEM (**D, F** J. Feilberg, no. G.B.U. 5570, C-Greenland herb., s.n.; **E** H. Andersen & J. Feilberg, no. G.B.U. 4424, C-Greenland herb., s.n.) **G** distribution map (red circle – on Stellaria borealis subsp. borealis; yellow circles – on S. calycantha; blue circle – on S. crassipes). Scale bars: 0.5 cm (**A**); 10 µm (**B, C**); 5 µm (**D–F**); 500 km (**G**).

**Hosts and distribution within the studied area** — On Caryophyllaceae: Stellaria borealis subsp. borealis (Alsine borealis (Bigelow) Britton) – West Greenland; S. calycantha – West Greenland; S. crassipes – West Greenland (Fig. 27G).

**Specimens examined or recorded.**

On **Stellaria borealis** Bigelow subsp. **borealis**:

**WG**, Søndre Isortoq, 65°20'N, 1888, leg. K. Rosenvinge, s.n. (n.v.; not found in C; Rostrup 1891, as ‘U. violacea’).

On **Stellaria calycantha** (Ledeb.) Bong.:

**WG**, Sisimiut, valley behind the dump, 66°55'48"N, 53°38'24"W, 18 Aug 2016, leg. H. Knudsen, no. HK 16.196 (C-F-108447!).

**WG**, Qingua, Buksefjorden, 63°56'N, 50°55'W, alt. 10 m, 16 Aug 1973, leg. J. Feilberg, no. G.B.U. 5570 (C-Greenland herb.!, s.n.).

**WG**, Sioralik, Alàngordlia, 63°39'N, 50°42'W, 7 Aug 1972, leg. H. Andersen & J. Feilberg, no. G.B.U. 4424 (C-Greenland herb.!, s.n.).

**WG**, Nupiluk, 60°46'N, 46°10'W, alt. 100 m, 21 Jul 1962, leg. C. Hansen et al., Plantae Vasculares Groenlandicae Exsiccatae, no. 200 (SOM 108468!).

On **Stellaria crassipes** Hultén:

**WG**, Narsarsuaq, 61°10'N, 45°25'W, 9 Aug 1984, leg. T. Læssøe, no. TL 84.463 (C-F-107981!); **ditto**, 11 Aug 1984, leg. T. Læssøe, no. TL 84.617 (C-F-107986!).

**Known hosts** — On Caryophyllaceae: Arenaria spp., Cerastium spp., Moehringia lateriflora (L.) Fenzl (Arenaria lateriflora L.), Myosoton aquaticum (L.) Moench (Cerastium aquaticum L.), Stellaria spp.

**General distribution. Europe. Asia. North America**: Canada, Greenland, U.S.A.

**Comments** — The circumscription of some Stellaria species distributed in the Arctic is not satisfactorily resolved (see the comments to Stellaria in Elven et al. 2018). In the current treatment, the host plants of Microbotryum stellariae are listed as they are identified in the respective phanerogam herbaria.

#### 8(29) Microbotryum vinosum (Tul. & C. Tul.) Denchev, Mycotaxon 50: 331, 1994. ≡ Ustilago vinosa Tul. & C. Tul., Ann. Sci. Nat., Bot., Sér. 3, 7: 96, 1847. ≡ Microbotryum vinosum (Tul. & C. Tul.) G. Deml & Prillinger, in Prillinger, Deml, Dörfelt, Laaser, and Lockau, Bot. Acta 104: 10, 1991 (nom. inval., no reference to the basionym; ICN Art. 41.1). — Holotype on Oxyria digyna (as ‘*O. reniformis*’), Great Britain, Scotland, Angus (as ‘Forfarshire’), leg. W. Gardiner, s.n.

Figs 1D, 28A–F

[Uredo vinosa Berk., in litt. ad Tulasne (nom. nud.)].

**Infection** systemic, all flowers of an inflorescence affected. **Sori** in the four perianth-segments of each flower swelling them considerably and filling them with pulverulent, vinaceous spore mass. Ovaries and anthers remain intact. **Spores** globose, subglobose, ovoid or broadly ellipsoidal, sometimes ellipsoidal, 6.5–9.5(–10.5) × (5.5–)6.5–8(–9) (8.0 ± 0.7 × 7.1 ± 0.5) µm (n/_3_ = 300), light vinaceous; wall reticulate, 0.9–1.4 µm thick (including reticulum), meshes (5–)6–9(–10) per spore diameter, polyhedral or irregular, 0.4–1.7(–2.0) µm wide, muri up to 0.5 µm high. In SEM meshes rugulose on the bottom. **Spore germination** (after Brefeld 1895: 134, Pl. 8, Figs 13–15; Ingold 1983: 577, Fig. 4) results in 4-celled basidia (often in 3 + 1 pattern) producing lateral and terminal basidiospores, which fuse two by two giving rise to hyphae.

**Figure 28.**
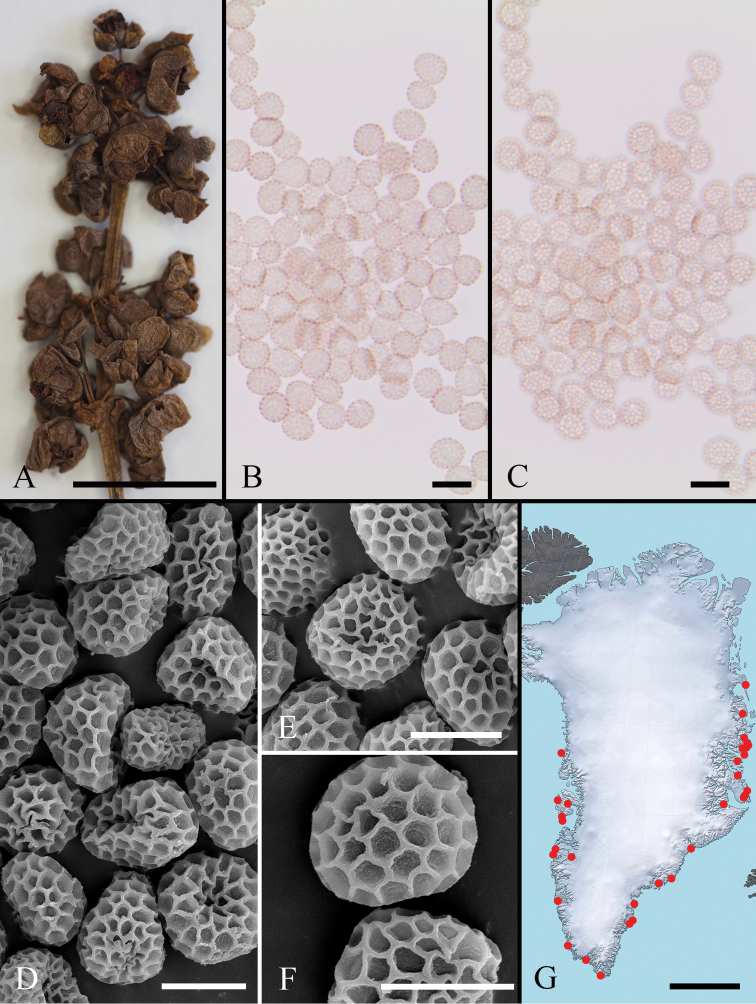
Microbotryum vinosum on Oxyria digyna. **A** Habit (B. Fredskild, no. 5113, C-Greenland herb., s.n.) **B, C** spores in LM, in median and surface view, respectively (M. Møller et al., no. 261, C-Greenland herb., s.n.) **D–F** spores in SEM (B. Fredskild, no. 5113, C-Greenland herb., s.n.) **G** distribution map. Scale bars: 0.5 cm (**A**); 10 µm (**B, C**); 5 µm (**D–F**); 500 km (**G**).

**Hosts and distribution within the studied area** — On Polygonaceae: Oxyria digyna – West and East Greenland (Fig. 28G).

**Specimens examined or recorded.**

On **Oxyria digyna** (L.) Hill:

**WG**, Upernavik, Smedeøen, ca 72°47'N, 56°08'W, 30 Jul 1931, leg. F. Johansen, s.n. (C-F-107995!, as ‘*U. vinosa*’).

**WG**, Disko Island, Kingigtok near Vajgattet, alt. 1600 ft, 70°08'N, Aug 1890, leg. N. Hartz, s.n. (C-F-102451!, 102452!, as ‘*U. vinosa*’; Rostrup 1891, as ‘*U. vinosa*’); **ditto**, Kutdlisat, 70°03'N, 1889, leg. N. Hartz, s.n. (n.v.; not found in C; Rostrup 1891, as ‘*U. vinosa*’); **ditto**, Qeqertarsuaq (as ‘Godhavn’), 69°15'N, leg. A. Dahl, s.n. (C-F-102461!, as ‘*U. vinosa*’).

**WG**, Kronprinsens Islands (S of Disko Island), Imerigsoq, 69°01'N, 53°18–20'W, 31 Jul 1980, leg. M. Møller et al., no. 261 (C-Greenland herb.!, s.n.).

**WG**, North Isortok Fjord, 67°10'N, 1 Aug 1886, leg. K. Rosenvinge, s.n. (C-F-102454!, as ‘*U. vinosa*’; Rostrup 1888, as ‘*U. vinosa*’).

**WG**, South Kangerdluarsuk, 67°00'N, 5 Aug 1884, leg. E. Warming & Th. Holm, s.n. (C-F-102453!, as ‘*U. vinosa*’; Rostrup 1888, as ‘*U. vinosa*’).

**WG**, Sisimiut, near the airport, 66°55'48"N, 53°38'24"W, 17 Aug 2016, leg. H. Knudsen, no. HK 16.180d (C-F-108423!, as ‘*U. vinosa*’).

**WG**, Godthåbsfjord, Kuaninguit, 64°12'N, 51°35'W, alt. 100 m, 29 Jul 1979, leg. J. Feilberg, no. 2072 (C-Greenland herb.!, s.n.).

**WG**, Smallesund, 61°32'N, 1888, leg. K. Rosenvinge, s.n. (n.v.; not found in C; Rostrup 1891, as ‘*U. vinosa*’).

**WG**, Tugtutôq Island, central part, 60°48'N, 46°30'W, 27 Jul 1963, leg. C. Hansen & K. Jakobsen, no. F.1737 (C-Greenland herb.!, s.n.).

**WG**, Tornarssuk, 59°55'N, 44°22'W, 13 Aug 1970, leg. B. Fredskild, no. 5113 (C-Greenland herb.!, s.n.).

**EG**, Basiskæret, 76°46'N, 18°39'W, 31 Aug 1907, leg. A. Lundager, “Danmark” Expeditionen 1906–1908, s.n. (C-F-102450!, 102463!, as ‘*U. vinosa*’); **ditto**, leg. A. Lundager, “Danmark” Expeditionen 1906–1908, no. 744 (C-F-107992!, as ‘*U. vinosa*’).

**EG**, ‘Eirik Raudes Land’ (area bounded between the latitudes 71°30'–75°40'N), leg. A. Hagen (n.v.; specimens from 12 localities, recorded by Hagen 1947, as ‘*U. vinosa*’, but not found in the herbarium in Oslo).

**EG**, Dronning Margrethe II Land, Hochstetter Forland, Jónsbú (NE of the mouth of Ardencaple Fjord), 75°19.2'N, 20°23.3'W, 3 Aug 1933, leg. A. Hagen, the Norwegian Expedition to NE Greenland 1933, s.n. (O!, s.n., two specimens, as ‘*U. vinosa*’).

**EG**, Vesle Finsch Island, ca 74°00'N, 20 Jul 1933, leg. A. Hagen, the Norwegian Expedition to NE Greenland 1933, s.n. (O!, s.n., as ‘*U. vinosa*’).

**EG**, Hold with Hope, sine dat., leg. N. Hartz, Expeditio Danica in Groenlandiam orientalem 1891–1892, s.n. (C-F-102460!, as ‘*U. vinosa*’; Rostrup 1894, as ‘*U. vinosa*’); **ditto**, Knudshoved, ca 73°43.9'N, 20°27'W, 15 Aug 1933, leg. A. Hagen, the Norwegian Expedition to NE Greenland 1933, s.n. (O!, s.n., as ‘*U. vinosa*’); **ditto**, Grytvika, ca 73°43.5'N, 20°29.6'W, 18 Aug 1933, leg. A. Hagen, the Norwegian Expedition to NE Greenland 1933, s.n. (O!, s.n., as ‘*U. vinosa*’); **ditto**, Myggbukta (on the north side of Mackenzie Bugt), ca 73°29.4'N, 21°33.4'W, 19 Aug 1933, leg. A. Hagen, the Norwegian Expedition to NE Greenland 1933, s.n. (O!, s.n., as ‘*U. vinosa*’); **ditto**, Stormdalen, ca 73°29.5'N, 20°46.9'W, 9 Aug 1933, leg. A. Hagen, the Norwegian Expedition to NE Greenland 1933, s.n. (O!, s.n., as ‘*U. vinosa*’).

**EG**, Holland Island, 73°36'N, 20°21'W, 13 Aug 1933, leg. A. Hagen, the Norwegian Expedition to NE Greenland 1933, s.n. (O!, s.n., as ‘*U. vinosa*’).

**EG**, Bontekoe Island in Foster Bugt, ca 73°08'N, 23 Jul 1933, leg. A. Hagen, the Norwegian Expedition to NE Greenland 1933, s.n. (O!, five specimens, s.n., as ‘*U. vinosa*’).

**EG**, Geographical Society Island, Husbukta, ca 72°49.7'N, 22°52.5'W, 9 Aug 1929, leg. J. Vaage (O!, s.n.).

**EG**, Scoresby Land, Antarctic Havn (as ‘Antarctic hamna’), 72°01'N, 25 Jul 1933, leg. A. Hagen, the Norwegian Expedition to NE Greenland 1933, s.n. (O!, s.n., as ‘*U. vinosa*’); **ditto**, 26 Jul 1933, leg. A. Hagen, the Norwegian Expedition to NE Greenland 1933, s.n. (O!, s.n., as ‘*U. vinosa*’).

**EG**, Jameson Land, alt. ca 490 m, 5 Aug 1891, leg. N. Hartz, Expeditio Danica in Groenlandiam orientalem 1891–1892, s.n. (C-F-102459!, as ‘*U. vinosa*’; Rostrup 1894, as ‘*U. vinosa*’).

**EG**, Liverpool Land, the east coast, Kangertivit Anginersaat (as ‘Storefjord’), ca 71°05'N, July 1933, leg. A. Noe-Nygaard, no. 422 (C-F-108001!, as ‘*U. vinosa*’); **ditto**, the Liverpool coast side of Hurry Inlet, 70°50'N, 1900, leg. C. Krusse, Expeditio Danica in Groenlandiam orientalem 1900, s.n. (C-F-102447!, as ‘*U. vinosa*’; Rostrup 1904, as ‘*U. vinosa*’).

**EG**, Nerlerit Inaat/Constable Pynt, 70°45'36"N, 22°39'W, 15 Aug 2017, leg. H. Knudsen, s.n. (C-F-111320!, as ‘*U. vinosa*’); **ditto**, 70°44'24"N, 22°40'12"W, 1 Aug 2017, leg. H. Knudsen, no. HK 17.012 (C-F-104902!, as ‘*U. vinosa*’).

**EG**, Danmark Island, 70°30'N, 26°15'W, August 1891, leg. N. Hartz, Expeditio Danica in Groenlandiam orientalem 1891–1892, s.n. (C-F-102462!, as ‘*U. vinosa*’; Rostrup 1894, as ‘*U. vinosa*’).

**EG**, Kangerdlugssuaq Fjord, ca 68°N, 12 Aug 1932, leg. T. Bøcher, The Scoresby Sound Committee’s 2^nd^ East Greenland Expedition in 1932 to King Christian IX’s Land, no. 679 (C-F-102448!, 102456!); **ditto**, 16 Aug 1932, leg. T. Bøcher, no. 681 (C-F-102457!); **ditto**, 19 Aug 1932, leg. T. Bøcher, no. 682 (C-F-102455!) – all specimens initially as ‘*U. vinosa*’.

**EG**, Eskimo Island, 66°15'N, 35°15'W, 3 Aug 1899, leg. C. Krusse, Expeditio Danica in Groenlandiam orientalem 1898–1899, s.n. (C-F-102449!, as ‘*U. vinosa*’; Rostrup 1904, as ‘*U. vinosa*’).

**EG**, Tasiilaq, Kingak Angmagsivik, 65°57'N, 37°10'W, 21 Aug 1902, leg. C. Krusse, Expeditio Danica in Groenlandiam orientalem 1901–1902, s.n. (C-F-102458!, as ‘*U. vinosa*’; Rostrup 1904, as ‘*U. vinosa*’).

**EG**, Oksefjord, 64°37'N, 18 Sep 1933, leg. R. Bøgvad, no. 382 (C-F-107994!, as ‘*U. vinosa*’).

**EG**, Siorartussoq Island, 63°35'N, 40°42'W, 22 Aug 1970, leg. M. Astrup & L. Kliim-Nielsen, no. G.B.U. 1233 (C-Greenland herb.!, s.n.).

**EG**, Graahs Fjord, Imaersivik Island (as ‘Nukarfik, Graahs overvintringshavn’), 63°22'N, ca 41°06'W, 11 Aug 1932, leg. R. Bøgvad, no. 512 (C-F-107991!, as ‘*U. vinosa*’).

**Known hosts** — On Polygonaceae: Oxyria digyna.

**General distribution. Europe**: Arctic and Subarctic Europe and in mountains southwards to Spain, Italy, and Bulgaria. **Asia. North America**: Alaska, Canada, Greenland, mountains in the western U.S.A.

**Earlier reports from Greenland**: Rostrup (1888, 1891, 1894, 1904), Clinton (1902, 1904, 1906), Lind (1927, 1933, 1934), Hagen (1947), Vánky and Oberwinkler (1994) — all records as ‘*U. vinosa*’.

**Comments** — Both the host plant and the smut fungus are circumpolar–alpine species (Hultén and Fries 1986: 657).

Microbotryum vinosum is one of the most widespread smut fungi in Greenland and other Arctic and boreal parts of North America, Europe, and Russian Far East (e.g. in Alaska, Canadian Arctic Archipelago, northern Labrador, Svalbard, Jan Mayen, Iceland, Faeroes, Fennoscandia, Murmansk, Novaya Zemlya — Rostrup 1891; Blytt 1896; Lind 1927, 1934; Hagen 1941, 1947, 1950a; Linder 1947; Lindeberg 1959; Savile 1959, 1961; Parmelee 1969; Karatygin et al. 1999). In North America, Microbotryum vinosum reaches southwards to the mountains of western U.S.A. (in Washington, Wyoming, Colorado, and California — Clinton 1904; Linder 1947; Fischer 1953; Farr and Rossman 2019). In the higher mountains of the temperate zone in Europe and Asia, Oxyria digyna is so regularly infected that M. vinosum was found by us on a considerable part of the phanerogamic specimens inspected for infection in the herbaria.

### Orphanomyces Savile, Canad. J. Bot. 52: 342, 1974. — Type: O. arcticus (Rostr.) Savile.

**Sori** external on the leaf surface of sedges (Carex), as black, often confluent crusts. Mycelium **systemic, perennial. Infected plants** do not flower. **Spores** single or in loose balls, moderately large, with brown, coarsely sculptured walls. **Host-parasite interaction** by intracellular hyphae, coated by an electron-opaque matrix. Mature **septa** poreless (Vánky 2013).

#### 1(30) Orphanomyces arcticus (Rostr.) Savile, Canad. J. Bot. 52: 342, 1974. ≡ Tilletia arctica Rostr., Bot. Tidsskr. 15: 230, 1886. ≡ Cintractia arctica (Rostr.) Lagerh., in Blytt, Forh. Vidensk.-Selsk. Christiania 1896(6): 30, 1896. ≡ Ustilago arctica (Rostr.) B. Lindeb., Symb. Bot. Upsal. 16(2): 110, 1959. — Holotype on Carex macloviana (as ‘*C. festiva*’), Norway, Troms, Tromsø, 1885, leg. E. Warming, s.n. (C).

Fig. 29A–G

**Infection** systemic. **Sori** forming irregular crusts on the leaf epidermis of an infected plant; spore mass semi-agglutinated, blackish brown. **Infected plants** do not flower. **Spores** single, subglobose, irregular, broadly ellipsoidal or ovoid, sometimes irregularly elongated, (12–)13–18(–19.5) × (10.5–)11.5–14(–15) (14.9 ± 1.4 × 12.7 ± 0.7) µm (n/_2_ = 200), medium reddish brown; wall reticulate or foveolate-reticulate, 0.9–1.4 µm thick, meshes polyhedral or irregular, 0.4–2.0(–2.5) µm wide, muri up to 0.4(–0.5) µm high. In SEM meshes smooth or rugulose on the bottom.

**Figure 29.**
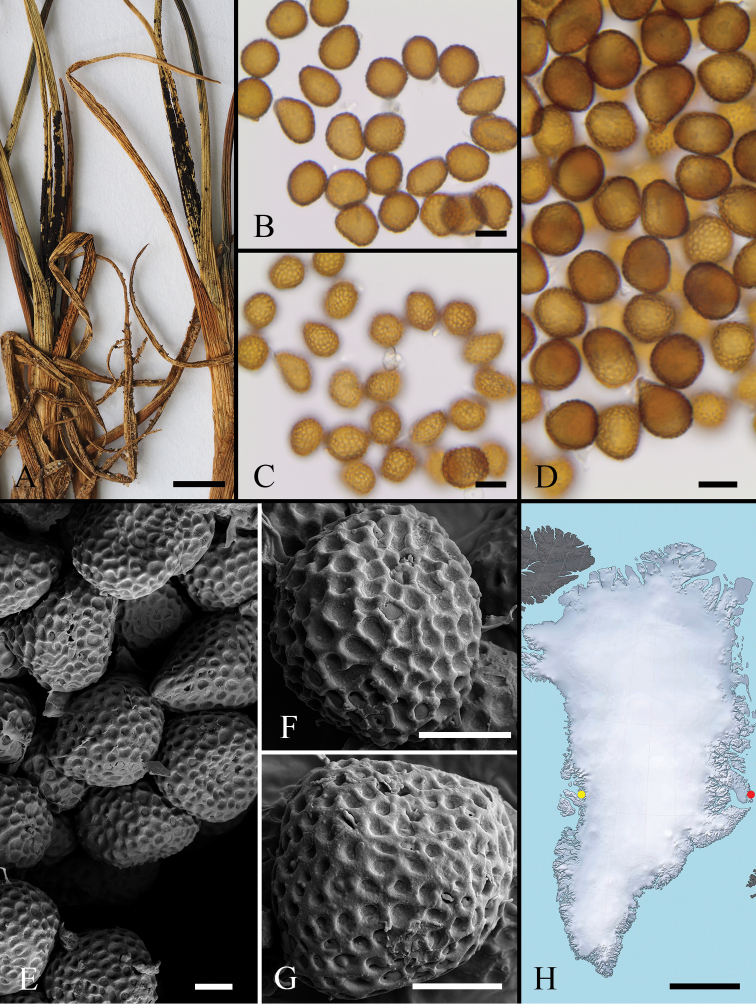
Orphanomyces arcticus on Carex (?) lachenalii (**A–C**) and C. maritima (**D–G**). **A** Habit (C-F-102485) **B–D** spores in LM (**B, C** C-F-102485, in median and surface view, respectively; **D** C-F-107976) **E–G** spores in SEM (C-F-107976) **H** distribution map (red circle – on Carex (?) lachenalii; yellow circle – on C. maritima). Scale bars: 0.5 cm (**A**); 10 µm (**B–D**); 5 µm (**E–G**); 500 km (**H**).

**Hosts and distribution within the studied area** — On Cyperaceae: Carex (?) lachenalii – East Greenland; C. maritima – West Greenland (Fig. 29H).

**Specimens examined or recorded.**

On **Carex** (?) **lachenalii** Schkuhr:

**EG**, Liverpool Land, the Liverpool coast side of Hurry Inlet, 70°50'N, 5 Aug 1900, leg. C. Krusse, Expeditio Danica in Groenlandiam orientalem 1900, s.n. (C-F-102485!, as ‘T. arctica’ on Carex sp.; Rostrup 1904, as ‘Tilletia arctica’).

On **Carex maritima** Gunnerus:

**WG**, Nuussuaq Peninsula (Nûgssuaq Pen.), Patorfik, 70°41'N, 17 Jul 1921, leg. A.E. Porsild, s.n. (C-F-107976!, the host as ‘C. incurva Lightf.’). On the label, the latitude is incorrect as ‘70°21'N’ instead of 70°41' or 70°42'N. In Porsild, A. (1926: 168), there is a note that the latitude of another locality is given incorrectly on the printed labels of some specimens, distributed by A.E. Porsild.

**Known hosts** — On Cyperaceae: Carex brunnescens (Pers.) Poir. (C. vitilis Fr.), C. canescens L. (C. cinerea Pollich), C. canescens × C. lachenalii, C.davalliana Sm., C. ebenea Rydb., C. eburnea Boott, C. glareosa Schkuhr ex Wahlenb., C. haydeniana Olney (C. nubicola Mack.), C. lachenalii (C. lagopina Wahlenb., C. tripartita All.), C. macloviana d’Urv. (C. festiva Dewey), C. maritima, C.scabrifolia Steud., C. stenophylla Wahlenb.

**General distribution. Europe**: Iceland, Norway, Sweden, Finland, France, Austria. **Asia**: Russian Far East, Mongolia, China. **North America**: Canada, Greenland, western U.S.A.

**Earlier reports from Greenland**: Rostrup (1904, as ‘Tilletia arctica’).

**Comments** — The spore germination of this smut fungus is insufficiently studied. Durán and Safeeulla (1968: 241) succeeded to germinate spores of ‘Cintractia arctica’ but only in water (not in nutrient media). The germination reported by them resulted in formation of long, sinuous, septate ‘promycelia with lateral outgrowths’ (op. cit., Figs 27, 28).

Both Carex lachenalii and C. maritima (regarding its distribution in the Northern Hemisphere; see the comments to Anthracoidea pseudofoetidae) are circumpolar–alpine species. The smut fungus, Orphanomyces arcticus, is also an Arctic–alpine species. It is a rarely collected species, distributed as follows: in Europe in Iceland and Fennoscandia, and in the Alps and the Pyrenees; in Asia in the Kamchatka Peninsula and mountains in Mongolia and China; and in North America in the eastern Canadian Arctic Archipelago and Greenland, and in mountains in western U.S.A. (Wyoming, Utah, Colorado) (Rostrup 1886, 1904; Blytt 1896; Lind 1934; Liro 1938; Fischer 1953; Lindeberg 1959; Jørstad 1963; Savile and Parmelee 1964; Jørstad and Gjærum 1966; Schmiedeknecht and Puncag 1966; Durán and Safeeulla 1968; Azbukina et al. 1995; Braun 1999; Guo 2000; Kruse et al. 2013; Farr and Rossman 2019).

Although Orphanomyces arcticus had been reported from Greenland by Rostrup (1904), this record was not included in the monographic treatment of the North American smut fungi of Fischer (1953). The only known record of Orphanomyces arcticus from Greenland is based on an infected plant, identified as ‘Carex sp.’, collected by C. Krusse during the Danish expedition to East Greenland in 1900. The plant collections from this expedition are listed in Kruuse (1905), and Carex lachenalii (as ‘*C. lagopina*’) is the only sedge, collected along Hurry Inlet (or with a locality labeled more generally as ‘Scoresby Sund’), that is currently known as a host of Orphanomyces arcticus. That is why, in this treatment the host plant of the smut fungus recorded by Rostrup (1904) is referred to Carex lachenalii.

Carex maritima is reported here for the first time as a host of Orphanomyces arcticus in Greenland.

### Planetella Savile, Canad. J. Bot. 29: 326, 1951. — Type: P. lironis Savile.

A monotypic genus. **Sori** in female flowers of sedges (Carex), around aborted nuts, forming black, hard bodies. For diagnostic characters of the **sori** and **spores**, see the description of P. lironis given below. **Host-parasite interaction** (after Vánky 2013) by intracellular hyphae, coated by an electron-opaque matrix. Mature **septa** (after Vánky 2013) poreless.

#### 1(31) Planetella lironis Savile, Canad. J. Bot. 29: 327, 1951. ≡ Sphacelotheca lironis (Savile) Thirum. & M.D. Whitehead, Sydowia 27: 85, 1975. ≡ Anthracoidea lironis (Savile) M. Piepenbr., in Agerer et al. (eds.), Frontiers in Basidiomycote Mycology: 159, 2004. — Holotype on Carex maritima, Canada, Nunavut, Chesterfield Inlet, 63°21'N, 90°42'W, 23 Aug 1950, leg. D.B.O. Savile, no. 1575 & C.T. Watts (DAOM 25883). Paratypes: on Carex maritima, Canada, Nunavut, Chesterfield Inlet, 9 Jul 1950, leg. D.B.O. Savile, no 921 & C.T. Watts (DAOM 25885); ditto, 20 Aug 1950, leg. D.B.O. Savile, no 1559 & C.T. Watts (DAOM 25886); on C. sabulosa (as ‘*C. leiophylla*’), Canada, Yukon, Carcross, 13 Jul 1949, leg. J.M. Gillett, no. 3772 (DAOM 25884).

Fig. 30A–E

**Sori** in some female flowers, around aborted nuts, as subglobose, hard bodies, ca 2 mm long, covered by a thick, yellow-brown peridium that later flakes away exposing a black, agglutinated (semi-agglutinated on the surface) spore mass. **Spores** slightly flattened, with a thick-walled, medium reddish brown equatorial band and two, thin-walled, light yellow-brown polar areas; in plane view suborbicular, orbicular, broadly elliptical or slightly irregularly rounded, in plane view (10.5–)11–13.5(–14.5) × (9.5–) 10–12.5(–13) (12.1 ± 0.6 × 11.2 ± 0.6) µm (n = 100); equatorial band 6.0–8.2 µm wide; in plane view polar areas suborbicular, orbicular, elliptical, broadly elliptical or slightly irregularly rounded, 5.5–7.5(–8.5) µm long; wall unevenly thickened, (1.5–)1.7–2.5(–2.7) µm thick at the equatorial band, 0.5–1.0(–1.2) µm thick at the polar areas, minutely verruculose, spore profile not affected. In SEM spore wall minutely verruculose; warts densely spaced, less than 0.2 µm in height, usually isolated. **Spore germination** unknown.

**Figure 30.**
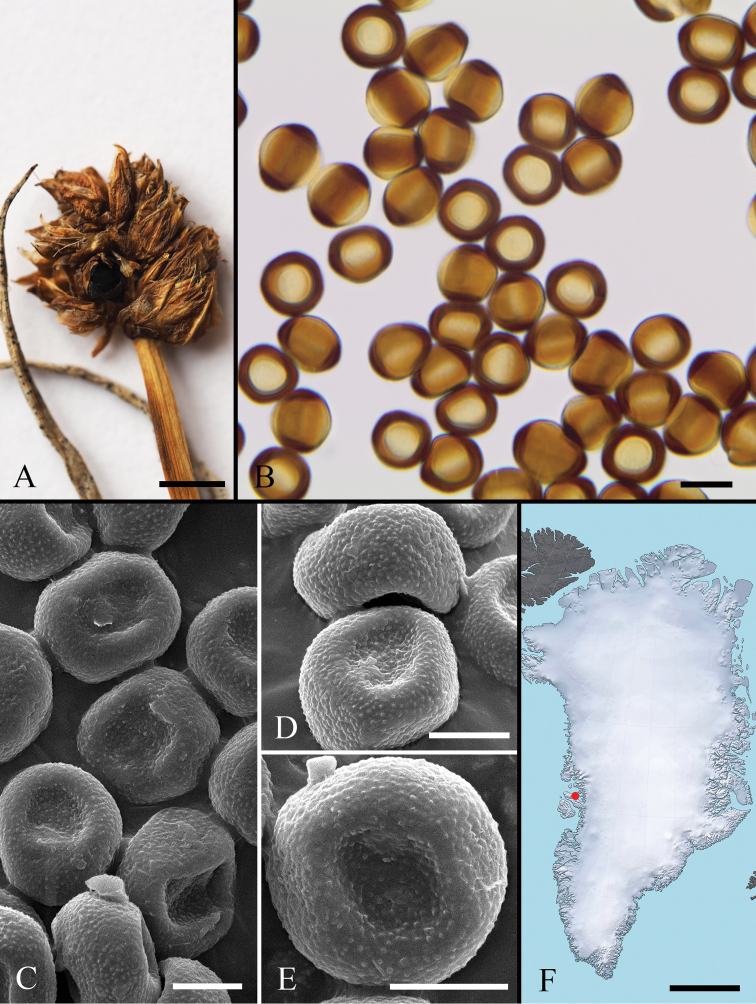
Planetella lironis on Carex maritima (T. Sørensen, no. 9196, C-Greenland herb., s.n.). **A** Habit **B** spores in LM**C–E** spores in SEM**F** distribution map. **D, E** reproduced, with permission, from Denchev & Denchev 2018. Scale bars: 0.5 cm (**A**); 10 µm (**B**); 5 µm (**C–E**); 500 km (**F**).

**Hosts and distribution within the studied area** — On Cyperaceae: Carex maritima – West Greenland (Fig. 30F).

**Specimens examined or recorded.**

On **Carex maritima** Gunnerus:

**WG**, Avannaata, Nuussuaq Peninsula (as ‘Nûgssuaq Pen.’), Kûtsiaq, 70°40'N, 52°27'W, 19 Aug 1947, leg. T. Sørensen, The Danish Botanical Expedition to West Greenland 1947, no. 9196 (C-Greenland herb.!, s.n.; Denchev and Denchev 2018).

**Known hosts** — On Cyperaceae: Carex maritima (C. incurva Lightf.), C. sabulosa Turcz. ex Kunth (C. leiophylla Mack.).

**General distribution. North America**: Canada (Yukon, Nunavut), Greenland.

**Earlier reports from Greenland**: Denchev and Denchev (2018).

**Comments** — As noted in the comments to Anthracoidea pseudofoetidae, Carex maritima is a widespread species, with bipolar distribution (in South America from Ecuador to Argentina), being a circumpolar–alpine species in the Northern Hemisphere — distributed there in Alaska, Canada, Greenland, and northern Eurasia, as well as in alpine regions of Europe and Central Asia. On this sedge, Planetella lironis is known only from the type locality in eastern Canada and a locality in West Greenland (Savile 1951; Denchev and Denchev 2018). It is noteworthy that P. lironis is not reported from North Europe and the alpine regions of Central Europe, which are among the best studied regions in the world for smut fungi, i.e. its absence there is not due to inadequate studies.

The second host, Carex sabulosa, has a very restricted distribution in North America. It is known from only 14 localities in Yukon and one in Alaska (Murray 2002; Baikal Sedge Recovery Team 2012). Carex sabulosa is also known from East Siberia, Kazakhstan, and North Mongolia (Egorova 1999). Planetella lironis is found at most localities of C. sabulosa in Yukon (Baikal Sedge Recovery Team 2012), but has never been found in Asia. Elven et al. (2018) recognize two subspecies within C. sabulosa: subsp. sabulosa (widespread in Siberia) and subsp. leiophylla (Mack.) A.E. Porsild (in Yukon and one in Alaska).

Planetella lironis is a remarkable example of a smut fungus with restricted distribution although its principal host is a widespread plant species (Denchev and Denchev 2018). Because the locality in the Yukon Territory is non-Arctic, the distribution of this smut fungus may be defined as northern North American.

### Schizonella J. Schröt., Beitr. Biol. Pflanzen 2: 362, 1877. — Type: S. melanogramma (DC.) J. Schröt.

**Sori** in leaves of Cyperaceae as black, short or long, pustulate streaks with agglutinated to powdery spore mass. **Spores** originally in pairs, arising by internal division of a mother cell, later may be separated into single spores. In S. cocconii spores born in pairs are agglutinated into balls. **Spore germination** of Ustilago-type. **Host-parasite interaction** by intracellular hyphae, coated by an electron-opaque matrix. Mature **septa** poreless (Vánky 2013).

#### Key to the relevant Schizonella species

**Table:** 

1	Spores light to medium yellow-brown. [On Carex myosuroides]	**S. elynae**
1*	Spores dark reddish brown. [On other species of Carex]	**S. melanogramma**

#### 1(32) Schizonella elynae (A. Blytt) Liro, Ann. Acad. Sci. Fenn., Ser. A 42(1): 308, 1936. ≡ Schizonella melanogramma var. elynae A. Blytt, Forh. Vidensk.-Selsk. Christiania 1896(6): 33, 1896 (as ‘β *elynae*’). ≡ Schizonella ‘scirpina’ [sic] (A. Blytt) Cif., Fl. Ital. Crypt., Pars 1, Fungi, Fasc. 17: 246, 1938. — Lectotype on Carex myosuroides (as ‘Elyna spicata’), Norway, Oppland, Dovre, Hjerkinn, 8 Aug 1889, leg. A. Blytt, s.n. (O) (design. by Lindeberg 1959: 57).

Fig. 31A–E

**Infection** systemic. **Sori** in leaves as striae or irregular spots, initially covered by the silvery epidermis which later ruptures disclosing a semi-agglutinated, blackish brown mass of spores. **Spores** joined in pairs, sometimes in threes, often separating into single spores, depressed on the contact side, in plane view suborbicular, irregular, broadly elliptical or ovate in outline, in plane view 6–9(–10.5) × (5.5–)6–8.5(–9.5) (7.6 ± 0.8 × 7.0 ± 0.7 µm (n/_1_ = 100), in side view usually irregularly hemispherical, light to medium yellow-brown; wall unevenly thickened, (0.8–)1.0–1.6(–1.9) µm thick, thinner and lighter on the contact side, smooth. In SEM spore wall rugulose or densely punctate-minutely verruculose; ornaments up to 0.15 µm in height; contact side with a rounded, concave area.

**Figure 31.**
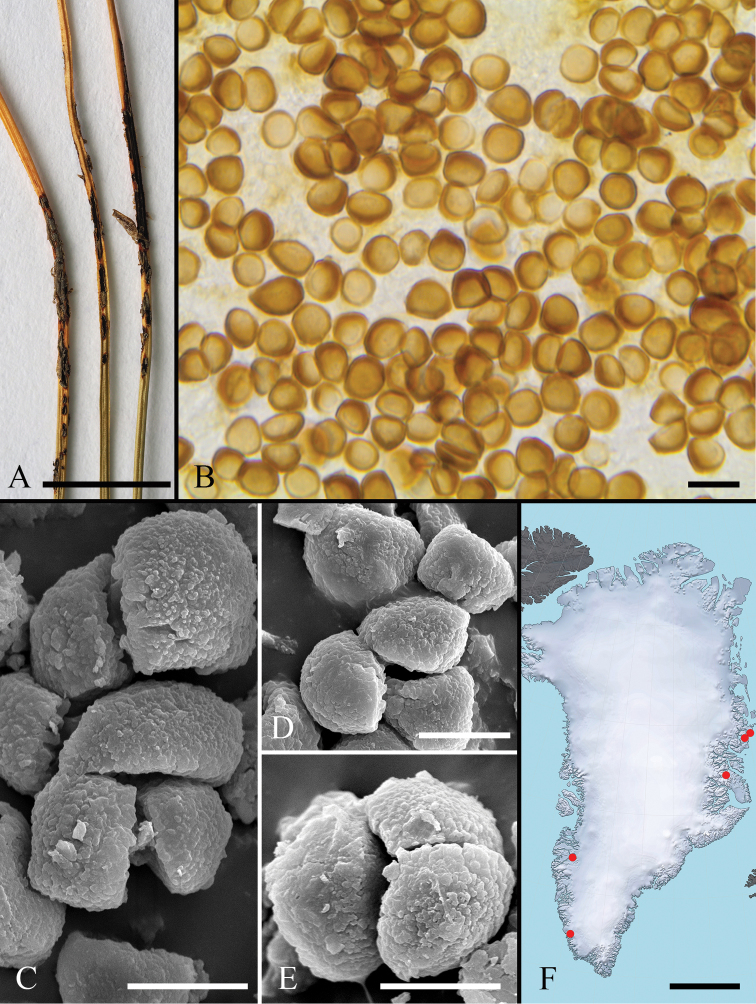
Schizonella elynae on Carex myosuroides (S. Frederiksen & L.B. Jørgensen, no. 68-1550, C-Greenland herb., s.n.). **A** Habit **B** spores in LM**C–E** spores in SEM**F** distribution map. Scale bars: 0.5 cm (**A**); 10 µm (**B**); 5 µm (**C–E**); 500 km (**F**).

**Hosts and distribution within the studied area** — On Cyperaceae: Carex (the Myosuroides clade): Carex myosuroides – West and East Greenland (Fig. 31F).

**Specimens examined or recorded.**

On **Carex myosuroides** Vill. (Elyna myosuroides (Vill.) Fritsch; E. spicata Schrad., E. bellardii (All.) K. Koch, Kobresia bellardii (All.) Degl., K. myosuroides (Vill.) Fiori & Paol., K. scirpina Willd.):

**WG**, Søndre Strømfjord, near the airport, near a large lake, 66°59'N, alt. 100 m, 11 Aug 1983, leg. J. Poelt & H. Ullrich, s.n. (GZU Acc. no. 98-83, n.v., the host as ‘K. myosuroides’; det. K. Vánky).

**WG**, NE of Qingua, 62°18'N, 49°10'W, alt. 730 m, 21 Jul 1968, leg. S. Frederiksen & L.B. Jørgensen, no. 68-1550 (C-Greenland herb.!, s.n.).

**EG**, Wollaston Forland, Herschell Bjerg (as ‘Kapp Herschel’), ca 74°16'N, 29 Jul 1929, leg. J. Vaage, s.n. (n.v.; not found in O; Hagen 1947, as ‘Schi. melanogramma’); **ditto**, Herschellhus, ca 74°14.6'N, 19°41.1'W, 1 Aug 1933, leg. A. Hagen, the Norwegian Expedition to NE Greenland 1933, s.n. (n.v.; not found in O; Hagen 1947, as ‘Schi. melanogramma’).

**EG**, Vesle Finsch Island, ca 74°00'N, 18 Jul 1933, leg. A. Hagen, the Norwegian Expedition to NE Greenland 1933, s.n. (n.v.; not found in O; Hagen 1947, as ‘Schi. melanogramma’).

**EG**, Alpefjord, Stauning Alper, 28 Jul 1933, leg. A. Hagen, the Norwegian Expedition to NE Greenland 1933, s.n. (n.v.; not found in O; Hagen 1947, as ‘Schi. melanogramma’).

**Known hosts** — On Cyperaceae: Carex myosuroides.

**General distribution. Europe**: Iceland, Norway, Sweden, Germany, Austria, Italy. **Asia**: Russia (East Siberia). **North America**: Canada, Greenland.

**Earlier reports from Greenland**: Hagen (1947, as ‘Schi. melanogramma’).

**Comments** — Carex myosuroides is a circumpolar–alpine species (Hultén and Fries 1986: 423; Elven et al. 2018), distributed in Eurasia and North America. There are two smut fungi on this host plant: Anthracoidea elynae and Schizonella elynae. Whereas A. elynae is a widespread smut fungus, S. elynae seems to be uncommon all over the area of its host.

Schizonella elynae is known from North Europe (Iceland, Norway, and Sweden), the Alps (Germany, Austria, and Italy), East Siberia (Lena-Kolyma region — Bolshoy Anyuy River), and Canada (Blytt 1896; Lindeberg 1959; Jørstad 1963; Parmelee 1969; Govorova 1990; Karatygin et al. 1999; Helgi Hallgrímsson and Guðríður Gyða Eyjólfsdóttir 2004; Kruse et al. 2019). It has been previously reported also from East Greenland (Hagen 1947) but under the name S. melanogramma. In Canada, S. elynae is known from the Canadian Arctic Archipelago (Victoria Island and Baffin Island; Parmelee 1969; specimens in DAOM) and ‘an alpine region’ in British Columbia (Parmelee 1969). Based on the scarce information about the distribution of this smut fungus, we consider it as a circumpolar–alpine species.

#### 2(33) Schizonella melanogramma (DC.) J. Schröt., s. lat., Beitr. Biol. Pflanzen 2: 362, 1877. ≡ Uredo melanogramma DC., Fl. Franç., ed. 3 (Paris) 6: 75, 1815. ≡ Caeoma melanogramma (DC.) Schltdl., Linnaea 1: 238, 1826. ≡ Puccinia melanogramma (DC.) Unger, Ueber den Einfluß des Bodens, etc.: 217, 1836. ≡ Thecaphora melanogramma (DC.) Lév., Ann. Sci. Nat., Bot., Sér. 3, 8: 373, 1847. ≡ Geminella melanogramma (DC.) Magnus, Hedwigia 14: 19, 1875. — Lectotype on Carex digitata, France, Jura, leg. J.F. de Chaillet (design. by Liro 1938: 305).

Fig. 32A–G

[For the nomenclature of this fungus in its broad circumscription, see Vánky 2011: 498].

**Infection** systemic. **Sori** in leaves as striae or irregular spots, initially covered by the epidermis which later ruptures, disclosing a semi-agglutinated, blackish brown mass of spores. **Spores** joined in pairs, often separating into single spores, depressed on the contact side, in plane view suborbicular, broadly elliptical, irregular or ovate in outline, in plane view (6–)7–10(–11) × (5.5–)6.5–10(–11) (8.8 ± 1.0 × 7.9 ± 0.8) µm (n/_1_ = 100), in side view usually irregularly hemispherical, dark reddish brown; wall unevenly thickened, (0.8–)1.0–1.7(–2.0) μm thick, thinner and lighter on the contact side, smooth. In SEM spores almost smooth to rugulose-punctate; ornaments up to 0.10 μm in height; with a rounded, concave area on the contact side. **Spore germination** (after Brefeld 1895: 148–150, PI. 9, Figs 6–12; Ingold 1992: 166, Figs 28–29; Vánky 2011: 498) results in a 4-celled basidium of 3 + 1 arrangement, where the fourth cell remains included in the spore, the distal, three-celled part normally separates from the rest. Laterally and terminally the basidium develops ovoid to elongate basidiospores.

**Figure 32.**
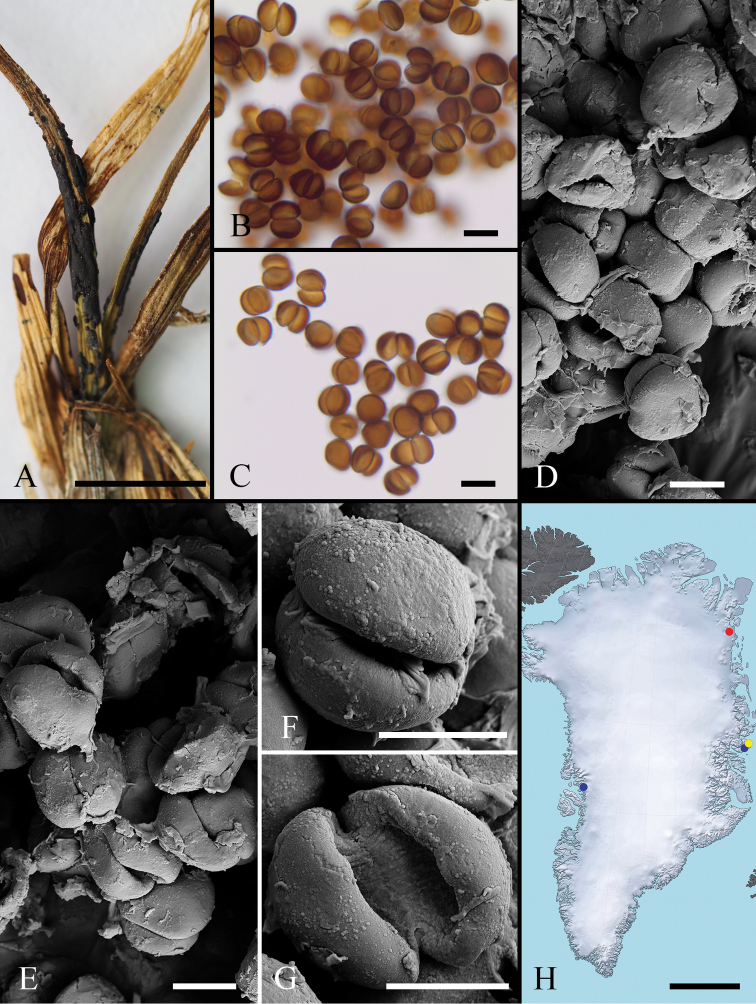
Schizonella melanogramma on Carex fuliginosa subsp. misandra (C-F-107987). **A** Habit **B, C** spores in LM**D–G** spores in SEM**H** distribution map (red circle – on Carex fuliginosa subsp. misandra; yellow circle – on C. nardina; blue circles – on C. rupestris). Scale bars: 0.5 cm (**A**); 10 µm (**B, C**); 5 µm (**D–G**); 500 km (**H**).

**Hosts and distribution within the studied area** — On Cyperaceae: Carex fuliginosa subsp. misandra, C. nardina s. lat., C. rupestris – West and East Greenland (Fig. 32H).

**Specimens examined or recorded.**

On **Carex fuliginosa** subsp. **misandra** (R. Br.) Nyman (C. misandra R. Br.):

**EG**, in southernmost Kronprins Christian Land, Blåsø, 29 Jul 1987, leg. C. Bay, s.n. (C-F-107987!, the host as ‘C. misandra’).

On **Carex nardina** (Hornem.) Fr., **s. lat.**:

**EG**, Strindberg Land (as ‘Strindberghalvøya’), near the Danish Hut, 30 Jul 1933, leg. A. Hagen, the Norwegian Expedition to NE Greenland 1933, s.n. (n.v.; not found in O; Hagen 1947).

On **Carex rupestris** All.:

**WG**, Maamorilik, NE end of Qaumarujuk Fjord, 71°09'N, 51°15'W, 9 Aug 1983, leg. J. Poelt & H. Ullrich, s.n. (GZU Acc. no. 98-83, n.v.; det. K. Vánky).

**EG**, Hold with Hope, Myggbukta (on the north side of Mackenzie Bugt), NW of the Norwegian Station (at 73°29.4'N), 31 Jul 1933, leg. A. Hagen, the Norwegian Expedition to NE Greenland 1933, s.n. (n.v.; not found in O; Hagen 1947).

**EG**, Strindberg Land (as ‘Strindberghalvøya’), ca 1 km E of the Danish Hut, 30 Jul 1933, leg. A. Hagen, the Norwegian Expedition to NE Greenland 1933, s.n. (n.v.; not found in O; Hagen 1947).

**Known hosts** — On Cyperaceae: on 76 species of Carex (Vánky 2013).

**General distribution. Europe. Asia. North America**: Canada, Greenland, U.S.A.

**Earlier reports from Greenland**: Hagen (1947).

**Comments** — In the present treatment, S. melanogramma is considered in its broad sense.

In the keys to the relevant Schizonella species, both spore color and length are usually used for distinguishing S. elynae from S. melanogramma. In the current case, however, only one specimen of S. melanogramma from Greenland (on C. fuliginosa subsp. misandra) was available to the authors and unfortunately, its sori were too young. Because of this reason, the spore sizes in the description are smaller than the typical ones for S. melanogramma, and spore length is not used in the key to this species.

Carex fuliginosa subsp. misandra is a new host for this smut fungus in Greenland.

### Stegocintractia M. Piepenbr., Begerow & Oberw., Mycologia 91: 497, 1999. — Type: S. luzulae (Sacc.) M. Piepenbr., Begerow & Oberw.

**Infection** systemic. **Sori** on plants in the Juncaceae, in all spikelets or around pedunculi of an infected inflorescence, forming a black, agglutinated spore mass with a powdery surface. Young sori covered by a fungal peridium, sterile stroma lacking. **Spores** single, pigmented (brown), ornamented, without appendages. **Host-parasite interaction** by intracellular hyphae, coated by an electron-opaque matrix. Mature **septa** poreless (Vánky 2013).

#### Key to the relevant Stegocintractia species

**Table:** 

1	Spores 16.5–23.5 µm long, verruculose-echinulate. [On Luzula confusa, L. nivalis]	**S. hyperborea**
1*	Spores 19.5–30 µm long, foveolate. [On Luzula multiflora]	**S. luzulae**

#### 1(34) Stegocintractia hyperborea (A. Blytt) M. Piepenbr., Nova Hedwigia 70(3–4): 321, 2000. ≡ Ustilago hyperborea A. Blytt, Forh. Vidensk.-Selsk. Christiania 1896(6): 28, 1896. ≡ Cintractia hyperborea (A. Blytt) Liro, Ann. Acad. Sci. Fenn., Ser. A 42(1): 42, 1938 (nom. illegit., ICN Art. 53.1); non Cintractia hyperborea Cif., Ann. Mycol. 29: 64, 1931 (q.e. Anthracoidea elynae (Syd.) Kukkonen). ≡ Cintractia dovrensis Jørst., in Zundel, Pennsylvania State Coll. School Agric. Dept. Bot. Contr. 176: 27, 1953. — Holotype on Luzula confusa, Norway, Oppland, Dovre, Fokstuhö, 14 Aug 1893, leg. A. Blytt (O-F72813).

Fig. 33A–F

**Infection** systemic. **Sori** in all spikelets of an infected plant, filling the basal part of the perianth and surrounding the spikelet axis, more or less enclosed by the perianth segments, initially covered by a thin peridium which soon flakes away exposing an initially agglutinated, later powdery spore mass. **Spores** slightly flattened, in plane view suborbicular, slightly irregular, broadly elliptical or orbicular, in plane view (16.5–)17.5–22(–23.5) × (15.5–)16.5–20(–21) (19.7 ± 1.1 × 18.0 ± 1.0) µm (n/_3_ = 300), medium to dark reddish brown; wall two-layered, unevenly thickened, (3.0–)3.2–4.3(–4.7) µm thick (including the 0.7–1.3 µm thick inner layer), usually with two thinner and lighter stripe-like areas on the opposite flattened sides of the spores, moderately verruculose-echinulate, ornaments up to 0.5(–0.6) µm in height, spore profile affected. In SEM ornaments densely spaced, isolated or confluent in small groups; with an elongated or sometimes rounded concave areas of the flattened sides.

**Figure 33.**
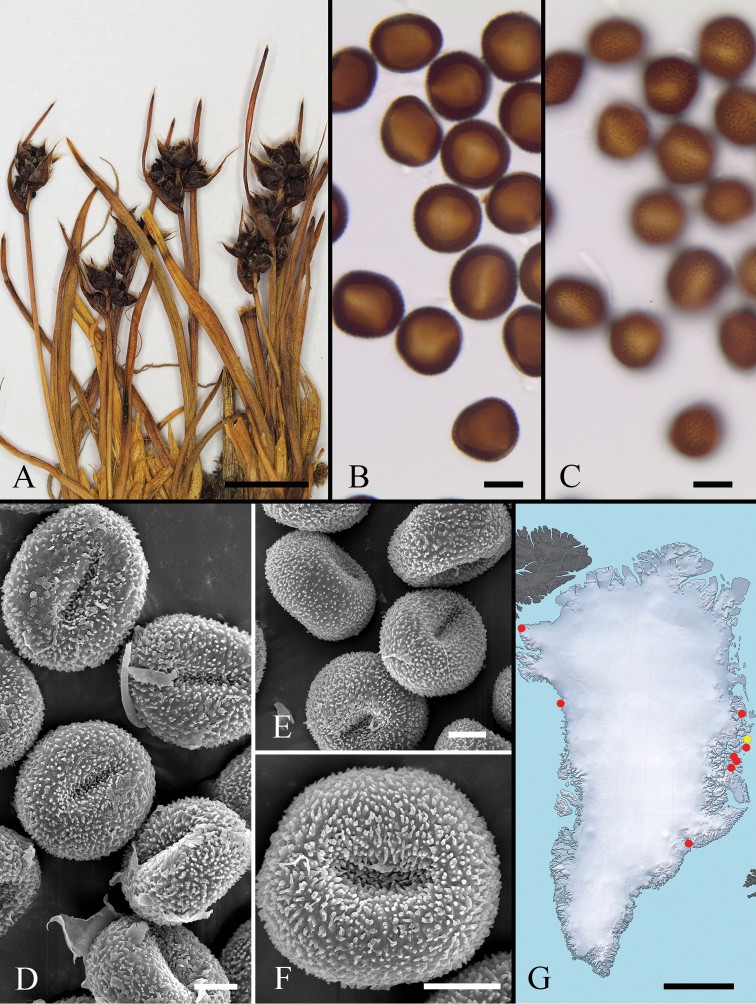
Stegocintractia hyperborea on Luzula confusa. **A** Habit (4 Aug 1929, leg. J. Vaage, s.n., O, s.n.) **B, C** spores in LM, in median and surface view, respectively (4 Aug 1929, leg. J. Vaage, s.n., O, s.n.) **D–F** spores in SEM (B. Fredskild & C. Bay, Pl. Vasc. Groenland. Exsicc., no. 672, C-Greenland herb., s.n.) **G** distribution map (red circles – on Luzula confusa; yellow circle – on L. nivalis). Scale bars: 0.5 cm (**A**); 10 µm (**B, C**); 5 µm (**D–F**); 500 km (**G**).

**Hosts and distribution within the studied area** — On Juncaceae: Luzula confusa, L. nivalis – North and East Greenland (Fig. 33G).

**Specimens examined or recorded.**

On **Luzula confusa** Lindeb.:

**NG**, Foulk Fjord, in clivo ad Etah, 78°18'N, 11–12 Aug 1899, leg. H.G. Simmons, s.n. (O!, s.n., as ‘Ci. hyperborea’; Hagen 1947, as ‘Ci. hyperborea’).

**NG**, Melville Bugt, Tugtuligssuaq, 75°23'N, 58°35'W, alt. 350 m, 16 Aug 1979, leg. B. Fredskild & C. Bay, Plantae Vasculares Groenlandicae Exsiccatae, no. 672 (C-Greenland herb.!, s.n.).

**EG**, Dronning Margrethe II Land, Hochstetter Forland, Jónsbú (NE of the mouth of Ardencaple Fjord), ca 75°19.2'N, 20°23.3'W, 3 Aug 1933, leg. A. Hagen, the Norwegian Expedition to NE Greenland 1933, s.n. (O!, s.n., as ‘Cintractia sp.’; Hagen 1947, as ‘Ci. hyperborea’).

**EG**, Gael Hamke Bugt, Jackson Island, ca 73°55'N, 12 Aug 1933, leg. A. Hagen, the Norwegian Expedition to NE Greenland 1933, s.n. (O!, s.n., as ‘Cintractia sp.’; Hagen 1947, as ‘Ci. hyperborea’).

**EG**, Hold with Hope, Troldsøen (as ‘Trollvatnet’), ca 73°29'N, 20°39'W, 9 Aug 1933, leg. A. Hagen, the Norwegian Expedition to NE Greenland 1933, s.n. (O!, s.n., as ‘Cintractia sp.’; Hagen 1947, as ‘Ci. hyperborea’).

**EG**, Ymer Island, Celsius Bjerg (as ‘Celsiusfjellet’), ca 73°08'N, 4 Aug 1929, leg. J. Vaage, s.n. (O!, s.n., as ‘Cintractia sp.’; Hagen 1947, as ‘Ci. hyperborea’).

**EG**, Geographical Society Island, 15 km W of Husbukta (ca 72°49.7'N, 22°52.5'W), 17 Aug 1930, leg. P.F. Scholander, s.n. (O!, s.n., as ‘Cintractia sp.’; Hagen 1947, as ‘Ci. hyperborea’).

**EG**, Traill Island, Holm-Vika (as ‘Holmvika’) near Kong Oscar Fjord, ca 72°30.1'N, 24°00.3'W), 11 Aug 1929, leg. J. Vaage, s.n. (O!, s.n., as ‘Cintractia sp.’; Hagen 1947, as ‘Ci. hyperborea’); **ditto**, Kapp Simpson, ca 72°08.1'N, 22°11.6'W, 12 Aug 1929, leg. J. Vaage, s.n. (O!, s.n., as ‘Cintractia sp.’; Hagen 1947, as ‘Ci. hyperborea’).

**EG**, ‘Kangudlugsuak’ (probably misspelled Kangerdlugsuak Fjord, currently, Kangerlussuaq Fjord, ca 68°20–25'N, where L. confusa is known to be distributed, see Devold and Scholander 1933: 111), 18 Jun 1889, leg. ?, Ryder’s Expedition (n.v.; Liro 1938: 43, as ‘Ci. hyperborea’).

On **Luzula nivalis** (Laest.) Spreng. (syn. L. arctica Blytt):

**EG**, Gael Hamke Bugt, Jackson Island, ca 73°55'N, 12 Aug 1933, leg. A. Hagen, the Norwegian Expedition to NE Greenland 1933, s.n. (O!, s.n., as ‘Cintractia sp.’ on ‘Luzula arctica’; Hagen 1947, as ‘Ci. hyperborea’).

**Known hosts** — On Juncaceae: Luzula confusa, L. nivalis.

**General distribution. Arctic and Subarctic Eurasia**: Spitsbergen, Norway, Sweden, Russia (Wrangel Island). **Arctic North America**: Canada (Baffin Island), Greenland.

**Earlier reports from Greenland**: Liro (1938, as ‘Ci. hyperborea’), Hagen (1947, as ‘Ci. hyperborea’).

**Comments** — The distribution of Stegocintractia hyperborea is restricted to the Arctic and Subarctic regions. This smut fungus infects only two wood rushes, L. confusa and L. nivalis, both belonging to Luzula sect. Thyrsanochlamydeae (sensu Kirschner 2002; in *Flora Europaea*, Chrtek and Křísa 1980, considered as ‘L. sect. Nivales’). The principal host of this smut fungus, Luzula confusa, is a circumpolar species, widely distributed in the North American Arctic (Hultén and Fries 1986: 169; Swab 2000). On this host, S. hyperborea is reported from the eastern Canadian Arctic Archipelago (only one collection from Baffin Island, published by Linder 1947, and Savile 1957, as ‘Ci. luzulae’), Greenland (Liro 1938; Hagen 1947; and additional data in this treatment), Spitsbergen (from a single locality, Adventfjorden, at ca 78°10'N, recorded by Lind 1928; see also Gjærum 1991; Elvebakk et al. 1996; Tojo et al. 2013), Norway (Blytt 1896; Jørstad 1963; Gjærum 1972), Swedish Lapland (Liro 1938; Selander 1950; Lindeberg 1959), and Wrangel Island (Govorova 1990; Karatygin et al. 1999; Piepenbring 2000; Karatygin 2012).

The only record of S. hyperborea on Luzula nivalis, also a circumpolar species (Hultén and Fries 1986: 170; Aiken et al. 2007), is from East Greenland (Hagen 1947).

A third host, Luzula arcuata, is listed in Vánky (2011), but it is erroneously added to the host plants on the base of Lindeberg’s (1959: 119) treatment of Stegocintractia hyperborea (as ‘Ustilago hyperborea’) in Sweden where L. confusa (that had been already recorded from Sweden by Liro 1938, and Selander 1950) is accepted as a synonym of L. arcuata.

Stegocintractia hyperborea is a rarely reported smut fungus. Surprisingly, the highest number of its localities are in Greenland. On the basis of available information, the distribution pattern of S. hyperborea is an amphi-Atlantic (western)–European (northern) & Asian (northeastern Arctic) species, with one Atlantic–northern European part area and another Far East Arctic part area. Since both host plants are circumpolar species, it is still unclear whether S. hyperborea is a fungus with remarkably large disjunctions in the distribution (in the Canadian Arctic and Russian Arctic) or these disjunctions reflect insufficient sampling. In both cases, S. hyperborea is a good example of a smut fungus that does not follow the distribution of its hosts.

#### 2(35) Stegocintractia luzulae (Sacc.) M. Piepenbr., Begerow & Oberw., Mycologia 91: 497, 1999. ≡ Ustilago luzulae Sacc., Mycotheca Veneta: no. 73, 1873. ≡ Cintractia luzulae (Sacc.) G.P. Clinton, J. Mycol. 8: 143, 1902. — Type on Luzula forsteri, Italy, ‘in sylva Cansiglio, agri Tarvisini’, leg. G.A. de Bérenger.

Fig. 34A–G

**Infection** systemic. **Sori** in all spikelets of an infected plant, filling the basal part of the perianth and surrounding the spikelet axis, more or less enclosed by the perianth segments, initially covered by a thin peridium which soon flakes away exposing an initially agglutinated, later powdery spore mass. **Spores** slightly flattened, in plane view suborbicular, orbicular or broadly elliptical, sometimes slightly irregular or ovate, in plane view (19.5–)20.5–28.5(–30) × (18.5–)19.5–25(–26) (25.0 ± 1.9 × 22.3 ± 1.4) µm (n/_2_ = 200), medium to dark reddish brown; wall unevenly thickened, (1.6–)1.8–3.2(–3.5) µm thick (a faint, 0.7–1.3 μm thick inner layer may be observed in some spores), often with a thinner, slightly paler rounded area of 8–13 µm diam, foveolate. In SEM spore wall shallow-foveolate, foveoles rugulose on the bottom, wall densely punctate to minutely verruculose between foveoles. **Spore germination** (after Piepenbring 2000: 324) results in ramified hyphae.

**Figure 34.**
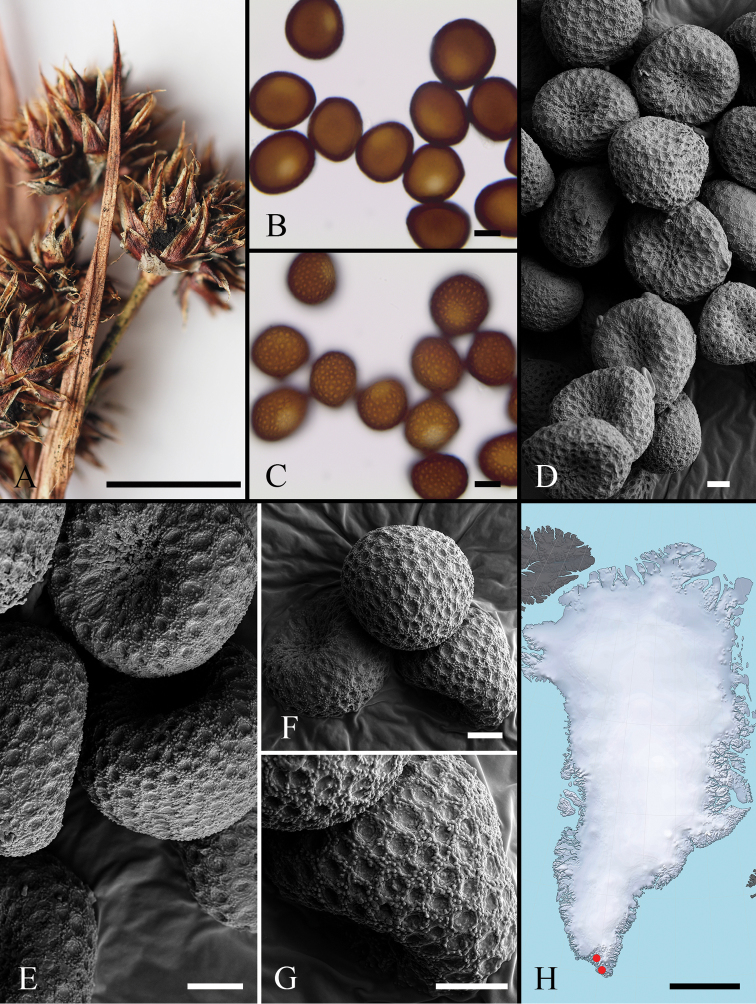
Stegocintractia luzulae on Luzula multiflora (C-F-111319). **A** Habit **B, C** spores in LM, in median and surface view, respectively **D–G** spores in SEM**H** distribution map. Scale bars: 0.5 cm (**A**); 10 µm (**B, C**); 5 µm (**D–G**); 500 km (**H**).

**Hosts and distribution within the studied area** — On Juncaceae: Luzula multiflora – West Greenland (Fig. 34H).

**Specimens examined or recorded.**

On **Luzula multiflora** (Ehrh.) Lej.:

**WG**, Igaliku, 60°59'N, 45°25'W, 15 Aug 2018, leg. H. Knudsen, no. HK 18.179 (C-F-111319!, as ‘U. luzulae’).

**WG**, Tasermiut Fjord, Qinqua-valley at Taserssuaq Lake, 60°16'N, 44°33'W, 28 Jul 1984, leg. T. Læssøe, no. TL 84.095 (C-F-107977!, as ‘U. luzulae’).

**Known hosts** — On Juncaceae: Luzula campestris (L.) DC., L. echinata (Small) F.J. Herm., L. forsteri (Sm.) DC., L. luzulina (Vill.) Racib. (L. flavescens (Host) Gaudin), L. luzuloides (Lam.) Dandy & Wilmott (L. albida (Hoffm.) DC., L. nemorosa (Pollich) E .Mey.), L. multiflora s. lat., L. multiflora subsp. frigida (Buchenau) V.I. Krecz. (L. frigida (Buchenau) Sam.), L. nivea (Nathh.) DC., L. pilosa (L.) Willd., L. spicata (L.) DC., L. sudetica (Willd.) Schult., L. sylvatica (Huds.) Gaudin subsp. sylvatica, L. sylvatica subsp. sieberi (Tausch) K. Richt. (L. sieberi Tausch), L. wahlenbergii Rupr.

**General distribution. Europe. Asia** (Russian Far East). **North America**: Alaska, Greenland, midwestern U.S.A.

**Comments** — Stegocintractia luzulae is reported here for the first time from Greenland. It was found on Luzula multiflora, a circumboreal-polar species (in its broad circumscription).

Stegocintractia luzulae is a circumboreal species, found on thirteen species of Luzula. It is an easily overlooked smut fungus, with records mainly from Fennoscandia and Central Europe (Liro 1938; Lindeberg 1959; Jørstad 1963; Vánky 1985a; Zogg 1986; Scholz and Scholz 1988; Zwetko and Blanz 2004; Riegler-Hager 2007; Klenke and Scholler 2015). From North America, S. luzulae has been previously recorded only from Alaska (on Luzula multiflora subsp. frigida, Savile 1957), and the midwestern U.S.A. (on L. campestris from Indiana, Fischer 1953, and on L. echinata from Illinois, Boewe 1964).

### Tilletia Tul. & C. Tul., Ann. Sci. Nat., Bot., Sér. 3, 7: 112, 1847. — Type: T. caries (DC.) Tul. & C. Tul.

**Infection** systemic or local. **Sori** on host plants in the Poaceae, most commonly in the ovaries, which fill with a semi-agglutinated or powdery spore mass intermixed with sterile cells. In some species, the sori are formed on leaves and culms, as streaks. Exceptionally, the sori appear as swellings on the culms or cover the surface of the leaves, or form witches’ brooms. Peridium and columella lacking. **Spores** single, medium to large sized, usually ornamented (reticulate, cerebriform, verrucose, tuberculate or with cylindrical projections), rarely smooth, often encased in a hyaline gelatinous sheath. **Sterile cells** usually present between the spores, solitary, variously shaped, smooth but also weakly or evidently ornamented, hyaline or slightly pigmented, naked or sheathed. **Spore germination** by means of an aseptate basidium (holobasidium), bearing terminal basidiospores which often conjugate in situ, giving rise to infection hyphae, blastospores and ballistospores (secondary sporidia), or basidiospores numerous, acicular, giving rise to infection hyphae without conjugation. **Host-parasite interaction** by intercellular hyphae; interaction apparatus is lacking. **Septal pore** is a dolipore traversed by two membranous plates, pore caps lacking (after Vánky 2013).

#### 1(36) Tilletia cerebrina Ellis & Everh. ex Sacc., Syll. Fung. 7: 483[bis], 1888. ≡ Tilletia cerebrina Ellis & Everh., J. Mycol., 3: 56, 1887 (nom. inval., a provisional name; ICN Art. 36.1). — Holotype on Deschampsia cespitosa, U.S.A., Rocky Mountains, leg. F.L. Scribner, no. 17-87 (NY 03021318).

Fig. 35A–F

= Tilletia airae A. Blytt, Forh. Vidensk.-Selsk. Christiania 1896(6): 31, 1896. — Holotype on Deschampsia cespitosa (as ‘Aira caespitosa’), Norway, Troms, Tromsøysund, Renøen, 3 Aug 1882, leg. B. Esmark, s.n. (O) (syn. by Lindeberg 1959: 70). 

= Tilletia airina Syd., Ann. Mycol. 35: 259, 1937. — Holotype on Aira caryophyllea, Portugal, Madeira Is., Plateau Paul da Serra, alt. 1400 m, August 1936, leg. G. Viennot-Bourgin, no. 45 (Herb. ?); isotypes BPI 172338, 195150, H.U.V. 14984 (syn. by Durán and Fischer 1961: 46).

**Infection** systemic, all spikelets of a panicle affected. **Sori** in all ovaries of an infected plant, broadly ellipsoid or ovoid, 1.0–1.5 × 0.7–1.0 mm, with a short, acute tip, bearing a rudimentary style and stigmas, partially visible between spreading floral bracts, covered by a thin, purplish brown or yellow-brown pericarp that later ruptures to expose a powdery, dark reddish brown mass of spores and sterile cells. **Sterile cells** irregular, sometimes subglobose, broadly ellipsoidal, ellipsoidal or reniform, (12–)13–19(–20.5) × (7.5–)8.5–14(–15.5) µm, hyaline; cell wall (1.0–)1.2–1.7(–2.0) µm thick. In SEM cell wall smooth. **Spores** globose, subglobose or broadly ellipsoidal, sometimes ovoid or slightly irregularly rounded, (22–)23–28(–29.5) × (20.5–)21.5–26(–27.5) (25.5 ± 1.4 × 23.9 ± 1.4) µm (n/_1_ = 100), medium reddish brown, cerebriform to incompletely reticulate; spore wall 2.5–3.2(–3.5) µm thick (including reticulum); muri (21–)23–27(–29) on equatorial circumference, in optical median view subacute, acute or blunt, (0.7–)0.9–1.7(–2.0) µm high. In SEM interspaces smooth. **Spore germination** (after Siang 1954; Vánky 2011) results in aseptate basidia developing (3–)4–6(–8) apical, multinucleate, long-cylindrical basidiospores that germinate without fusion, producing infection hyphae and multinucleate ballistospores. Repeated germination of multinucleate ballistospores gives rise to uninucleate ballistospores.

**Figure 35.**
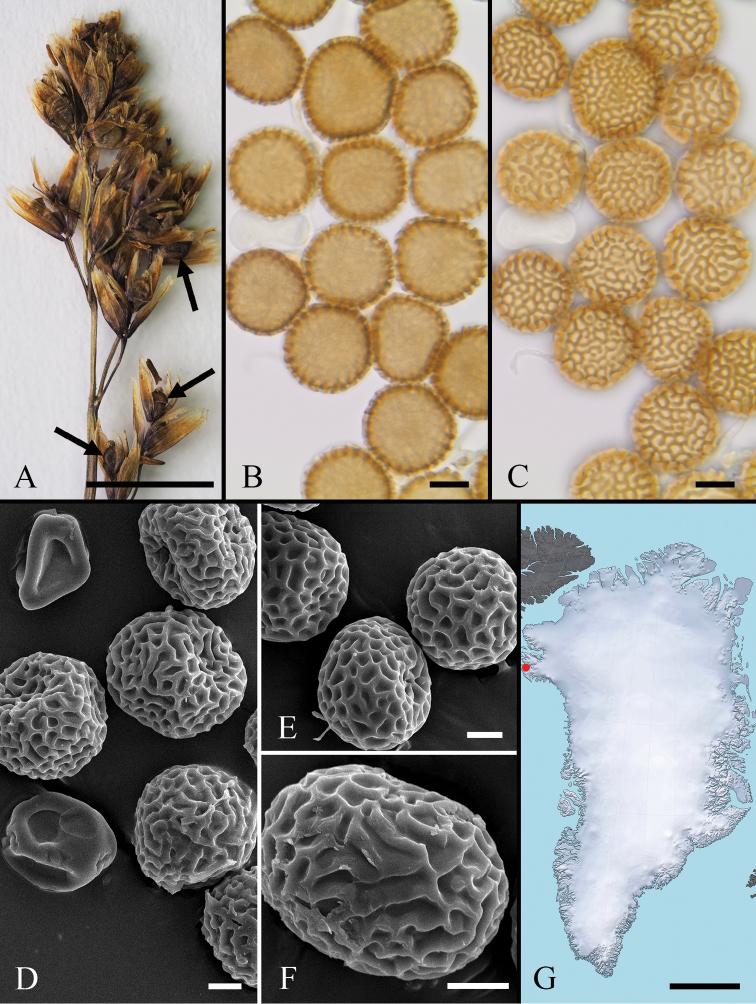
Tilletia cerebrina on Deschampsia cespitosa subsp. septentrionalis (C-F-102484). **A** Habit (black arrows show sori) **B, C** spores in LM, in median and surface view, respectively **D–F** spores and sterile cells in SEM**G** distribution map. Scale bars: 0.5 cm (**A**); 10 µm (**B, C**); 5 µm (**D–F**); 500 km (**G**).

**Hosts and distribution within the studied area** — On Poaceae: Deschampsia brevifolia – North Greenland (Fig. 35G).

**Specimens examined or recorded.**

On **Deschampsia brevifolia** R. Br. (D. cespitosa subsp. brevifolia (R. Br.) Tzvelev, D. cespitosa subsp. septentrionalis Chiapella, D. arctica (Spreng.) Merr.):

**NG**, Wolstenholme Fjord (as ‘Wolstenholm Sound’), Thule, ca 76°30–33'N, 1919, leg. J.N. Nygaard, s.n. (C-F-102483!, 102484!, the host as ‘D. arctica Fries’; WSP 63725!; Ostenfeld 1923; Liro 1938; Durán and Fischer 1961).

**Known hosts** — On Poaceae: Aira caryophyllea L., A. cupaniana Guss., A. praecox L., Deschampsia brevifolia, D. cespitosa (L.) P. Beauv. (Aira cespitosa L., Deschampsia glauca Hartm.), D. danthonioides (Trin.) Munro, D. elongata (Hook.) Munro, D. media (Gouan) Roem. & Schult. (D. juncea (Vill.) P. Beauv.), D. koelerioides Regel.

**General distribution. Europe**: UK (Scotland), Norway, Sweden, Finland, Germany, Austria, Czech Republic, Romania, Spain, Italy, Bulgaria. **Asia**: Russia (West Siberia, northeastern Arctic — Wrangel Island, Far East), Kazakhstan, Uzbekistan. **Africa**: Madeira. **North America**: Alaska, Canada, Greenland, western and north-central U.S.A. **South America**: Argentina. **Australia.**

**Earlier reports from Greenland**: Ostenfeld (1923), Liro (1938: 355), Durán and Fischer (1961).

**Comments** — In Greenland, Tilletia cerebrina is only known from Thule, with a single gathering from 1919 on Deschampsia brevifolia. Some authors (e.g. Kawano 1966; Tzvelev 1976; Chiapella and Probatova 2003; Chiapella 2016) considered Deschampsia brevifolia as part of D. cespitosa complex, with a rank of subspecies. In this case, the correct name is D. cespitosa subsp. septentrionalis Chiapella, because the combination proposed by Tsvelev, D. cespitosa subsp. brevifolia (1974), is an illegitimate name, as a later homonym of D. cespitosa var. brevifolia Griseb. (1852). Other authors (e.g. Aiken et al. 2007; Saarela et al. 2017; Elven et al. 2018) suggested this plant to be treated as a distinct species.

From Europe, Tilletia cerebrina is reported from Scotland (Legon and Henrici 2019), Fennoscandia (Liro 1938; Lindeberg 1959; Jørstad 1963; Gjærum 1972), and mountains in Central, South, and SE Europe (Ciferri 1938; Liro 1938; Vánky 1985a; Schmid-Heckel 1988; Denchev 2001; Scholz and Scholz 2000; Almaraz 2002; Zwetko and Blanz 2004; Klenke and Scholler 2015; Herb. IMI records). In Asia, this smut fungus is known from West Siberia, Wrangel Island, Russian Far East, Kazakhstan, and Uzbekistan (Ramazanova et al. 1987; Govorova 1990; Azbukina and Karatygin 1995; Karatygin et al. 1999; Karatygin 2012); in Africa – only from Madeira (Sydow 1937; Vánky et al. 2011). From North America, T. cerebrina is recorded from Alaska (Cash 1953), Canada (Southampton Island, Fischer 1953; Durán and Fischer 1961), Greenland, western U.S.A. (Washington, Oregon, Idaho, Wyoming, Colorado, and California) and north-central U.S.A. (Minnesota) (Fischer 1953; Durán and Fischer 1961; Farr and Rossman 2019). This fungus is recorded also from Argentina — on Deschampsia cespitosa (Hirschhorn 1964), and Australia — on Aira caryophyllea and A. cupaniana (Vánky and Shivas 2008). Since these host plants are adventive species in Argentina and Australia, T. cerebrina is considered here as a circumpolar–alpine species.

Tilletia cerebrina was initially proposed as a provisional name of a smut fungus on Deschampsia cespitosa from the Rocky Mountains, possessing spores 22–28 µm in diameter (Ellis and Everhart 1887). According to Clinton (1904, 1906), the spore length of the type specimen is chiefly 24–30 µm; according to Fischer (1953), the spore diameter of measured American specimens is 22–28 µm (as in the diagnosis of Ellis and Everhart). The Greenlandic specimen fits well these data; its spores measured (22–)23–28(–29.5) µm in length. European specimens of T. cerebrina on Deschampsia cespitosa, however, possess larger spores. For example, 28–32(–37) × 23–32 µm for a specimen of T. cerebrina on Deschampsia cespitosa from the Southern Carpathians (Romania, Vánky 1985a), and 26–34.5 × 24–33 (30.8 ± 1.5 × 28.7 ± 1.7) µm for a specimen from the Rhodopes (Bulgaria, Denchev 2001). According to Lindeberg (1959), the spore diameter of specimens of T. cerebrina from the Scandes is 27–32 µm. Our measurements of specimens from Scandinavia also confirm this statement. This indicates that a comparative morphological and molecular study of European and American specimens of T. cerebrina is needed.

### Urocystis Rabenh. ex Fuckel, Jahrb. Nassauischen Vereins Naturk. 23–24: 41, 1870 (nom. conserv.). — Type: U. occulta (Wallr.) Rabenh. ex Fuckel.

[Urocystis Rabenh., Klotzsch. Herb. Vivum Mycol., ed. 2, no. 393, 1857 (nom. nud.)]. 

= Tuburcinia Fr., Syst. Mycol. 3(2): 439, 1832 (nom. rej.). — Type: T. orobanches (Mérat) Fr. (q.e. Urocystis orobanches (Mérat) A.A. Fisch. Waldh.). 

= Ginanniella Cif., Fl. Ital. Crypt., Pars 1. Fungi, Fasc. 17, Ustilaginales: 150, 1938. — Type: G. trientalis (Berk. & Broome) Cif. (q.e. Urocystis trientalis (Berk. & Broome) B. Lindeb.).

**Infection** usually systemic. **Sori** mostly in leaves and stems, sometimes in flowers or seeds, less often in roots of both mono- and dicotyledonous host plants, as brown or blackish brown streaks, spots, swellings or galls, containing a mass of spore balls, usually powdery. **Spore balls** persistent, composed of one to many, pigmented, fertile spores, surrounded by paler and smaller sterile cells. **Spore germination** of Tilletia-type. **Anamorph** present in some species. **Host-parasite interaction** by haustoria with enlarged interaction zones. **Septal pore** simple, with membrane caps and two non-membranous plates closing the pore (Vánky 2013).

#### Key to the relevant Urocystis species, based on host plant taxonomy

**Table:** 

**On Cyperaceae**		
On Carex		**U. fischeri**
**On Juncaceae**		
On Juncus		**U. tothii**
**On Poaceae**		
On Leymus		**U. agropyri**
On Trisetum		**U. triseti**
**On Ranunculaceae**		
On Ranunculus		**U. nivalis**
On Thalictrum		**U. sorosporioides**

#### 1(37) Urocystis agropyri (Preuss) A.A. Fisch. Waldh., Bull. Soc. Imp. Naturalistes Moscou 40: 258, 1867. ≡ Uredo agropyri Preuss, in Sturm, Deutschl. Fl., Abt. III, Die Pilze Deutschlands, Bd. 6 (Heft 25–26): 1 and Tabl. 1, 1848. ≡ Polycystis agropyri (Preuss) J. Schröt., Beitr. Biol. Pflanzen 2: 376, 1877. ≡ Tuburcinia agropyri (Preuss) Liro, Ann. Univ. Fenn. Aboëns., Ser. A 1(1): 15, 1922. — Type on Elymus repens (as ‘Queckengrass’), Germany, Sachsen, Hoyerswerda, leg. C.G.T. Preuss.

Figs 1E, 36A–F

= Urocystis preussii J.G. Kühn, in Rabenhorst, Fungi Europ. Exsicc., no. 1898, 1873. — Type on Elymus repens (as ‘Triticum repens’), Germany, near Halle, leg. J. Kühn, s.n.; isotypes in Rabenhorst, Fungi Europ. Exsicc., no. 1898. 

= Tuburcinia elymi Cif., Ann. Mycol. 29: 17, 1931. ≡ Urocystis elymi (Cif.) Schwarzman, Fl. Sporov. Rast. Kazakhstana [Crypt. Fl. Kazakhstan] 2: 317, 1960. — Type on Elymus virginicus L., U.S.A. (syn. by Zundel 1953: 306). 

= Tuburcinia agropyri-juncei Vienn.-Bourg., Soc. Mycol. France 69: 336, 1954 (nom. inval., no Latin diagnosis). ≡ Urocystis agropyri-juncei Vienn.-Bourg. ex H. Zogg, Cryptogamica Helvetica 16: 112, 1986 (nom. inval.). 

**Infection** systemic. **Sori** in leaves as long striae between the veins, initially covered by the epidermis which later ruptures, disclosing a powdery, blackish brown mass of spore balls. **Spore balls** irregular, broadly ellipsoidal, subglobose, ovoid or ellipsoidal, composed of 1–2(–3) central spores (1 = 74.5 %, 2 = 22.3 %, 3 = 3.2 %; n/_1_ = 588) surrounded by a discontinuous to almost continuous layer of sterile cells, (17–)18.5–24(–25) × (14–)15–19(–20) µm (with 1 spore), (20–)21–28(–30.5) × (17–)18–22(–23) µm (with 2 spores). **Sterile cells** broadly elliptical, suborbicular, irregular, elliptical or ovate in outline, sometimes collapsed, (3–)4–11(–12) µm long, pale yellowish to light yellowish brown; cell wall on the side distal to the spores 0.5–0.8 µm thick, on the side proximal to the spores thicker, 0.7–1.0 µm. In SEM cell wall smooth, at the collapsed region often rugulose. **Spores** subglobose, broadly ellipsoidal, irregular, ovoid or ellipsoidal, often slightly flattened on one to few places, (12–)13–17.5(–18.5) × (10–)11–14.5(–15.5) (15.3 ± 1.2 × 13.2 ± 1.0) µm (n/_3_ = 300), medium reddish brown; wall slightly unevenly thickened, 0.7–1.0 µm thick. In SEM spore wall rugulose.

**Figure 36.**
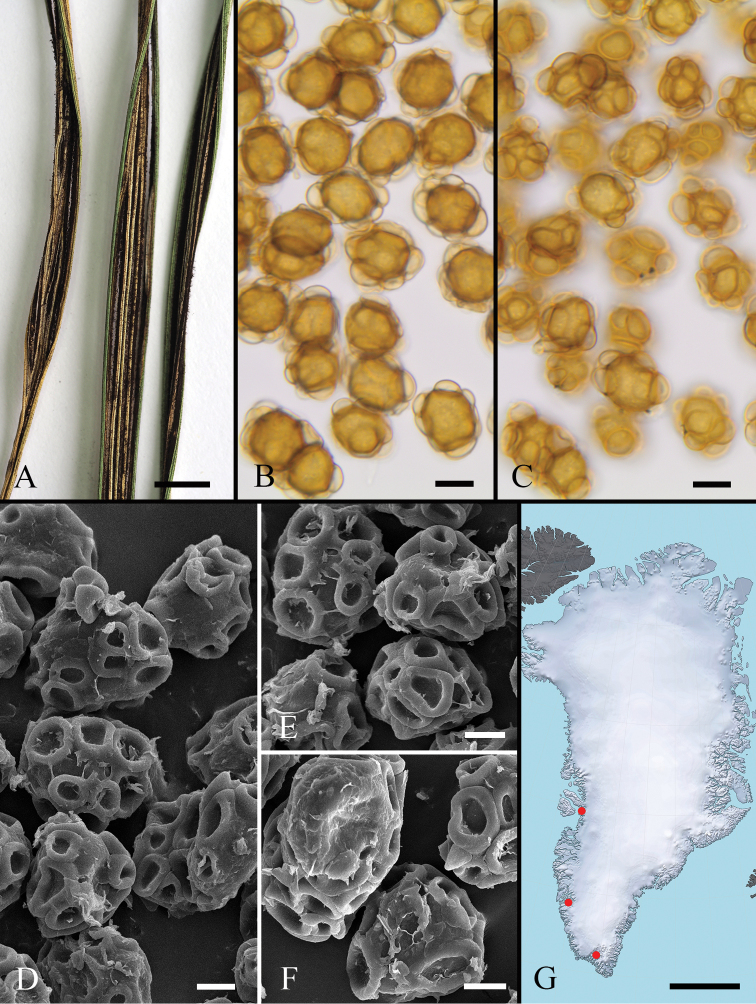
Urocystis agropyri on Leymus arenarius. **A** Habit (C-F-111316) **B, C** spore balls in LM (C-F-111316) **D–F** spore balls in SEM (C-F-102482) **G** distribution map. Scale bars: 0.5 cm (**A**); 10 µm (**B, C**); 5 µm (**D–F**); 500 km (**G**).

**Hosts and distribution within the studied area** — On Poaceae: Leymus arenarius – West Greenland (Fig. 36G).

**Specimens examined or recorded.**

On **Leymus arenarius** (L.) Hochst. (Elymus arenarius L.):

**WG**, Ritenbenk Island (NW of Arve-Prinsens Ejland), eastern and southeastern bays, 69°46'N, July 1890, leg. N. Hartz, s.n. (C-F-102480!, C-F-102481!, the host as ‘E. arenarius’; Rostrup 1891, as ‘E. arenarius’).

**WG**, Ameralik (Lysefjord), lower sidebranch Ameragdla, at Eqaluit, 64°12'N, 50°21'W, 22 Jul 1895, leg. C.H. Ostenfeld, s.n. (C-F-102482!, the host as ‘E. arenarius’).

**WG**, Qassiarsuk, Timmisap Timaa, 61°10'48"N, 45°31'12"W, 12 Aug 2018, leg. H. Knudsen, no. HK 18.089 (C-F-111316!).

**Known hosts** — On Poaceae: Agropyron spp., Elymus spp., Leymus spp., Psathyrostachys juncea (Fisch.) Nevski.

**General distribution**: cosmopolitan.

**Earlier reports from Greenland**: Rostrup (1891), Clinton (1902, 1904, 1906).

**Comments** — Urocystis agropyri s. lat. is a complex of species, recorded on a wide range of grass hosts (43 species, according to Vánky 2011).

#### 2(38) Urocystis fischeri Körn., Hedwigia 16: 34, 1877. ≡ Tuburcinia fischeri (Körn.) Liro, Ann. Univ. Fenn. Aboëns., Ser. A 1(1): 29, 1922. — Lectotype on Carex ‘acuta’ (? C. nigra — s. Liro 1938: 447, and Scholz and Scholz 1988: 353), Germany, near Kassel, 1851, leg. H. Riess, s.n. (UPS) (design. by Lindeberg 1959: 87); isolectotypes in Rabenhorst, Herb. Vivum Mycol., no. 1696 (as ‘Uredo agropyri’).

Fig. 37A–F

= Urocystis caricis Ule, Verh. Bot. Vereins Prov. Brandenburg 25: 215, 1884. — Lectotype on Carex fiacca, Germany, Koburg, W slope of Stiefvater, June 1879, leg. E. Ule, s.n. (as ‘Urocystis agropyri’) (B, design. by Scholz and Scholz 1988: 353) (syn. by Lindeberg 1959: 87). 

**Infection** systemic. **Sori** in leaves as long striae between the veins, initially covered by the epidermis which later ruptures, disclosing a semi-agglutinated, very dark reddish brown mass of spore balls. **Spore balls** irregular, subglobose, ovoid, broadly ellipsoidal or ellipsoidal, composed of 1–2(–3) central spores (1 = 73.0 %, 2 = 23.5 %, 3 = 3.5 %; n/_1_ = 451) surrounded by a continuous, sometimes almost continuous layer of sterile cells, (18–)19–23.5(–26.5) × (14.5–)15.5–19.5(–21) µm (with 1 spore), (20.5–)21.5–31(–34.5) × (17–)18.5–24(–25.5) µm (with 2 spores). **Sterile cells** usually irregular, sometimes broadly elliptical, elliptical or ovate in outline, collapsed, 5–9.5(–11.5) µm long, medium yellowish brown to medium reddish brown; cell wall on the side distal to the spores 0.5–0.7 µm thick, on the side proximal to the spores thicker, 0.8–1.2 μm. In SEM cell wall smooth. **Spores** subglobose, broadly ellipsoidal, slightly irregular or ovoid, sometimes cuneate, sometimes slightly flattened on one to a few places, (13–)14–18.5(–20.5) × (11–)12–15.5(–16.5) (16.0 ± 1.4 × 13.5 ± 1.0) µm (n/_1_ = 100), medium or dark reddish brown; wall slightly unevenly thickened, 0.8–1.4 µm thick. In SEM spore wall smooth.

**Figure 37.**
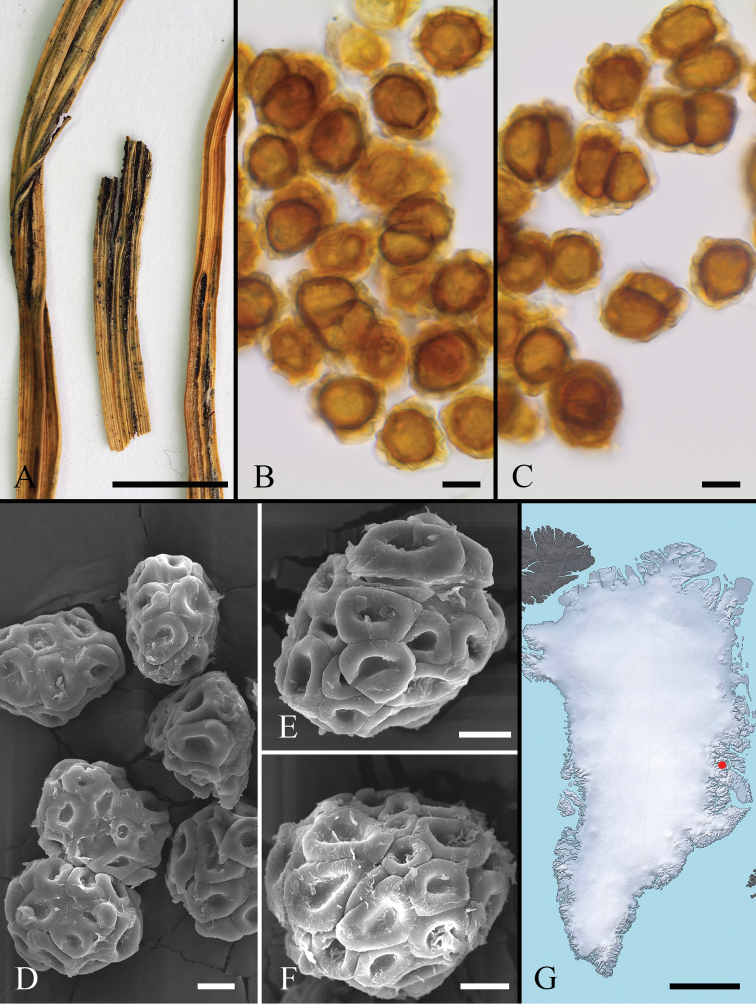
Urocystis fischeri on Carex bigelowii (T. Sørensen, no. 3137, C-Greenland herb., s.n.). **A** Habit **B, C** spore balls in LM**D–F** spore balls in SEM**G** distribution map. Scale bars: 0.5 cm (**A**); 10 µm (**B, C**); 5 µm (**D–F**); 500 km (**G**).

**Hosts and distribution within the studied area** — On Cyperaceae: Carex bigelowii – East Greenland (Fig. 37G).

**Specimens examined or recorded.**

On **Carex bigelowii** Torr. ex Schwein.:

**East Greenland**, Lyells Land, Kap Hedlund, 72°43.6'N, 26°11.2'W, 11 Jul 1932, leg. T. Sørensen, The Three-year expedition to East Greenland 1931–1933 under the leadership of Dr. L. Koch, no. 3137 (C-Greenland herb.!, s.n., the host as ‘C. rigida’).

**Known hosts** — On Cyperaceae: Carex spp.

**General distribution: Europe**: Iceland, Republic of Ireland, UK, Norway, Sweden, Finland, Denmark, Estonia, Lithuania, France, Germany, Poland, Switzerland, Austria, Czech Republic, Slovakia, Romania, Spain, Russia. **Asia**: Iran, Kyrgyzstan, China. **North America**: Canada, Greenland, western U.S.A.

**Comments** — For the first time, description of Urocystis on Carex was published by Fischer von Waldheim (1867: 258). He transferred Uredo agropyri Preuss (a name, erroneously ascribed by Fischer von Waldheim to Persoon) to Urocystis. This combination was proposed in a strange way — by introducing Carex acuta, as a host plant of Urocystis agropyri, but not citing the type host of Uredo agropyri (‘Queckengrass’, q.e. Elymus repens), and by describing Urocystis agropyri on the base of a specimen on Carex (provided with a beautiful, color figure; op. c., Table 3, Fig. 28). Later, this combination was re-proposed (Fischer von Waldheim 1869–1870: 107 and 131), but without a description of the fungus. The fact that Fischer von Waldheim has accepted U. agropyri as a smut fungus both on ‘Agropyrum’ and Carex is apparent from his list of host plants (op. c., 129 and 131). Based on what is written in the latter article and taking into account that the species of Urocystis on Elymus and Carex are distinct species, Körnicke (1877: 34) described a new species on Carex, U. fischeri. Lindeberg (1959), however, considered U. fischeri Körn. as a *nomen nudum* (i.e. as a name of a taxon published without a description or diagnosis or reference to a previously and validly published one), and accepted that Winter (1881) was the first to use a validly published name of a species of Urocystis on Carex. After Lindeberg’s consideration, many authors have treated U. fischeri Körn. as an invalidly published name (e.g., Vánky 1985a, 1994, 2011; Scholz and Scholz 1988; Azbukina and Karatygin 1995; Zwetko and Blanz 2004; Cline and Farr 2006; Guo 2011; Kruse et al. 2013; Klenke and Scholler 2015), accepting the name ‘U. fischeri Körn ex G. Winter’ as correct. The diagnosis of Körnicke, however, provides the minimum necessary information about this fungus: (i) a species that belongs to the genus Urocystis (thus possessing all characteristics of this genus), and (ii) that is specialized on Carex (with designation of C. acuta, as a type host). This descriptive information was sufficiently diagnostic to permit differentiation of this species from the other species in Urocystis at that time, when there was no other Urocystis on Carex. What is given by Winter (1881: 120) as Urocystis fischeri Körn., interpreted by Lindeberg (1959: 87) and some subsequent authors as validation of the name proposed by Körnicke in 1877, is just citation of a previously and validly published name. Additionally, the name in Winter (1881) cannot be considered as a name of a new species because it includes as a synonym another name, Uredo agropyri Preuss, that had been previously and validly published in 1848.

In its current circumscription (Vánky 2011), Urocystis fischeri is known as a polyphagous smut fungus, recorded on 28 species of sedges. In Europe, this fungus is reported on 23 species of sedges (Vánky 1994), with records from the Arctic region (Iceland), Atlantic Europe (Ireland, Scotland, England, Denmark, the Atlantic Pyrenees), Fennoscandia, Northeast (Estonia, Lithuania), Central, Southwest, and East (central part of European Russia) Europe (Rostrup 1903; Lind 1913; Liro 1938 as ‘Tuburcinia fischeri’; Gutner 1941, as ‘Tub. fischeri’; Urban 1958, as ‘Tub. fischeri’; Lindeberg 1959; Jørstad 1963; Jørstad and Gjærum 1966; Mordue and Ainsworth 1984; Vánky 1985a; Zogg 1986; Scholz and Scholz 1988; Azbukina and Karatygin 1995; Almaraz 2002; Helgi Hallgrímsson and Guðríður Gyða Eyjólfsdóttir 2004; Zwetko and Blanz 2004; Karatygin 2012; Kruse et al. 2013; Smith and Lutz 2014). As U. fischeri is distributed on numerous host plants with different habitat preferences, in Europe this smut fungus is recorded from quite different habitats, e.g. grasslands close to the sea, low and medium altitude hay meadows, mountain habitats, alpine and subalpine grasslands. Most of the Central European localities are in the Alps, High Tatras, and Carpathians. In Asia, U. fischeri is known from Iran, Kyrgyzstan, and China (Domashova 1960; Ershad 2009; Guo 2011; Vánky and Abbasi 2013).

In North America, U. fischeri is a rarely recorded smut fungus, reported only from Alberta, Manitoba (Bisby et al. 1938; Fischer 1953), and western U.S.A. (Wyoming, Colorado, and California — Fischer 1953; Herb. BPI records).

Urocystis fischeri is reported here for the first time from Greenland. This record is very interesting as it is the northernmost locality of this fungus (at 72°43'N) and its only locality in the High Arctic.

#### 3(39) Urocystis nivalis (Liro) Zundel, Pennsylvania State Coll. School Agric. Dept. Bot. Contr. 176: 327, 1953. ≡ Tuburcinia nivalis Liro, Ann. Univ. Fenn. Aboëns., Ser. A 1(1): 72, 1922. — Holotype on Ranunculus nivalis, Sweden, Lule Lappmark, Jokkmokk parish, Karranes, 22 Jul 1912, leg. T. Lindfors, s.n. (UPS).

Fig. 38A–F

= Tuburcinia murashkinskyi Cif., Ann. Mycol. 29: 63, 1931. ≡ Urocystis murashkinskyi (Cif.) Zundel, Pennsylvania State Coll. School Agric. Dept. Bot. Contr. 176: 327, 1953. — Holotype on Ranunculus altaicus (as ‘R. frigidus’), Russia, Siberia, Omsk, Altay Mts, 1 Sep 1924, leg. Plotnikov, s.n., comm. K.E. Murashkinsky (Herb. ?) (syn. by Lindeberg 1959: 93). 

**Infection** systemic. **Sori** in stems and leaves, forming swellings, initially covered by the epidermis which later ruptures irregularly, disclosing a powdery, blackish brown mass of spore balls. **Spore balls** usually irregular, composed of 1–5(–7) central spores (1 = 26.6 %, 2 = 36.7 %, 3 = 20.6 %, 4 = 8.8 %, 5 = 4.3 %, 6 = 2.3 %, 7 = 0.7 %; n/_1_ = 738) surrounded by few sterile cells, sterile cells sometimes lacking, (16.5–)18–25(–28) × (11.5–)13–19(–21) µm (with 1 spore), (21–)23–29(–32) × (14–)16–23(–24.5) µm (with 2 spores), (24–)26–34(–37) × (18–)20–27(–29) µm (with 3 spores), (27–)29–39(–41) × (21–)23–29(–31) µm (with 4 spores). **Sterile cells** usually irregular, sometimes broadly elliptical, suborbicular, elliptical or ovate in outline, collapsed, (6–)7–15.5(–17) µm long, medium yellowish brown or medium reddish brown; cell wall on the side distal to the spores 0.5–0.8 µm thick, on the side proximal to the spores thicker, 0.8–1.3 µm. In SEM cell wall smooth to rugulose. **Spores** variable in shape, irregular, subpolyhedral, subglobose, broadly ellipsoidal, ellipsoidal, elongate or ovoid, sometimes cuneate, (12–)13.5–21.5(–23.5) × (10.5–)11.5–15.5(–17) (17.3 ± 2.0 × 13.3 ± 1.2) µm (n/_1_ = 100), medium reddish brown; wall slightly unevenly thickened, 0.8–1.5 µm thick. In SEM spore wall rugulose to minutely verruculose.

**Figure 38.**
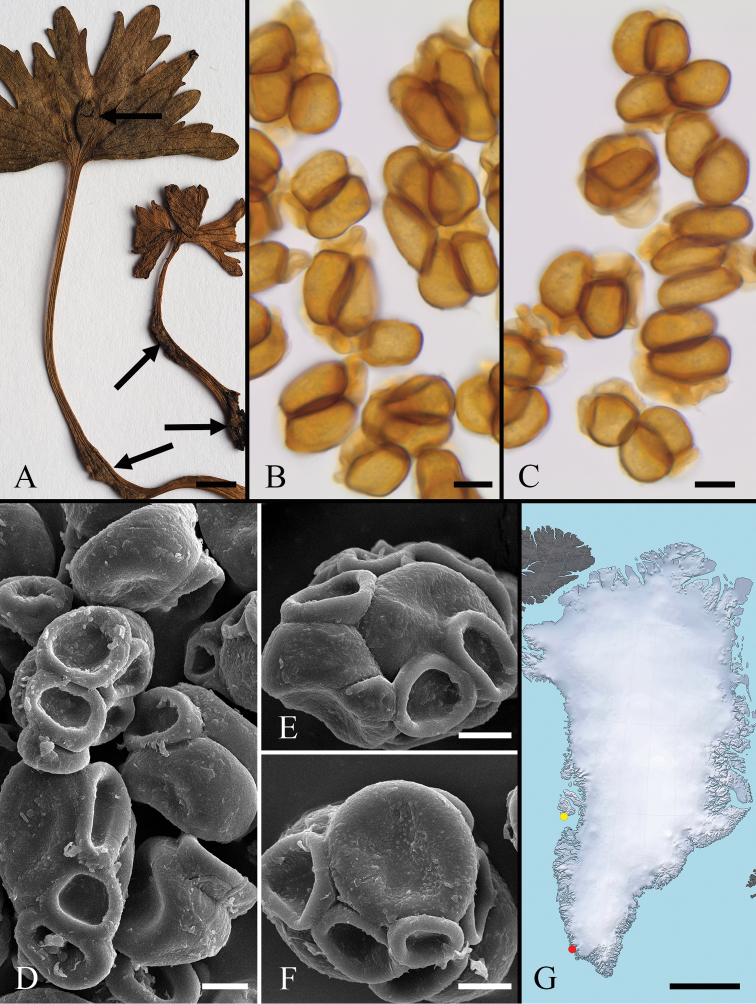
Urocystis nivalis on Ranunculus acris aggr. (C-F-102479). **A** Habit (black arrows show sori) **B, C** spore balls in LM**D–F** spore balls in SEM**G** distribution map (red circle – on Ranunculus acris aggr.; yellow circle – on R. nivalis). Scale bars: 0.5 cm (**A**); 10 µm (**B, C**); 5 µm (**D–F**); 500 km (**G**).

**Hosts and distribution within the studied area** — On Ranunculaceae: Ranunculus acer – West Greenland (Fig. 38G).

**Specimens examined or recorded.**

On **Ranunculus acris** aggr. (R. acer auct.):

**WG**, Tigsaluk, 61°20'N, 8 Aug 1889, leg. N. Hartz, s.n. (C-F-102479!, as ‘Ur. anemones’ on ‘R. acer’; Rostrup 1891, as ‘Ur. anemones’).

On **Ranunculus nivalis** L.:

**WG**, Disko Island, Qeqertarsuaq (as ‘Godhavn’), ‘Skavafjeldet’ (probably, Skarvefjeld, ca 69°17'N, 53°26'W), 15 Aug 1923, leg. E. Ekman, s.n. (n.v., Liro 1938, as ‘Tuburcinia nivalis’).

**Known hosts** — On Ranunculaceae: Ranunculus acris L., R. affinis R. Br., R. albertii Regel & Schmalh., R. altaicus Laxm. (R. frigidus Willd.), R. nivalis L., R. pygmaeus Wahlenb., R. sulphureus Sol., R. trautvetterianusC. Regel ex Ovcz.

**General distribution: Europe**: Fennoscandia. **Asia**: Russia (northeastern Arctic, Far East, Altay Mts), Kazakhstan. **North America**: Greenland.

**Earlier reports from Greenland**: Liro (1938, as ‘Tuburcinia nivalis’).

**Comments** — For the first time from Greenland, this smut fungus was reported by Rostrup (1891) but as Urocystis anemones on Ranunculus acer. Urocystis anemones is specialized on hosts in the genera Anemone, Anemonastrum, and Anemonella and must be removed from the list of the smut fungi in Greenland, as a wrongly identified species. Ranunculus acris L. (R. acer auct. plur.) belongs to a group of taxa, referred to a species aggregate, R. acris aggr. The circumscription of these taxa is not satisfactorily resolved (Elven et al. 2018). The second host plant of Urocystis nivalis, reported from Greenland, is R. nivalis that is a circumpolar species.

Urocystis nivalis is a rarely recorded species. In Europe, it is known from Norway, Sweden, and Finland (mainly, from the northern Fennoscandia — Liro 1938; Lindeberg 1959; Jørstad 1963; Jørstad and Gjærum 1966). From Asia, U. nivalis is recorded from the northeastern Arctic (Kaneliveem River), Russian Far East (Kamchatka Peninsula), Altay Mts, and Kazakhstan (Trans-Ili Alatau Range) (Gutner 1941; Schwarzman 1960; Govorova 1990; Karatygin et al. 1999). In North America, U. nivalis is known only from Greenland. It is a circumpolar–alpine species, belonging to a North American (Arctic)–European (northern) & Asian (northeastern Arctic) & Asian (central) distribution pattern.

#### 4(40) Urocystis sorosporioides Körn. ex Fuckel, Symb. Mycol., Nachtr. 3: 10, 1875. ≡ Tuburcinia sorosporioides (Körn. ex Fuckel) Liro, Ann. Univ. Fenn. Aboëns., Ser. A 1(1): 77, 1922. — Lectotype on Thalictrum minus, Germany, Bonn, Rheinwiesen, 17 May 1874, leg. F. Körnicke, s.n. (Herb. Barb.-Boiss., no. 1852) (design. by Scholz and Scholz 1988: 394).

Fig. 39A–F

[Urocystis sorosporioides Körn., in litt. ad Fuckel (nom. nud.)]. 

**Infection** systemic. **Sori** in petioles and stems, forming up to 1 cm long, usually fusiform swellings, or in leaves, forming irregular swellings, initially covered by the epidermis which later ruptures irregularly, disclosing a powdery, blackish brown mass of spore balls. **Spore balls** irregular, broadly ellipsoidal, subglobose, ellipsoidal, ovoid or elongate, (13.5–)17–43(–50) × (13.5–)15–34(–38) µm, composed of (1–)2–9(–14) central spores (1 = 1.2 %, 2 = 5.2 %, 3 = 8.4 %, 4 = 10.9 %, 5 = 17.9 %, 6 = 18.7 %, 7 = 12.2 %, 8 = 10.2 %, 9 = 6.2 %, 10 = 3.0 %, 11 = 2.7 %, 12 = 1.5 %, 13 = 1.2 %, 14 = 0.7 %; n/_1_ = 403) surrounded by a discontinuous to almost continuous layer of sterile cells. **Sterile cells** usually irregular, sometimes broadly elliptical, suborbicular, elliptical or ovate in outline, collapsed, (4.5–)5.5–12.5(–14) µm long, light or medium yellowish brown; cell wall on the side distal to the spores 0.5–0.7 µm thick, on the side proximal to the spores thicker, 0.7–1.2 µm. In SEM cell wall smooth. **Spores** irregular, subglobose, broadly ellipsoidal, ellipsoidal, elongate or ovoid, sometimes cuneate, (8.5–)9.5–14.5(–16) × (7.5–)8–11(–12) (11.7 ± 1.4 × 9.4 ± 0.9) µm (n/_3_ = 300), medium reddish brown; wall 0.7–1.1 µm thick. In SEM spore wall smooth to rugulose.

**Figure 39.**
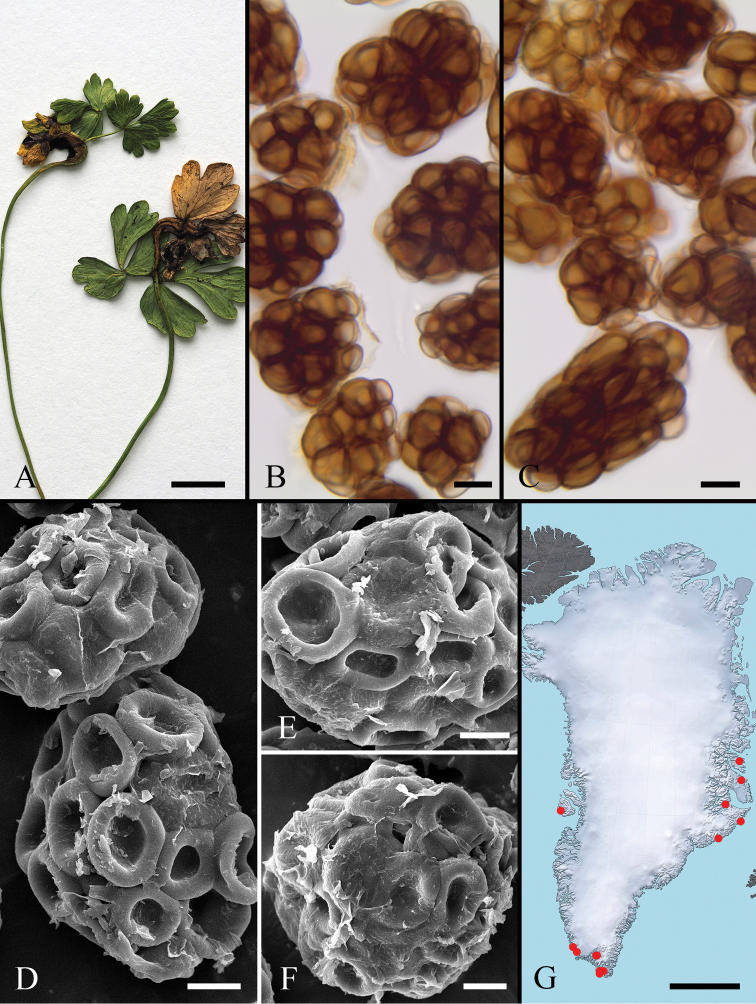
Urocystis sorosporioides on Thalictrum alpinum. **A** Habit (C-F-111318) **B, C** spore balls in LM (C-F-111318) **D–F** spore balls in SEM (leg. J. Just, s.n., C-Greenland herb., s.n.) **G** distribution map. Scale bars: 0.5 cm (**A**); 10 µm (**B, C**); 5 µm (**D–F**); 500 km (**G**).

**Hosts and distribution within the studied area** — On Ranunculaceae: Thalictrum alpinum – West and East Greenland (Fig. 39G).

**Specimens examined or recorded.**

On **Thalictrum alpinum** L.:

**WG**, Disko Island, north coast of Disko Fjord, Kuanitsoroq (as ‘Kvannitsorok’), 69°33'N, 54°17'W, 1902, leg. M. Porsild, no. 833 (C-F-108004!).

**WG**, Tasiusaq, 61°45'N, an. 1889, leg. N. Hartz, s.n. (C-F-102475!; Rostrup 1891); **ditto**, Tasiusaq, 61°45'N, 1889, leg. N. Hartz, s.n. (C-F-102478!; Rostrup 1891).

**WG**, Sermiligârssuk (as ‘Sermiliarsuk’), 61°30'N, 4 Aug 1889, leg. N. Hartz, s.n. (C-F-102476!; Rostrup 1891).

**WG**, Kangilinnguit, 61°12'36"N, 48°07'12"W, 20 Aug 2018, leg. H. Knudsen, no. HK 18.254 (C-F-111318!).

**WG**, Narsarsuaq, 61°10'04"N, 45°24'18"W, 15 Aug 2015, leg. H. Knudsen, no. HK 15.146 (C-F-8297!); **ditto**, Narsarsuaq, 61°09'N, 45°25'12"W, 13 Aug 2018, leg. H. Knudsen, no. HK 18.128 (C-F-111317!).

**WG**, Qassiarsuk (as ‘Kagssiarssuk’), 61°09'N, 45°32'W, 7 Sep 1970, leg. J. Just, s.n. (C-Greenland herb.!, s.n.).

**WG**, Kangerdluk, 60°13'N, 44°19'W, 15 Jul 1966, leg. P. Gravesen & C. Hansen, no. 66-1361 (C-Greenland herb.!, s.n.).

**WG**, Maukarneq, 60°01'N, 44°48'W, 7 Aug 1964, leg. C. Hansen & P.M. Petersen, no. 64-413 (C-Greenland herb.!, s.n.).

**EG**, Geographical Society Island, Husbukta, ca 72°49.7'N, 22°52.5'W, 15 Aug 1930, leg. J. Vaage, s.n. (O!, s.n., as ‘Tub. sorosporioides’; Hagen 1947, as ‘Tub. sorosporioides’).

**EG**, Fleming Fjord (as ‘Fleming Inlet’), approx. 71°45'N, sine dat., leg. N. Hartz, the G. Amdrup’s Expedition to East Greenland in 1898–1900, s.n. (n.v.; not found in C; Rostrup 1904).

**EG**, Danmark Island, Hekla Havn, 70°27'N, 12 Aug 1891, leg. N. Hartz, Expeditio Danica in Groenlandiam orientalem 1891–1892, s.n. (C-F-102477!; Rostrup 1894).

**EG**, Kap Dalton (as ‘Cape Dalton’), ca 69°24.7'N, 24°04'W, sine dat., leg. C. Krusse, the G. Amdrup’s Expedition to East Greenland in 1898–1900, s.n. (n.v.; not found in C; Rostrup 1904).

**EG**, Kong Christian IX Land, Blosseville Kyst, bottom of fjord N of Kap Ravn, 68°33'N, 28°W, 30 Jul 1932, leg. T.W. Bøcher, no. 214 (C-F-108003!).

**Known hosts** — On Ranunculaceae: Thalictrum alpinum, T. aquilegiifolium L., T. fendleri Engelm. ex A. Gray, T. flavum L., T. foetidum L., T. minus L., T. olympicum Boiss. & Heldr., T. petaloideum L., T. polygamum Muhl. ex Spreng., T. simplex L., T. sparsiflorum Turcz. ex Fisch. & C.A. Mey., T. sultanabadense Stapf.

**General distribution: Europe**: Iceland, Faeroes, UK, Norway, Sweden, Finland, Russian Arctic, Denmark, France, Belgium, Netherlands, Germany, Switzerland, Austria, Romania, Ukraine, Spain, Italy, North Macedonia, Bulgaria, central regions of European Russia. **Asia**: Russia (Arctic, W & E Siberia, Far East), Japan, Caucasus, Iran, Kazakhstan, Uzbekistan, Turkmenistan, Kyrgyzstan, Tadzhikistan, Mongolia, China. **North America**: Canada, Greenland, western and northeastern U.S.A.

**Earlier reports from Greenland**: Rostrup (1891, 1894, 1904), Clinton (1902, 1904, 1906), Hagen (1947), Zundel (1953).

**Comments** — The name Urocystis sorosporioides was initially proposed by F. Körnicke, in personal correspondence to L. Fuckel. It was validly published by Fuckel in 1875 in the book version of his ‘*Symbolae mycologicae. Beiträge zur Kenntniss der Rheinischen Pilze.* Dritter Nachtrag.’ (Fuckel 1875: 10). The journal version of the third addendum was published in 1876, in *Jahrbücher des Nassauischen Vereins für Naturkunde* (Fuckel 1876). Some authors (e.g., Lindeberg 1959; Vánky 1985, 1994, 2011; Scholz and Scholz 1988; Azbukina and Karatygin 1995; Azbukina et al. 1995; Guo 2011; Klenke and Scholler 2015) considered this name as a *nomen nudum*, i.e. as a name of a species published without a description or diagnosis. The diagnosis of Fuckel, however, provides the minimum necessary information about the fungus: a species that belongs to the genus Urocystis (thus possessing all characteristics of this genus), and that develops sori on leaves, on their undersurface (hypophyllous). A host plant (Thalictrum minus), type locality (‘Auf der Rheinwiesen bei Bonn’), and collector name (Koernicke) are correctly designated. Thus, the descriptive information provided in the protologue of U. sorosporioides is sufficiently diagnostic to permit recognition of this species from the other species in Urocystis. Even nowadays, this smut fungus continues to be the only representative of the genus Urocystis on hosts in Thalictrum. What is given by Fischer von Waldheim (1877a, b) as ‘Urocystis sorosporioides Koern.’, interpreted by Lindeberg (1959: 98) and some subsequent authors as validation of Körnicke’s name, is just citation of a previously and validly published fungal name.

Urocystis sorosporioides is a circumpolar–alpine species recorded from the Northern Hemisphere on twelve species of Thalictrum. In Europe, this fungus is reported on six species of Thalictrum (Vánky 1994), with records from the Arctic region (Iceland, the northernmost Fennoscandia, Russian Arctic), Atlantic Europe (Faeroes, Scotland, England, Belgium, Netherlands, Denmark), Fennoscandia, Central, South, and SE Europe, Ukraine, central regions of European Russia, and Ural Mts (Blytt 1896; Rostrup 1901, 1903; Lind 1913; Liro 1938; Jørstad 1943, 1963; Urban 1958; Lindeberg 1959; Jørstad and Gjærum 1966; Mordue and Ainsworth 1984; Vánky 1985a; Zogg 1986; Scholz and Scholz 1988; Azbukina and Karatygin 1995; Denchev 2001; Almaraz 2002; Helgi Hallgrímsson and Guðríður Gyða Eyjólfsdóttir 2004; Zwetko and Blanz 2004; Karatygin 2012; Vanderweyen and Fraiture 2014; Klenke and Scholler 2015). As U. sorosporioides is distributed on numerous host plants with different habitat preferences, in Europe this smut fungus is recorded from quite different habitats: alpine and subalpine grasslands (e.g. in the French and Spanish Pyrenees, Alps, Pirin Mts), mesic grasslands, and dry grasslands (e.g. Ponto-Sarmatic steppes in NE Romania and Ukraine). In Asia, U. sorosporioides is known from Russia, Japan, Caucasus, Iran, the Central Asian Republics, Mongolia, and China (Schwarzman 1960; Schmiedeknecht and Puncag 1966; Kakishima 1982; Ramazanova et al. 1987; Govorova 1990; Azbukina and Karatygin 1995; Braun 1999; Karatygin et al. 1999; Guo 2011; Vánky and Abbasi 2013). In North America, U. sorosporioides is a rarely recorded smut fungus, reported only from Ontario (Fischer 1953), western and northeastern U.S.A. (Fischer 1953; Herb. BPI records), and Greenland.

#### 5(41) Urocystis tothii Vánky, Bot. Not. 129: 416, 1976. — Holotype on Juncus compressus, Hungary, Heves Co, near Nagyiván, ‘Hortobágy, Zámpuszta’, alt. ca 90 m, 19 Jun 1974, leg. S. Tóth, s.n. (H.U.V. 3073); isotypes in Vánky, Ustilag. Exsicc., no. 194.

Fig. 40A–E

= Tuburcinia lagerheimii var. obscura Liro, Ann. Univ. Fenn. Aboëns., Ser. A 1(1): 35, 1922. ≡ Urocystis lagerheimii var. obscura (Liro) Zundel, Pennsylvania State Coll. School Agric. Dept. Bot. Contr. 176: 323, 1953. — Holotype on Juncus gerardii, Finland, Nyland, Helsinki, Lill-Bådö, August 1915, leg. J.I. Liro, s.n. (H). 

**Infection** systemic. **Sori** in leaves as long striae between the veins, initially covered by the epidermis that later ruptures, or in interior of culms which later ruptures longitudinally to expose the very dark reddish brown, powdery mass of spore balls. **Spore balls** irregular, broadly ellipsoidal, subglobose, ovoid or ellipsoidal, composed of 1–5(–9) central spores (1 = 10.7 %, 2 = 26.4 %, 3 = 28.3 %, 4 = 18.9 %, 5 = 10.5 %, 6 = 3.3 %, 7 = 0.9 %, 8 = 0.5 %, 9 = 0.5 %; n/_1_ = 428) surrounded by a continuous layer of sterile cells, (14–)15.5–22(–24) × (13.5–)14.5–19(–20) µm (with 1 spore), (19–)20–26(–27.5) × (16–)17–21(–22) µm (with 2 spores), (22–)23–29(–31) × (17.5–)18.5–24(–25) µm (with 3 spores), (25–)27–35(–39) × (22–)23–28(–30) µm (with 4 spores). **Sterile cells** usually irregular in outline, collapsed, (4–)5–11.5(–13) μm long, medium yellowish brown or medium reddish brown; cell wall 0.6–1.0 µm thick. In SEM cell wall rugulose. **Spores** subglobose, irregular, broadly ellipsoidal, ovoid or ellipsoidal, sometimes cuneate, often slightly flattened on one to a few places, (10–)11–16.5(–17.5) × (9.5–)10–13(–14) (13.6 ± 1.3 × 11.4 ± 0.9) µm (n/_1_ = 100), medium or dark reddish brown; wall slightly unevenly thickened, 0.9–1.3 µm thick.

**Figure 40.**
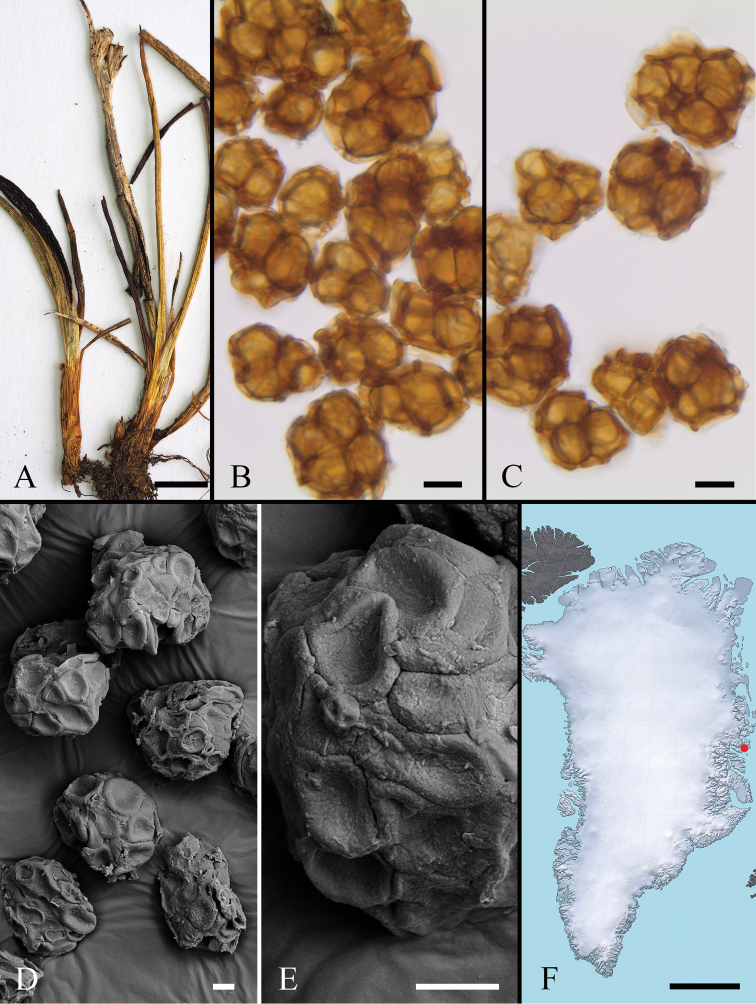
Urocystis tothi on Juncus biglumis (leg. A. Hagen, s.n., O, s.n.). **A** Habit **B, C** spore balls in LM**D, E** spore balls in SEM**F** distribution map. Scale bars: 0.5 cm (**A**); 10 µm (**B, C**); 5 µm (**D, E**); 500 km (**F**).

**Hosts and distribution within the studied area** — On Juncaceae: Juncus biglumis – East Greenland (Fig. 40F).

**Specimens examined or recorded.**

On **Juncus biglumis** L.:

**EG**, Hold with Hope, Myggbukta, near the Norwegian Station (on the north side of Mackenzie Bugt), ca 73°29.4'N, 31 Jul 1933, leg. A. Hagen, the Norwegian Expedition to NE Greenland 1933, s.n. (O!, s.n., as ‘Tub. junci’; Hagen 1947, as ‘Tub. junci’).

**Known hosts** — On Juncaceae: Juncus articulatus L., J. biglumis, J. compressus Jacq., J. gerardii Loisel.

**General distribution: Europe**: Finland, Hungary, Romania. **Asia**: Kazakhstan. **North America**: Greenland. **Australasia**: New Zealand.

**Comments** — Four Urocystis species are known on Juncus: U. johansonii (Lagerh.) Magnus, U. junci Lagerh., U. lagerheimii Bubák, and U. tothii. Of these, U. lagerheimii and U. johansonii can be easily distinguished – the former by the larger spores, (14.5–) 16–24(–27) µm long (Vánky 2011), the latter by the characteristic sorus shape and location, as bulb-like swellings at the basal part of the leaves (Vánky 2011; Denchev and Denchev 2016). Urocystis junci differs from U. tothii by having spore balls composed of (1–)2–10(–23) central spores (based on examination of the type specimen, Vánky 1995: 215), while the spore balls of U. tothii are composed of 1–8(–10) central spores (Vánky 2011).

Among these species, only Urocystis junci has been previously reported from North America: on Juncus biglumis from Greenland (Hagen 1947) and the Canadian Arctic Archipelago (Ellesmere Island, Savile and Parmelee 1964), and on J. balticus Willd. from U.S.A. (Wyoming, Nevada, and Nebraska – Fischer 1953; Zundel 1953; Farr et al. 1989). In the course of the present study, the only available specimen of U. junci from Greenland (recorded by Hagen 1947, as ‘Tuburcinia junci’) was revised and compared with a specimen of U. junci on Juncus filiformis from Bulgaria (SOMF 1975), and with an isotype of U. tothii (Vánky, Ustilag. Exsicc., no. 194). It was found that the Greenlandic specimen possesses spore balls composed of 1–5(–9) spores, corresponding to U. tothii. Thus, Urocystis junci must be removed from the list of the smut fungi in Greenland, as a wrongly identified species. Urocystis tothii is recorded here as a new species for Greenland and North America. Juncus biglumis is a new host of U. tothii.

It is worth noting that the specimens from U.S.A. on Juncus balticus are identified by Fischer (1953) as U. junci, although their spore balls are described as composed of one to several spores, mostly 2–4, that does not match this species. For this reason, a comparative molecular and morphological study of Urocystis on Juncus in U.S.A. and Canada is required.

The record from New Zealand (Vánky and McKenzie 2002) is based on a specimen on Juncus articulatus that is a naturalized species in this country. Since the information about the distribution of U. tothii is insufficient, it is impossible for this fungus to be assigned to any distribution pattern.

#### 6(42) Urocystis triseti (Cif.) Zundel, Pennsylvania State Coll. School Agric. Dept. Bot. Contr. 176: 335, 1953. ≡ Tuburcinia triseti Cif., Ann. Mycol. 29: 14, 1931. — Holotype on Trisetum spicatum, Norway, Troms, Tromsö, August 1900, leg. G. Lagerheim, s.n. (S); isotype H.U.V. 6391.

Fig. 41A–F

**Infection** systemic. **Sori** in leaves as long striae between the veins, initially covered by the epidermis that later ruptures, disclosing a powdery, blackish brown mass of spore balls. **Spore balls** irregular, broadly ellipsoidal, subglobose, ovoid or ellipsoidal, composed of 1–4(–6) central spores (1 = 13.5 %, 2 = 37.9 %, 3 = 32.9 %, 4 = 12.8 %, 5 = 2.2 %, 6 = 0.7 %; n/_1_ = 407) surrounded by continuous, sometimes almost continuous layer of sterile cells, (16–)17–23(–26) × (14.5–)15.5–19(–20) µm (with 1 spore), (18.5–)20–26(–29) × (15–)17–21(–22.5) µm (with 2 spores), (23–)24–33(–36) × (17–)18.5–25(–27) µm (with 3 spores), (26–)28–35(–39) × (21–)22–27(–29) µm (with 4 spores). **Sterile cells** usually irregular, sometimes broadly elliptical, elliptical, suborbicular or ovate in outline, collapsed, (5–)6–12(–14) µm long, medium yellowish brown to medium reddish brown; cell wall on the side distal to the spores 0.5–0.7 µm thick, on the side proximal to the spores thicker, 0.7–1.2 µm. In SEM cell wall punctate to minutely verruculose. **Spores** irregular, broadly ellipsoidal, subglobose, ovoid, ellipsoidal or cuneate, often slightly flattened on a few places, (10.5–)11.5–17.5(–18.5) × (9.5–)10.5–13.5(–14.5) (15.0 ± 1.4 × 12.0 ± 1.0) µm (n/_1_ = 100), medium reddish brown; wall slightly unevenly thickened, 0.8–1.3 µm thick.

**Figure 41.**
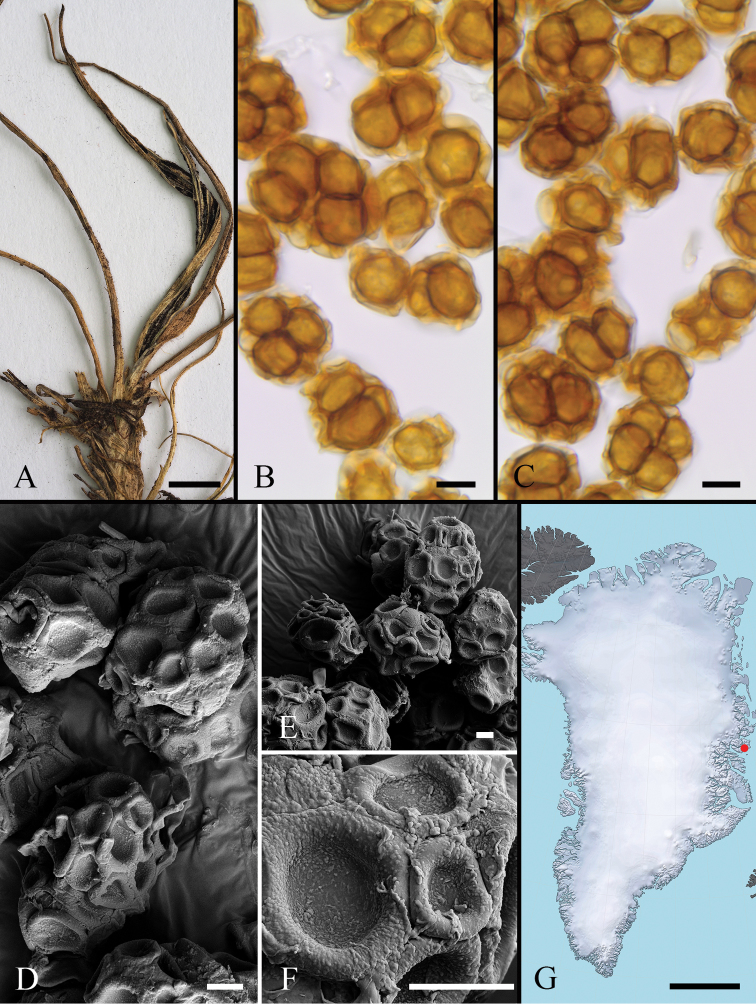
Urocystis triseti on Trisetum spicatum (leg. A. Hagen, s.n., O, s.n.). **A** Habit **B, C** spore balls in LM**D–F** spore balls in SEM**G** distribution map. Scale bars: 0.5 cm (**A**); 10 µm (**B, C**); 5 µm (**D–F**); 500 km (**G**).

**Hosts and distribution within the studied area** — On Poaceae: Trisetum spicatum – East Greenland (Fig. 41G).

**Specimens examined or recorded.**

On **Trisetum spicatum** (L.) K. Richt.:

**EG**, Hold with Hope, Myggbukta (on the north side of Mackenzie Bugt), 73°29.4'N, 19 Aug 1933, leg. A. Hagen, the Norwegian Expedition to NE Greenland 1933, s.n. (O!, s.n., as ‘Tub. agropyri’; Hagen 1947, as ‘Tub. triseti’).

**Known hosts** — On Poaceae: Trisetum alpestre (Host) P. Beauv., T. flavescens (L.) P. Beauv. (T. pratense Pers.), T. spicatum (T. spicatum var. maidenii (Gand.) Fernald, T. subspicatum P. Beauv.).

**General distribution: Europe**: Russian Arctic (Novaya Zemlya), Norway, Sweden, Finland, France, Switzerland, Slovakia, Italy. **North America**: Canada (Nunavut), Greenland, northwestern U.S.A.

**Earlier reports from Greenland**: Hagen (1947, as ‘Tub. triseti’).

**Comments** — Urocystis triseti is a circumpolar–alpine species that is rarely recorded. In Europe, it is known from the Russian Arctic (Novaya Zemlya; Karatygin et al. 1999), Fennoscandia (Liro 1938; Jørstad 1943, 1963; Lindeberg 1959), France, Switzerland, Slovakia, and Italy (Schellenberg 1911, as ‘*U. agropyri*”; Vánky 1985a; Scholz and Scholz 1988; Tomasi 2014). Urocystis triseti is given by Vánky (1994, 2011) as distributed only in Europe, but actually, it is reported also from North America on Trisetum spicatum, as follows: from Canada, Nunavut, Chesterfield Inlet, at ca 63°20'N (Savile 1953; as ‘*U. agropyri*’ on T. spicatum var. maidenii), from East Greenland (Hagen 1947), and from U.S.A., Wyoming, the Medicine Bow Mts (Raper et al. 1954; as ‘*U. agropyri*’). From Nunavut and Wyoming this smut fungus is reported as U. agropyri, but obviously these records are referable to U. triseti, as evidenced by the comments of Savile (1953: 666). The Canadian and Greenlandic finds are located in the Arctic region, furthermore, the Greenlandic locality, at 73°29.4'N, represents the northernmost known record for this fungus.

### Ustilentyloma Savile, in Savile and Parmelee, Canad. J. Bot. 42: 708, 1964 — Type: U. pleuropogonis Savile.

**Sori** in leaves of host plants in the Poaceae, forming spots on the leaf surface. **Spores** single, in loose or compact groups, embedded in the host tissue, not powdery, subhyaline or pale yellow. Resembles Entyloma but germination of Ustilago-type. **Host-parasite interaction** by intercellular hyphae lacking interactions with deposits of specific fungal vesicles. **Septa** with simple pores lacking caps (Vánky 2013).

#### 1(43) Ustilentyloma pleuropogonis Savile, in Savile and Parmelee, Canad. J. Bot. 42: 708, 1964. — Holotype on Pleuropogon sabinei, Canada, Nunavut, Queen Elizabeth Islands, Ellef Ringnes Island, 1.5 mi. SW of Isachsen, 78°46'N, 103°36'W, 15 Jul 1960, leg. D.B.O. Savile, no. 4247F (DAOM 92912!). Paratypes: Canada, Nunavut, Queen Elizabeth Islands, Ellef Ringnes Island, 28 Jul 1960, leg. D.B.O. Savile, no. 4316B (DAOM 92913!); Greenland, Peary Land, Brønlund Fjord, Heilpria Land, 82°10'N, 31°W, 27 Jul 1949, leg. K. Holmen, no. 6675 (DAOM 92914!).

**Sori** in leaves, forming on the leaf surface elongate, scattered, light yellowish brown, 0.5–2 mm long spots. **Spores** embedded in the mesophyll, broadly elliptical or irregularly polygonal, rarely orbicular to suborbicular in outline, 10–21.5 × 8–15 µm, L/w = 1.22, hyaline; wall smooth (after Denchev 1995). **Spore germination** (after Savile and Parmelee 1964: 708) results in a 4-celled, hyaline basidium, ca 23–30 × 5–6.5 µm; producing ellipsoidal, thin-walled, hyaline basidiospores, 6.5–8 × 3–3.5 µm.

For illustration of spores, see Denchev (1995).

**Hosts and distribution within the studied area** — On Poaceae: Pleuropogon sabinei – North Greenland.

**Specimens examined or recorded.**

On **Pleuropogon sabinei** R. Br.:

**NG**, Peary Land, Brønlund Fjord, Heilpria Land, 82°10'N, 31°W, 27 Jul 1949, leg. K. Holmen, no. 6675 (DAOM 92914!).

**Known hosts** — On Poaceae: Pleuropogon sabinei.

**General distribution: Arctic North America**: Canada, Greenland.

**Earlier reports from Greenland**: Savile and Parmelee (1964), Denchev (1995).

**Comments** — Pleuropogon sabinei is a circumpolar species while Ustilentyloma pleuropogonis is known only from the North American High Arctic — the Canadian Arctic Archipelago and North Greenland (Savile and Parmelee 1964; Denchev 1995). In the mycological collections, there are only three specimens of this smut fungus, kept at DAOM and studied by Savile (in Savile and Parmelee 1964) and Denchev (1995). Our efforts to find an infected plant among the specimens of Pleuropogon sabinei in the Greenland Herbarium in Copenhagen were unsuccessful. Since all three specimens of Ustilentyloma pleuropogonis are very scant, the only specimen measured by Denchev (1995) was one of the paratypes, DAOM 92913.

The Canadian and Greenlandic finds are located in the Ellesmere Land – Northern Greenland floristic region (Bay 1992; Elven et al. 2018). The Greenlandic locality on Peary Land, at 82°10'N, is one of the northernmost localities known for a smut fungus.

## Excluded species

### Entyloma caricinum Rostr., Meddel. Grønland 3: 532, 1888.

On **Carex bigelowii** Torr. ex Schwein.:

**East Greenland**, Anoritûp Kangerdlua (as ‘Anoritok’), 61°32'N, 1883–1885, leg. P. Eberlin, s.n. (Rostrup 1888, the host as ‘Carex rigida’).

**Comments** — The holotype was seen by us. There is no smut fungus in this specimen.

## Geographic ranges and distribution of the smut fungi in Greenland

As observed by Nannfeldt (1979: 6), the maximum geographic range of a parasitic fungus is the range of its host (or the combined ranges of its hosts). Parasitic fungi usually have a smaller range than the host plant because of the ecological demands of the fungi and their dispersal efficiency.

Assessment of geographic ranges depends on the accumulation of distribution records. The level of completeness of that information varies among regions. In the discussed case, it is a real problem since the number of records from Greenland, Arctic Canada, Alaska, Siberia, and Arctic Russia is limited. The current assessment is based on existing collections and available literature records of smut fungi from Greenland. Because all smut fungi are associated with plants, the distribution patterns applied here correspond to the phytogeographical patterns of the Arctic plants, as they are circumscribed in Elven et al. (2018): **circumpolar** — “more or less continuous throughout the Arctic and often the northernmost boreal parts of Eurasia and North America (including Greenland) but excluding the temperate mountain ranges”; **circumpolar–alpine** — “circumpolar and with occurrences in one or more temperate mountain ranges south of the boreal zone”; **circumboreal** — “more or less continuous throughout the boreal and often the temperate parts of Eurasia and North America (sometimes including southern Greenland)”; **circumboreal–polar** — “a combined circumboreal and circumpolar pattern, from boreal/temperate to Arctic”. The species referred to as ‘Arctic’ have more restricted distribution in the Arctic than the circumpolar species.

Twelve groups of smut fungi are distributed in Greenland (Table 2):

**Table 2.** Geographic ranges and distribution of the smut fungi in Greenland.

Species	Distribution	High Arctic	Low Arctic	Subarctic
Anthracoidea altera	Arctic	+	+	
Anthracoidea bigelowii	circumboreal–polar	+	+	+
Anthracoidea capillaris	circumboreal–polar	+	+	+
Anthracoidea caricis	circumboreal		+	
Anthracoidea elynae	circumpolar–alpine	+	+	+
Anthracoidea heterospora	bipolar		+	+
Anthracoidea karii	circumboreal–polar		+	+
Anthracoidea limosa	circumboreal–polar		+	
Anthracoidea lindebergiae	circumpolar–alpine		+	
Anthracoidea liroi	circumpolar	+		
Anthracoidea misandrae	circumpolar–alpine	+		
Anthracoidea nardinae	circumpolar–alpine	+	+	
Anthracoidea paniceae	circumboreal	+		
Anthracoidea pseudofoetidae	Arctic–alpine	+		
Anthracoidea rupestris	circumpolar–alpine	+	+	
Anthracoidea scirpi	circumboreal		+	+
Anthracoidea scirpoideae	Amphi-Beringian–North American (N)–Cordilleran			+
Anthracoidea turfosa	Amphi-Atlantic–European		+	
Anthracoidea verrucosa	North American (N)–Cordilleran		+	
Entyloma microsporum	cosmopolitan	+		
Haradaea nivalis	Arctic	+		
Microbotryum arcticum	Arctic	+		
Microbotryum bistortarum	circumboreal–polar	+	+	+
Microbotryum koenigiae	circumboreal–polar	+	+	+
Microbotryum lagerheimii	Amphi-Atlantic–European		+	+
Microbotryum pustulatum	circumboreal–polar	+	+	+
Microbotryum silenes-acaulis	circumpolar–alpine	+	+	+
Microbotryum stellariae	circumboreal–polar		+	+
Microbotryum vinosum	circumpolar–alpine	+	+	+
Orphanomyces arcticus	Arctic–alpine	+		
Planetella lironis	North American (N)	+		
Schizonella elynae	circumpolar–alpine	+	+	+
Schizonella melanogramma	circumboreal–polar	+		
Stegocintractia hyperborea	circumpolar	+	+	
Stegocintractia luzulae	circumboreal			+
Tilletia cerebrina	circumpolar–alpine	+		
Urocystis agropyri	cosmopolitan		+	+
Urocystis fischeri	circumboreal–polar	+		
Urocystis nivalis	circumpolar–alpine		+	+
Urocystis sorosporioides	circumpolar–alpine	+	+	+
Urocystis tothii		+		
Urocystis triseti	circumpolar–alpine	+		
Ustilentyloma pleuropogonis	Arctic	+		
**Total**	–	29	26	19

**circumpolar** (2 species): Anthracoidea liroi and Stegocintractia hyperborea;

**Arctic** (4): Anthracoidea altera, Haradaea nivalis, Microbotryum arcticum (an eastern American Arctic species), and Ustilentyloma pleuropogonis (an eastern American Arctic species);

**circumpolar–alpine** (12): Anthracoidea elynae, A. lindebergiae, A. misandrae, A. nardinae, A. rupestris, Microbotryum silenes-acaulis, M. vinosum, Schizonella elynae, Tilletia cerebrina, Urocystis nivalis, Ur. sorosporioides, and Ur. triseti;

**Arctic–alpine** (2): Anthracoidea pseudofoetidae and Orphanomyces arcticus;

**circumboreal–polar** (10): Anthracoidea bigelowii, A. capillaris, A. karii, A. limosa, Microbotryum bistortarum, M. koenigiae, M. pustulatum, M. stellariae, Schizonella melanogramma, and Urocystis fischeri;

**circumboreal** (4): Anthracoidea caricis s. lat., A. paniceae, A. scirpi, and Stegocintractia luzulae;

**North American** (northern) (1): Planetella lironis;

**amphi-Beringian–North American (northern)–Cordilleran** (1): Anthracoidea scirpoideae;

**North American (northern)–Cordilleran** (1): Anthracoidea verrucosa;

**amphi-Atlantic–European** (2): Anthracoidea turfosa and Microbotryum lagerheimii;

**bipolar** distribution (1): Anthracoidea heterospora;

**cosmopolitan** (2): Entyloma microsporum and Urocystis agropyri.

Urocystis tothii is not included in this analysis (see the comments to this species).

The most numerous distribution groups are the following:

circumpolar–alpine and Arctic–alpine species – 14 species;

circumboreal–polar species – 10;

circumpolar and Arctic species – 6.

With regard to their distribution in Greenland, the established species fall into six groups:

occurring only in North Greenland (2 species): Tilletia cerebrina and Ustilentyloma pleuropogonis;

occurring in North and East Greenland (2): Microbotryum arcticum and Stegocintractia hyperborea;

occurring in North, West, and East Greenland (3): Anthracoidea elynae, A. nardinae, and Microbotryum bistortarum;

occurring only in West Greenland (14): Anthracoidea altera, A. capillaris, A. heterospora, A. limosa, A. lindebergiae, A. scirpi, A. scirpoideae, Entyloma microsporum, Microbotryum lagerheimii, M. stellariae, Planetella lironis, Stegocintractia luzulae, Urocystis agropyri, and Ur. nivalis;

occurring only in East Greenland (9): Anthracoidea liroi, A. paniceae, A. pseudofoetidae, A. turfosa, A. verrucosa, Haradaea nivalis, Urocystis fischeri, Ur. tothii, and Ur. triseti;

occurring in West and East Greenland (13): Anthracoidea bigelowii, A. caricis, A. karii, A. misandrae, A. rupestris, Microbotryum koenigiae, M. pustulatum, M. silenes-acaulis, M. vinosum, Orphanomyces arcticus, Schizonella elynae, S. melanogramma, and Urocystis sorosporioides.

Nine species are relatively widespread in West and East Greenland: Anthracoidea bigelowii, A. elynae, A. nardinae, A. rupestris, Microbotryum bistortarum, M. pustulatum, M. silenes-acaulis, M. vinosum, and Urocystis sorosporioides.

The most widely distributed smut fungi in Greenland are Anthracoidea bigelowii, A. elynae, Microbotryum bistortarum, and M. vinosum.

With regard to the biogeographical zones in the Arctic, the smut fungi established in Greenland occur in the following zones (Table 2):

High Arctic (29 species): Anthracoidea altera, A. bigelowii, A. capillaris, A. elynae, A. liroi, A. misandrae, A. nardinae, A. paniceae, A. pseudofoetidae, A. rupestris, Entyloma microsporum, Haradaea nivalis, Microbotryum arcticum, M. bistortarum, M. koenigiae, M. pustulatum, M. silenes-acaulis, M. vinosum, Orphanomyces arcticus, Planetella lironis, Schizonella elynae, S. melanogramma, Stegocintractia hyperborea, Tilletia cerebrina, Urocystis fischeri, Ur. sorosporioides, Ur. tothii, Ur. triseti, and Ustilentyloma pleuropogonis;

Low Arctic (26 species): Anthracoidea altera, A. bigelowii, A. capillaris, A. caricis, A. elynae, A. heterospora, A. karii, A. limosa, A. lindebergiae, A. nardinae, A. rupestris, A. scirpi, A. turfosa, A. verrucosa, Microbotryum bistortarum, M. koenigiae, M. lagerheimii, M. pustulatum, M. silenes-acaulis, M. stellariae, M. vinosum, Schizonella elynae, Stegocintractia hyperborea, Urocystis agropyri, Ur. nivalis, and Ur. sorosporioides.

Subarctic (19 species): Anthracoidea bigelowii, A. capillaris, A. elynae, A. heterospora, A. karii, A. scirpi, A. scirpoideae, Microbotryum bistortarum, M. koenigiae, M. lagerheimii, M. pustulatum, M. silenes-acaulis, M. stellariae, M. vinosum, Schizonella elynae, Stegocintractia luzulae, Urocystis agropyri, Ur. nivalis, and Ur. sorosporioides.

The highest number of species are found in the High Arctic. Ten species, Anthracoidea bigelowii, A. capillaris, A. elynae, Microbotryum bistortarum, M. koenigiae, M. pustulatum, M. silenes-acaulis, M. vinosum, Schizonella elynae, and Urocystis sorosporioides, are recorded from all three zones.

## Host plants of the smut fungi in Greenland

In the study area, 45 plant species, belonging to 17 genera, were found to be infected by smut fungi (Table 3).

**Table 3.** List of host plant species with their respective smut fungi.

Host plant	Smut fungus
Bistorta vivipara	Microbotryum bistortarum
Bistorta vivipara	Microbotryum pustulatum
Carex atrofusca	Anthracoidea misandrae
Carex bigelowii	Anthracoidea bigelowii
Carex bigelowii	Entyloma caricinum
Carex bigelowii	Urocystis fischeri
hybrids of Carex bigelowii	Anthracoidea bigelowii
hybrids of Carex bigelowii	Anthracoidea heterospora
Carex boecheriana	Anthracoidea capillaris
Carex brunnescens	Anthracoidea karii
Carex canescens	Anthracoidea karii
Carex capillaris	Anthracoidea capillaris
Carex concolor	Anthracoidea bigelowii
Carex deflexa var. deflexa	Anthracoidea caricis
Carex fuliginosa subsp. misandra	Anthracoidea altera
Carex fuliginosa subsp. misandra	Anthracoidea misandrae
Carex fuliginosa subsp. misandra	Schizonella melanogramma
Carex glareosa > see the comments to A. karii	
Carex gynocrates > see the comments to A. karii	
Carex lachenalii	Orphanomyces arcticus
Carex lachenalii > see the comments to A. karii	
Carex macloviana var. macloviana	Anthracoidea verrucosa
Carex maritima	Anthracoidea pseudofoetidae
Carex maritima	Anthracoidea sp.
Carex maritima	Orphanomyces arcticus
Carex maritima	Planetella lironis
Carex misandra > s. C. fuliginosa subsp. misandra	
Carex myosuroides	Anthracoidea elynae
Carex myosuroides	Schizonella elynae
Carex nardina s. lat.	Anthracoidea nardinae
Carex nardina s. lat.	Schizonella melanogramma
Carex nardina subsp. hepburnii	Anthracoidea nardinae
Carex nigra	Anthracoidea heterospora
Carex parallela subsp. parallela	Anthracoidea turfosa
Carex parallela > see the comments to A. karii	
Carex rariflora	Anthracoidea limosa
Carex rupestris subsp. rupestris	Anthracoidea rupestris
Carex rupestris	Schizonella melanogramma
Carex scirpoidea subsp. scirpoidea	Anthracoidea scirpoideae
Carex simpliciuscula	Anthracoidea lindebergiae
Carex stans > see Carex concolor	
Carex subspathacea	Anthracoidea liroi
Carex vaginata	Anthracoidea paniceae
Deschampsia arctica > see Deschampsia brevifolia	
Deschampsia brevifolia	Tilletia cerebrina
D. cespitosa subsp. septentrionalis > s. D. brevifolia	
Elymus arenarius > see Leymus arenarius	
Elyna myosuroides > see Carex myosuroides	
Juncus biglumis	Urocystis tothii
Kobresia myosuroides > see Carex myosuroides	
Kobresia scirpina > see Carex myosuroides	
Kobresia simpliciuscula > see Carex simpliciuscula	
Koenigia islandica	Microbotryum koenigiae
Leymus arenarius	Urocystis agropyri
Luzula confusa	Stegocintractia hyperborea
Luzula multiflora	Stegocintractia luzulae
Luzula nivalis	Stegocintractia hyperborea
Lychnis alpina > see Viscaria alpina	
Oxyria digyna	Microbotryum vinosum
Pleuropogon sabinei	Ustilentyloma pleuropogonis
Polygonum viviparum > see Bistorta vivipara	
Ranunculus acris aggr. (R. acer auct.)	Urocystis nivalis
Ranunculus nivalis	Urocystis nivalis
Ranunculus pygmaeus	Entyloma microsporum
Sagina nivalis	Haradaea nivalis
Scirpus cespitosus > see Trichophorum cespitosum	
Silene acaulis	Microbotryum silenes-acaulis
Silene uralensis subsp. arctica	Microbotryum arcticum
Stellaria borealis subsp. borealis	Microbotryum stellariae
Stellaria calycantha	Microbotryum stellariae
Stellaria crassipes	Microbotryum stellariae
Thalictrum alpinum	Urocystis sorosporioides
Trichophorum cespitosum subsp. cespitosum	Anthracoidea scirpi
Trisetum spicatum	Urocystis triseti
Viscaria alpina	Microbotryum lagerheimii

Only plants belonging to six families (Cyperaceae, Poaceae, Juncaceae, Ranunculaceae, Caryophyllaceae, and Polygonaceae), out of a total of 55 in the flora of Greenland, are infected by smut fungi. Cyperaceae is the plant family with the highest number of hosts (23 species) and with the highest percentage of the infected species, compared to the total number of the species in Greenland (39 %) (Table 4). Carex is the genus with the highest number of host species (22). Both Carex maritima and C. fuliginosa subsp. misandra are infected by three smut fungi, while each of the following five plants, Bistorta vivipara, Carex bigelowii, C. myosuroides, C. nardina, and C. rupestris, is a host of two smut fungi. The total number of the host plants (45 species) is 8.5 % out of a total of 532 vascular plants in the flora of Greenland (Christian Bay, in prep.).

**Table 4.** Comparison of the number of host species to the number of the species in Greenland.

Families	Number of host species	Number of species in Greenland	% of species infected
Cyperaceae	23	59	39
Poaceae	4	66	7
Juncaceae	4	18	22
Ranunculaceae	4	16	25
Caryophyllaceae	7	30	23
Polygonaceae	3	9	33
Total	45		

## Conclusions

The present work is a first monographic treatment of the smut fungi of Greenland, one of the insufficiently studied areas in the world regarding this taxonomic group of parasitic fungi. The purpose of this investigation was to improve the taxonomic knowledge about the smut fungi of Greenland and the Arctic.

A total of 43 species belonging to 11 genera on 45 host plants (making 56 smut-host combinations) are distributed in Greenland. Two species, Anthracoidea pseudofoetidae and Urocystis tothii are newly recorded from North America. Thirteen species, Anthracoidea altera, A. capillaris, A. limosa, A. liroi, A. pseudofoetidae, A. scirpoideae, A. turfosa, Microbotryum lagerheimii, M. stellariae, Schizonella elynae, Stegocintractia luzulae, Urocystis fischeri, and Ur. tothii, are reported for the first time from Greenland. Three fungus-host combinations, Anthracoidea capillaris on Carex boecheriana, Anthracoidea pseudofoetidae on Carex maritima, and Urocystis tothii on Juncus biglumis, are new for science. Five plants are reported as new hosts of smut fungi already known from Greenland: Carex nigra for Anthracoidea heterospora, Carex canescens for Anthracoidea karii, Carex fuliginosa subsp. misandra for Anthracoidea misandrae, Carex maritima for Orphanomyces arcticus, and Carex fuliginosa subsp. misandra for Schizonella melanogramma. Three species previously reported in the literature, Microbotryum violaceum s. str. (recorded as ‘Ustilago violacea’), Urocystis anemones, and U. junci, are removed from the list of the smut fungi of Greenland as wrongly identified. Additional distribution records are given for 12 species hitherto known from Greenland: Anthracoidea bigelowii, A. caricis, A. elynae, A. lindebergiae, A. misandrae, A. nardinae, A. rupestris, A. scirpi, Schizonella melanogramma, Stegocintractia hyperborea, Urocystis agropyri, and Ur. sorosporioides.

## Nomenclatural novelties

### New combination

**Table:**

**Carex macroprophylla subsp. subfilifolia** (T.V. Egorova, Jurtzev & V.V. Petrovsky) Denchev & T. Denchev		37
